# Broad‐Spectrum, Cell Envelope‐Active Marinocyclin Antibiotics From a Coral‐Derived Bacterium Are Effective Against Colistin‐Resistant Bacteria

**DOI:** 10.1002/anie.7705008

**Published:** 2026-09-03

**Authors:** Clara Chepkirui, Sandra Silva, Jan Straetener, Chandrashekhar Padhi, Tina Keller‐Costa, Tess Lamer, Laura Reinke, Heike Brötz‐Oesterhelt, Rodrigo Costa, Jörn Piel

**Affiliations:** ^1^ Institute of Microbiology Eidgenössische Technische Hochschule (ETH) Zürich Zurich Switzerland; ^2^ iBB – Institute for Bioengineering and Biosciences and i4HB – Institute for Health and Bioeconomy, Instituto Superior Técnico Lisbon Portugal; ^3^ Department of Bioengineering Instituto Superior Técnico Lisbon Portugal; ^4^ Interfaculty Institute of Microbiology and Infection Medicine, Microbial Bioactive Compounds, University of Tübingen Tübingen Germany; ^5^ Cluster of Excellence “Controlling Microbes to Fight Infection” Tübingen Germany; ^6^ German Center for Infection Research (DZIF) Braunschweig Germany

**Keywords:** antibacterial activity, antibiotics, biology, microbiology, natural product, peptidoglycan

## Abstract

The marine bacterial genus *Aquimarina* comprises diverse members with numerous natural product biosynthetic gene clusters but few characterized compounds. Here we report a novel class of lipopeptides with exceptional antibiotic activity, named marinocyclins, isolated from *Aquimarina megaterium* EL43 associated with the octocoral *Eunicella labiata*. The major congener marinocyclin A exhibited potent and uniform activity against a broad panel of drug‐resistant gram‐negative and gram‐positive pathogens, including ESKAPE bacteria. The natural product efficiently compromised the outer and inner bacterial membranes, leading to rapid cell permeabilization and lysis. This activity profile was mediated by the ability to bind lipopolysaccharides, anionic phospholipids enriched in bacterial membranes, and peptidoglycan precursors. Eukaryotic cytotoxicity required higher doses than antibacterial activity. Genomic data suggest a nonribosomal biosynthetic origin for marinocyclins. These findings position marinocyclins as a promising new scaffold for antibiotic development and highlight the potential of *Aquimarina* spp. as a source of novel antibiotics. Further medicinal chemistry optimization of marinocyclins could enhance their prokaryotic selectivity to generate leads for treating infections caused by drug‐resistant pathogens.

## Graphical abstract

Marinocyclins, polycationic macrocyclic depsipeptides, were discovered by genome mining from the marine coral‐derived bacterium *Aquimarina megaterium*. The compounds have broad‐spectrum activity against gram‐positive and gram‐negative ESKAPE bacteria. They interact with anionic lipopolysaccharide, phosphatidylglycerol, cardiolipin, and lipid II, disrupting the integrity of the outer and inner membranes. Their antibacterial activity is more homogeneous than that of polymyxins and is not affected by prominent colistin resistance determinants.

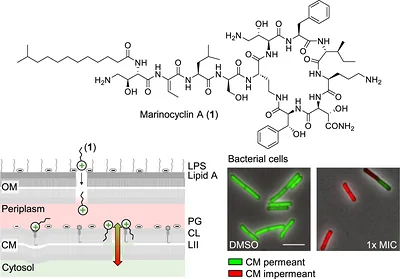

## 1 Introduction

Bacterial natural products have historically provided the foundation for most antibiotic classes in clinical use [1]. The rise of antimicrobial resistance has created an urgent, currently unmet need for new antibiotic leads, particularly against therapeutically challenging Gram‐negative pathogens [2]. While the well‐established bacterial natural product sources increasingly yield already known compounds [3], ample evidence exists that the genome‐guided exploration of underexamined bacterial lineages and habitats reveals chemical novelty that could reinvigorate antibiotic discovery [4].

Such studies previously suggested members of the bacterial genus *Aquimarina* (family *Flavobacteriaceae*, phylum *Bacteroidota*) as a rich but largely unexplored potential source of new antibiotics [5]. *Aquimarina* spp. are Gram‐negative, rod‐shaped marine bacteria frequently isolated from seawater, marine sediments, and from eukaryotic hosts such as algae, sponges, corals, and shellfish, where they occur in complex host‐associated microbial communities or epibiotic biofilms [5, 6, 7, 8]. To date, 39 species have been validly described, and additional candidate species lacking cultured representatives were detected by metagenomic DNA sequencing [9].

Polyketide biosynthesis genes were first detected in the genus *Aquimarina* by homology‐based screening of cultivated, sponge‐associated bacteria [10], stimulating targeted research on novel natural products from this taxon. Research in our (J.P. and R.C.) labs provided the polyketide cuniculene [11] and the antibiotic nonribosomal peptide–polyketide hybrids aquimarins [12] as the first characterized natural products from *Aquimarina*. Cuniculene and aquimarins are produced by strains Aq349 and Aq135, respectively, that were both isolated from marine sponges [10] and are now formally described as the type strains of the new species *Aquimarina spinulae* and *Aquimarina aquimarini* [13]. A recent large‐scale genome mining survey identified *Aquimarina* as the most promising source of putatively novel natural products within the family *Flavobacteriaceae*, highlighting this marine genus as an emerging resource for natural product research [5]. Its potential for new antibiotics was further underscored recently by a study that appeared during the preparation of the manuscript for this work and reported the chemical synthesis of hypothetical, genome‐predicted lipopeptides that yielded compounds active against diverse drug‐resistant bacterial pathogens [14]. However, the structures of the actual natural products remain unknown.

Here we report the discovery of marinocyclins, novel lipopeptide antibiotics from *Aquimarina megaterium* EL43 cultivated from the octocoral *Eunicella labiata* [15, 16]. Marinocyclin A showed potent activity against all tested bacterial strains, including challenging drug‐resistant gram‐negative pathogens. This rare activity profile might bear promise as a new therapeutic lead.

## 2 Results and Discussion

### 2.1 Genome‐Guided Identification of Marinocyclins

In our analyses of *Aquimarina* strains, we observed that crude acetone extracts from *A. megaterium* EL43 exhibited antibacterial activity against *Escherichia coli* and *Bacillus subtilis*. This strain was therefore selected for further studies. Subsequent antiSMASH [17] analysis of the *A. megaterium* EL43 genome sequence [9, 16] revealed the presence of ten biosynthetic gene clusters (BGCs). Closer inspection suggested arylpolyene lipids, a siderophore with high similarity to fulvivirgamides [18], carotenoids, a type III PKS product, ribosomally synthesized and posttranslationally modified peptides (RiPPs), nonribosomal lipopeptides, and the previously characterized polyketide cuniculene as products of these BGCs (Table S1). We focused on the lipopeptides as potential products of the single NRPS BGC in the genome, since this compound class is relevant as clinical antibiotics and the architecture of the gene cluster suggested that the biosynthetic product was new. The 46.5 kb NRPS locus (Figure 1, Table S2) showed low architectural similarity to characterized clusters from the MIBiG database and contained two core NRPS biosynthetic genes jointly encoding one loading and 11 extension modules in addition to a predicted oxygenase and further genes. Prediction of the incorporated amino acids by antiSMASH and PRISM 4 [19] suggested the presence of several non‐proteinogenic residues (Figure 1, Table S3). PRISM 4 also suggested the presence of an N‐terminal fatty acyl moiety with ten carbons, which for the total predicted structure would result in a monoisotopic mass in the range of *m/z* 1382 or 1364 Da for linear or cyclic peptide forms, respectively (Figure 1). However, it was unclear how close these values were to the actual peptide masses, since we did not expect the fatty acid prediction to be of high confidence.

**FIGURE 1:**
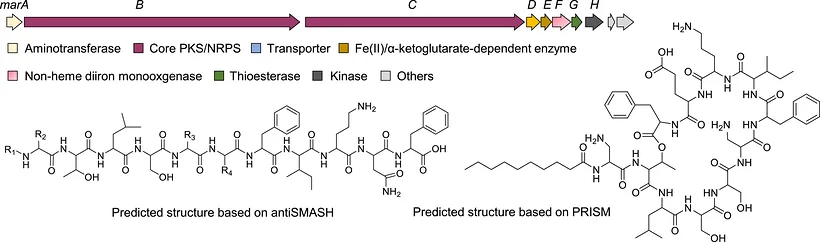
The NRPS BGC identified in the genome of the coral‐associated *A. megaterium* EL43 (top) and structures predicted by antiSMASH 8.0.4 and PRISM 4 (bottom).

Analysis of the crude extracts by high‐performance liquid chromatography coupled to heated electrospray ionization high‐resolution mass spectrometry (HPLC‐HESI‐HRMS) revealed a group of compounds with molecular ions at *m/z* 1450–1500 (Figure S1). Their tandem MS (MS^2^) fragmentation pattern suggested a cyclic peptide portion and an open‐chain moiety containing a dehydrated Thr (dehydrobutyrine, Dhb), a Leu or Ile, and a Ser residue in series (Figure S1), matching the antiSMASH as well as the PRISM predictions (Thr, Leu, Ser). These compounds were therefore target candidates and isolated by repeated reverse‐phase HPLC, yielding seven congeners, named marinocyclins A‐G (**1–7**) (Figure 2). These were subjected to structure elucidation via NMR in conjunction with the HPLC‐HESI‐HRMS data.

**FIGURE 2:**
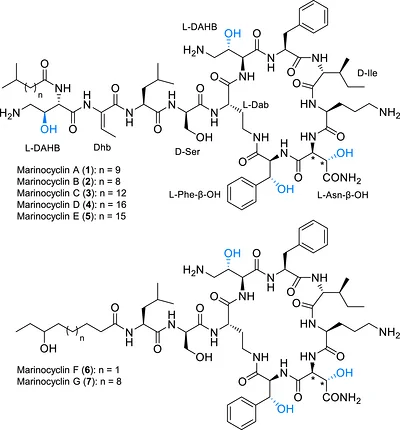
Structures of marinocyclins A‐G (**1–7**) isolated from *A. megaterium* EL43. The configuration of Asn‐β‐OH at positions marked by an asterisk (*) was assigned based on genomic data (absolute configuration at the α position) and the elution order of FDVA derivatized amino acid monomers reported in the literature [20, 21] for a similar amino acid residue (relative configuration at the β position). Amino acid hydroxylations are highlighted in blue.

### 2.2 Structure Elucidation of Marinocyclins

Marinocyclin A (**1**, Figure 2) with the molecular formula C_71_H_114_N_16_O_18_ established from the HPLC‐HESI‐HRMS data (*m/z* 1479.8567 [M+H]^+^, *m/z* 740.4312 [M + H]^2+^) (Figure S2) was obtained from cell pellet extracts. Signals corresponding to the α‐methine of amino‐acid residues (δ 4.14–5.03) and phenyl groups (δ 7.20–7.40) were recorded in the ^1^H NMR spectrum (Figures S5 and S10, Table S4). The HSQC spectrum suggested several methylenes, methines, oxymethines, and aliphatic methyl groups (Figure S7 and S14, Table S4). Analysis of COSY and HMBC data (Figures S6, S8, S9, S11, and S15) revealed the presence of eleven amino acid residues, including four non‐proteogenic ones. The peptide moiety consisted of two unusual 2,4‐diamino‐3‐hydroxybutyrate (DAHB) residues in addition to dehydrobutyrine (Dhb), leucine (Leu), serine (Ser), 2,4‐diaminobutyrate (Dab), phenylalanine (Phe), isoleucine (Ile), ornithine (Orn), β‐hydroxyasparagine (Asn‐β‐OH), and β‐hydroxyphenylalanine (Phe‐β‐OH) units (Figure 2, Table S3). The connection of Leu/DAHB to Dhb was established from the HMBC correlations of Leu α‐methine proton (δ 4.19) to DhB C‐1 (δ 167.5) and the NOE correlations of Dhb ‐NH proton (δ 9.62) to both Leu (δ 7.93) and DAHB (δ 8.14) ‐NH protons and DAHB‐H‐3 (δ 3.95) (Figures S9, S12, S13, and S15). This was further supported by the MS^2^ data (Figures S3 and S4). HMBC correlations of the α‐methine protons to the C‐1 carboxyl carbon in conjunction with the NOESY correlations of the NH protons to the α‐methine protons were used to determine the sequence of the amino acids (Figures S8 and S15). HMBC correlations of the Dab C‐4 amine protons to the Phe‐β‐OH C‐1 carbonyl and NOESY correlations of the Dab C‐4 NH proton (δ 7.34) to the α‐methine proton of Phe‐β‐OH (δ 4.19) confirmed macrocyclic ring formation through condensation of the C‐terminal Phe β OH residue and the Dab amino group (Figure S9 and S15). From the HRESI‐MS data, a fatty acid acyl moiety with 13 carbons was predicted for the observed mass. The fatty acyl chain was elucidated as an 11‐methyldodecanoyl moiety based on the HMBC and COSY NMR data. The NMR‐derived structure of **1** agreed with the MS^2^ fragmentation (Figures S3 and S4) that had yielded ions corresponding to a linear tetrapeptide connected to the N‐terminal fatty acyl moiety and the cyclic heptapeptide core. Attempts to linearize **1** at basic conditions resulted in the isomerization of **1**, but no MS ions corresponding to linear products were detected (Figure S16). Similarly, chymotrypsin digest of **1** did not yield a linear product (Figure S16).

Marinocyclin B (**2**) isolated from the pellet extracts had the molecular formula C_70_H_112_N_16_O_18_ deduced from the HPLC‐HESI‐HRMS data (*m/z* 1465.8485 [M + H]^+^, *m/z* 733.4265 [M + H]^2+^) (Figure S17). A mass difference of ‐14 Da compared to **1** and a similar MS^2^ fragmentation pattern suggested a similar structure between the two compounds, with **2** having one less methylene unit (Figures S18 and S19). The 1 and 2D NMR data (Figures S20–S23, Table S5) of **2** were similar to those of **1**, with the difference localized to the fatty acid chain based on the integration factor of the methylene multiplet (δ 1.28–1.32). Therefore, the lipid moiety in **2** was determined as a 10‐methylundecanoyl unit. Further marinocyclin variants, named marinocyclin C (**3**), D (**4**), and E (**5**), which were isolated from the pellet extract as minor congeners, had the deduced molecular formulae C_74_H_120_N_16_O_18_ (*m/z* 1521.8739 [M + H]^+^, *m/z* 761.4402 [M + H]^2+^) and C_78_H_128_N_16_O_18_, (*m/z* 1577.9371 [M + H]^+^, *m/z* 789.4710 [M + H]^2+^), and C_77_H_126_N_16_O_18_ (*m/z* 1563.9220 [M + H]^+^, *m/z* 782.4636 [M+H]^2+^), respectively (Figures S24, S31, and S43). MS^2^ fragmentation patterns and NMR data (Figures S25–S30, S32–S42, and S44–S49, Tables S6–S8) of these compounds were similar to those of **1**, with the difference localized to the fatty acyl chain. Based on the molecular formulae of **3**, **4**, and **5**, these compounds differ from **1** by additional C_3_H_6_, C_7_H_14_, and C_6_H_12_ units, respectively. By MS^2^, fragments with *m/z* 355.26, 411.36, and 397.31 were attributed to fatty acid‐DAHB fragments of **3**, **4**, and **5**, respectively, were recorded (Figures S25–S26, S32–S33, and S44–S45). Therefore, the structures of **3**, **4**, and **5** were concluded to contain 14‐methylpentadecanoyl, 18‐methylnonadecanoyl, and 17‐methyloctadecanoyl lipidations, respectively (Figure 2).

Marinocyclin F (**6**) and G (**7**), with the predicted molecular formulae C_58_H_91_N_13_O_16_ (*m/z* 1226.6368 [M+H]^+^, *m/z* 613.8220 [M+H]^2+^) and C_65_H_105_N_13_O_16_ (*m/z* 1324.7191 [M + H]^+^, *m/z* 662.8633 [M + H]^2+^) (Figure 2, Figures S50 and S55), were isolated as further minor congeners from the pellet extracts. Comparison with the 1D and 2D NMR data of **1** revealed for **6** the absence of signals for Dhb and the N‐terminal DAHB (Figures S51–S54, Table S9). This suggested **6** as a peptide similar to **1,** with the first two amino acids missing but carrying a fatty acyl moiety. The latter was elucidated as 6‐hydroxyoctanoic acid from the COSY and HMBC correlations (Figures S52 and S54, Table S9). The position of the hydroxy group was confirmed based on the HMBC correlations of the terminal methyl group (δ 0.90) to C‐7 (δ 33.7) and C‐6(δ 77.0). Although we were able to isolate marinocyclin G (**7**), the amount was insufficient to acquire good NMR data. Its proton NMR and HSQC spectra were superimposable with those of **6** (Figures S56–S58). The predicted molecular formula differs from that of **6** by +C_7_H_14_. The MS^2^ data indicated similar structures for the two molecules, with **7** having a 13‐hydroxypentadecanoyl moiety.

In an MS^2^ molecular ion network analysis of *A. megaterium* crude extracts, several additional compounds clustered together with the identified marinocyclins (Figure S1), suggesting further congeners, but their titers were too low for isolation (Table S10).

Superficially, the marinocyclin structures resemble those of polymyxins. However, marinocyclins are distinguished by the presence of several β‐hydroxylated amino acid residues and a linear tetrapeptide chain. Additional similarities to reported natural products exist to the turnercyclamycin lipopeptide antibiotics from the shipworm symbiont *Teredinibacter turnerae* that also contain several β‐hydroxylated amino acid residues including the rare DAHB unit [20]. Otherwise, turnercyclamycins differ from marinocyclins.

### 2.3 Stereochemical Assignment by Advanced Marfey's Analysis

To determine the absolute configuration, marinocyclin A (**1**) was hydrolyzed, and the resulting mixture was derivatized with *N*_α_‐(2,4‐dinitro‐5‐fluorophenyl)‐l‐valinamide (FDVA) and subjected to HPLC‐HESI‐HRMS analysis. The results were compared to the FDVA‐derivatized DAHB, Leu, Ser, Dab, Phe, Ile, Orn, and Phe‐β‐OH standards. This analysis revealed the presence of l‐configured Leu, Dab, Phe, and Orn (Figure S61, Table S3). Ser and Ile retention times matched those of the corresponding d‐configured standards (Figure S61, Table S3). The retention time of derivatized Phe‐β‐OH was identical to that of the tested (*2S,3R*)‐3‐hydroxy‐3‐phenylalanine FDVA standard.

To determine the configuration of DAHB, a diastereomeric mixture of (*2S,3S*)‐ and (*2R,3S*)‐3‐DAHB in a 73:27 ratio was synthesized from hydroxyectoine as the starting material (Figure S60) [22]. The FDVA derivatives of these diastereomers eluted at slightly different retention times and could be distinguished by peak height ratios. The retention time of the major diastereomer, assigned as the (*2S,3S*)‐3‐hydroxy‐2,4‐diaminobutyrate FDVA derivative, matched that of the derivatized DAHB unit of the natural product **1**.

To assign the stereochemistry of Asn‐β‐OH, the acid hydrolysate of **1** was derivatized with both L‐FDVA and D‐FDVA, and the elution order was compared to those reported in the literature [23] due to the lack of authentic standards. Because Asn‐β‐OH residues are converted to Asp‐β‐OH in the hydrolysis step, the retention times of the derivatized hydrolysate of **1** were compared with those of both l/d‐FDLA‐d‐*threo*‐Asp‐β‐OH (7.2‐7.3 min) and l/d‐FDLA‐d‐*erythro*‐Asp‐β‐OH (10.0–10.5 min) as reported by Miller et al. [20] and Li et al. [21]. Our analyte retention times (6.81 and 7.14 min) were close to those of the *threo* form. Without a standard, it was difficult to ascertain the configuration of the α carbon because of the very similar retention times reported for the α‐l‐ and α‐d‐ *threo* forms (0.1 min retention time difference). Therefore, the orientation at the α carbon was assigned based on the genomic data as l, since the corresponding NRPS region contained neither an epimerase domain nor an epimerizing condensation (C) domain (see further below), and the specificity of the extending C domain was determined as l (^L^C_L_ domain) [24].

The dehydrobutyrine (Dhb) olefinic double bond was assigned as *E*‐configured due to observed NOESY correlations of the methyl protons to the leucine NH proton and of the α proton (δ 5.75) to the DAHB amide proton (δ 4.37). Electronic circular dichroism (ECD) curves of **1**, **2**, and **4** were also acquired. Comparable Cotton effects in the range of 190–270 nm were recorded for all compounds (Figure S62), suggesting similar stereochemistry for the isolated compounds.

### 2.4 Proposed Biosynthesis of Marinocyclins

The *A. megaterium* EL43 genome harbored only one NRPS BGC, which was a convincing candidate for marinocyclin biosynthesis. The cluster (Figure 3, Table S2), named *mar*, contains the two genes *marB* and *marC* that together encode a loading module and 11 NRPS extension modules, mirroring the fatty acyl starter and 11 amino acid residues in marinocyclins A‐E. The structure suggested by PRISM, based on the predicted adenylation domain substrate specificities, largely agreed with the residues elucidated by NMR (Table S3). The only divergences were Dab instead of Ser, DAHB, Asn‐β‐OH, and Phe‐β‐OH, which all carry additional hydroxyl groups compared to the PRISM prediction, and Dhb being a dehydrated version of the predicted Thr. The differences for the hydroxylated residues might be due to oxygenation at a later biosynthetic stage. Candidate enzymes for the hydroxylations are the free‐standing enzymes MarE homologous to Fe(II)/α‐ketoglutarate enzymes, which are often responsible for amino acid hydroxylations in peptides [25, 26, 27], and MarF with similarity to CmlA‐type non‐heme diiron monooxygenases. Although the latter enzymes are less common in secondary metabolism, CmlA was shown to catalyze β‐hydroxylation of a peptidyl carrier protein‐bound l‐*p*‐aminophenylalanine residue in chloramphenicol biosynthesis [28].

**FIGURE 3:**
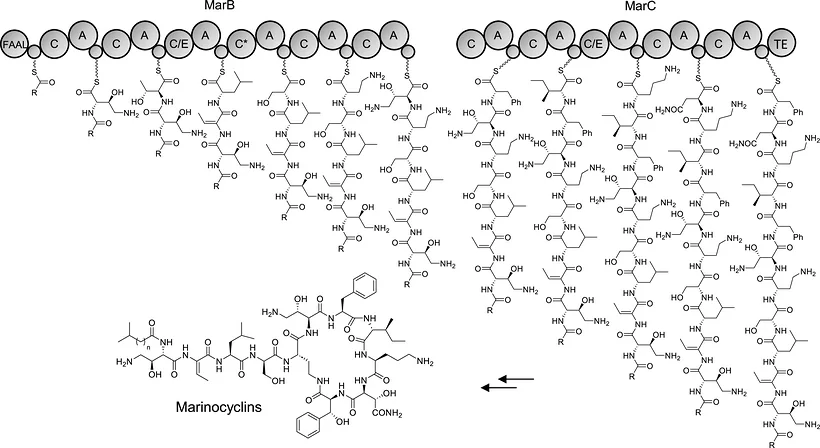
The *mar* NRPS and proposed biosynthetic model. The timing of the four amino acid hydroxylations is speculative and was hypothesized based on predicted A domain selectivities. A, adenylation domain; C, condensation domain; C*, dehydrating condensation domain (*C*_modAA_); C/E dual condensation/epimerization domain FAAL, fatty acyl ACP ligase; R, fatty acyl residue; TE, thioesterase domain. Small circles are carrier protein domains.

To determine whether MarE and MarF can hydroxylate the free amino acids Dab, Asn, and Phe, or if these enzymes act at a later biosynthetic stage, MarE and MarF were each cloned and heterologously expressed in *E. coli* with N‐terminal polyhistidine tags. MarE was mostly insoluble, even when expressed as an N‐terminal small ubiquitin‐like modifier (SUMO) fusion protein, while MarF was highly soluble in *E. coli* with a simple His tag (Figure S63). Incubation of purified MarE and α‐ketoglutarate with each of l‐Dab, l‐Asn, and l‐Phe found no hydroxylated products after Fmoc‐derivatization and LC‐MS analysis (Figure S64). Similarly, incubation of purified MarF, methyl viologen, and each amino acid with either sodium thionite or NADH as electron donors yielded no hydroxylated products after Fmoc derivatization and LC‐MS analysis (Figure S65). These results suggest that MarE and MarF act on these amino acid substrates after they are bound to the NRPS, or potentially on the PCP‐bound or free peptides, as is the case in other pathways using homologs of these enzymes [29, 30, 31]. Since these experiments were not successful, the role of the *mar* BGC in marinocyclin biosynthesis remains putative even if no other NRPS or matching ribosomal peptide BGC was identified in the genome.

While no epimerization domains were identified, two NRPS extension modules (5, and 9) harbor dual epimerization/condensation (E/C) domains [32] that match the Marfey's data (Figure S61, Table S3). These C/E domains are likely responsible for the epimerization of the Ser and Ile residues incorporated by the respective upstream modules. Another divergent C domain with similarity to dual elongation/dehydration domains (*C*_modAA_ domains) [33] is located in extension module 3, collinear to the location of the Dhb residue in the peptide structure.

Among the remaining biosynthetic enzymes encoded by the BGC, MarA resembles aminotransferases and might generate Dab from aspartate semialdehyde. The latter metabolite is generated in primary metabolism from aspartate via carboxylate phosphorylation and reduction. MarH with homology to aspartate kinases might increase the titer of the phosphorylated intermediate.

### 2.5 Marinocyclin A (**1**) Shows Broad‐spectrum Activity and Homogeneous Potency Across Bacterial Genera

The major congener marinocyclin A (**1**) was tested for antimicrobial activity against a panel of clinically important bacterial pathogens. We observed broad and potent activity against both Gram‐positive and Gram‐negative WHO priority pathogens [34], with minimal inhibitory concentrations (MICs) of 2 to 8 µg/mL across the entire ESKAPE panel (Figures S66–S68, Table 1, Tables S12 and S13). This includes *Enterococcus faecium*, *Staphylococcus aureus*, *Klebsiella pneumoniae*, *Acinetobacter baumannii*, *Pseudomonas aeruginosa*, and *Enterobacter* spp. Susceptible reference strains and multidrug‐resistant clinical isolates were covered with comparable efficacy. As the fatty acylated cyclic heptapeptide of **1** bears superficial structural resemblance to polymyxins [35], we assessed polymyxin B and polymyxin E (colistin) side by side. Marinocyclin A stands out by unusually homogeneous potency across the full spectrum of bacterial isolates tested. Notably, *E. coli* and *K. pneumoniae* strains carrying globally disseminated polymyxin resistance determinants, plasmid‐borne *mcr‐1* [36, 37] or chromosomal *pmr* mutations [38], remained fully susceptible to **1** (Figure S69, Table 1, Table S13).

**TABLE 1:** Minimum inhibitory concentrations (MICs, µg/mL) of marinocyclin and comparator antibiotics determined by broth microdilution according to CLSI (Clinical Laboratory Standards Institute) guidelines; upper part, activity against *B. subtilis* and representatives of the ESKAPE panel; lower part, activity against strains carrying the colistin‐resistance determinant *mrc‐1*.

Strains		Resistance		MIC (µg/mL)	
			1	Polymyxin B	Colistin
*Bacillus subtilis* 168			2–4	4–8	16–32
*Staphylococcus aureus* ATCC 29213			4–8	64	> 64
*Staphylococcus aureus* USA300		MRSA^a^	4–8	32–64	> 64
*Enterococcus faecium* BM 4147‐1			4	> 64	> 64
*Enterococcus faecium* 6011		VRE^b^	2–4	16	> 64
*Escherichia coli* ATCC 25922			4	0.5	≤ 0.06–0.25
*Escherichia coli* Tü21‐10		Multires^c^	2–4	≤ 0.06	0.125
*Escherichia coli* Tü21‐13		Multires.	2–4	≤ 0.06–0.125	≤ 0.06–0.125
*Escherichia coli* Tü21‐32		Multires.	2–4	≤ 0.06	< 0.06
*Escherichia coli* Tü21‐34		Multires.	2–4	≤ 0.06	< 0.06
*Klebsiella pneumoniae* ATCC 12657			2–4	0.5	≤ 0.06–0.125
*Klebsiella pneumoniae* ATCC 27799		Multires.	2–4	≤ 0.06	≤ 0.06–0.125
*Enterobacter aerogenes* ATCC 13048			4	0.25–1	≤ 0.06—0.25
*Enterobacter aerogenes* UR9466		Multires.	4	≤ 0.06‐0.125	0.25–1
*Acinetobacter baumannii* 09987			2–4	0.5–1	0.5–2
*Acinetobacter baumannii* I‐7486		Multires.	2	≤ 0.06–0.125	0.25–1
*Pseudomonas aeruginosa* ATCC 27853			4	2	0.5
*Pseudomonas aeruginosa* B63230		Multires.	4	≤ 0.06–0.25	4–8
*Escherichia coli* J53			4–8	0.125–0.25	≤ 0.06–0.125
*Escherichia coli* J53 pTOPO			4–8	≤ 0.06–0.125	≤ 0.06
*Escherichia coli* J53 pTOPO_*mcr‐1*_prom		Colistin^R^ ^d^	4–8	0.25–1	1
*Escherichia coli* J53 TC pKP2442		Colistin^R^	4–8	2–4	2–4
*Klebsiella pneumoniae* KP2442		Colistin^R^	8	4–8	8

*Note*: Values represent concentration ranges reproducible across ≥ 3 biological replicates.

^a^ MRSA: methicillin‐resistant *S. aureus*.

^b^ VRE: vancomycin‐resistant enterococci.

^c^ Multires.: multiresistant.

^d^ Colistin^R^: colistin‐resistant. Plasmid pKP2442 carries *mrc‐1* [36].

### 2.6 Marinocyclin A (**1**) Destroys the Integrity of the Gram‐Negative Outer Membrane

The comparable activity of **1** against gram‐positive and Gram‐negative bacteria suggested a conserved target structure and implied that **1** can cross the Gram‐negative outer membrane. The asymmetric outer membrane, composed of an external lipopolysaccharide (LPS) leaflet and an internal phospholipid layer, forms a permeability barrier to many antibiotics. Charged small molecules < 600 Da typically translocate via porins, but due to its substantially higher molecular weight, this route is blocked to **1**. The cyclic, polycationic scaffold of **1**, containing three free amino groups spaced by hydrophobic amino acids and extended by a lipophilic tail, agrees with a capability for “self‐promoted uptake” [39]. Therefore, we assessed the capacity of **1** to permeabilize the outer membrane of *E. coli* using the lipopolysaccharide‐impermeant dye 1‐*N*‐phenylnapthylamine (NPN). The fluorescent dye has a low quantum yield in aqueous solution but emits bright fluorescence when interacting with phospholipids in the cytoplasmic membrane. As NPN cannot pass through an intact lipopolysaccharide layer, it can be used to detect its breach [40]. **1** triggered a strong, concentration‐dependent NPN influx at doses well below the MIC, indicating efficient disruption of the outer membrane and outperforming both polymyxin B and colistin (Figure 4A). Based on what is known for polymyxins, which serve as a reference for self‐promoted uptake [39, 41], we propose that the positively charged Dab and DAHB groups of **1** bind to the negative phosphates in the lipid A and core oligosaccharide region of LPS, thereby displacing Ca^2+^ and Mg^2+^ and weakening the lateral interactions in the LPS layer. Thereby, the hydrophobic tail of **1** or non‐polar motifs of the macrocycle reach the hydrophobic region of the LPS molecules, allowing **1** to pass through the weakened outer membrane.

**FIGURE 4:**
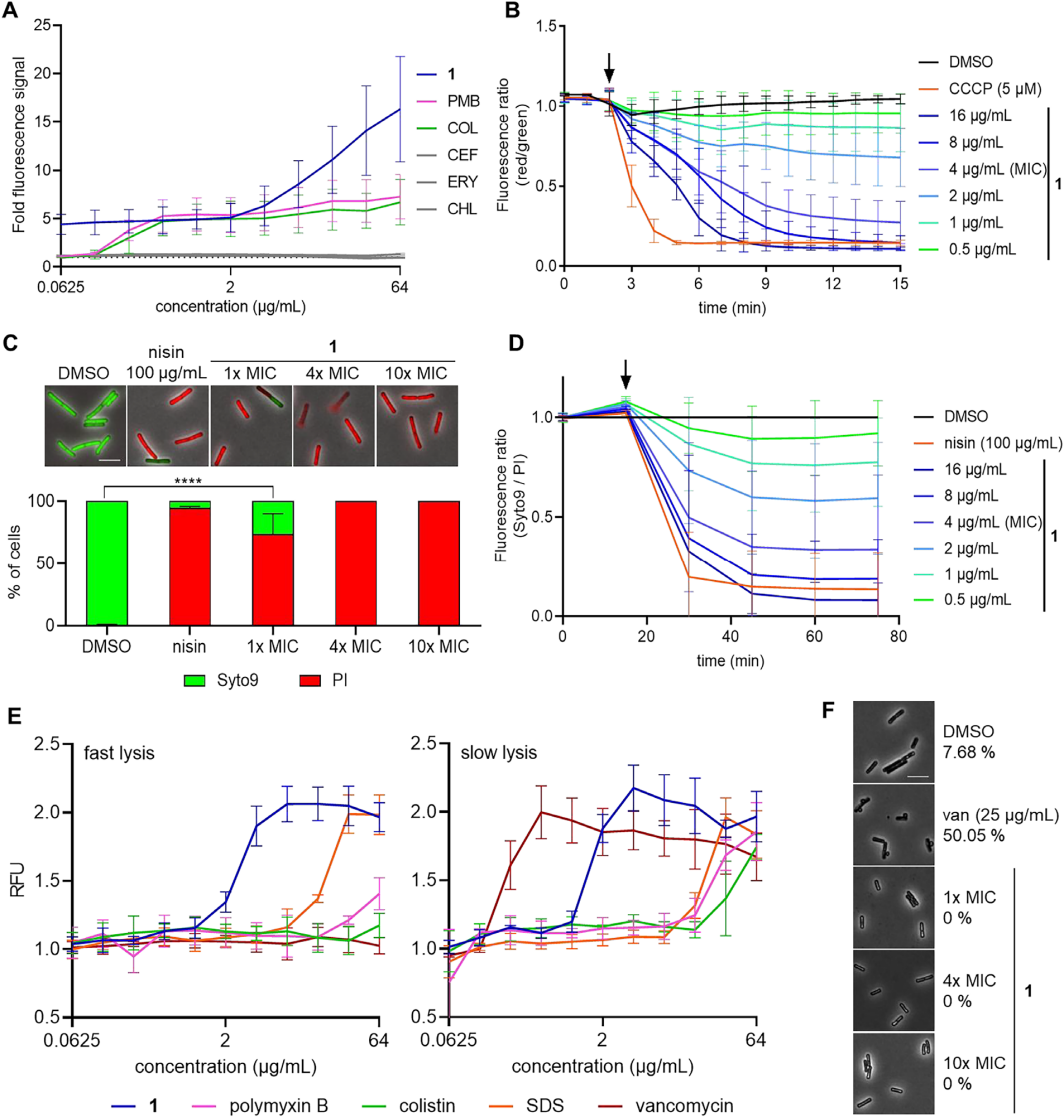
Marinocyclin A (**1**) rapidly disrupts bacterial membranes. (A) Outer membrane permeabilization of *E. coli* measured by NPN uptake (*n* = 3, normalized to the untreated control, ± SD). Polymyxin B (PMB) and colistin (COL) served as positive controls; cefoxitin (CEF), erythromycin (ERY), and chloramphenicol (CHL) as negatives. (B) Cytoplasmic membrane depolarization in *B. subtilis* assessed by DiOC_2_(3) (*n* = 3, ± SD). CCCP as positive, DMSO (1%) as negative control. Arrow marks compound addition. (C) Single‐cell imaging of *B. subtilis* membrane permeabilization after 30 min using Syto9 (green, membrane‐permeant) and propidium iodide (red, impermeant for intact membranes). Scale bar, 5 µm. Nisin as positive, DMSO (1%) as negative control; bar chart: quantification of > 100 cells/condition (*n* = 3, ± SD; ****p < 0.0001, two‐way ANOVA). (D) Time‐ and concentration‐resolved cytoplasmic membrane permeabilization in *B. subtilis* by spectroscopic readout of the Syto9/PI green‐to red fluorescence ratio (*n* = 3, ± SD). Nisin as positive, DMSO (1%) as negative control. (E) Rapid lysis of *B. subtilis* at MIC‐level marinocyclin, compared with detergent SDS (positive control for fast lysis, after 5 min) and vancomycin (positive control for slow lysis, after 60 min), *n* = 3, ± SD. (F) Membrane blebbing in *B. subtilis* after 30 min, indicative of peptidoglycan weakening, occurred for vancomycin but not for **1**. Scale bar, 5 µm. Percent blebbing was quantified from > 100 cells per replicate and condition (*n* = 3). Vancomycin as positive, DMSO (1%) as negative control.

### 2.7 Marinocyclin A (**1**) Rapidly Disrupts the Integrity of the Bacterial Cytoplasmic Membrane

Upon reaching the periplasm, **1** has access to the cytoplasmic membrane. To investigate the impact on the cytoplasmic membrane independently of the outer membrane, we used the Gram‐positive bacterium *Bacillus subtilis* as a model. A panel of isogenic, luciferase‐based bioreporter strains, which collectively inform on the stress pathways most frequently perturbed by antibiotics [42, 43], revealed modest but reproducible activation of marker genes for general envelope stress (*P_ypuA_‐luc*) and for interference with undecaprenyl phosphate cycling (*P_liaI_‐luc*) (Figure S70), pointing to the cytoplasmic membrane as a primary site of action in *B. subtilis*. In contrast, the reporters for DNA and RNA stress and ribosome stalling were not induced. Consistent with this, **1** elicited dose‐dependent cytoplasmic membrane depolarization within minutes, as quantified by 3,3'‐diethyloxacarbocyanine iodide (DiOC_2_(3)) staining (Figure 4b). To distinguish ionophoric activity from physical membrane disruption, we tested uptake of propidium iodide (PI), a membrane‐impermeant dye that fluoresces only upon binding to DNA. Bright intracellular fluorescence appeared after 15 min at 1× MIC marinocyclin A (Figure 4c,d), demonstrating loss of cytoplasmic membrane integrity. We corroborated these findings using the *B. subtilis* strain BAU‐102, which reports cell lysis through the release of periplasmic β‐galactosidase. The assay protocol enables differentiation between cell wall synthesis inhibitors that release β‐galactosidase only after prolonged exposure and compounds with surfactant activity, which show an almost immediate release. **1** caused a robust signal after short‐term exposure, its strength matching that of SDS and substantially exceeding that of polymyxins (Figure 4e), consistent with rapid membrane rupture. By contrast, even after exposure of *B. subtilis* to **1** at 10× MIC for 30 min, we did not observe any indication of peptidoglycan lesions. In this assay, cells are incubated with the bioactive compounds before being fixed with acetic acid/methanol. If peptidoglycan integrity is impaired, the cytoplasmic membrane bulges through peptidoglycan lesions, and these membrane “blebs” are captured by the fixative and imaged. No blebs appeared in the marinocyclin‐treated sample (Figure 4f). This result concurs with the notion that the cytoplasmic membrane forms the target of **1**, because membrane disruption relieves the turgor pressure required for bulging.

The uniform, broad‐spectrum activity of **1** implies that a) the outer membrane does not form a rate‐limiting barrier and b) the cytoplasmic membrane is the key bactericidal target, consistent with the rapid loss of cytoplasmic membrane integrity at the MIC. Also, polymyxins cause progressive cytoplasmic membrane destabilization, but less effectively than **1** [39, 41]. The remarkably homogeneous potency of **1** across phylogenetically distinct gram‐positive and Gram‐negative species contrasts with clinically used cyclic peptide antibiotics. Colistin is restricted to gram‐negative bacteria, and daptomycin to Gram‐positives. Interestingly, during the completion of our study, a publication by Li et al. reported the chemical synthesis of hypothetical lipopeptide natural products predicted from *Aquimarina* spp. BGCs, which included the *mar* BGC [14]. One synthetic peptide inspired by the *mar* cluster, termed aquicidine L, exhibited comparably potent activities against gram‐negative bacteria but was less active against Gram‐positive strains. However, the predicted compound differed from the actual natural product in five amino acid positions, a non‐cyclic structure, and the fatty acyl moiety. A synthetic macrocyclic variant of aquicidine L with a cyclization topology distinct from that of **1** displayed the highest activities against Gram‐positive bacteria. These two complementary activities are exquisitely combined in the natural peptide.

### 2.8 Marinocyclin A (**1**) Requires Higher Concentrations and Longer Exposure Times to Inhibit Eukaryotic Cells

To determine whether marinocyclin also compromises the cytoplasmic membrane integrity of eukaryotic cells, we assessed propidium iodide (PI) entry into the cytoplasm of the human lung adenocarcinoma cell line A549 (Figure 5a,b). Following 30 min of exposure to 64 µg/mL of **1**, approximately 30% of the eukaryotic cells showed PI fluorescence, but this trend did not yet reach statistical significance. By contrast, a 16‐fold lower concentration (4 µg/mL) applied for only 15 min was sufficient to permeabilize 75% of *B. subtilis* cells (Figure 4c). To evaluate cytotoxic effects after prolonged exposure, we measured metabolic activities of three different eukaryotic cell lines after 24 h of incubation with **1**. Under reducing conditions, characteristic of viable, metabolically active cells, the dye resazurin is enzymatically reduced to fluorescent resorufin. This conversion was inhibited by **1**, with half‐maximal inhibitory concentrations (IC_50_) of 15–30 µg/mL (Figure 5c), values that are approximately 4–10 times higher than the MIC values determined for diverse bacterial species (Table 1).

**FIGURE 5:**
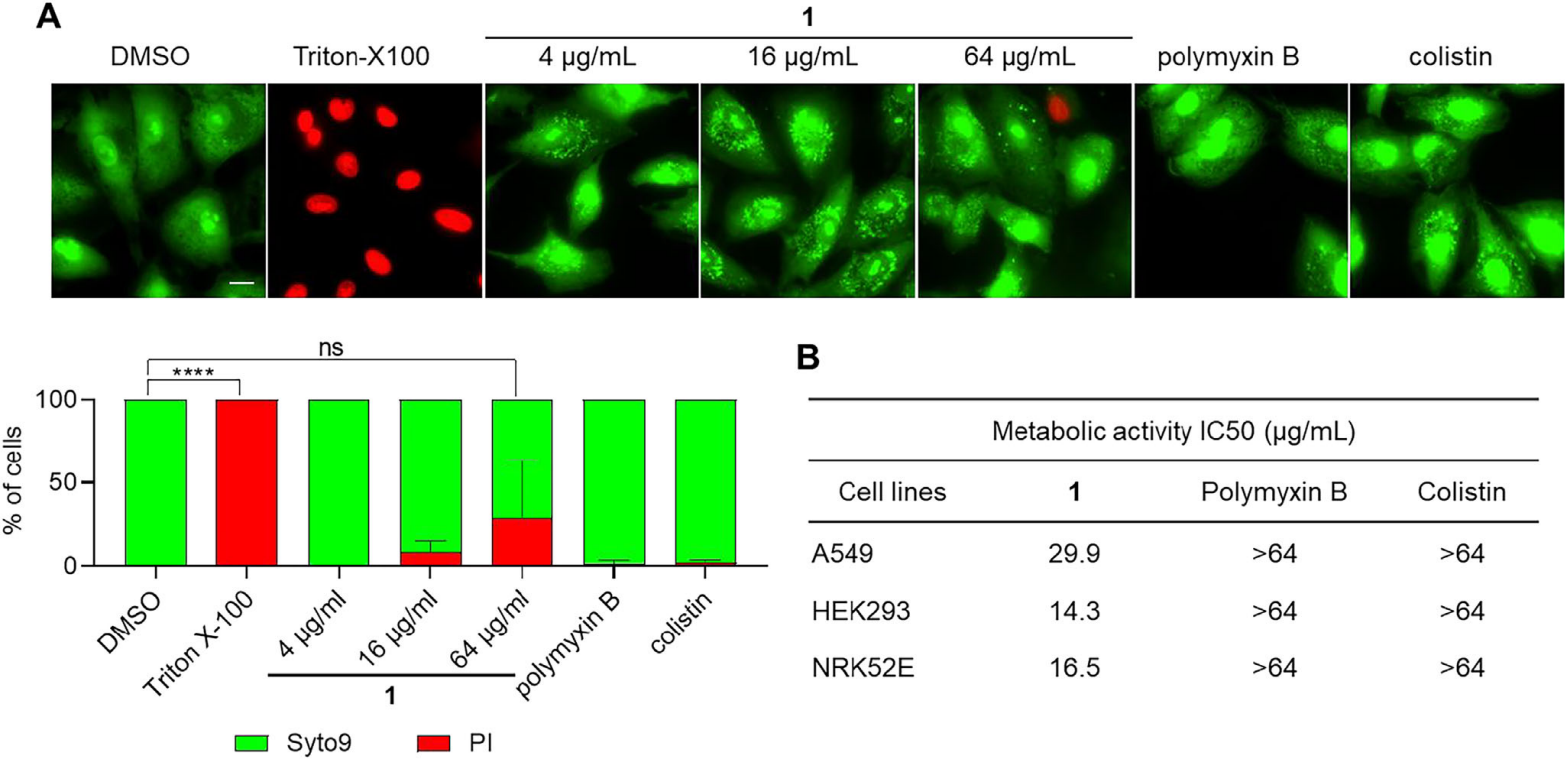
Limited permeabilization of eukaryotic membranes by marinocyclin A (**1**). (A) Top: Syto9/propidium iodide imaging of eukaryotic cytoplasmic membrane integrity in A549 cells after 30 min of marinocyclin exposure. Polymyxin B and colistin are used at 64 µg/mL. Scale bar, 20 µm. Triton X‐100 (0.1%) and DMSO (1%) served as controls. Bottom: Bar chart showing statistics of the experiment depicted in a). Quantification from > 100 cells per condition (*n* = 3, ± SD, two‐way ANOVA, ns, *p* > 0.06, ****p < 0.0001). (B) Cytotoxicity assessed by resazurin reduction; IC_50_ values indicate 50% metabolic inhibition.

### 2.9 Marinocyclin A (**1**) Interacts With Anionic Phospholipids, Lipopolysaccharides, and Peptidoglycan Precursors

Given the pronounced effects of **1** on membrane integrity, we next examined whether the compound binds to membrane lipids. To this end, we continuously monitored *S. aureus* growth for 24 h in the absence or presence of **1**, and after preincubating **1** with various membrane lipids. Lipids that stably bound **1** prevented its interaction with the bacterial surface, thereby enabling growth of cultures at concentrations above the MIC (Figure 6a). Screening phospholipids with varied head groups, fatty‐acid chain lengths, and degrees of saturation revealed a clear trend: **1** exhibited a strong binding preference for phospholipids containing negatively charged head groups and unsaturated fatty‐acid chains, whereas phospholipids with positively charged head groups or saturated chains showed no antagonism (Figure 6a). For example, the anionic lipids phosphatidylglycerol PG18:1 and cardiolipin (i.e., diphosphatidylglycerol, CL18:1) bound strongly, while their saturated 18:0 counterparts, as well as positively charged lysyl phosphatidylglycerol, did not antagonize. The negatively charged isoprenoid C_15_‐PP also antagonized **1**, suggesting that undecaprenyl pyrophosphate (C_55_‐PP), a product of peptidoglycan transglycosylation, may also serve as a binding partner for **1**.

**FIGURE 6:**
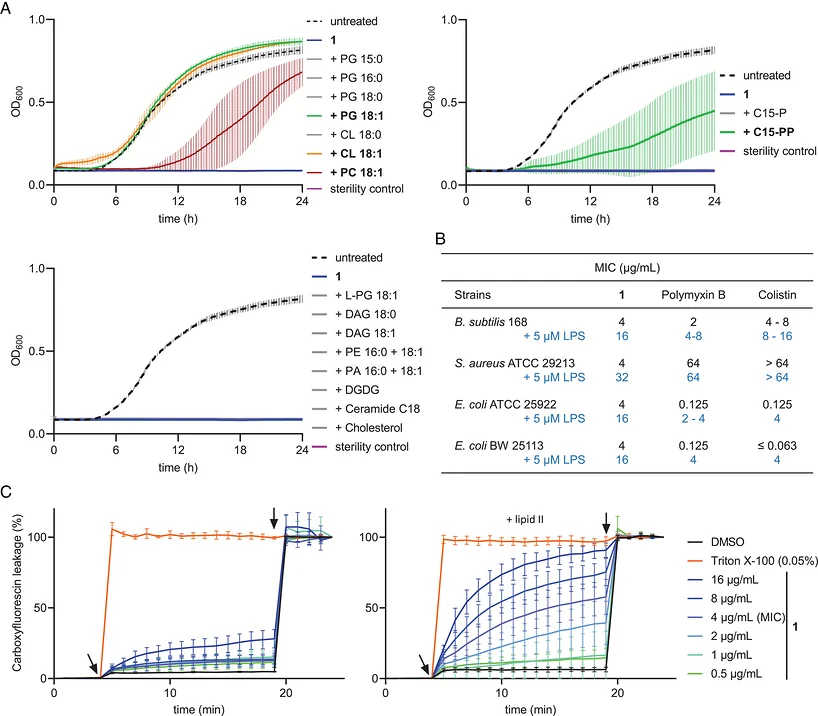
Marinocyclin A (**1**) binds anionic membrane lipids and LPS. (a) Antagonization of **1** (4× MIC, 16 µg/mL) by pre‐incubation with purified lipids (4‐fold molar excess, 15 min). Interaction of **1** with purified lipids prevents its ability to inhibit bacterial growth (recorded at OD_600_). Purified lipids successfully competing with cells for marinocyclin A‐binding are indicated in bold (*n* = 3, mean ± SD). (b) LPS‐dependent antagonization of **1** reflected by an MIC increase after preincubation with purified *E. coli* LPS (5 µM; *n* = 3). (c) Permeabilization of large unilamellar vesicles (GUVs) resembling the phospholipid head group composition in *B. subtilis* membranes (±0.5% added lipid II) by **1**, monitored via fluorescence readout of leaking carboxyfluorescein (*n* = 3, mean ± SD). First arrow, compound addition; second arrow, addition of Triton X‐100 as internal positive control. DMSO (4%) was used as the negative control.

While these interactions account for the disruptive effects on the cytoplasmic membrane, they do not explain the initial encounter of **1** with the surface of gram‐negative bacteria, which is composed of lipopolysaccharides (LPS) and not phospholipids. Indeed, **1** also bound purified *E. coli* LPS, and this competition resulted in a 4‐ to 8‐fold reduction in antibacterial activity (Figure 6b), which provides a rationale for the outer membrane compromising function of marinocyclin A.

The interaction with multiple lipid species suggested an antibacterial mode of action based on general disturbance of membrane topology rather than inhibition of a single protein target. Therefore, we next assessed if **1** can also disrupt the integrity of artificial liposomes. Large unilamellar vesicles (1 µm diameter) were prepared, the head‐group composition of which resembled that of the *B. subtilis* cytoplasmic membrane, and which consisted of phosphatidylglycerol, cardiolipin, and phosphatidylethanolamine. We also tested the influence of adding a low proportion (0.5%) of the peptidoglycan precursor lipid II (undecaprenyl diphospho‐*N*‐acetylmuramoyl‐pentapeptide‐*N*‐acetyl glucosamine). Within the vesicle lumen, the fluorophore carboxyfluorescein was contained at a self‐quenching concentration, and an increase in fluorescence signaled release of the fluorophore by vesicle perforation. Notable carboxy‐fluorescein leakage occurred in these protein‐free, non‐energized vesicles lacking osmotic pressure at 16 µg/mL marinocyclin A. Addition of lipid II improved the pore‐forming capacity of **1** by 10‐fold (Figure 6c), indicating that lipid II, which also contains the C_55_‐PP moiety, is another important interaction partner. In contrast, the permeabilizing capacity of polymyxin B and colistin was not markedly enhanced by lipid II (Figure S71).

Bacterial cytoplasmic membranes are abundant in the anionic phospholipids PG and CL, which strongly interact with cationic **1**, whereas zwitterionic phosphatidylcholine, a major component of eukaryotic membranes, showed weaker binding. In addition, anionic lipid II is characteristic of bacterial membranes. The lipid preference of **1** could explain the ∼10‐fold higher concentration required to permeabilize eukaryotic cytoplasmic membranes. This emerging selectivity represents an important parameter for optimizing the therapeutic window. **1** also displayed a substantial preference for unsaturated over saturated fatty‐acid chains. Thus, **1** may accumulate in regions of increased fluidity (fluid microdomains, RIFs) [44] in bacterial cells, where the formation of rigid lipid–peptide clusters could trigger rupture.

The speed at which bacterial killing occurs supports a biophysical process of membrane disruption. Already 5 min after marinocyclin exposure, the earliest time point analyzed in the NPN assay, the outer membrane was strongly permeabilized. The same time span was sufficient to collapse the electrochemical gradients across the cytoplasmic membrane. The Bau‐reporter showed rapid, detergent‐like lysis kinetics, and the membrane‐impermeant dye propidium iodide had already entered the cytoplasm after 15 min, again the earliest time point analyzed. In a time‐kill study, 15 min were sufficient to eradicate an *S. aureus* culture to the detection limit (≥ 4 log reduction in colony‐forming units, CFU, Figure S72), indicating rapid bactericidal activity. Inhibition of a metabolic reaction generally takes longer to take effect because the critical metabolite must first be depleted. Also, inhibition of transport processes essential for cell envelope lipid homeostasis has been associated with slower killing or less severe phenotypes in previous studies. For instance, zosurabalpin, a tethered macrocyclic peptide targeting the multimeric ABC transporter LptB_2_FGC in *A. baumannii* shuttling LPS from the cytoplasmic to the outer membrane, required 20 h at 4× MIC to reach 3 log CFU reduction, while colistin tested side‐by‐side achieved ≥ 4 log reduction within 2 h [45]. The Mla (maintenance of lipid asymmetry) pathway, consisting of the ABC transporter MlaFEBD and associated proteins MlaA and MlaC, performs retrograde lipid trafficking to remove ectopic phospholipids from the outer leaflet of the outer membrane and return them to the cytoplasmic membrane. Deleting components of the Mla system in various Gram‐negative bacteria resulted in enhanced sensitivity to SDS‐EDTA and antibiotics, increased shedding of outer membrane vesicles, and modulation of virulence, but no severe growth defects under standard laboratory conditions [46, 47].

In interacting with a diverse set of binding partners, including phospholipids, peptidoglycan precursors, and LPS, **1** exhibits polypharmacology, which likely slows the development of resistance. In agreement, we did not manage to obtain resistant colonies of *E. coli* after 13 consecutive days of passaging in serial dilutions of **1**. Next, we assessed the impact of known resistance determinants of structurally or mechanistically related antibacterial agents. Polymyxin resistance has become a global concern. The phosphoethanolamine (pEtN) transferase encoded by plasmid‐borne *mcr‐1* attaches pEtN to lipid A [48]. Mutations in the sensor kinase PmrB lead to constitutive expression of enzymes that decorate LPS with pEtN or 4‐amino‐4‐deoxy‐L‐arabinose (Ara4N) [38]. Both modifications reduce the net negative charge of the lipid A region of LPS, which diminished the activity of colistin, but **1** was not affected in *E. coli* (Figure S69, Table 1, and Table S13). Cationic antimicrobial peptides are often inhibited by modifications reducing the negative charge of phospholipids, teichoic or lipoteichoic acids [49], but **1** was insensitive to such modulations in *S. aureus* (Table S14). We also tested the daptomycin resistance factor MprF, a lysyl‐PG synthase/flippase in *S. aureus*, given that daptomycin likewise binds to PG and LII, but **1** was insensitive to MprF activity (Table S14). Finally, analysis of isogenic *E. coli* mutants (Table S14) demonstrated that **1** is not a substrate of AcrAB–TolC, the major efflux pump of *E. coli* [50].

Colistin (polymyxin E) remains a last‐resort treatment for severe infections caused by multidrug‐resistant gram‐negative pathogens. Marinocyclins represent a new broad‐spectrum antibacterial scaffold, unaffected by key resistance determinants of colistin and other antimicrobial peptides, and capable of efficiently targeting both the outer and inner membranes of diverse bacterial pathogens. The molecular insights provided here form the foundation for enhancing prokaryotic selectivity toward optimizing these antibiotics for therapeutic use.

## 3 Conclusion

Novel antibiotic classes with broad activity against Gram‐negative pathogens and without cross‐resistance to marketed drugs are exceedingly rare. Marinocyclins from the coral‐associated bacterium *A. megaterium* represent such a class, defined by a new chemical scaffold. The main congener marinocyclin A (**1**) displayed an exceptionally broad spectrum of activity, potently inhibiting multidrug‐resistant Gram‐negative and Gram‐positive pathogens that are exceedingly difficult to treat with current antibiotics. **1** evaded efflux and surface charge‐based resistance mechanisms and showed limited toxicity toward eukaryotic membranes. The marinocyclins highlight the potential of the underexplored marine genus *Aquimarina* as a source of novel antibiotic scaffolds. Future efforts to enhance the selectivity of marinocyclin derivatives for prokaryotic over eukaryotic membranes could yield promising preclinical candidates to address the urgent need for new therapies against drug‐resistant bacterial infections. More broadly, these findings support the potential of understudied microbes and habitats to reinvigorate the stagnated antibiotic pipeline with novel chemical diversity.

## Author Contributions

**Clara Chepkirui**: investigation, writing – original draft, methodology, validation, visualization, writing – review and editing, formal analysis, supervision, data curation, conceptualization. **Sandra Silva**: conceptualization, investigation, writing – original draft, methodology, validation, formal analysis, data curation, resources. **Jan Straetener**: methodology, validation, visualization, writing – original draft, formal analysis, data curation. **Chandrashekhar Padhi**: investigation, methodology, validation, writing – review and editing, formal analysis. **Tina Keller‐Costa**: supervision, conceptualization, formal analysis. **Tess Lamer**: investigation, formal analysis, validation, writing – review and editing, methodology, conceptualization. **Laura Reinke**: investigation, methodology, validation, visualization, formal analysis. **Heike Brötz‐Oesterhelt**: conceptualization, supervision, data curation, project administration, formal analysis, visualization, methodology, writing – original draft, writing – review and editing, funding acquisition. **Rodrigo Costa**: conceptualization, supervision, data curation, project administration, formal analysis, visualization, writing – review and editing, funding acquisition, writing – original draft, methodology, investigation, validation, resources. **Jörn Piel**: conceptualization, investigation, funding acquisition, writing – original draft, writing – review and editing, visualization, validation, methodology, formal analysis, project administration, resources, supervision, data curation.

## Conflicts of Interest

The authors declare no conflicts of interest.

## Supporting information




**Supporting File**: anie74512‐sup‐0001‐SuppMat.pdf.

## Data Availability

The data that support the findings of this study are available in the Supporting Information of this article.
