# Supplementary material for: Broad‐Spectrum, Cell Envelope‐Active Marinocyclin Antibiotics From a Coral‐Derived Bacterium Are Effective Against Colistin‐Resistant Bacteria
Source: Angew Chem Int Ed Engl. 2026 Sep 3;65(43):e7705008. doi: 10.1002/anie.7705008 (PMC13676389; doi:10.1002/anie.7705008)
Supplement: Supplementary file 1 [file ANIE-65-e7705008-s001.pdf]

Broad-spectrum, cell envelope-active marinocyclin antibiotics from a coral-derived bacterium are effective against colistin-resistant bacteria

Clara Chepkirui,[1,2] Sandra Silva,[3,4] Jan Straetener,[5,6] Chandrashekhar Padhi,[1,7] Tina Keller-Costa,[3,4] Tess Lamer,[1] Laura Reinke,[5,6,8] Heike Brötz-Oesterhelt[5,6,8*] Rodrigo Costa,[3,4*] and Jörn Piel[1*]

[1]Institute of Microbiology, Eidgenössische Technische Hochschule (ETH) Zürich, Zurich, Switzerland

[2]Current address: Institute of Food Chemistry, Leibniz University Hannover, Hannover, Germany

[3]iBB - Institute for Bioengineering and Biosciences and i4HB - Institute for Health and Bioeconomy, Instituto Superior Técnico, Lisbon, Portugal

[4]Department of Bioengineering, Instituto Superior Técnico, Lisbon, Portugal

[5]Interfaculty Institute of Microbiology and Infection Medicine, Microbial Bioactive Compounds, University of Tübingen, Auf der Morgenstelle 28, 72076 Tübingen, Germany.

[6]Cluster of Excellence "Controlling Microbes to Fight Infection"

[7]Current address: Department of Chemistry, University of Illinois, Urbana, IL, USA.

[8]German Center for Infection Research (DZIF)

Table of contents

General materials and methods 6
Identification of *Aquimarina megaterium* EL43........ 6
Bioinformatic analysis........ 6
Optimization of growth conditions for *A. megaterium* EL43 6
Large-scale cultivation of *A. megaterium* EL43........ 6
Tandem MS (MS^2) molecular network analysis 7
Attempts to generate linearized marinocyclin A (1)........ 7
Acid hydrolysis and Marfey's analysis of marinocyclin A (1)........ 8
Advanced Marfey's analysis........ 8
Synthesis of (2*S*,3*S*) and (2*R*,3*S*)-2,4-diamino-3-hydroxybutanoic acid........ 8
Molecular Biology 9
Enzyme Expression and Purification........ 9
MarE β-Hydroxlation Enzyme Assay........ 9
MarF β-Hydroxlation Enzyme Assay 10
Amino Acid Fmoc Derivatization and LC-MS 10
Determination of minimum inhibitory concentrations (MICs)........ 10
Bioreporter assay for target pathway elucidation 11
1-*N*-Phenylnaphthylamine (NPN) uptake assay........ 11
Membrane potential assay 11
Cytoplasmic membrane permeabilization 11
Lysis assay (Bau reporter)........ 12
Peptidoglycan integrity assessment 12
Lipid antagonization assay 12
Artificial membrane vesicle permeabilization assay........ 12
Cytotoxicity assay........ 13
Time-kill curve assay 13
Chemical data........ 13
DNA sequences........ 14
Supplementary tables........ 16
Supplementary Figures 40
References 87

List of Tables

Table S1. BGC types on the genome of *A. megaterium* EL43 predicted by antiSMASH. 16
Table S2. Proteins of the marinocyclin (*mar*) biosynthetic gene cluster. 17
Table S3. Comparison of antiSMASH 8.0.4- and PRISM 4-predicted adenylation (A) domain substrate specificities of the mar NRPS and amino acid residues present in marinocyclins. 18
Table S4. NMR data for marinocyclin A (**1**) in methanol-d_4 and DMSO-d_6 (^{1}H 600 MHz, ^{13}C150 MHz) 19
Table S5. NMR data for marinocyclin B (**2**) in methanol-d_4 (^{1}H 600 MHz, ^{13}C150 MHz)............................ 22
Table S6. NMR data for marinocyclin C (**3**) in methanol-d_4 (^{1}H 600 MHz, ^{13}C150 MHz) 24
Table S7. NMR data for marinocyclin D (**4**) in methanol-d_4 (^{1}H 600 MHz, ^{13}C 150 MHz) 26
Table S8. NMR data for marinocyclin E (**5**) in methanol-d_4 (^{1}H 600 MHz, ^{13}C 150 MHz)........................... 29
Table S9. NMR data for marinocyclin F (**6**) in methanol-d_4 (^{1}H 600 MHz, ^{13}C 150 MHz)........................... 31
Table S10. MS^2 molecular network analysis of *A. megaterium* crude extract 33
Table S11. Bacterial test strains used in this study 34
Table S12. Minimal Inhibitory Concentrations (MIC) of marinocyclin A (**1**), B (**2**), and D (**4**) for a panel of bacteria by Method A. 37
Table 13. Minimal Inhibitory Concentrations (MIC) of marinocyclins A (**1**), B (**2**), and D (**4**) for a panel of polymyxin-resistant *E. coli* clinical isolates with mutations in *pmrB* and *pmrC* 38
Table S14. Minimal Inhibitory Concentrations (MIC) for efflux pump mutants in *E. coli* and surface charge mutants in *S. aureus*. 38

List of figures

Figure S1. Total ion chromatogram (TIC) and MS^2 molecular network analysis of the pellet-derived extract of *A. megaterium* EL43 cultivated in marine broth. 40

Figure S2. HPLC-HESI-HRMS data of marinocyclin A (**1**). 41

Figure S3. HESI-HRMS MS^2data of marinocyclin A (**1**) 42

Figure S4. MS^2 fragmentation of marinocyclin A (**1**). 42

Figure S5. ^{1}H NMR spectrum of marinocyclin A (**1**) in methanol-d_4 (^{1}H 600 MHz, ^{13}C 150 MHz)........ 43

Figure S6. COSY spectrum of marinocyclin A (**1**) in methanol-d_4 (^{1}H 600 MHz). 43

Figure S7. HSQC spectrum of marinocyclin A (**1**) in methanol-d_4 (^{1}H 600 MHz, ^{13}C 150 MHz) 44

Figure S8. HMBC spectrum of marinocyclin A (**1**) in methanol-d_4 (^{1}H 600 MHz, ^{13}C 150 MHz). 44

Figure S9. Zoomed HMBC spectrum of marinocyclin A (1) in methanol-d_4 (1H 600 MHz, 13C 150 MHz). 45

Figure S10. ^{1}H NMR spectrum of marinocyclin A (**1**) in DMSO-d_6 (^{1}H 600 MHz, ^{13}C 150 MHz). 45

Figure S11. COSY spectrum of marinocyclin A (**1**) in DMSO-d_6 (^{1}H 600 MHz). 46

Figure S12. NOESY spectrum of marinocyclin A (**1**) in DMSO-d_6 (^{1}H 600 MHz). 46

Figure S13. Zoomed NOESY spectrum of marinocyclin A (1) in DMSO-d_6 (1H 600 MHz). 47

Figure S14. HSQC spectrum of marinocyclin A (**1**) in DMSO-d_6 (^{1}H 600 MHz, ^{13}C 150 MHz). 47

Figure S15. Structure elucidation of marinocyclin A (**1**). Top: NOESY correlations of **1**, bottom: COSY and HMBC correlations of **1**. 48

Figure S16. HPLC-HESI-HRMS data of marinocyclin A (**1**), product of **1** after chymotrypsin digest and hydrolysis of **1** with NaOH.. 49

Figure S17. HPLC-HESI-HRMS data of marinocyclin B (**2**). 50

Figure S18. HESI-$HRMS^2$ data of marinocyclin B (**2**)........ 51

Figure S19. MS^2 fragmentation of marinocyclin B (**2**). 51

Figure S20. ^{1}H NMR spectrum of marinocyclin B (**2**) in methanol-d_4 (^{1}H 600 MHz). 52

Figure S21. COSY spectrum of marinocyclin B (**2**) in methanol-d_4 (^{1}H 600 MHz). 52

Figure S22. HSQC spectrum of marinocyclin B (**2**) in methanol-d_4 (^{1}H 600 MHz, ^{13}C 150 MHz). 53

Figure S23. HMBC spectrum of marinocyclin B (**2**) in methanol-d_4 (^{1}H 600 MHz, ^{13}C 150 MHz). 53

Figure S24. HPLC-HESI-HRMS data of marinocyclin C (**3**). 54

Figure S25. HESI-$HRMS^2$ data of marinocyclin C (**3**). 55

Figure S26. MS^2 fragmentation of marinocyclin C (**3**). 55

Figure S27. ^{1}H NMR spectrum of marinocyclin C (**3**) in methanol-d_4 (^{1}H 600 MHz). 56

Figure S28. COSY spectrum of marinocyclin C (**3**) in methanol-d_4 (^{1}H 600 MHz). 56

Figure S29. HSQC spectrum of marinocyclin C (**3**) in methanol-d_4 (^{1}H 600 MHz, ^{13}C 150 MHz). 57

Figure S30. HMBC spectrum of marinocyclin C (**3**) in methanol-d_4 (^{1}H 600 MHz, ^{13}C 150 MHz). 57

Figure S31. HPLC-HESI-HRMS data of marinocyclin D (**4**). 58

Figure S32. HESI-$HRMS^2$ data of marinocyclin D (**4**) 59

Figure S33. MS^2 fragmentation of marinocyclin D (**4**). 59

Figure S34. ^{1}H spectrum of marinocyclin D (**4**) in methanol-d_4 (^{1}H 600 MHz)........ 60

Figure S35. COSY spectrum of marinocyclin D (**4**) in methanol-d_4 (^{1}H 600 MHz). 60

Figure S36. HSQC spectrum of marinocyclin D (**4**) in methanol-d_4 (^{1}H 600 MHz, ^{13}C 150 MHz). 61

Figure S37. HMBC spectrum of marinocyclin D (**4**) in methanol-d$_4$ (^{1}H 600 MHz, ^{13}C 150 MHz). 61
Figure S38. ^{1}H NMR spectrum of marinocyclin D (**4**) in DMSO-d$_6$ (^{1}H 600 MHz). 62
Figure S39. COSY spectrum of marinocyclin D (**4**) in DMSO-d$_6$ (^{1}H 600 MHz). 62
Figure S40. HSQC spectrum of marinocyclin D (**4**) in DMSO-d$_6$ (^{1}H 600 MHz, ^{13}C 150 MHz). 63
Figure S41. HMBC spectrum of marinocyclin D (**4**) in DMSO-d$_6$ (^{1}H 600 MHz, ^{13}C 150 MHz). 63
Figure S42. NOESY spectrum of marinocyclin D (**4**) in DMSO-d$_6$ (^{1}H 600 MHz). 64
Figure S43. HPLC-HESI-HRMS data of marinocyclin E (**5**). 65
Figure S44. HESI-HRMS^2 data of marinocyclin E (**5**) 66
Figure S45. MS^2 fragmentation of marinocyclin E (**5**). 66
Figure S46. ^{1}H NMR spectrum of marinocyclin E (**5**) in methanol-d$_4$ (^{1}H 600 MHz). 67
Figure S47. COSY spectrum of marinocyclin E (**5**) in methanol-d$_4$ (^{1}H 600 MHz). 67
Figure S48. HSQC spectrum of marinocyclin E (**5**) in methanol-d$_4$ (^{1}H 600 MHz, ^{13}C 150 MHz). 68
Figure S49. HMBC spectrum of marinocyclin E (**5**) in methanol-d$_4$ (^{1}H 600 MHz, ^{13}C 150 MHz). 68
Figure S50. HPLC-HESI-HRMS data of marinocyclin F (**6**). 69
Figure S51. ^{1}H NMR spectrum of marinocyclin F (**6**) in methanol-d$_4$ (^{1}H 600 MHz). 70
Figure S52. COSY spectrum of marinocyclin F (**6**) in methanol-d$_4$ (^{1}H 600 MHz). 70
Figure S53. HSQC spectrum of marinocyclin F (**6**) in methanol-d$_4$ (^{1}H 600 MHz, ^{13}C 150 MHz). 71
Figure S54. HMBC spectrum of marinocyclin F (**6**) in methanol-d$_4$ (^{1}H 600 MHz, ^{13}C 150 MHz). 71
Figure S55. HPLC-HESI-HRMS data of marinocyclin G (**7**). 72
Figure S56. ^{1}H NMR spectrum of marinocyclin G (**7**) in methanol-d$_4$ (^{1}H 600 MHz). 73
Figure S57. HSQC spectrum of marinocyclin G (**7**) in methanol-d$_4$ (^{1}H 600 MHz, ^{13}C 150 MHz). 73
Figure S58. Overlayed ^{1}H NMR spectra of marinocyclin F (**6**) and marinocyclin G (**7**) in methanol-d$_4$ (^{1}H 600 MHz). 74
Figure S59. Proposed structure of marinocyclin G (**7**). 74
Figure S60. ^{1}H NMR spectrum of (2*S*,3*S*) and (2*R*,3*S*)-2,4-diamino-3-hydroxybutanoic acid diastereomeric mixture in D$_2$O (^{1}H 600 MHz). 75
Figure S61. Marfey's analysis of marinocyclin A (**1**)**.** Comparison of Extracted Ion Chromatograms (EICs) of FDVA-derivatized marinocyclin A (**1**) hydrolysate to the EICs of the D- and L-amino acid standards. ... 76
Figure S62. Electronic circular dichroism (ECD) spectra of marinocyclins A (**1**), B (**2**), and D (**4**) in ethanol. 77
Figure S63. Purification of putative marinocyclin amino acid hydroxylases 77
Figure S64. β-Hydroxylation assay of purified Fe^{II}/α-ketoglutarate-dependent enzyme MarE with free amino acid substrates. 78
Figure S65. β-Hydroxylation assay of purified non-heme diiron monooxygenase MarE with free amino acid substrates. 79
Figure S66. Minimal inhibitory concentration (MIC) determination of marinocyclins against *A. baumannii* DSM 30007, *K. pneumoniae* DSM 25721, *S. enterica* STM SB300, and *E. coli* DSM 22664.. 80
Figure S67. MIC determination of marinocyclins against *P. aeruginosa* DSM 24599, *P. aeruginosa* DSM 1117, *P. agglomerans* DSM 3493, and *P. stutzeri* LMG 5383. 81
Figure S68. MIC determination of marinocyclins against *E. coli* DSM 1003, *E. coli* BW25112, *B. subtilis* 168 DSM 10, and *S. epidermidis* ATCC 12228. 82
Figure S69. MIC determination of marinocyclins against *E. coli* clinical isolates. 83

Figure S70. Marinocyclin A-treated *B. subtilis* senses cell envelope stress**.** Stress-response profiling of marinocyclin A (**1**) in isogenic *B. subtilis* 1S34 reporter strains. 84

Figure S71. Permeabilization of large unilamellar vesicles (GUVs) resembling the phospholipid head group composition in *B. subtilis* membranes (+/- added 0.5% lipid **II**) by polymyxin B, colistin, and nisin. 85

Figure S72. Time-kill curve of *S. aureus* ATCC 29213 treated with marinocyclin A 86

General materials and methods

The mass spectrometric (MS) data were acquired on a Thermo Scientific Q Exactive or LTQ Orbitrap XL mass spectrometer coupled to a Dionex Ultimate 3000 high-performance liquid chromatography (HPLC) system operated by Xcalibur 4.1 and Chromeleon Xpress 7.2 (Thermo Scientific) respectively (column: Kinetex 2.6 µm XB-C18 100 Å 150x4.6 nm; solvent A (H_2O + 0.1% formic acid); solvent B (MeCN + 0.1% formic acid); gradient, 5% B for 0.5 min increasing to 100% B in 18 min and then maintaining 100% B for 1 min; flow rate 0.7 mL/min; 27 °C UV-Vis detection 200–600 nm combined with *m/z* detection (scan range 100–2500 *m/z*, capillary voltage 4500 V, dry temperature 200 °C). NMR spectra were recorded on a Bruker Avance III spectrometer equipped with a cryoprobe at 500 MHz and 600 MHz for ^{1}H NMR and 125 MHz and 150 MHz for ^{13}C NMR at 298 K, operated by TopSpin 3.5/4.1 (Bruker). Chemical shifts were referenced to the corresponding solvent peaks. Data were analyzed using Xcalibur 4.1, TopSpin 4.1, and MestReNova (Mestrelab Research). Agilent Infinity 1260 HPLCs operated by Open Lab CDS 2.2 (Agilent) were used for isolation. Optical rotation data were acquired at room temperature on a Jasko P-200 series polarimeter.

Identification of *Aquimarina megaterium* EL43

A. megaterium EL43 was isolated from a specimen of the gorgonian octocoral *Eunicella labiata* sampled in the northeast Atlantic Ocean, near the Portuguese coast.[1] The corresponding genome assembly is accessible through the accession number GCA_943422735. The strain was identified as *A. megaterium* based on > 99% Average Nucleotide Identities (ANI) and > 97% digital DNA-DNA hybridization probabilities with *A. megaterium* XH134(T), the type strain of the species. [2]

Bioinformatic analysis

Biosynthetic gene clusters (BGCs) present in the *A. megaterium* EL43 genome were annotated using antiSMASH 8.0.4. [3] Prediction of adenylation domain specificities for amino acids (Table S3) and condensation domain functional subtypes was achieved by combining antiSMASH and PRISM 4 outputs.[4]

Optimization of growth conditions for *A. megaterium* EL43

Bacteria were cultivated at 24 °C in 500 mL Erlenmeyer flasks containing 200 mL of one of the following liquid media: self-made marine broth (MB) (5 g bacteriological peptone, 1 g yeast extract, 33 g instant ocean (Aquarium Systems), 1 L water); PH-103[12] medium (20 g tryptone, 5 g yeast extract, 4 g D-glucose, 4 g maltose, 4 g CaCO3, 27 g instant ocean, 1 L water); chitin medium (3 g of pre washed and dried chitin, 3 g casamino acids (Bacto), 27 g instant ocean, 1 L water) and commercial marine broth (DIFCO 2216). Following cultivation for 4 days, the pellet and supernatant were separated by centrifugation. The supernatant was extracted with an equal volume of ethyl acetate. The organic phase was collected and evaporated to dryness under vacuum. The pellet was extracted with 200 mL of acetone in an ultrasonic bath for 30 minutes, and the solvent was evaporated under vacuum. 100 mL of water was added to the remaining water phase and extracted similarly as the supernatant. The resulting extracts were analysed by HPLC coupled to heated electrospray ionization-high resolution MS (HPLC-HESI-HRMS). Cultures grown in marine broth (DIFCO 2216) showed the most diverse total ion chromatogram. The target masses were identified in the pellet extract.

Large-scale cultivation of *A. megaterium* EL43

20 L of liquid cultures of *A. megaterium* EL43 were grown in MB (DIFCO 2216). 2 L Erlenmeyer flasks containing 1 L of the medium (total 20 L) were inoculated with 1% of a pre-inoculum culture and incubated for four days at 24 ºC and 150 rpm. The pellet and supernatant were separated by centrifugation. The pellet

was extracted three times with 700 mL of acetone in an ultrasonic bath for 30 minutes, filtered, and the solvent was evaporated under vacuum to dryness.

Natural product isolation

Crude extracts from the pellet were dissolved in methanol and fractionated using an Agilent 1260 Infinity preparative reverse-phase HPLC system equipped with a Phenomenex column (Luna C18, 5 µm, 250 x 21.2 mm) and coupled to an Agilent G1315D diode array detector. Deionized water with 0.05% trifluoroacetic acid (TFA) (solvent A) and acetonitrile with 0.05% TFA (solvent B) were used as mobile phases. A flow rate of 12 mL/min was applied. An elution gradient of 5% solvent B for 3 minutes followed by a gradient increase from 5–100% solvent B in 50 minutes, and final isocratic conditions at 100% solvent B for 10 minutes was applied. UV detection was carried out at 210, 254, and 280 nm. LC-MS analysis of the fractions collected allowed the identification of the fractions containing the compounds of interest (fractions F6-F7). A second round of purification was performed on a Phenomenex Kinetex C18 column (5 µm, 21.2 x 250 mm), with an elution gradient of 30-100% for solvent B for 30 minutes, followed by isocratic conditions at 100% solvent B for 10 min. Fractions containing the target compounds were identified by HPLC-HESI-HRMS. Further purification of the identified fractions was carried out by semi-preparative reverse-phase HPLC, equipped with a Luna C18 column (5 µm, 250 x 10 mm). Elution gradient: solvent B at 30% for 3 minutes, followed by a gradual increase of solvent B concentration until 70% in 30 minutes, and a second increase of solvent B up to 100% in five minutes.

Tandem MS (MS^2) molecular network analysis

Raw HPLC-HESI-HRMS spectra were converted to the open-access mzXML format with the ProteoWizard tool msConvert GUI v3.0. in centroid mode before being uploaded to the GNPS[5] website with the WinSCP v5.19.1 tool. Classical molecular network analysis (v28.2) was carried out in GNPS as follows: All MS^2 fragment ions within +/-17 Da of the precursor *m/z* were eliminated, and only the top 6 fragment ions in the +/-50 Da window were selected. Tolerances for precursor and fragment ions were set to 0.01 Da. Edges having a cosine score below 0.7 and fewer than six matching peaks were eliminated once the network was created. Furthermore, any edges between two nodes had to be within their top 10 most similar nodes; otherwise, they were disregarded. The lowest-scoring edges were eliminated from molecular families until the molecular family size was below 100. Cytoscape v3.8.2 was used for network visualization.

Attempts to generate linearized marinocyclin A (1)

Chymotrypsin digest: 0.3 mg of marinocyclin A (**1**) was dissolved in 1mL of 100 mM Tris-HCl, 10 mM CaCl2 (pH 8.0). 50 µL of lyophilized Chymotrypsin dissolved in 1mM HCl (1 µg/µL) was added, and the reaction mixture was incubated at 25 °C for 18 hours. An aliquot (10 µL) of the reaction mixture was analyzed using HPLC-HESI-HRMS (Bruker Maxis Impact). A Phenomenex Kinetex® 2.6 µm XB-C18 100 Å (150 × 4.6 mm, 1 mL/min) column, heated to 30°C, was used with a gradient elution using H_2O (mobile phase A) and MeCN (mobile phase B) containing formic acid (0.1%). A gradient elution from 5% to 100% B in 20 minutes was applied.

Hydrolysis with NaOH: 0.2 mg of marinocyclin A (**1**) was dissolved in 1 mL of 0.02 M, 1 M and 5 M and stirred at room temperature for 18 h. The reaction mixtures were evaporated to dryness and resuspended in 400 µL of methanol, and MS data were acquired.

Acid hydrolysis and Marfey's analysis of marinocyclin A (1)

Hydrolysis and Marfey's analysis were carried out according to Fujii et al.,[6] with slight modifications. Briefly, 1 mg of marinocyclin A (**1**) was dissolved in 200 µL of 6 M HCl and heated at 100 ºC with stirring for 18h. The reaction mixture was dried under reduced pressure and washed twice with 400 µL of water. The resulting dry mixture was dissolved in 200 µL of water. To 50 µL of the hydrolysate were added 20 µL of freshly prepared 1M $NaHCO_3$ and 100 µL of N_α-(2,4-dinitro-5-fluorophenyl)-L-valinamide (FVDA (4 mg/mL in acetone). The solution was incubated at 50 ºC for 1h. After cooling at room temperature, the reaction mixture was quenched with 20 µL 1 N HCl. The solution was diluted with 250 µL of acetonitrile for HPLC-HESI-HRMS analysis. Analysis was performed on a Thermo Scientific Q Exactive or LTQ Orbitrap XL mass spectrometer coupled to a Dionex Ultimate 3000 HPLC system operated by Xcalibur 4.1 and Chromeleon Xpress 7.2 (Thermo Scientific) software, respectively (column: Kinetex 2.6 µm XB-C18 100 Å 150x4.6 nm; solvent A (H_2O + 0.1% formic acid); solvent B (MeCN + 0.1% formic acid), flow; 0.8 mL/min. A gradient from 5% solvent B from 0-1min, followed by a gradient increase to 100% solvent B in 20 min and a linear gradient at 100% solvent B for 1 min. For Ile isomers, a gradient from 5-100% in 55 minutes was applied. 1 mg of amino acid standards was dissolved in water and derivatized with FDVA in the same manner described above for the acid hydrolysate for retention time comparison. Both L and D standards were used for Ser and Leu, and D-*allo*-Ile and DL-Ile for Ile standards.

Advanced Marfey's analysis

To determine the stereochemistry at C-2 and C-3 of the Asn-β-OH residue, an advanced Marfey's method was used. [7] Briefly, 50 µL of the hydrolysate of 1 was derivatized with L- and D-FDVA using the same method as above. An aliquot (10 µL) of each derivative was analyzed using HPLC-HRMS applying similar conditions described by Li et. [8] Briefly, a Phenomenex Kinetex® 2.6 µm XB-C18 100 Å (150 × 4.6 mm, 1 mL/min) column, heated to 40 °C was used with a gradient elution using H_2O (mobile phase A) and MeCN (mobile phase B) containing formic acid (0.1%). The gradient elution started at 27% B from 0–20 min, followed by a gradient shift from 27–45% B from 20 to 30 min, 45–27% B from 30 to 32 min, and 27% B from 32 to 40 min. The L-FDLA derivative eluted at a retention time of 7.14 mins, while the D-FDLA derivative eluted at a retention time of 6.81 mins. The retention times obtained were compared to those reported by Li et al. and Miller et al,[9] (L-FDLA-D-*threo*-β-OH-Asp (7.2 mins), L-FDLA-L-*threo*-β-OH-Asp (7.3 mins). D-FDLA-L-*threo*-β-OH-Asp (7.2 min), L-FDLA-L-*threo*-β-OH-Asp (7.3 min), D-FDLA-L-*erythro*-β-OH-Asp (10.0 min), and L-FDLA-L-*erythro*-β-OH-Asp (10.5 min).

Synthesis of (2*S*,3*S*) and (2*R*,3*S*)-2,4-diamino-3-hydroxybutanoic acid

(2*S*,*3S*)-2,4-Diamino-3-hydroxybutanoic was synthesized from hydroxyectoine as the starting material according to a published procedure. [10] Briefly, 50 g of hydroxyectoine was dissolved in 260 mL of water. 23 g of NaOH were added portion-wise, and the mixture was stirred until all NaOH was dissolved. The solution obtained was heated at 50 °C for 6 hours and then cooled to room temperature, and finally to 5 °C in an ice bath. HCl (6 N) was added carefully until pH = 4. The solution obtained was frozen with liquid nitrogen and then lyophilized. The white solid obtained was dissolved in 300 mL of HCl (6 N), and the mixture was heated at 110 °C for 3 hours. The solution obtained was diluted with 300 mL of water frozen with liquid nitrogen and lyophilized to give a of mixture of (2*S*,3*S*)-2,4-diamino-3-hydroxybutanoic acid and (*2R,3S*)-2,4-diamino-3-hydroxybutanoic acid (ratio 70:30) as a pale-yellow solid (80 g) (Figure 55). 1 mg of the product was derivatised with FVDA for Marfey's analysis in the same manner as for the amino acid standards described above.

Molecular Biology

DNA coding sequences for MarE with either an N-terminal hexahistidine tag or an N-terminal His_6-bdSUMO tag, and MarF with an N-terminal hexahistidine tag, were codon optimized for expression in *E. coli* using Twist Biosciences Codon Optimization Tool. Genes were synthesized by Twist Biosciences and inserted into pET_blank_Kan cloning vectors behind a T7-lacO-RBS sequence. *E. coli* BL21(DE3) TUNER cells were transformed with each plasmid, and cultures were stored as 20% glycerol stocks at −80 °C.

Enzyme Expression and Purification

Frozen glycerol stocks of *E. coli* BL21(DE3) TUNER cells transformed with the appropriate vector were inoculated into 50 mL of sterile LB media with kanamycin (50 µg/mL) as selective pressure. The cells were grown overnight at 37 °C with shaking at 200 rpm. The next day, 20 mL of the overnight culture was added to a 2 L baffled Erlenmeyer flask containing 500 mL of sterile Terrific Broth (TB) media with kanamycin added. The cultures were grown to an optical density of ~1.2 at 37 °C with shaking at 200 rpm, and then cooled on ice. Protein expression was induced by addition of isopropyl-β-D-1-thiogalactopyranoside (IPTG) to a final concentration of 0.05 mM for MarE-producing cultures or 0.2 mM IPTG for MarF-producing cultures. MarE-producing cultures also had fresh, sterile $FeSO_4$ added to a final concentration of 0.05 mM. The flasks were vigorously shaken for an additional 22 h at 20 °C. Cells were then harvested by centrifugation (5000 × *g*, 10 min, 4 °C) and the pellets were stored at −80 °C. MarE was mostly insoluble and obtained in low yield, so prior to freezing the cell pellets the periplasm was removed via osmotic shock using a literature method.[11] Briefly, the cell pellets were resuspended in 5 mL/g sucrose buffer (50 mM HEPES, 20% sucrose, 1 mM EDTA, pH 7.9). The cells were again pelleted by centrifugation at 7000 × *g* for 30 min at 4 °C. The supernatant was discarded and the pellet resuspended in 5 mL/g of 5 mM $MgSO_4$ and incubated on ice for 10 min. Cells were finally pelleted by centrifugation at 5000 × *g* for 20 min, the supernatant was discarded and the pellet was at –80 °C until lysis.

Frozen cell pellets were resuspended (40 mL buffer/500 mL culture media) evenly in ice cold lysis buffer (50 mM HEPES, 300 mM NaCl, and 5 mM imidazole, pH 8.0) and lysed by sonication while kept on ice. DNase I (Thermo Fisher Scientific, 1 U) was added and the lysate kept on ice for 30 min with occasional inversion. The cellular debris was removed by centrifugation (20,000 × *g*, 30 min, 4 °C), and then Ni-NTA resin (Qiagen) was added to the clarified supernatant, and the mixture was gently shaken for 1 h at 4 °C. The mixture was then loaded onto a fritted column, and the flow through collected at 4 °C. The resin was washed with 25 mL of lysis buffer containing 20 mM imidazole, and then the fusion protein was eluted with elution buffer (50 mM HEPES, 300 mM NaCl, 500 mM imidazole, pH 7). The buffer was exchanged to remove imidazole and proteins concentrated using an Amicon Ultra Centrifugal Filter, 10 kDa molecular weight cut-off (5000 × *g*, 4 °C). Purification samples were analysed using SDS-PAGE, and concentration was measured by absorbance at 280 nm using a Nanodrop spectrophotometer. Aliquots of the enzyme solutions were stored at −80 °C. His_6-MarF was obtained in a purified yield of 42 mg from 500 mL of media, while 4.5 mg of soluble His_6-MarE was obtained from 500 mL of culture media.

MarE β-Hydroxylation Enzyme Assay

β-Hydroxlation assays with His_6-MarE were conducted according to a modified literature procedure.[12] A 100 µL solution containing HEPES buffer (50 mM, pH 7), purified His_6-MarE (70 µM), 1 mM α-ketoglutarate, 1 mM amino acid (L-Dab, L-Phe, or L-Asn), 0.5 mM fresh $(NH_4)_2Fe(SO_4)_2$, and 0.5 mM fresh ascorbate was prepared and the reaction was allowed to proceed for 3 h at room temperature. Negative controls lacking each of the reaction components were also conducted at the same time. The reaction was quenched by addition of 100 µL of acetonitrile, and then centrifuged for 15 minutes at 15000 × *g*. The supernatant was removed for Fmoc derivatization.

MarF β-Hydroxlation Enzyme Assay

β-Hydroxlation assays with His_6-MarF were conducted according to a modified literature procedure.[13,14] A 50 µL solution containing HEPES buffer (50 mM, pH 7), purified His_6-MarF (100 µM), 1 mM amino acid (L-Dab, L-Phe, or L-Asn), and either A) fresh 10 µM methyl viologen with 1 mM NADH, or B) fresh 100 µM methyl viologen with 500 µM $Na_2S_2O_4$ was prepared and the reaction was allowed to proceed for 3 h at room temperature. Negative controls lacking each of the reaction components were also conducted at the same time. The reaction was quenched by addition of 50 µL of acetonitrile, and then centrifuged for 15 minutes at 15000 × *g*. The supernatant was removed for Fmoc derivatization.

Amino Acid Fmoc Derivatization and LC-MS

Derivatization of amino acid products from β-hydroxylation enzyme assays was conducted according to a literature procedure.[15] 100 µL of quenched hydroxylation assay mixture was mixed with 50 µL of 200 mM sodium borate (pH 8.5) and 20 µL of fresh 10 mM Fmoc-Cl in acetonitrile. After incubation at room temperature for 10 min, 20 µL of 100 mM hydroxylamine was added. The solution was centrifuged for 15 minutes at 15000 × *g* and then 10 µL of the mixture was analyzed using LC-MS and a Kinetex 2.6 µm XB-C18 100 Å LC Column (150 x 4.6 mm). The LC method used was as follows: 0–2 min 5% B, 2–19 min ramp from 5% to 80% B, 19–20 min ramp from 80% to 100% B, 20–21 min 100% B (solvent A, 0.1% formic acid in water; solvent B, 0.1% formic acid in acetonitrile). LC-MS data was analyzed using Thermo Scientific Freestyle, and chromatogram images were assembled using GraphPad Prism.

Determination of minimum inhibitory concentrations (MICs)

Initial MIC assays against a panel of bacteria were conducted with marinocyclin A-B (**1-2**) and D (**4**) at ETH Zurich (Table S12 and S13) in a serial dilution assay (Method A). The other isolated compounds were not tested because of the low amounts obtained. MIC tests were conducted in 96-well microtiter plates in nutrient broth (NB). The test organisms' cultures were adjusted an OD_{600} of 0.04. 100 µL of the test pathogen was aliquoted in all the rows except row A, which was aliquoted with 186.7 µL. 13.3 µL aliquots of compounds **1-2** and **4** at 1.5 mg/mL in MeOH and the reference drugs (kanamycin 50 µ/mL in water) were pipetted on the first row (A) of the plate and mixed by repeated pipetting, before transferring 100 µL to the second row. A 1:1 serial dilution was done in the subsequent rows, and 100 µL was discarded after the last row. Plates were incubated at 37 °C for 24 h. The lowest concentration of the drug preventing visible growth of the pathogen was taken as the MIC (Table S12 and S13, Figure S58-61).

Further MIC assays were conducted with marinocyclin A (**1**) at the University of Tübingen against a broader bacterial panel. Here, the broth microdilution method was used according to guidelines of the Clinical and Laboratory Standards Institute (CLSI) (Method B, Table 1 and S14).[16] Briefly, 10 mg/mL stock solutions of test compounds were prepared in DMSO and serial two-fold dilutions of each compound (64–0.06 µg/mL) were prepared in cation-adjusted Mueller–Hinton broth in polystyrene round-bottom 96-well plates (Sarstedt). Wells were inoculated with bacterial strains to a final density of 5×10^5 colony-forming units (CFU/mL) in a 100 µl final assay volume. Plates were incubated for 20 h at 37 °C, and minimal inhibitory concentrations (MICs) were recorded as the lowest concentrations that prevented visible growth. Growth controls contained 1% DMSO. To assess lipopolysaccharide (LPS) antagonization, MIC assays were performed in parallel in the presence or absence of 5 µM *E. coli* LPS, added during preparation of the dilution series.

Bioreporter assay for target pathway elucidation

Expression of stress-responsive marker genes was quantified using isogenic, sporulation-deficient *Bacillus subtilis* 1S34 strains carrying promoter–firefly luciferase fusions for *helD*, *yorB*, *bmrC*, *ypuA*, or *liaI*.[17] Cultures were grown in LB supplemented with 5 µg/mL erythromycin to an OD_{600} of 0.9, then diluted to an OD_{600} of 0.02 in Belitzky minimal medium (BMM; *yheI* reporter) or LB (all other reporters). Two-fold dilution series of marinocyclin A (**1**) starting at 4× MIC (16 µg/mL) were prepared in 60 µl broth in white, flat-bottom 96-well plates and inoculated with 60 µl of cell suspension. Plates were incubated at 37 °C for bioreporter-specific durations: 1 h (*ypuA*, *liaI*), 1.5 h (*yvgS*), 3.5 h (*yorB*), and 4 h (*yheI*). Luciferase activity was measured immediately after the addition of 60 µl citrate buffer (0.1 M, pH 5) containing 2 mM luciferin using a TECAN Spark reader. Antibiotics targeting the respective pathways served as positive controls (rifampicin for *helD*, ciprofloxacin for *yorB*, chloramphenicol for *bmrC,* vancomycin for *ypuA*, *liaI*).

1-*N*-Phenylnaphthylamine (NPN) uptake assay

Outer membrane permeabilization was assessed as described.[18] An exponentially growing culture of *E. coli* BW25113 in polypeptone medium was harvested by centrifugation (4,700 g, 5 min), washed, and resuspended in 5 mM HEPES buffer to an OD_{600} of 0.5. The suspension was transferred to a black, flat-bottom 96-well plate containing two-fold serial dilutions of test compounds (64–0.03 µg/mL) in 5 mM HEPES supplemented with 10 µM 1-N-phenylnaphthylamine (NPN). After 5 min of incubation at 37 °C, fluorescence was recorded using a TECAN Spark plate reader (excitation 350 nm, emission 420 nm). Permeabilization was quantified by background subtraction and normalization to untreated controls.

Membrane potential assay

Membrane depolarization was monitored in *B. subtilis* 168 using the voltage-sensitive dye $DiOC_2(3)$ (3,3'-diethyloxacarbocyanine iodide). Exponentially growing cells in LB supplemented with 0.1% glucose were harvested (4,700 g, 5 min) and resuspended in Dulbecco's phosphate-buffered saline (DPBS, Thermo Fisher) with 0.1% glucose to an OD_{600} of 0.5. Cells were stained with 30 µM $DiOC_2(3)$ for 15 min in the dark and transferred into a black, flat-bottom 96-well plate. Fluorescence was recorded in a Tecan Spark plate reader for 2 min to establish a baseline (excitation 485 nm; emission 530 nm (green) and 630 nm (red)), followed by the addition of a twofold marinocyclin A (**1**) dilution series and continued measurement for 15 min. Carbonyl cyanide *m*-chlorophenyl hydrazone (CCCP, 5 µM) and 1% DMSO served as positive and negative controls, respectively.

Cytoplasmic membrane permeabilization

Membrane permeabilization was visualized using the Live/Dead BacLight kit (Invitrogen). For microscopy of *B. subtilis* 168, exponentially growing cells in LB were treated with marinocyclin A (**1**) at 10xMIC (40 µg/mL), 4xMIC (16 µg/mL), or 1xMIC (4 µg/mL) for 30 min. A 1:1 mixture of Syto9 and propidium iodide (PI) was added (0.2 µl per 100 µl culture), followed by 15 min incubation in the dark. Samples were imaged on agarose (1%) pads using a Nikon Eclipse Ti-E microscope (excitation 472 nm, emission 520 nm (Syto9) and excitation 555 nm, emission 617 nm (PI)), equipped with an Orca Flash 4.0 LT camera (Hamamatsu). Images were acquired using NIS Elements Advanced Research (Nikon) and further analysed using NIS Elements. Statistical analysis was performed in ImageJ. For testing the eukaryotic A549 cell line, 1x 10^4 cells were seeded in 100 µl Dulbecco's Modified Eagle Medium (Gibco), 10% FBS, 1% MEM Non-Essential Amino Acids (Gibco) per well of a cellview cell culture slide (polystyrene, glass bottom, 10 compartments; Greiner) and incubated overnight (37°C, 5% CO_2, 95% relative humidity). The next day, cell culture medium was renewed and supplemented with 0.4 µl of a 1:1 mixture of Syto9 and propidium iodide (PI) as well as marinocyclin A (**1**) (4 to 64 µg/mL), polymyxin B (64 µg/mL) or colistin (64 µg/mL). Cells were imaged after 30 min of compound exposure using a Nikon Eclipse TiE microscope as described above. DMSO as

negative control, 0.1% Triton X-100 as positive control. For kinetic analysis of *B. subtilis* 168, cells were resuspended in DPBS with 0.1% glucose to an OD_{600} of 0.5, stained with 1.5 µl per mL of Syto9/PI, and transferred to a black, flat-bottom 96-well plate. Fluorescence was recorded in a TECAN Spark plate reader at 480/528 nm (Syto9) and 540/621 nm (PI) for 15 min to establish a baseline. A twofold dilution series of marinocyclin A (**1**) was added, and fluorescence was monitored for 75 min. Nisin (100 µg/mL) and 1% DMSO as positive and negative controls, respectively.

Lysis assay (Bau reporter)

Bacterial lysis was quantified using *B. subtilis* BAU-102 as described by Falk et al.[19] Cultures in TSB were grown to an OD_{600} of 0.2, and 90 µl of culture was added to a black, flat-bottom 96-well plate containing 10 µl of compound (64–0.06 µg/mL). After 5 min at room temperature (fast lysis) or 60 min at 37 °C (slow lysis), 10 µl of 4-methylumbelliferyl β-D-galactopyranoside (MUG, 400 µg/mL) was added. Plates were incubated for 60 min at room temperature in the dark, and fluorescence was measured (excitation 360 nm, emission 455 nm) in a Tecan Spark plate reader.

Peptidoglycan integrity assessment

Membrane bleb formation was assessed in *B. subtilis* 168 growing at 37 °C, 200 rpm in cation-adjusted Mueller–Hinton broth. At an OD_{600} of 0.35, cells were exposed to marinocyclin A (**1**) at 10xMIC (40 µg/mL), 4xMIC (16 µg/mL), or 1xMIC (4 µg/mL) for 30 min. Treated samples (25 µl) were added to 100 µl of acetic acid:methanol (1:3, v/v) and visualized by phase-contrast microscopy on agarose (1%) pads using a Nikon Eclipse Ti-E microscope. Images were acquired using an Orca Flash 4.0 LT camera (Hamamatsu) and NIS Elements Advanced Research (Nikon) and further analysed using NIS Elements. Vancomycin (25 µg/mL) and 1% DMSO served as positive and negative controls, respectively.

Lipid antagonization assay

Antagonization of the antibacterial activity of marinocyclins by phospholipids, cell wall precursors and lipopolysaccharide was evaluated by incubating marinocyclin A (**1**) at 4xMIC (16 µg/mL) with a four-fold molar excess of the respective lipid or precursor in 50 µl cation-adjusted Mueller–Hinton broth for 15 min at room temperature. *S. aureus* ATCC 29213 was then added to a final inoculum of $5x10^5$ CFU/mL, and OD_{600} was monitored every 10 min for 24 h using a TECAN Infinite M200 reader.

Artificial membrane vesicle permeabilization assay

For the preparation of large unilamellar vesicles (LUVs) with a head group composition resembling *B. subtilis* membranes,[20] 2 µmol lipids (Avanti Research) consisting of 81% phosphatidylglycerol (18:1), 14% phosphatidylethanolamine (16:0, 18:1), 5% cardiolipin (18:1) were added from stock solutions in $MeOH:CHCl_3$ (1:1, vol%) to 100 µl $MeOH:CHCl_3$ (1:1) followed by drying under a stream of N_2. The lipid film was resuspended in 0.1 M PBS (22.6 mM NaH_2PO_4, 77.4 mM Na_2HPO_4, 0.9% w/v NaCl, 0.02% w/v KCl; pH 7.4) containing 50 mM 5,6-carboxyfluorescein (CF) by vortexing and subsequently rehydrated for at least 30 min at a temperature above the lipid phase transition temperature (37- 40 °C). The lipid suspension was subjected to 3-5 freeze-thaw cycles, alternating between liquid nitrogen and hand-warm water, and then extruded 11 times through a 1 µm polycarbonate membrane using a mini-extruder (Avanti Research, working temperature set to 37–40 °C). Non-enclosed CF was removed by washing with 600 µl PBS and centrifugation (14,000 rpm, 4 °C, 15 min). The liposome suspension was distributed into black, flat-bottom 96-well plates, and fluorescence was measured (excitation 495 nm, emission 517 nm) in a TECAN Spark microplate reader. After 4 min of baseline measurement, compounds were added, and after 19 min, 1% Triton X-100 was added as an internal control. For lipid II-containing vesicles, 0.5% lipid II was added to the lipid mixture, and the working temperature during rehydration and extrusion was set to 60 °C.

Cytotoxicity assay

Cytotoxicity toward eukaryotic cells was quantified using a resazurin assay. In energized, metabolizing cells, resazurin is reduced to the fluorescent resorufin. Flat-bottom 96-well plates were seeded with $1x10^4$ cells per well in DMEM supplemented with 10% fetal bovine serum (FBS) and 1% MEM non-essential amino acids (Gibco) (A549 and NRK52E), or in RPMI supplemented with 10% FBS (HEK293). Cells were allowed to adhere overnight at 37 °C, 5% CO_2, and 95% relative humidity. Two-fold serial dilutions of test compounds were applied, and plates were incubated for an additional 24 h. Resazurin solution (10 µl, 1 mg/mL) was added, followed by overnight incubation. Fluorescence was measured (560 nm excitation, 600 nm emission) using a TECAN Infinite M200, and viability was calculated relative to untreated controls.

Time-kill curve assay

Time-kill assays were performed by adjusting an exponentially growing culture of *S. aureus* ATCC 29213 to an inoculum of 5x 10^5 colony-forming units (CFU/mL) and treating with marinocyclin A (**1**) at 0.25x MIC (1 µg/mL), 1xMIC (4 µg/mL), and 4xMIC (16 µg/mL). At defined time points (0 min, 15 min, 30 min, 60 min, 120 min, 240 min), culture aliquots were serially diluted 10-fold, and 10 µl of the appropriate dilutions were plated on Mueller-Hinton agar plates. After 20 h of incubation at 37 °C, the grown colonies were counted, and log10 CFU/mL was calculated.

Chemical data

Marinocyclin A (1): $[\alpha]_D^{27}$ -0.8 (c 1.5, MeOH); HRMS *m/z* 1479.8611 $[M+H]^+$, Δ+0.004amu, calcd $C_{71}H_{115}N_{16}O_{18}$, 1479.8575.
Marinocyclin B (2): $[\alpha]_D^{27}$ -1.2 (c 1.0, MeOH); HRMS *m/z* 1465.8485 $[M+H]^+$, Δ+0.007amu, calcd $C_{70}H_{113}N_{16}O_{18}$, 1465.8419.
Marinocyclin C (3): $[\alpha]_D^{27}$ –1.1 (c 0.4, MeOH); HRMS *m/z* 1521.8939 $[M+H]^+$, Δ-0.01amu, calcd $C_{74}H_{121}N_{16}O_{18}$, 1521.9044.
Marinocyclin D (4): $[\alpha]_D^{27}$ -1.1 (c 2.6, MeOH); HRMS *m/z* 1577.9571 $[M+H]^+$, Δ-0.01amu, calcd $C_{78}H_{129}N_{16}O_{18}$, 1577.9670.
Marinocyclin E (5). $[\alpha]_D^{27}$ -1.1 (c 0.8, MeOH); HRMS *m/z* 1563.9420 $[M+H]^+$, Δ-0.009amu calcd $C_{77}H_{127}N_{16}O_{18}$, 1563.9514.
Marinocyclin F (6): $[\alpha]_D^{27}$ -2.2 (c 0.2, MeOH); HRMS *m/z* 1226.6768 $[M+H]^+$, Δ-0.001amu, calcd $C_{58}H_{92}N_{13}O_{16}$, 1226.6785.
Marinocyclin G (7): $[\alpha]_D^{27}$ -3.2 (c 0.3, MeOH); HRMS *m/z* 1324.7988 $[M+H]^+$, Δ+0.01amu, calcd $C_{65}H_{106}N_{13}O_{16}$, 1324.7805.

DNA sequences.

His$_6$-MarE (expected MW = 36276)

ATGCACCACCACCACCATCATAATGAATTCCAAGTAATTCAAGAGAAACAAATATTAGAGCACATTCCGAT
ACGCAACGGAATCACTCGTAAGGAATTCTACCAAGACTTCGTTAACAAAGGTCAACCTGTGGTTATCCGAG
GGATGACCTCAGGGTGGAATGCGCTGGAATGGGACGTGGATTACTTCAAGGCCAAAGAGACAGGCGTTAAA
ATCCCAATTAAGGTTGGCAAGGTGAGCGAAGGCCGTCGGGAGATGATGTTAATTAGCCAATATGTGGAACT
GCTTGACGCATATGAAAAGAATGTTAAATTGGGGCTCGACGCTGACCCGGTGGGTTATTTACACGACATAC
CATTCTTCTACATGTTCCCTGAATATTTAGCTGACATATTACCATTTCCTAAAGAGTTACTCCCGGAGTGG
TATTGGCCAAAATGGCAAAACTATATTCAGTTCTTCATGGGCGCAACAGGAAGCTTGACACCGCTGCACTT
CGATACCTTACTGACCCACAACCTTTTCTTCCAAGTGGTAGGTCGAAAGCAATTCATTCTGATCGATGGTG
CTCAAAAGGAGCAATGTTACATTAAAGGCTGGCGGTGGTCTCGATTTGACCCAAAGAACCCAGATTATAAG
AAATTCCCTAAGGCTAAGGATTTGAATATAAAAGAGGCAATCCTGGAGCCTGGGGACATTCTCTACATGCC
ATCTGGCATGTTGCACCAGGTACACGGATTAAGCCAATCAATAAGCTTTAATATTGATTGGCACACGGTCA
AAAGTGCGAAGAAGGGTGTGGGGACCCTTTGGAAGGGTGCTCCATTGAAGAATGTGTACTACAATGCGTTG
GTGTACGCGGGATTAAAACTGAAAGTGTCGGAGAAATCTATTTTCCCACTTTACAAATCATACTTAAACTA
CATATCATGA

His$_6$-bdSUMO-MarE (expected MW = 45596)

ATGCACCACCACCATCACCACATGGGAAGCTCTCACATTAATTTAAAGGTTAAGGGGCAAGACGGGAACGA
GGTTTTCTTCCGAATAAAGCGTAGTACACAACTGAAGAAGCTGATGAACGCTTATTGTGACAGACAAAGCG
TGGACATGACCGCCATTGCCTTCTTATTCGATGGTCGTCGATTACGTGCGGAGCAAACACCCGACGAGCTT
GAGATGGAAGACGGCGACGAGATCGACGCCATGTTGCATCAGACCGGCGGCATGAACGAATTCCAAGTTAT
CCAGGAGAAACAAATATTGGAGCACATTCCAATCCGCAACGGCATTACACGAAAGGAGTTTTACCAGGACT
TTGTAAATAAAGGACAACCTGTCGTAATCCGCGGTATGACTAGTGGTTGGAATGCTCTGGAGTGGGACGTT
GACTACTTTAAGGCGAAGGAAACGGGCGTTAAAATCCCAATAAAGGTAGGAAAGGTTTCGGAAGGCCGGCG
TGAAATGATGTTAATTTCTCAGTACGTAGAGTTATTAGACGCGTACGAAAAGAACGTAAAGTTGGGGTTAG
ATGCTGATCCAGTTGGCTACTTGCACGACATCCCATTCTTCTATATGTTCCCCGAGTATCTGGCGGACATC
TTACCCTTCCCCAAGGAGTTGTTACCTGAGTGGTACTGGCCGAAATGGCAAAATTACATACAGTTCTTCAT
GGGAGCTACTGGCAGTCTGACTCCTCTGCATTTTGATACGCTCTTAACGCACAACCTGTTCTTCCAGGTAG
TAGGGCGCAAACAATTCATTTTGATCGACGGCGCGCAAAAGGAGCAATGTTACATCAAGGGCTGGCGCTGG
TCACGCTTCGACCCCAAGAATCCTGATTATAAGAAGTTTCCCAAGGCAAAGGATCTTAACATTAAGGAAGC
AATTTTGGAACCAGGTGACATCCTTTATATGCCATCCGGTATGTTACACCAAGTTCATGGGCTTTCTCAGT
CTATATCATTCAATATTGACTGGCATACAGTTAAGTCCGCCAAGAAGGGTGTGGGTACCCTTTGGAAAGGG
GCGCCCTTGAAGAACGTATACTACAACGCGTTAGTCTACGCCGGGTTGAAGCTGAAGGTTTCTGAGAAGAG
CATATTCCCACTTTACAAGTCCTACTTAAATTACATCTCCTAA

His$_6$-MarF (expected MW = 62658)

ATGCACCACCATCACCACCACAATACAAAGGTATACCTGAAGCCGAACGTCGTAATTGAACCTTTGTTCGA
CAATTGGTATGCGTGGAGCCACTTGATTAGTCCAGCCACTGGCGCGATGAACGTAGTAGGTCGTCACCTCA
AGATAATGTCTAGCTACATCCAAGCACCGGCCGCTCATGCCGCTGCGGTGAAGAATCCGAAAATGCTTGGC
GGCCCGTTCATTGATTACGGTGGTAAGCGTGTTGATGAAATCAAGAACCTTAAGGAAGAGACATTGCAGAA
ACAAAAGGACTTAATCGCCTTTAACGATGCGGTCAAAGAACTGGACGCCTTATTAAAGTCTAAGGCAAAGG
GTTTCAGTCTGGAACCTCTTTATGACGAAGTACCGGAGATACTGCAAGGCTACGTGGAATTGATATACGAT
CTGAATAACCAACCAAGTTTTCGCTTCTTCGAATCGTTACTGTACAAGAGTAAATTTTACAACCCAGACAG
TCAGTCTATCTTACTCTGGATTACTGAGAATGATGAACGCCCATTCGTCCTGAGTACCCCTAGATTACCAG
ATAGTAATCAAGTCCATCTGGACATTCCATTCGATCACAAGGGCCTTGACAAGTTATCACAAATGAAGCGC
ATACCGGGTTCTGTTTCAGAGATCAAAGAGCTCCTTGGCATATCAAAGGAGCAAGAGGAAGTGTTCGACAC
CTTCTTCACAACAGAACCACCAAAAGAGTACGATCAGTATACAGGTGATAAGGTTCGTATGCGTTACTTCG
GTCATGCAAGCATCCTGGTTGAGACGAAGGACATCAGTATAATGCTCGACCCTGTTATATCATATTACGGA
TATCATCAGGATGTCAGTAGATTCAGCGACATGGACCTGCCCGAGGTTATTGATTACGTGTTAATTACCCA
CAACCACCAAGACCACATATTATTCGAAACCCTCATTCCATTACGCCATAAAATTAAGAACATTGTTGTAC

CTAGATCCAGCAACGGTTCATTGCAAGACCCAAGTTTAAAGTTAATGTTACAAGCCATTGGTTTCGAGAAT
GTCATCGAAATAGACGAGATGGAAGCGATACATTTTAACGACTGTACTATTACCGGTGTTCCCTTCATCGG
CGAACATAGCGATCTGAACATCCAATGCAAGATCTGCCACCACGTCGCAGTTGGTGATTTTAAGATGTTAT
TCGTGGCGGACAGCTGTAACGTAGCCGCTAAGCTTTATGAGCATGTTCAAGCAGAGCTTGGTGACGTGGAC
GTCTTGTTCTTAGGGATGGAGTGCGATGGTGCGCCATTAAGCTGGCTCTATGGACCGCTCCTCACAGAGAC
TATAGACCGAGATAAAGATTTCTCACGCCGTCTGGCAGGCTCAAACTTCGAAAGAGGTAAGGCTCTTGTCG
AGGCGTTCTCCCCAAAGGAAGTGTACGTATATGCGATGGGTCAAGAACCATGGTTAGAGTTCATCAGCAGC
ATCAAATATACTGACGAATCAAACCCAATCATACAGAGCGATAAGCTTGTTCAATATTGTATGGACGGGAA
CATTATTAGTGAACGATTATTCGGAGAGAAGGAGATTTTATACAACAAGACCGAGGAGTCCAAGAAGGTTC
TTGAGTCTGTTTGA

Supplementary tables

Table S1. BGC types on the genome of *A. megaterium* EL43 predicted by antiSMASH. The marinocyclin BGC is highlighted in bold.

**BGC type**	**Most similar known cluster based on MIBiG analysis**	**Similarity**
Arylpolyene	flexirubin	77%
Siderophore	fulvivirgamides.	100%
RiPP		
Terpene		
Trans-AT PKS	cuniculene	100%
**NRPS**		
Terpene	carotenoid	28%
RiPP		
Type III PKS		
RiPP		

Table S2. Proteins of the marinocyclin (*mar*) biosynthetic gene cluster.

Protein	Protein size [aa]	Predicted function	Interpro hits	Closest UniProtKS/Swiss-Prot homolog (organism)	% Similarity	Accession number
**MarA**	470	Aminotransferase	IPR005814 (aminotransferase class-III)	Diaminobutyrate 2-oxoglutarate aminotransferase RhbA (*Sinorhizobium meliloti* 1021)	57%	Q9Z3R2.1
**MarB**	7149	Non-ribosomal peptide synthetase	Multiple	Tyrocidine synthase 3 TycC (*Brevibacillus parabrevis*)	52%	O30409.1
**MarC**	5619	Non-ribosomal peptide synthetase	Multiple	Tyrocidine synthase 3 TycC (*Brevibacillus parabrevis*)	56%	O30409.1
**MarD**	432	Major facilitator transporter	IPR011701 (major facilitator superfamily)	Lysophospholipid transporter LplT (*Yersinia enterocolitica* 8081)	46%	A1JPE9.1
**MarE**	304	Fe(II)/α-ketoglutarate enzyme	IPR056520 (cupin-like domain 8)	Lysine-specific demethylase JMJ30 (*Arabidopsis thaliana*)	49%	Q8RWR1.1
**MarF**	542	Diiron non-heme oxidoreductase	IPR041141 (diiron non-heme beta-hydroxylase, N-terminal domain)	4-Amino-L-phenylalanyl-[CmlP-peptidyl-carrier-protein] 3-hydroxylase (*Streptomyces venezuelae* ATCC 10712)	58%	F2RB80.1
**MarG**	252	Thioesterase	IPR029058 (alpha/beta hydrolase fold)	Gramicidin S biosynthesis protein GrsT [*Aneurinibacillus migulanus*]	53%	P14686.1
**MarH**	445	Asparte kinase	IPR001341 (aspartate kinase)	Aspartate kinase (*E. coli* K-12)	49%	P08660.2

Table S3. Comparison of antiSMASH 8.0.4- and PRISM 4-predicted adenylation (A) domain substrate specificities of the *mar* NRPS and amino acid residues present in marinocyclins.

A domain	Gene	antiSMASH prediction	PRISM 4 prediction	Amino acid present in marinocyclin
A1	*marB*	?	Dap	L-DAHB
A2	*marB*	Thr	Thr	Dhb
A3	*marB*	Leu	Leu	L-Leu
A4	*marB*	Ser	Ser	D-Ser
A5	*marB*	?	Ser	L-Dab
A6	*marB*	?	Dap	L-DAHB
A7	*marB*	Phe	Phe	L-Phe
A8	*marC*	Ile	Ile	D-Ile
A9	*marC*	Orn	Orn	L-Orn
A10	*marC*	Asn	Glu	L-Asn-β-OH
A11	*marC*	Phe	Phe	L-Phe-β-OH

Dap: 2,3-diaminopropionic acid, Dhb: dehydrobutyrine, DAHB: 2,4-diamino-3-hydroxybutyric acid, Dab: 2,4-diaminobutyric acid

Table S4. NMR data for marinocyclin A (**1**) in methanol-d4 and DMSO-d6 (^{1}H 600 MHz, ^{13}C150 MHz)

		Methanol-d6		DMSO-d6	
No.	Amino acid residues	^{13}C/HSQC	^{1}H NMR	^{13}C/HSQC	^{1}H NMR
1	Phe-β-OH	172.1, C			
2		63.2, CH	4.22 (m)	59.0, CH	4.19 (m)
3		73.3, CH	5.02 (d), *J*=5.7 Hz	71.1, CH	5.10 (m)
4		142.0, C		141.3, C	
5/9		127.2, CH	7.40 (m)	126.2, CH	7.34 (m)
6/8		129.3, CH	7.32 (m)	127.8, CH	7.29 (m)
7		129.1, CH	7.34 (m)	126.5, CH	7.26 (m)
NH					7.29 (m)
1	Asn-β-OH	173.2, C			
2		56.5, CH	4.90 (m)	55.9, CH	4.37 (m)
3		71.5, CH	4.57 (m)	69.9, CH	4.22 (m)
4		176.2, C			
NH					7.32 (m)
1	Orn	171.6, C			
2		54.9, CH	4.63 (dd), *J* = 10.1, 5.0 Hz	50.9, CH	4.65 (m)
3		31.4, CH_2	1.78 (m), 1.83 (m)	28.8, CH_2	1.66 (m), 1.75 (m)
4		24.8, CH_2	1.62 (m), 1.67 (m)	23.6, CH_2	1.45, (m), 1.53 (m)
5		39.8 CH_2	2.95, (m)	36.9, CH_2	2.80 (m)
NH					7.66 (m)
1	Ile	173.8, C			
2		57.4, CH	4.24 (d), *J* = 3.7 Hz	55.1, CH	4.17 (m)
3		36.0, CH	1.99 (m)	35.2, CH	1.86 (m)
4		26.6, CH_2	0.54, (dt), *J* = 14.3, 7.2 Hz 0.44 (dq), *J* = 7.9, 15.6 Hz	25.2, CH_2	0.59, (m) 0.63 (m)
5		12.0, CH_3	0.63 (t), J= 7.4 Hz	11.5, CH_3	0.59 (m)
6		14.1	0.60 (d), J= 6.9 Hz	14.1	0.56 (m)
					8.12 (m)
1	Phe	174.4 C			
2		58.0, CH	4.576 (dd), *J*=5.6, 11.3 Hz	54.8, CH	4.62 (m)
3		37.8, CH	2.96 (m), 3.14 (m)	36.9, CH	2.80 (m), 2.96 (m)
4		136.8, C		136.0, C	
5/9		130.1, CH	7.23 (m)	128.7, CH	7.26 (m)
6/8		129.9, CH	7.31 (m)	127.8, CH	7.29 (m)

7		128.1, CH	7.25 (m)	126.4, CH	7.20 (m)
NH					8.41(Br s)
1	DAHB	174.6, C			
2		56.8, CH	4.48 (d), *J* = 8.6 Hz	55.9, CH	4.37 (m)
3		69.7, CH	4.16 (m)	67.2, CH	4.09 (m)
4		43.0, CH_2	2.98 (m), 3.15 (m)	40.9	2.90 (m)
NH					8.24 (d)), *J* = 6.8 Hz
1	Dab	171.3			
2		53.2, CH	4.17 (m)	55.9, CH	4.43 (m)
3		32.8, CH_2	1.73 (m), 1.92 (m)	31.7, CH_2	1.64 (m), 1.87 (m)
4		36.3, CH_2	2.93 (m), 3.38 (m)	36.9 CH_2	3.02 (m)
NH					7.97 (m)
4-NH					7.37 (m)
1	Ser	173.6, C			
2		57.4, CH	4.40 (m)	55.3, CH	4.32 (m)
3		62.9, CH_2	3.87 (dd), *J*=4.7, 10.0 Hz 3.90(dd), *J*=4.6, 10.0 Hz	61.8, CH_2	3.61 (m)
NH					8.01 (d), *J* = 7.0 Hz
1	Leu	172.1, C			
2		53.4, CH	4.46 (m)	51.4, CH	4.31 (m)
3		40.6, CH_2	1.72(m)	40.0, CH_2	1.50 (m), 1.56 (m)
4		25.8, CH	1.72 (m)	23.9, CH	1.65 (m)
5		21.5, CH_3	0.95 (d), *J* = 6.1 Hz	22.4, CH_3	0.84 (d), J = 6.5 Hz
6		23.3, CH_3	0.98 (d), *J* = 6.1 Hz	23.1, CH_3	0.87 (d), *J* = 6.5 Hz
NH					7.93 (d) *J* = 7.9 Hz
1	Dhb	167.5, C			
2		131.4, C		131.0, C	
3		126.2, CH	5.85 (q), *J* = 7.3 Hz	122.8, CH	5.75 (q), *J*= 7.4 Hz
4		13.4 CH_3	1.91 (d), *J* = 7.3 Hz	12.9 CH_3	1.81 (d), *J* = 7.4 Hz
NH					9.62 (s)
1	DAHB	170.8, C			
2		57.4, CH	4.40 (dd), *J* = 7.4 Hz	55.9, CH	4.37 (m)
3		69.1, CH	4.13 (m)	67.6, CH	3.95 (m)
4		43.1, CH_2	2.97 (m), 3.14 (m)	41.3, CH_2	2.78 (m), 2.90 (m)
NH					8.14 (m)
1		177.1, C			
2		36.4, CH_2	2.33 (m)	35.0, CH_2	2.16 (m)

3	26.5, CH_2	1.63 (m)	25.0, CH_2	1.48 (m)
4	29,1, CH_2	1.32 (m)	28.8, CH_2	1.23 (m)
5-8	30.5, CH_2	1.29-1.31 (m)	28.8, CH_2	1.23(m)
9	28.2, CH_2	1.28 (m)	26.6, CH_2	1.22 (m)
10	40.1, CH_2	1.17 (m)	38.4, CH_2	1.12 (m)
11	28.9, CH	1.51 (m)	27.3, CH	1.48 (m)
12/13	22.8, CH_3	0.88 (d)J=6.7 Hz	21.3, CH_3	0.83(d) *J*=6.5 Hz

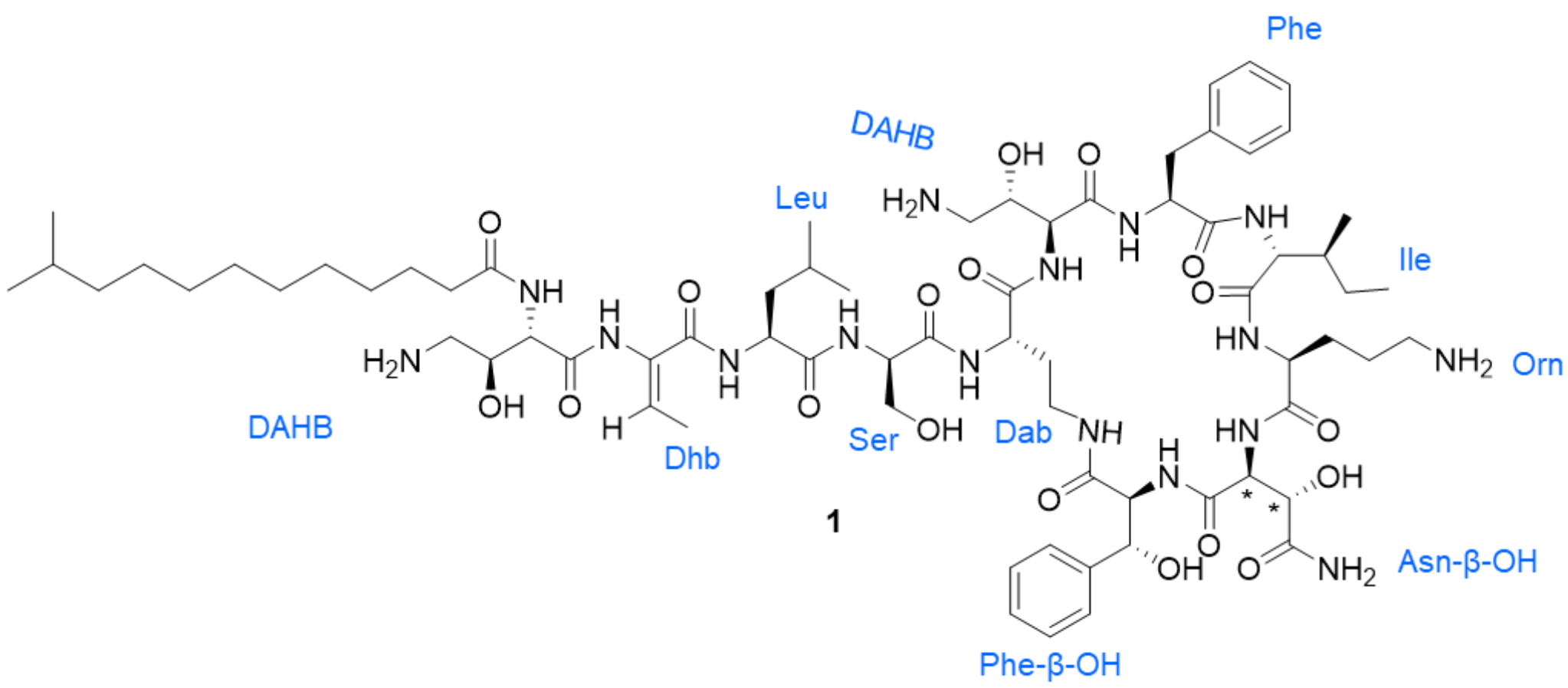

1

Table S5. NMR data for marinocyclin B (**2**) in methanol-d4 (^{1}H 600 MHz, ^{13}C150 MHz)

No.	Amino acid residues	^{13}C/HSQC	^{1}H NMR
1	Phe-β-OH	172.2, C	
2		63.8, CH	4.21 (m)
3		73.5, CH	5.02 (d) *J* = 5.4 Hz
4		142.2, C	
5/9		127.3, CH	7.40 (m)
6/8		129.7, CH	7.30 (m)
7		129.3, CH	7.34 (m)
1	Asn-β-OH	173.1, C	
2		56.3, CH	4.93 (m)
3		71.7, CH	4.58 (m)
4		176.5C	
1	Orn	171.6, C	
2		55.1, CH	4.62 (m), *J* = 10.4, 5.3 Hz
3		31.7, CH_2	1.79 (m), 1.85 (m)
4		24.9, CH_2	1.66 (m), 1.68 (m)
5		40.0 CH_2	2.96, (m)
1	Ile	173.5, C	
2		57.5, CH	4.24 (dd), *J* = 8.1, 3.6 Hz
3		36.1, CH	1.98 (m)
4		26.7, CH_2	0.43, (m)<br>0.53 (m)
5		12.2, CH_3	0.63 (t), *J*= 7.4 Hz
6		14.3, CH_3	0.60 (d), *J*= 7.3 Hz
1	Phe	174.3, C	
2		58.1, CH	4.56 (dd), *J*=5.0, 11.5 Hz
3		38.0, CH	2.92 (m), 3.16 (m)
4		136.9, C	
5/9		130.3, CH	7.23 (m)
6/8		129.7, CH	7.29 (m)
7		128.2, CH	7.23 (m)
1	DAHB	174.8, C	
2		57.2, CH	4.49 (d), *J* = 7.7 Hz
3		69.7, CH	4.18 (m)
4		43.1	2.98 (m), 3.16 (m)
1	Dab	171.3, C	
2		53.3, CH	4.14 (m)
3		32.8, CH_2	1.72 (m), 1.92 (m)
4		36.3, CH_2	2.94 (m), 3.40 (m)
1	Ser	173.8, C	
2		57.5, CH	4.41 (m)

3		63.1, CH_2	3.87 (dd), *J*=4.9, 9.7 Hz<br>3.90 (dd), *J*=4.5, 9.7Hz
1	Leu	171.8, C	
2		53.5, CH	4.46 (m)
3		40.8, CH_2	1.73 (m)
4		25.9, CH	1.73 (m)
5		21.7, CH_3<br>23.5, CH_3	0.95 (d), *J* = 6.4 Hz<br>0.99 (d), *J* = 6.4 Hz
1	Dhb	167.4 C	
2		131.0, C	
3		126.1, CH	5.85 (q), *J* = 7.4 Hz
4		13.5 CH_3	1.90 (d), *J* = 7.4 Hz
1	DAHB	170.7, C	
2		57.5, CH	4.42 (d), *J* = 7.5 Hz
3		69.10,	4.15 (m)
4		43.1	2.95 (m), 3.14 (m)
1		176.8, C	
2		36.7, CH_3	2.32 (m)
3		26.4, CH_2	1.63 (m)
4-7		30.3, CH_2	1.29-1.33 (m)
8		26.4, CH_2	1.33 (m)
9		40.2, CH_2	1.18 (m)
10		28.7, CH	1.52 (m)
11/12		22.8, CH_3	0.88 (d)J=6.7 Hz

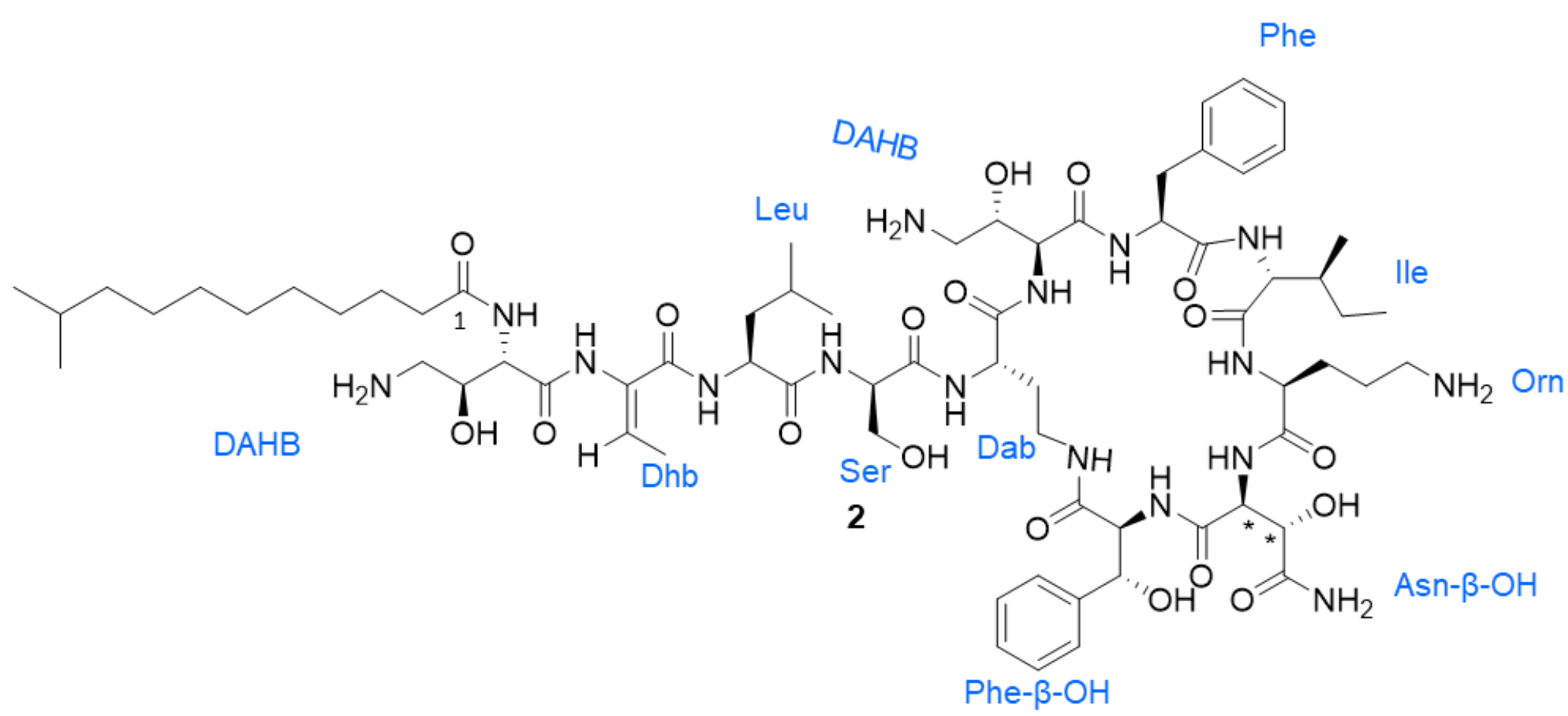

2

Table S6. NMR data for marinocyclin C (**3**) in methanol-d4 (^{1}H 600 MHz, ^{13}C150 MHz)

No.	Amino acid residues	^{13}C/HSQC	^{1}H NMR
1	Phe-β-OH	172.1, C	
2		63.3, CH	4.22 (m)
3		73.5, CH	5.04 (d), *J*=5.8 Hz
4		142.1, C	
5/9		127.3, CH	7.40 (m)
6/8		129.3, CH	7.31 (m)
7		129.3, CH	7.34 (m)
1	Asn-β-OH	173.2, C	
2		56.7, CH	4.89 (m)
3		71.6, CH	4.59 (m)
4		176.1, C	
1	Orn	171.5, C	
2		55.0, CH	4.63 (m)
3		31.6, CH_2	1.79 (m), 1.83 (m)
4		24.8, CH_2	1.62 (m), 1.66 (m)
5		39.9 CH_2	2.95, (m)
1	Ile	173.8, C	
2		57.5, CH	4.26 (d) *J* = 3.6 Hz
3		36.0, CH	1.99 (m)
4		26.5, CH_2	0.54, (dt), *J* = 7., 15.2 Hz<br>0.44 (dq), *J* = 7.9, 15.9 Hz
5		12.0, CH_3	0.63 (t), *J*= 7.4 Hz
6		14.1	0.60 (d), *J*= 7.1 Hz
1	Phe	174.6 C	
2		58.2, CH	4.576 (dd), *J*=5.9, 10.8 Hz
3		37.8, CH	2.96 (m), 3.14 (m)
4		136.8, C	
5/9		130.2, CH	7.24 (m)
6/8		129.9, CH	7.31 (m)
7		128.3, CH	7.25 (m)
1	DAHB	174.6, C	
2		56.8, CH	4.48 (d), *J* = 8.4 Hz
3		69.8, CH	4.14 (m)
4		43.1, CH_2	2.99 (m), 3.12 (m)
1	Dab	171.3	
2		53.2, CH	4.18 (m)
3		33.0, CH_2	1.73 (m), 1.88 (m)
4		36.3, CH_2	2.96 (m), 3.37 (m)
1	Ser	173.8, C	
2		57.4, CH	4.38 (m)

3		63.1, CH_2	3.89 (dd), *J*=4.8, 11.2 Hz<br>3.94(dd), *J*=4.9, 11.4 Hz
1	Leu	171.9, C	
2		53.5, CH	4.42 (m)
3		40.8, CH_2	1.73 (m)
4		25.9, CH	1.70 (m)
5		21.4, CH_3	0.95 (d), *J* = 6.3 Hz
		23.5, CH_3	0.98 (d), *J* = 6.4 Hz
1	Dhb	167.3, C	
2		130.5, C	
3		128.3, CH	5.85 (q), *J* = 7.1 Hz
4		13.7 CH_3	1.95 (d), *J* = 7.3 Hz
6		30.5, CH_2	1.29 (m)
1	DAHB	172.7, C	
2		58.0, CH	4.40 (dd), *J* = 6.8 Hz
3		71.3, CH	3.95 (m)
4		43.5, CH_2	2.99 (m), 3.11 (m)
1		177.1, C	
2		36.6, CH_3	2.32 (m)
3		26.4, CH_2	1.63 (m)
4		30.1, CH_2	1.33 (m)
5-11		30.5, CH_2	1.30-1.33 (m)
12		28.4, CH_2	1.28 (m)
13		40.3, CH_2	1.17 (m)
14		29.0, CH	1.52 (m)
15/16		22.9, CH_3	0.88 (d)J=6.7 Hz

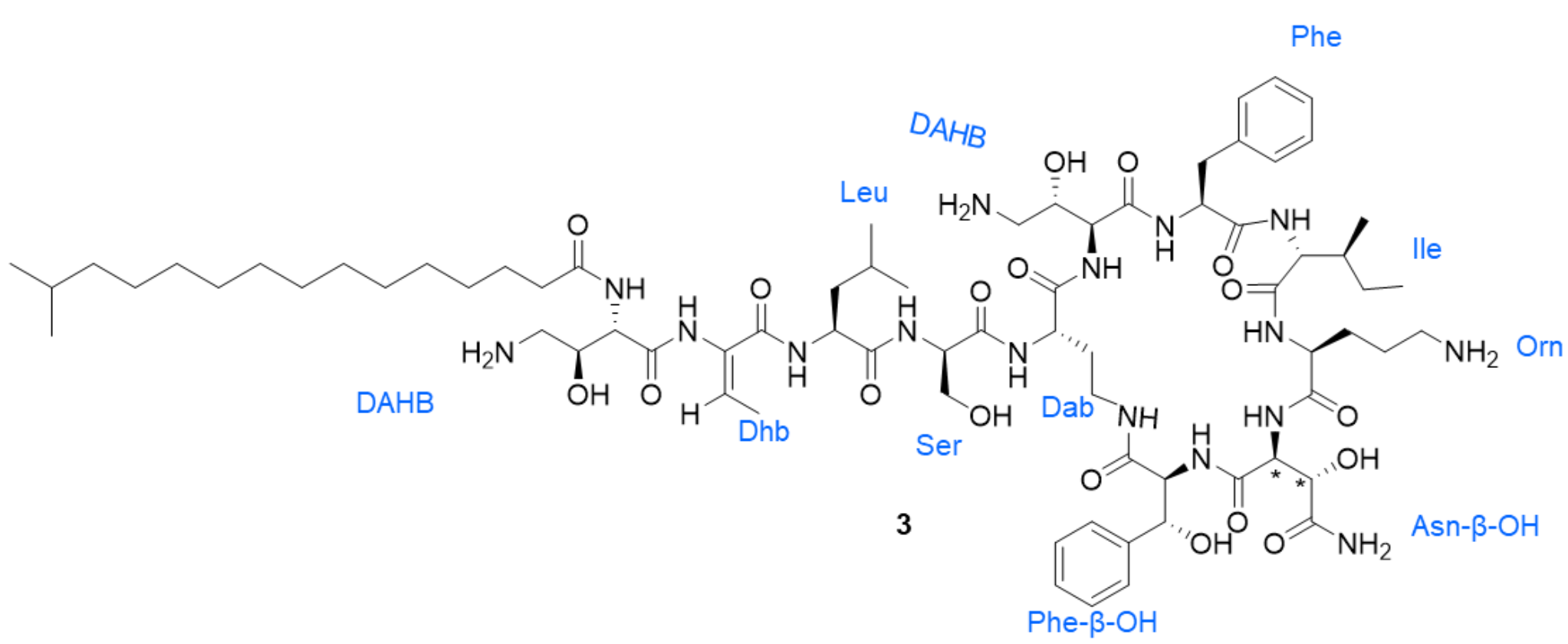

3

Table S7. NMR data for marinocyclin D (**4**) in methanol-d4 (^{1}H 600 MHz, ^{13}C 150 MHz)

		DMSO		MeOH	
No.	Amino acid residues	^{13}C/HSQC	^{1}H NMR		
1	Phe-β-OH			172.3, C	
2		59.0, CH	4.19 (m)	63.3, CH	4.22 (m)
3		71.1, CH	5.11 (m)	73.5, CH	5.02 (d), *J*=5.5 Hz
4		141.2, C		142.1, C	
5/9		126.2, CH	7.34 (m)	127.3, CH	7.39(m)
6/8		128.0, CH	7.29 (m)	129.6, CH	7.30 (m)
7		126.9, CH	7.27(m)	129.3, CH	7.35 (m)
OH			5.90(m)		
NH			7.26 (m)		
1	Asn-β-OH			173.2, C	
2		55.8, CH	4.37 (m)	56.6, CH	4.92 (m)
3		69.9, CH	4.22 (m)	71.6, CH	4.58 (m)
4				176.5, C	
NH			7.31 (m)		
OH			6.09 (m)		
1	Orn			171.6, C	
2		51.1, CH	4.67 (m)	55.0, CH	4.63 (dd), *J* = 10.2, 5.2 Hz
3		28.9, CH_2	1.70 (m), 1.75 (m)	31.5, CH_2	1.79 (m), 1.82 (m)
4		23.3, CH_2	1.45, (m), 1.55 (m)	25.0, CH_2	1.63 (m), 1.68 (m)
5		37.1, CH_2	2.82 (m)	40.0 CH_2	2.95, (m)
NH			7.64 (m)		
1	Ile			173.4, C	
2		55.1, CH	4.17 (m)	57.5, CH	4.25 (t), *J*= 3.9 Hz
3		35.3, CH	1.87 (m)	36.1, CH	1.99 (m)
4		25.2, CH_2	0.60, (m) 0.63 (m)	26.6, CH_2	0.53, (m) 0.43 (m)
5		11.7, CH_3	0.59 (m)	12.1, CH_3	0.63 (t), *J*= 7.7 Hz
6		14.1	0.58 (m)	14.3	0.61 (d), *J*= 6.9 Hz
NH			7.64 (m)		
1	Phe			174.2C	
2		54.8, CH	4.62 (m)	58.1, CH	4.57 (dd), *J*=5.5, 11.9 Hz
3		36.9, CH	2.81 (m), 2.99 (m)	37.8, CH	2.96 (m), 3.14 (m)

4		136.2, C		136.8, C	
5/9		128.5, CH	7.27 (m)	130.3, CH	7.24 (m)
6/8		128.0, CH	7.28 (m)	129.6, CH	7.31 (m)
7		126.4, CH	7.20 (m)	128.3, CH	7.25 (m)
NH			8.41 (m)		
1	DAHB			174.6, C	
2		55.8, CH	4.40	56.9, CH	4.47 (d), *J* = 7.9 Hz
3		67.0, CH	4.10 (m)	70.0, CH	4.16 (m)
4		40.6	2.89 (m)	43.1, CH_2	2.97 (m), 3.14 (m)
NH			8.28 (m)		
OH			5.77 (m)		
1	Dab			171.0	
2		55.8, CH	4.44 (m)	53.3, CH	4.17 (m)
3		31.1, CH_2	1.65 (m), 1.91 (m)	32.4, CH_2	1.76 (m), 1.92 (m)
4		36.9 CH_2	3.03 (m)	40.0, CH_2	
NH			7.97 (m)		
4-NH			7.34 (m)		
1	Ser			173.7 C	
2		55.1, CH	4.33 (m)	57.3, CH	4.37 (m)
3		61.7, CH_2	3.61 (m)	62.9, CH_2	3.87 (dd), *J*=4.6, 11.2 Hz<br>3.90 (dd), *J*=5.1, 11.2 Hz
NH			7.98 (m)		
OH			4.87 (m)		
1	Leu			171.8, C	
2		51.4, CH	4.32 (m)	53.7, CH	4.45 (m)
3		40.2, CH_2	1.52 (m), 1.57 (m)	40.8, CH_2	1.72(m)
4		24.1, CH	1.66 (m)	25.9, CH	1.72 (m)
5		22.5, CH_3	0.84 (d), J = 6.5 Hz	21.6, CH_3	0.96 (d), *J* = 6.2 Hz
6		23.1, CH_3	0.87 (d), *J* = 6.5 Hz	23.4, CH_3	0.99 (d), *J* = 6.2 Hz
NH			7.96 (m)		
1	Dhb	167.4 C		167.6 C	
2		131.0, C		130.9, C	
3		122.4, CH	5.76 (m)	126.5, CH	5.87 (q), *J* = 7.4 Hz
4		13.0 CH_3	1.82 (d), *J* = 7.3 Hz	13.6 CH_3	1.91 (d), *J* = 7.4 Hz
NH			9.61 (s)		
1	DAHB	170.7, C		170.8, C	
2		55.8, CH	4.38 (m)	57.4, CH	4.41 (dd),

				J = 2.0, 7.4 Hz
3	67.5, CH	3.95 (m)	69.0, CH	4.13 (m)
4	41.3	2.78 (m)	43.1, CH_2	2.97 (m), 3.14 (m)
NH		8.14 (m)		
OH		5.83 (m)		
1	173.1, C		177.1, C	
2	35.0, CH_2	2.17 (m)	36.6, CH_3	2.32 (m)
3	25.0, CH_2	1.48 (m)	26.5, CH_2	1.63 (m)
4-15	28.6, CH_2	1.23-1.25 (m)	30.5, CH_2	1.29-1.33 (m)
16	26.6, CH_2	1.23 (m)	28.3, CH_2	1.28 (m)
17	38.9, CH_2	1.13 (m)	40.2, CH_2	1.17 (m)
18	27.4, CH	1.49 (m)	28.9, CH	1.53 (m)
19/20	21.3, CH_3	0.82 (d) J=6.5 Hz	22.9, CH_3	0.88 (d) J=6.7 Hz

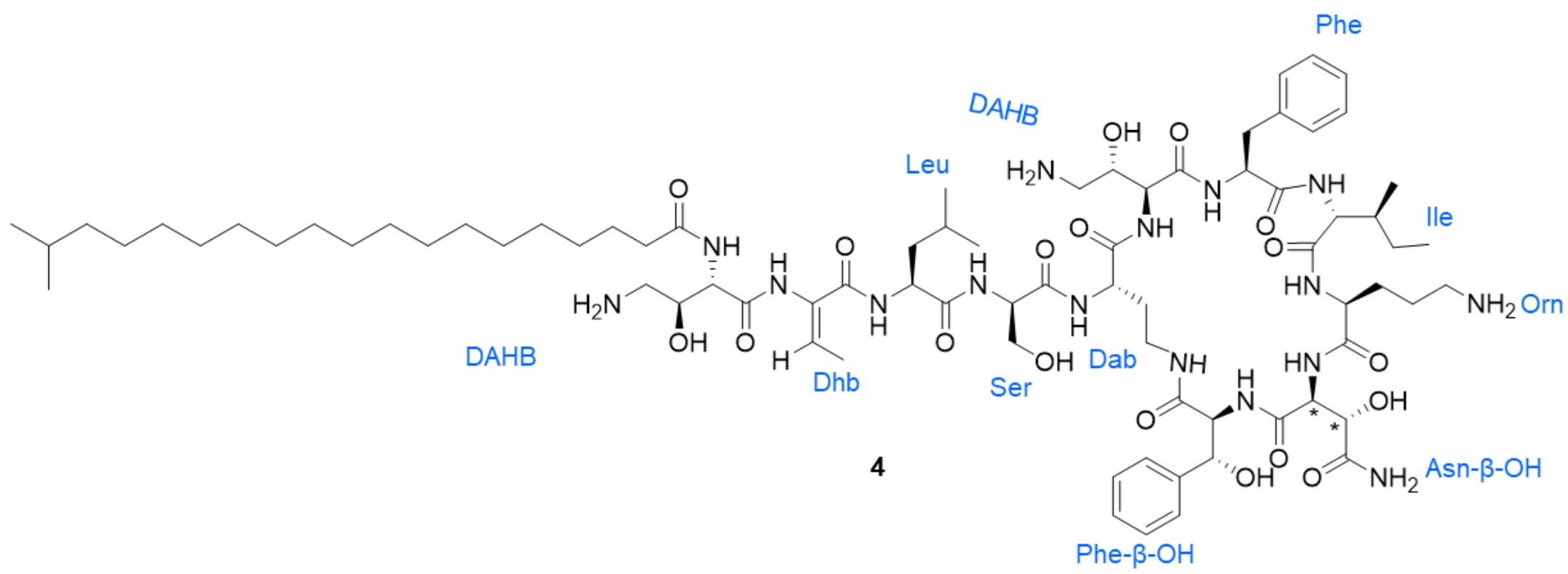

4

Table S8. NMR data for marinocyclin E (**5**) in methanol-d4 (^{1}H 600 MHz, ^{13}C 150 MHz).

No.	Amino acids residues	^{13}C/HSQC	^{1}H NMR
1	Phe-β-OH	172.1, C	
2		63.8, CH	4.21 (m)
3		73.3, CH	5.01 (d), *J* = 5.7 Hz
4		142.1, C	
5/9		127.31 CH	7.40 (m)
6/8		129.76 CH	7.28 (m)
7		129.2, CH	7.34 (m)
1	Asn-β-OH	173.1, C	
2		56.4, CH	4.91 (m)
3		71.4, CH	4.56 (m)
4		176.5, C	
1	Orn	171.6, C	
2		54.8, CH	4.63 (m), *J* = 10.0, 5.1 Hz
3		31.4, CH_2	11.84 (m)
4		24.5, CH_2	1.66 (m)
5		40.0 CH_2	2.96, (m)
1	Ile	173.5, C	
2		57.5, CH	4.24 (m)
3		35.9, CH	1.98 (m)
4		26.8, CH_2	0.45, (m)<br>0.55 (m)
5		12.1, CH_3	0.63 (t), *J*= 7.0 Hz
6		14.0, CH_3	0.60 (d), *J*= 7.2 Hz
1	Phe	174.3, C	
2		58.0, CH	4.56 (m)
3		37.9, CH	2.96 (m), 3.14 (m)
4		136.7, C	
5/9		130.0, CH	7.23 (m)
6/8		129.6, CH	7.30 (m)
7		128.1, CH	7.24 (m)
1	DAHB	174.9, C	
2		56.7, CH	4.49 (d), *J* = 8.0 Hz
3		69.6, CH	4.18 (m)
4		43.0	2.99 (m), 3.14 (m)
1	Dab	171.3, C	
2		53.3, CH	4.15 (m)
3		33.0, CH_2	1.71 (m), 1.93 (m)
4		36.3, CH_2	2.91 (m), 3.40 (m)
1	ser	173.7, C	
2		57.4, CH	4.41 (m)

3		62.9, CH_2	3.87 (dd), *J*=5.1, 10.5 Hz<br>3.90 (dd), *J*=4.5, 10.7Hz
1	Leu	171.8, C	
2		53.5, CH	4.46 (m)
3		40.8, CH_2	1.71 (m)
4		25.6, CH	1.72 (m)
5		21.4, CH_3	0.95 (d), *J* = 6.1 Hz
		23.2, CH_3	0.99 (d), *J* = 6.1 Hz
1	Dhb	167.5 C	
2		131.0, C	
3		126.0, CH	5.85 (q), *J* = 7.4 Hz
4		13.4 CH_3	1.91 (d), *J* = 7.4 Hz
1	DAHB	170.8, C	
2		57.4, CH	4.41 (d), *J* = 7.4 Hz
3		69.0,	4.13 (m)
4		43.0	2.99 (m), 3.14 (m)
1		176.9, C	
2		36.4, CH_3	2.32 (m)
3		26.4, CH_2	1.63 (m)
4-14		30.4, CH_2	1.30-1.33 (m)
15		28.2, CH_2	1.29 (m)
16		40.0, CH_2	1.17 (m)
17		28.9, CH	1.52 (m)
18/19		22.7, CH_3	0.88 (d)J=6.7 Hz

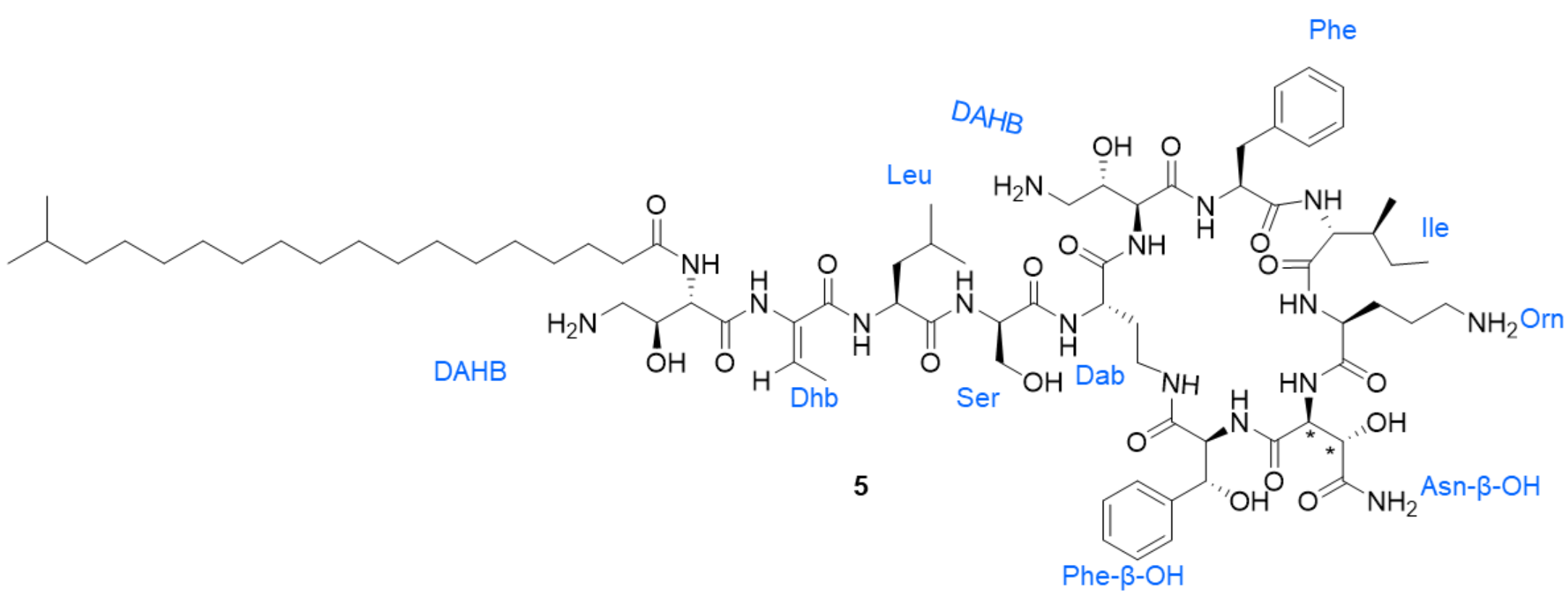

5

Table S9. NMR data for marinocyclin F (**6**) in methanol-d_4 (^{1}H 600 MHz, ^{13}C 150 MHz)

No.	Amino acid residues	^{13}C/HSQC	^{1}H NMR
1	β-OH-Phe		
2		63.2, CH	4.21 (m)
3		73.5, CH	5.04 (d), *J*=5.6 Hz
4		142.1, C	
5/9		127.5, CH	7.39(m)
6/8		129.8, CH	7.30 (m)
7		129.4, CH	7.34 (m)
1	β-OH-Asn		
2		56.7, CH	4.88 (m)
3		71.6, CH	4.57 (m)
4			
1	Orn		
2		55.1, CH	4.63 (m)
3		31.6, CH_2	1.77 (m), 1.82 (m)
4		25.0, CH_2	1.62 (m), 1.66 (m)
5		40.0 CH_2	2.93, (m)
1	Ile		
2		57.5, CH	4.25 (dd), *J*= 3.7, 8,8 Hz
3		36.1, CH	2.00 (m)
4		26.8, CH_2	0.57, (m), 0.42 (m)
5		12.3, CH_3	0.63 (t), *J*= 7.4 Hz
6		14.3	0.61 (d), *J*= 6.9 Hz
1	Phe		
2		58.2, CH	4.56 (m)
3		37.9, CH	2.95 (m), 3.14 (m)
4		136.7, C	
5/9		130.4, CH	7.23 (m)
6/8		129.9, CH	7.30 (m)
7		128.3, CH	7.24 (m)
1	DAHB		
2		56.7, CH	4.46 (d), *J* = 8.7 Hz
3		69.8, CH	4.15 (m)
4		43.5, CH_2	2.97 (m), 3.13 (m)
1	Dab		
2		53.2, CH	4.21 (m)
3		33.3, CH_2	1.75 (m), 1.90 (m)
4		36.3, CH_2	2.98 (m), 3.37 (m)
1	Ser		
2		57.4, CH	4.37 (m)

3		62.9, CH_2	3.88 (m), *J*=4.9, 8.6 Hz<br>3.90 (dd), *J*=5.2, 8.6 Hz
1	Leu		
2		53.0, CH	4.41 (m)
3		41.2, CH_2	1.70 (m)
4		25.9, CH	1.70 (m)
5		21.4, CH_3	0.92 (d), *J* = 6.0 Hz
6		23.5, CH_3	0.98 (d), *J* = 6.0 Hz
1			
2		35.7, CH_3	2.29 (m)
3		25.0, CH_2	1.62 (m)
4		23.7, CH_2	1.35 (m)
5		28.3, CH_2	1.28 (m)
6		77.0, CH	1.17 (m)
7		33.7, CH_2	1.63 (m), 1.75 (m)
8		7.7, CH_3	0.90 (d), *J*=7.6 Hz

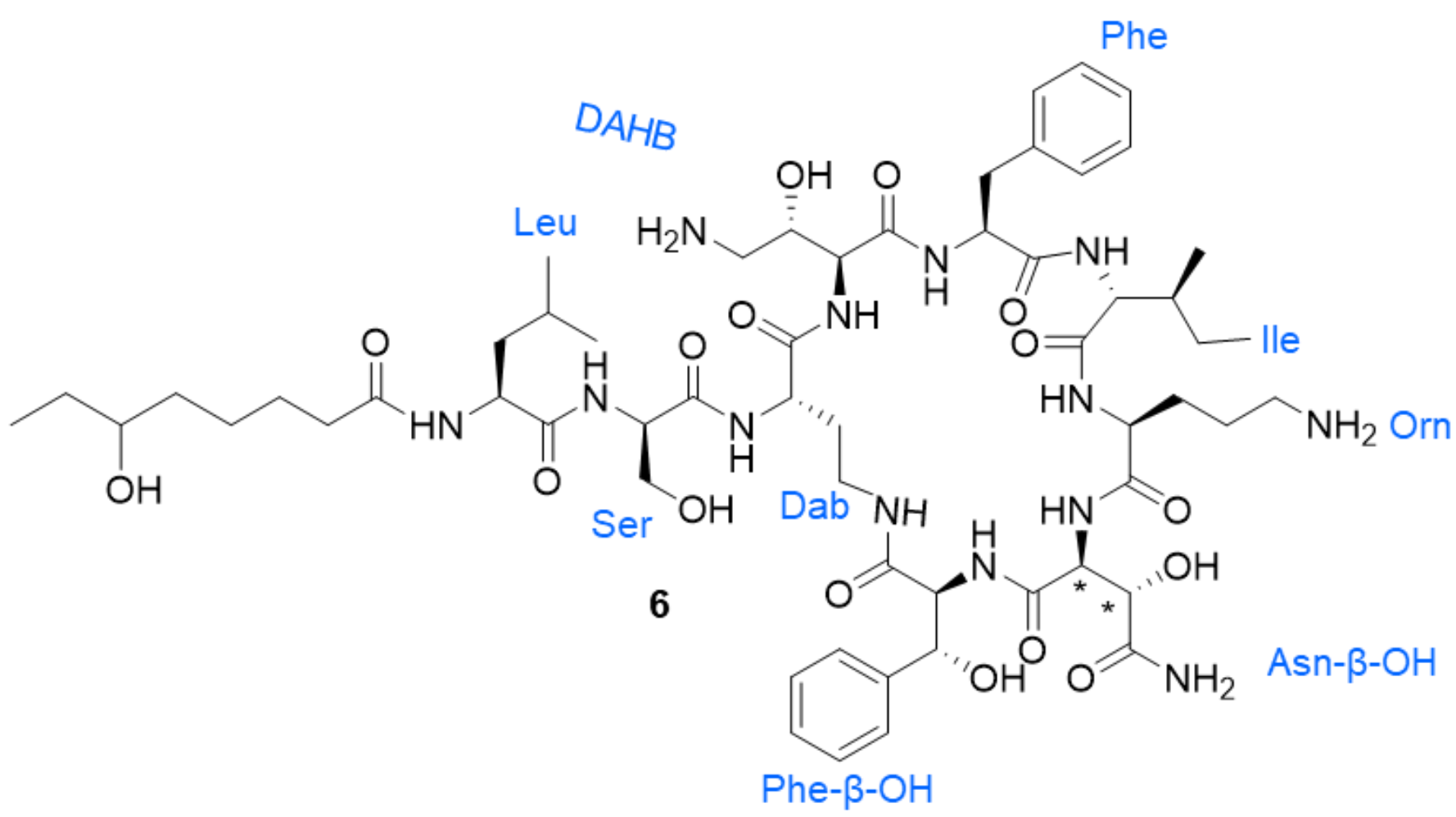

6

Table S10. MS^2 molecular network analysis of *A. megaterium* crude extract. Table entries are for identified marinocyclins as well as network nodes that suggest additional congeners.

$[M+H]^{2+}$	$[M+H]^{+}$	Isolated compound
613	1226	**Marinocyclin F**
656	1311	
662	1324	**Marinocyclin G**
670	1339	
677	1353	
684	1367	
691	1381	
698	1395	
705	1409	
712	1423	
719	1437	
726	1451	
**733**	**1465**	**Marinocyclin B**
**740**	**1479**	**Marinocyclin A**
747	1493	
754	1507	
**761**	**1521**	**Marinocyclin C**
768	1535	
775	1549	
**782**	**1563**	**Marinocyclin E**
**789**	**1577**	**Marinocyclin D**
810	1619	
831	1661	
838	1675	

Table S11. Bacterial test strains used in this study

Strain	MIC method	Reference / Comments
Bacillus subtilis 168 *trpC2*	Method B	Anagnostopoulos et al., J Bacteriol., 1961[21]
Bacillus subtilis 168 DSM10	Method A	German Collection of Microorganisms and Cell Cultures (DSMZ)
Bacillus subtilis BAU-102	Method B	Ulijasz et al., J Bacteriol., 1996[22]
Staphylococcus aureus ATCC 29213	Method B	American Type Culture Collection (ATCC), CLSI reference strain
Staphylococcus aureus USA300	Method B	Diep et al., Lancet, 2006[23]
Staphylococcus aureus SA113 wt	Method B	Weidenmaier et al., J Infect Dis., 2005[24]
Staphylococcus aureus SA113 Δ*dltA*	Method B	Weidenmaier et al., J Infect Dis., 2005[24]
Staphylococcus aureus SA113 Δ*tagO*	Method B	Weidenmaier et al., J Infect Dis., 2005[24]
Staphylococcus aureus SA113 Δ*mprF*	Method B	Ernst et al., mBio, 2018[25]
Staphylococcus epidermidis ATCC 12228	Method A	American Type Culture Collection (ATCC)
Enterococcus faecium BM 4147-1	Method B	Woodford et al., Antimicrob Agents Chemother, 1998[26]
Enterococcus faecium 6011	Method B	Werner et al., FEMS Microbiol Lett., 1997[27]
Escherichia coli ATCC 25922	Method B	American Type Culture Collection (ATCC), CLSI reference strain
Escherichia coli Tü21-10	Method B	University Hospital Tübingen, clinical isolate, 3MRGN*
Escherichia coli Tü21-13	Method B	University Hospital Tübingen, clinical isolate, 3MRGN*
Escherichia coli Tü21-32	Method B	University Hospital Tübingen, clinical isolate, 4MRGN*
Escherichia coli Tü21-34	Method B	University Hospital Tübingen, clinical isolate, 4MRGN*
Escherichia coli J53	Method B	Tietgen et al., Int J Antimicrob Agents, 2019[28]
Escherichia coli J53 pTOPO	Method B	Tietgen et al., Int J Antimicrob Agents, 2019[28]
Escherichia coli J53 pTOPO_mcr-1_prom	Method B	Tietgen et al., Int J Antimicrob Agents, 2019[28]

Escherichia coli J53 TC pKP2442	Method B	Tietgen et al., Int J Antimicrob Agents, 2019[28]
Escherichia coli BW 25113 wt	Method B	Baba et al., Mol Syst Biol., 2006[29]
Escherichia coli BW 25113 ∆*tolC*	Method B	Baba et al., Mol Syst Biol., 2006[29]
Escherichia coli BW 25113 ∆*acrA*	Method B	Baba et al., Mol Syst Biol., 2006[29]
Escherichia coli BW 25113 ∆*acrB*	Method B	Baba et al., Mol Syst Biol., 2006[29]
Escherichia coli DSM 22654	Method A	DSMZ
Escherichia coli DSM 10003	Method A	DSMZ
Escherichia coli 700455	Method A	Torres et al.~~,~~ *BMC Microbiol*., 2021[30]
Escherichia coli 721296	Method A	Torres et al.~~,~~ *BMC Microbiol*., 2021[30]
Escherichia coli 705963	Method A	Torres et al., *BMC Microbiol*., 2021[30]
Escherichia coli 709006	Method A	Torres et al.~~,~~ *BMC Microbiol*., 2021[30]
Salmonella enterica stm BS300 DSM 17058	Method A	DSMZ
Klebsiella pneumoniae ATCC 12657	Method B	ATCC, reference strain
Klebsiella pneumoniae ATCC 27799	Method B	ATCC, reference strain
Klebsiella pneumoniae KP2442	Method B	Tietgen et al., Int J Antimicrob Agents, 2019[28]
Klebsiella pneumoniae DSM 25721	Method A	DSMZ
Enterobacter aerogenes ATCC 13048	Method B	ATCC, reference strain
Enterobacter aerogenes UR9466	Method B	University Hospital Tübingen, clinical isolate
Pantoea agglomerans DSM 3493	Method A	DSMZ
Pseudomonas aeruginosa ATCC 27853	Method B	ATCC, CLSI reference strain
Pseudomonas aeruginosa B63230	Method B	Henrichfriese et al., Antimicrob Agents Chemother, 2005[31]
Pseudomonas aeruginosa DSM 24599	Method A	DSMZ

Pseudomonas aeruginosa DSM 1117	Method A	DSMZ
Pseudomonas stutzeri LMG 5383	Method A	Belgian Co-ordinated Collections of Microorganisms
Acinetobacter baumannii 09987	Method B	University Hospital Bonn, clinical isolate
Acinetobacter baumannii I-7486	Method B	University Hospital Bonn, clinical isolate
Acinetobacter baumannii DSM 30007	Method A	DSMZ

Table S12. Minimal Inhibitory Concentrations (MIC) of marinocyclin A (**1**), B (**2**), and D (**4**) for a panel of bacteria by Method A. MIC tests were conducted in 96-well microtiter plates in NB medium in a final volume of 200 µL and inoculated with bacterial cultures adjusted to an OD_{600} of 0.04. The plates were incubated at 37 °C for 24 hours. The lowest concentration of the drug preventing visible growth of the pathogen was taken as the MIC.

**Test organisms**	**MIC (µM)**			
	**1**	**2**	**4**	**Kanamycin (µg/mL)**
Acinetobacter baumannii DSM 30007	3	2.4	2.8	<0.04
Pseudomonas aeruginosa DSM 24599	12.7	12.0	11.9	<0.04
Pseudomonas aeruginosa DSM 1117	5.3	6.7	8.9	<0.04
Pseudomonas stutzeri LMG 5383	15.2	9.6	14.2	<0.04
Klebsiella pneumoniae DSM 25721	7.6	9.6	7.1	<0.04
Escherichia coli BW 25113	5.3	4.8	5.0	<0.04
Escherichia coli DSM 22654	5.3	4.8	5.0	<0.04
Escherichia coli DSM 10003	7.1	9.1	6.7	<0.04
Pantoea agglomerans DSM 3493	5.3	6.7	5.0	<0.04
Salmonella enterica stm BS300	5.3	6.7	5.0	<0.04
Bacillus subtilis 168 DSM 10	7.6	4.8	7.1	<0.04
Staphylococcus epidermidis	3.0	2.4	2.8	<0.04

Table 13. Minimal Inhibitory Concentrations (MIC) of marinocyclins A (**1**), B (**2**), and D (**4**) for a panel of polymyxin-resistant *E. coli* clinical isolates with mutations in *pmrB* and *pmrC*.[29] Chromosomal mutations in the sensor kinase PmrB lead to increased LPS modification. MIC tests were conducted in 96-well microtiter plates in NB medium by Method A.

Test organisms.	Mutations	MIC (µg/mL)			
		**1**	**2**	**4**	**Polymyxin B**
Escherichia coli 700455	*pmrB* (L197D)	25	12.5	25	6.3
Escherichia coli 721296	*pmrC* (C27Y) & *pmrB* (P97L)	25	25	25	12.5
Escherichia coli 705963	*pmrB* (P97A)	12.5	12.5	25	25
Escherichia coli 709006	*pmrB* (E169K)	25	25	25	6.3

Table S114. Minimal Inhibitory Concentrations (MIC) for efflux pump mutants in *E. coli* and surface charge mutants in *S. aureus*. The activity of marinocyclin A is not affected by the primary efflux pump of *E. coli* or the surface charge of *S. aureus*. MICs (µg/mL) of marinocyclin A for *E. coli* BW25113 wildtype *versus* isogenic AcrAB–TolC efflux pump mutants, and *S. aureus* SA113 wildtype *versus* isogenic cell-envelope–polarity mutants. DltA (D-alanyl-D-alanine carrier protein ligase)-deficient *S. aureus* lacks D-alanylation of lipoteichoic acids and teichoic acids, resulting in a higher negative surface charge. Also, *S. aureus* Δ*mprF* (Lys-PG synthase/flippase) has an increased negative surface charge, as less of the negatively charged phosphatidylglycerol is converted to the zwitterionic lysyl-PG. TagO-deficient *S. aureus* lacks the first enzyme in teichoic acid biosynthesis (*N*-glucosamine-1-phosphate-transferase), and the absence of teichoic acids reduces the negative surface charge. Altered surface charge only minimally affected the susceptibility of the *S. aureus* to marinocyclin A, with slightly increased activity against the more negatively charged Δ*dltA* mutant. Notably, MprF is a daptomycin resistance factor, and *S. aureus* Δ*mprF* is tenfold more sensitive to daptomycin than the wildtype,[32] but the activity of **1** was unaffected. Values represent ranges across ≥3 replicates.

Strains	MIC (µg/mL)
Escherichia coli BW 25113 wt	4
Escherichia coli BW 25113 Δ*tolC*	4
Escherichia coli BW 25113 Δ*acrA*	4
Escherichia coli BW 25113 Δ*acrB*	4
Staphylococcus aureus SA113 wt	4
Staphylococcus aureus SA113 Δ*dltA*	2 - 4
Staphylococcus aureus SA113 Δ*tagO*	4
Staphylococcus aureus SA113 Δ*mprF*	4

Supplementary Figures

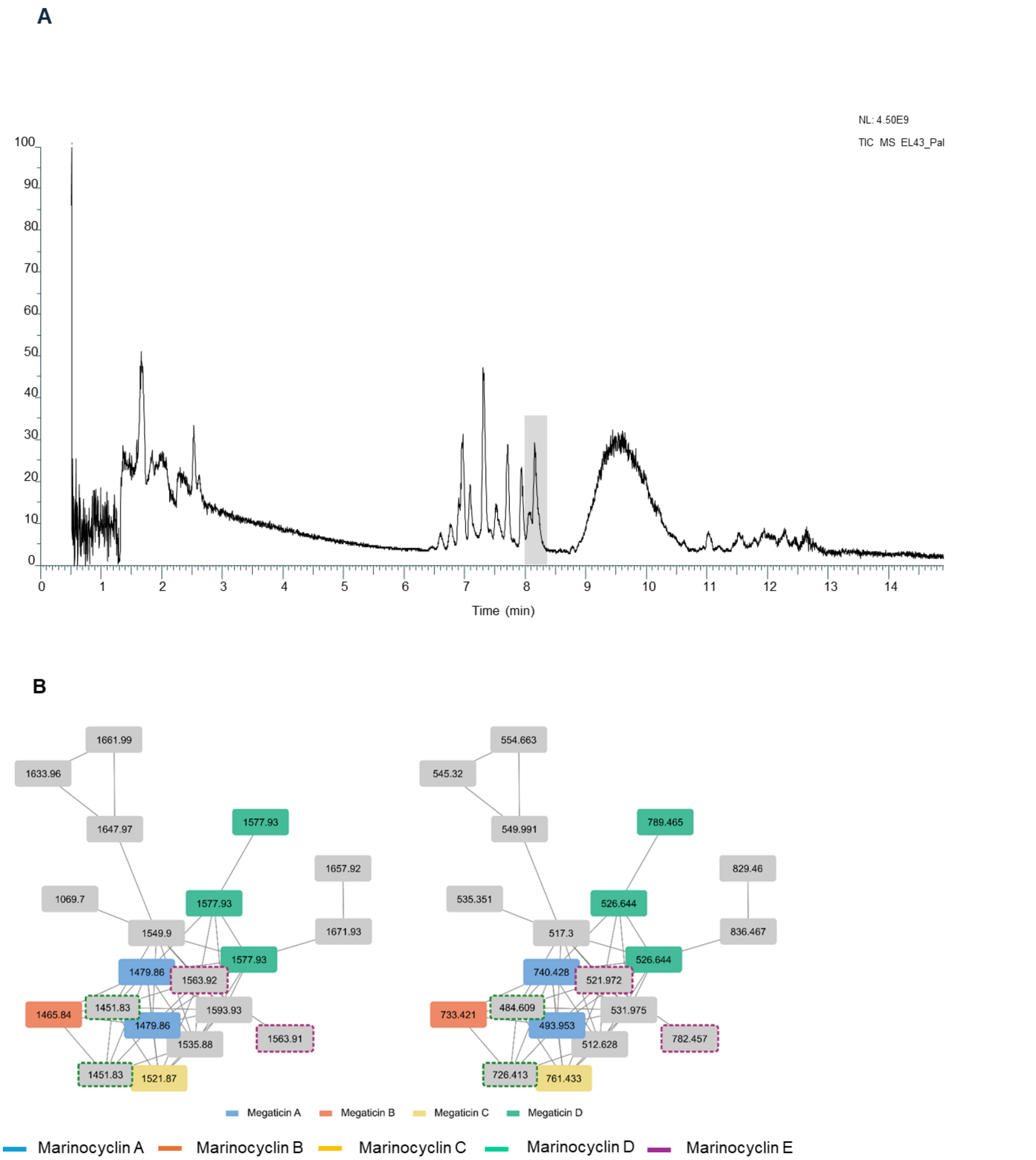

Figure S1. Total ion chromatogram (TIC) and MS^2 molecular network analysis of the pellet-derived extract of *A. megaterium* EL43 cultivated in marine broth. A) Total ion chromatogram (TIC) of pellet-derived extract of *A. megaterium* EL43 cultivated in marine broth. Marinocyclins are peaks are highlighted in grey. B) MS^2 molecular network analysis of the pellet-derived extract of *A. megaterium* EL43 cultivated in marine broth.

A molecular network constructed from HPLC-HESI-HRMS/MS data (left), precursor ions belonging to the target peptide family (right). Colored nodes: ions of the isolated peptides

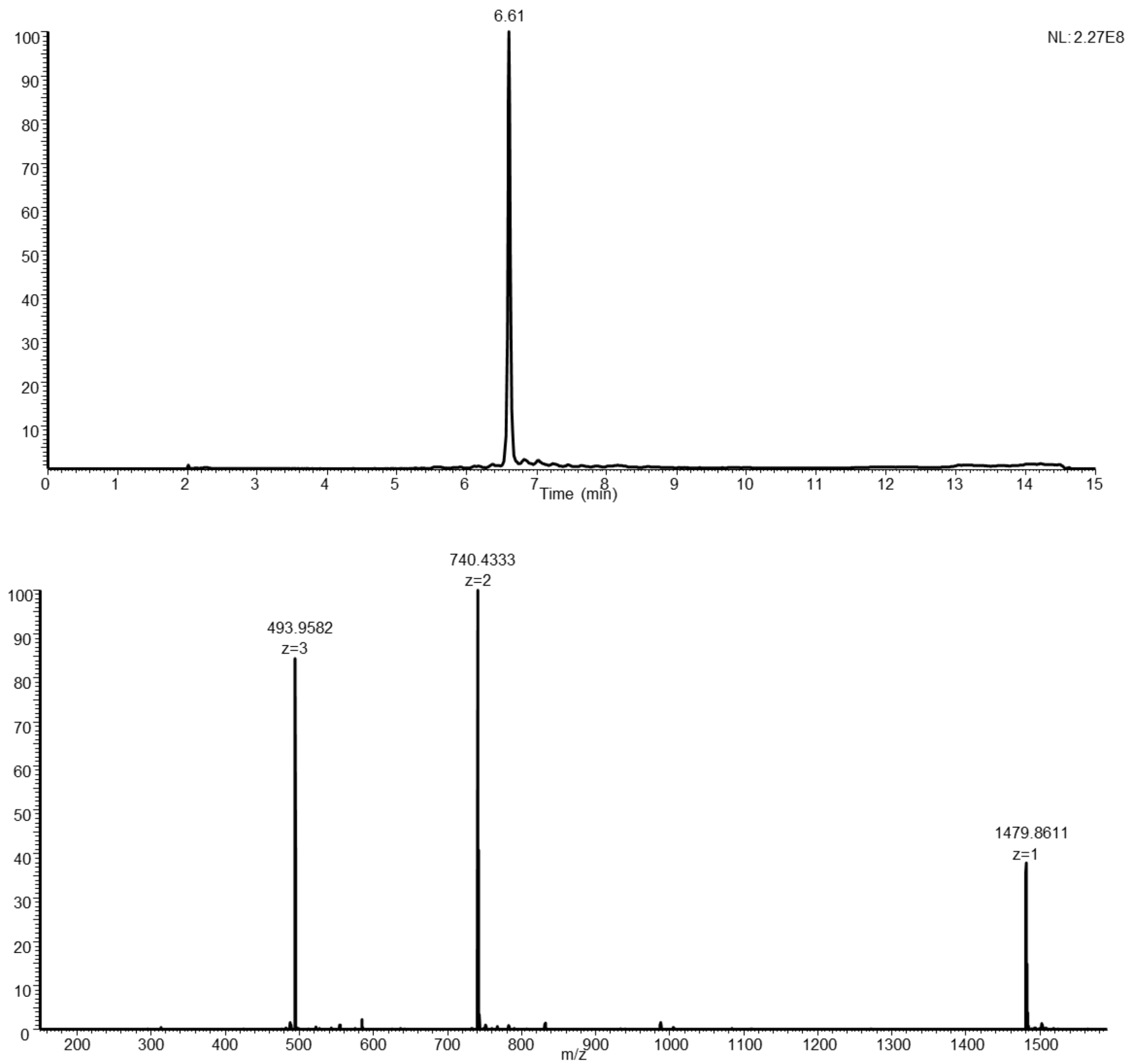

Figure S2. HPLC-HESI-HRMS data of marinocyclin A (**1**). Top: extracted ion chromatogram *m/z* 1479.8611, bottom: MS data of marinocyclin A (**1**) (*m/z* 1479.8611, $[M+H]^+$).

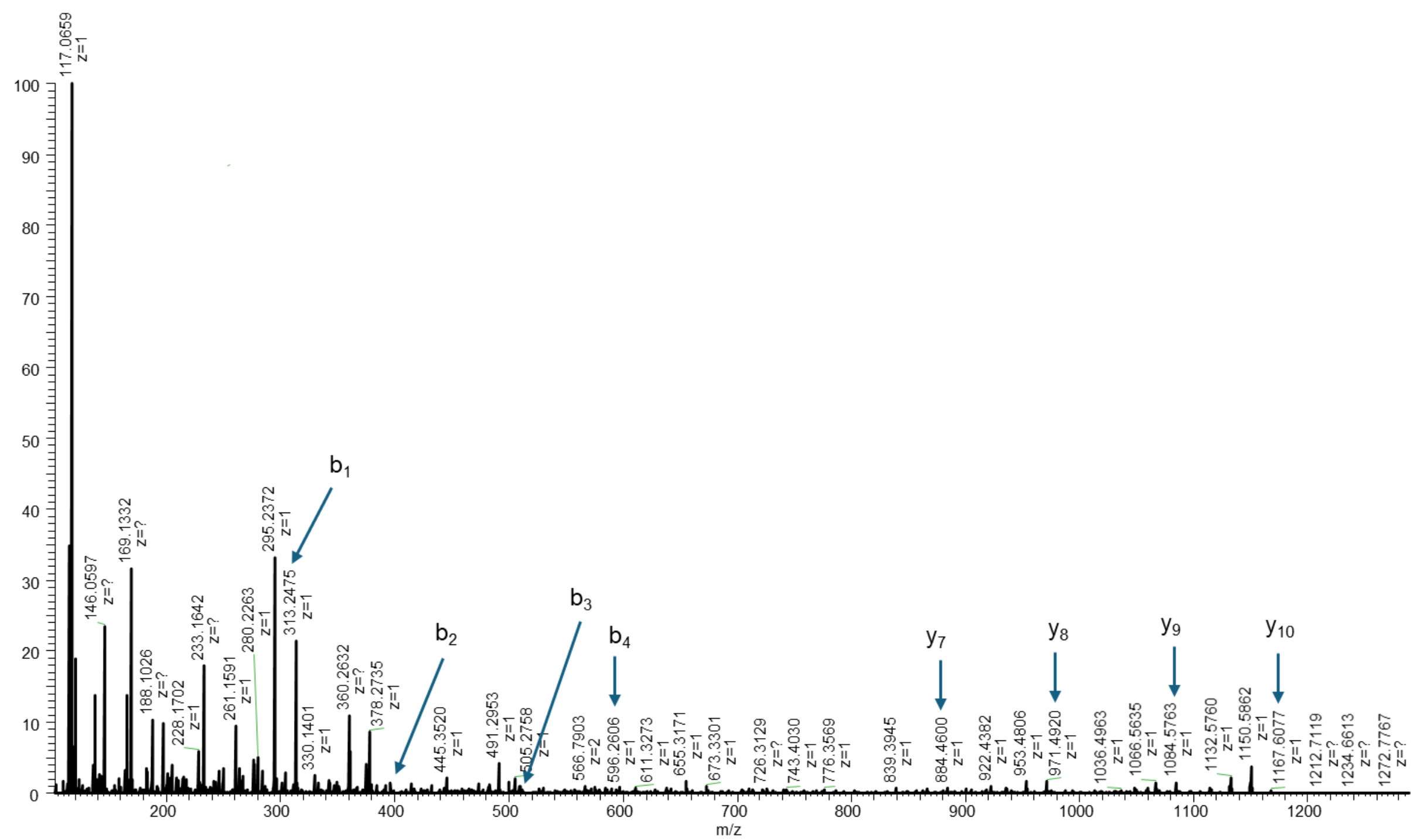

Figure S3. HESI-HRMS MS²data of marinocyclin A (**1**) (*m/z* 740.4315 $[M+H]^{2+}$).

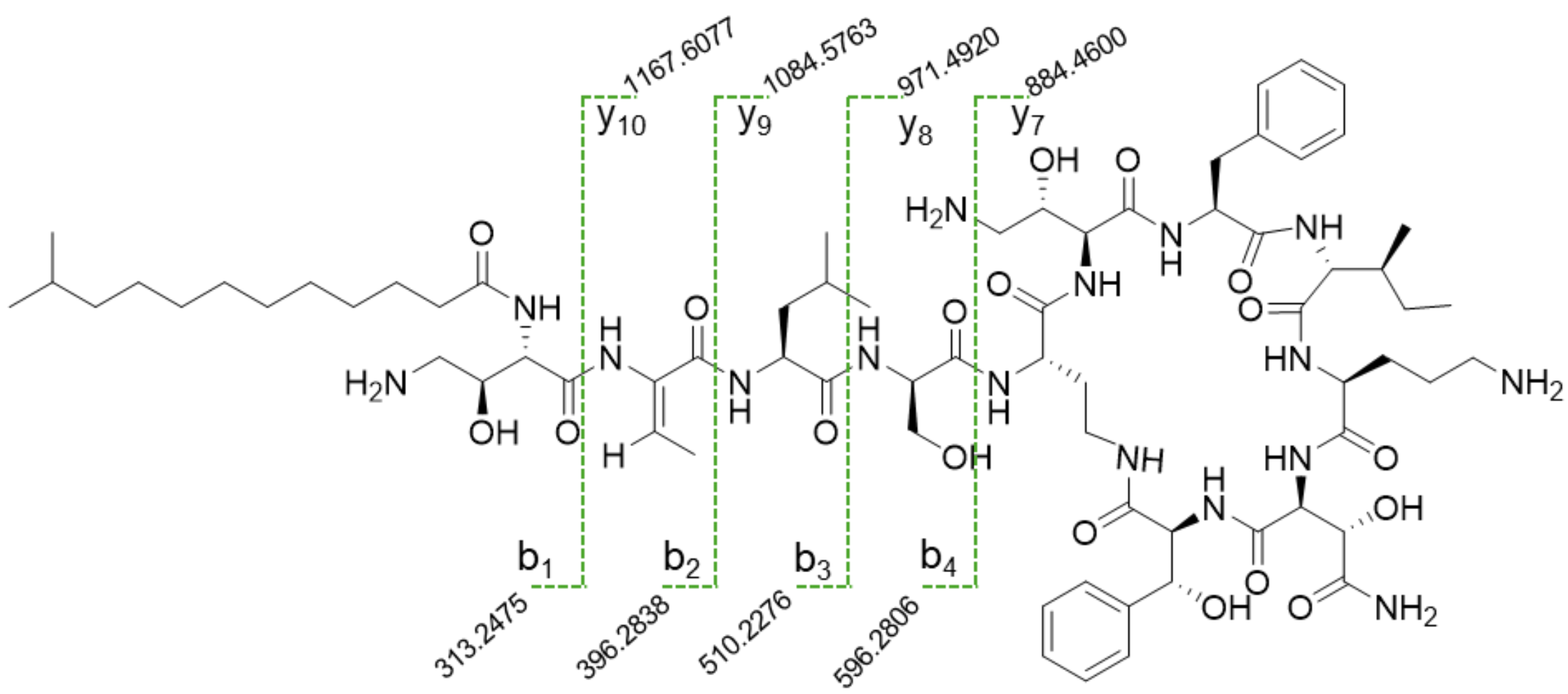

Figure S4. MS² fragmentation of marinocyclin A (**1**).

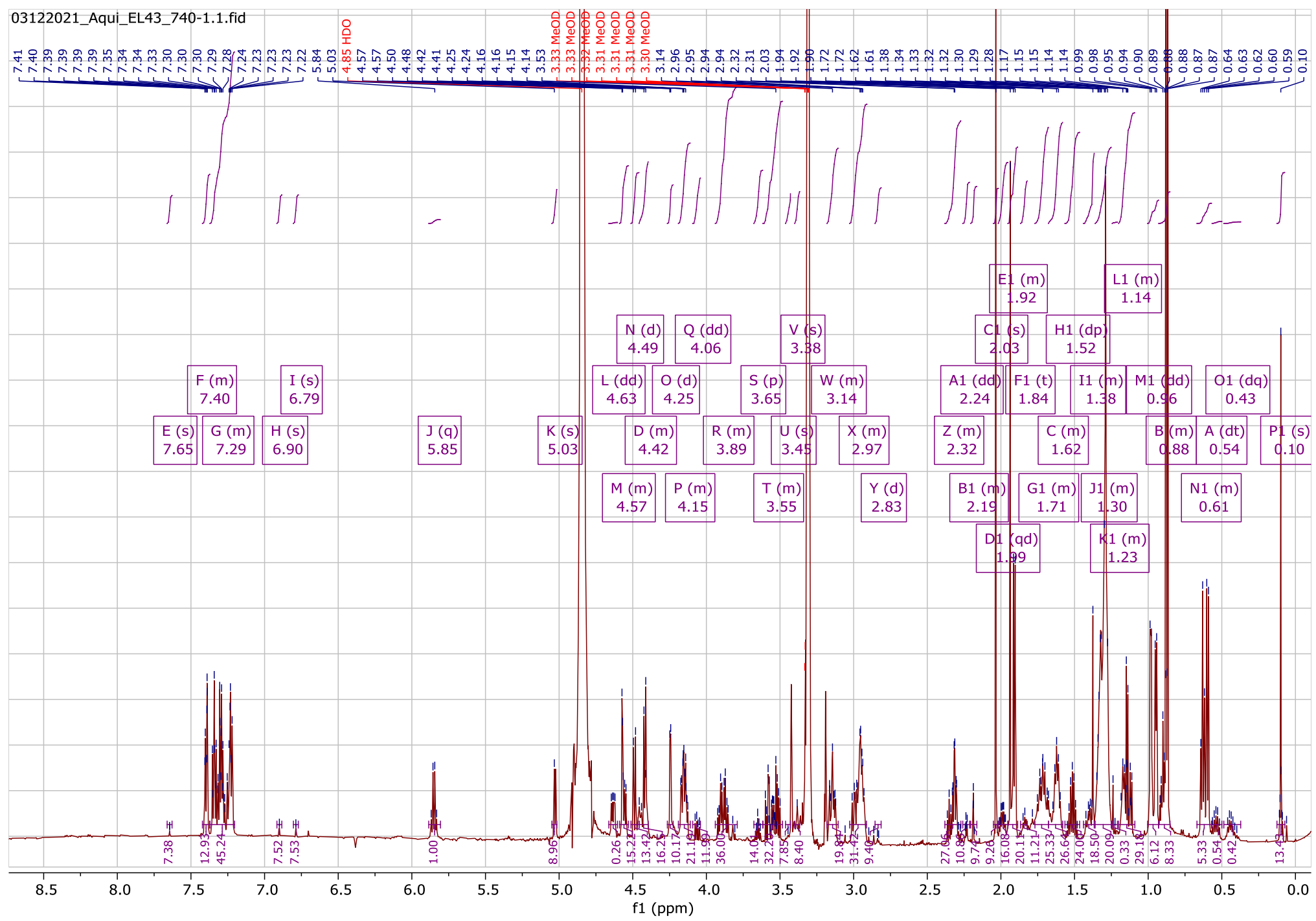

Figure S5. ^{1}H NMR spectrum of marinocyclin A (**1**) in methanol-d_4 (^{1}H 600 MHz, ^{13}C 150 MHz)

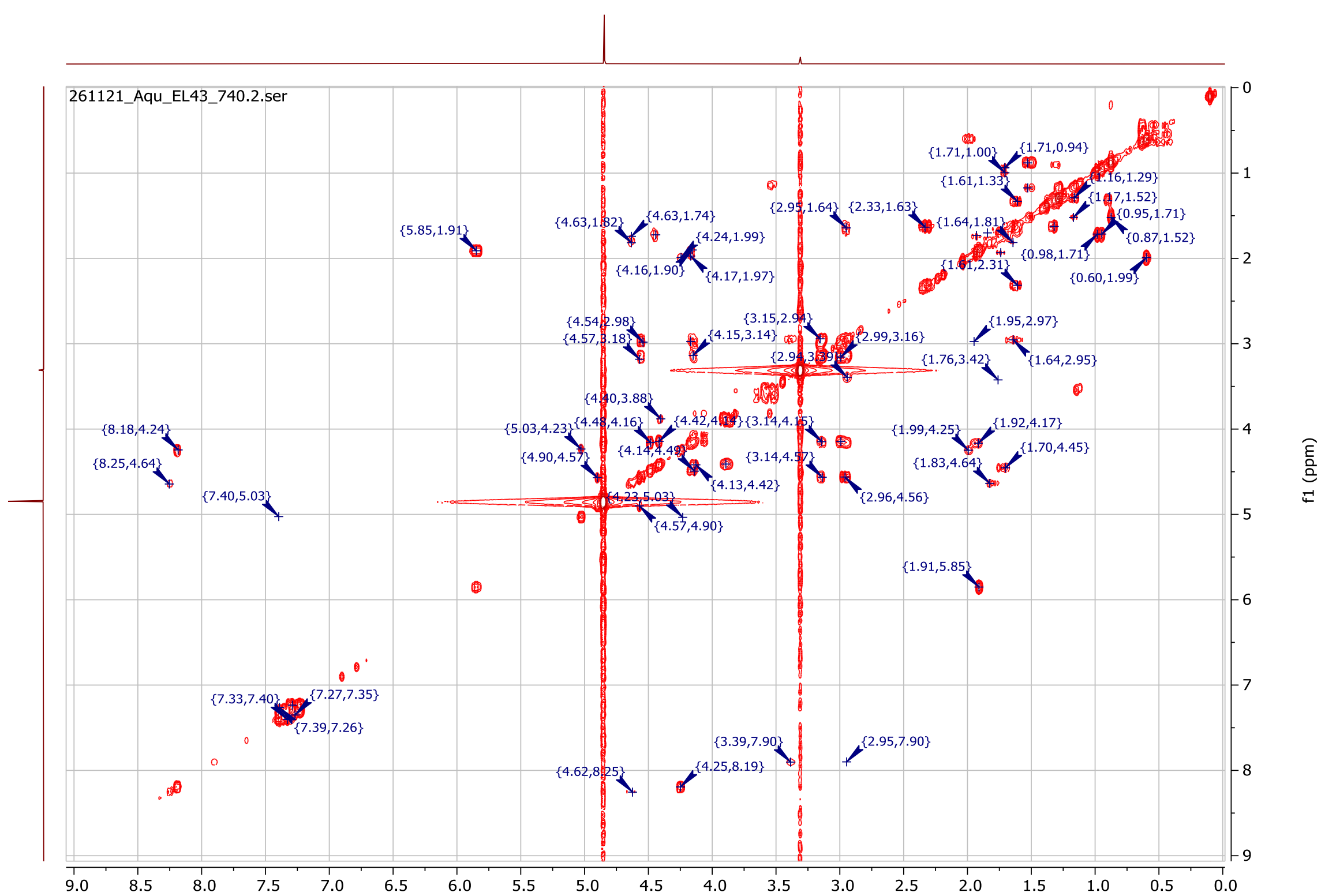

Figure S6. COSY spectrum of marinocyclin A (**1**) in methanol-d_4 (^{1}H 600 MHz).

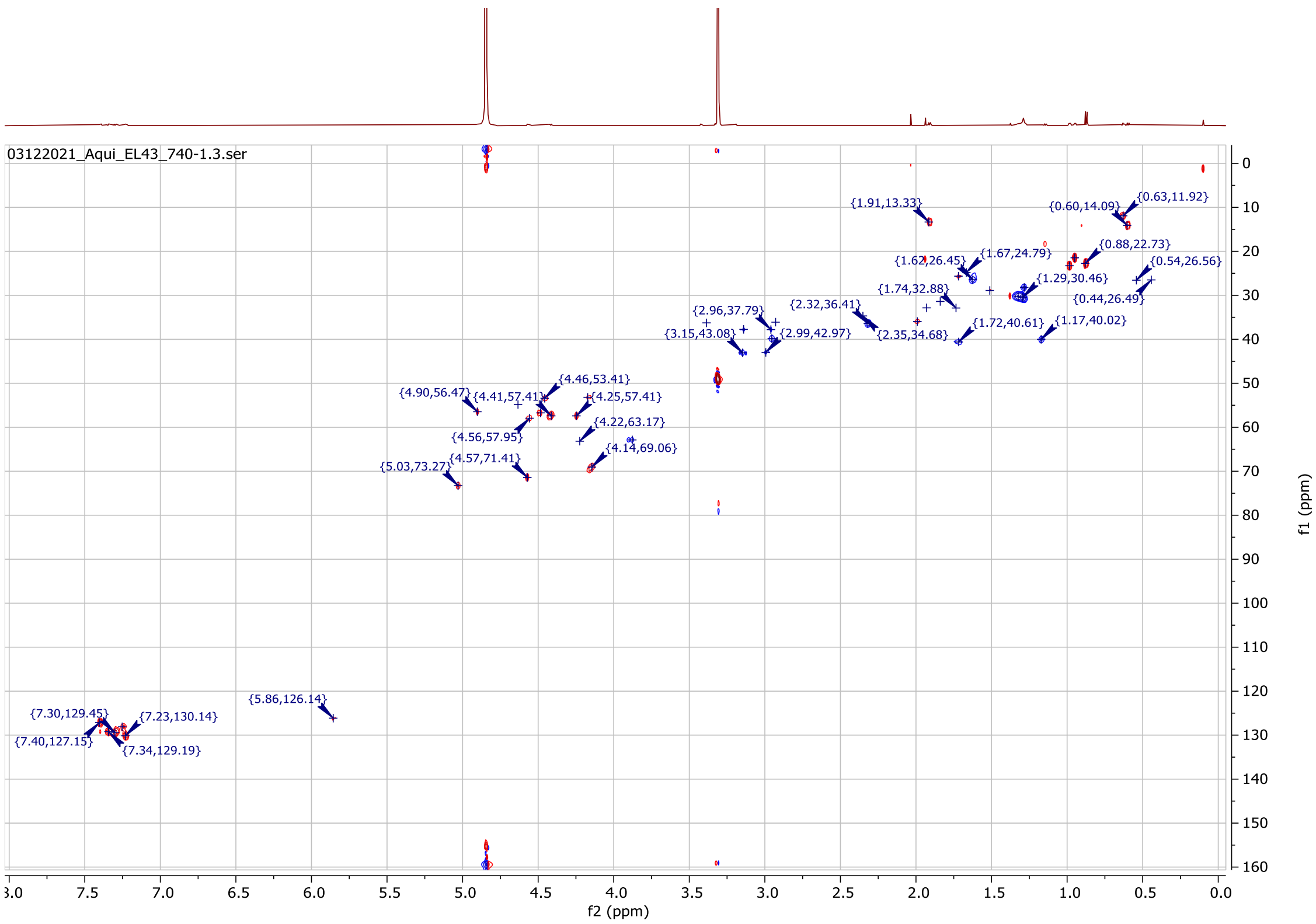

Figure S7. HSQC spectrum of marinocyclin A (**1**) in methanol-d4 (^{1}H 600 MHz, ^{13}C 150 MHz)

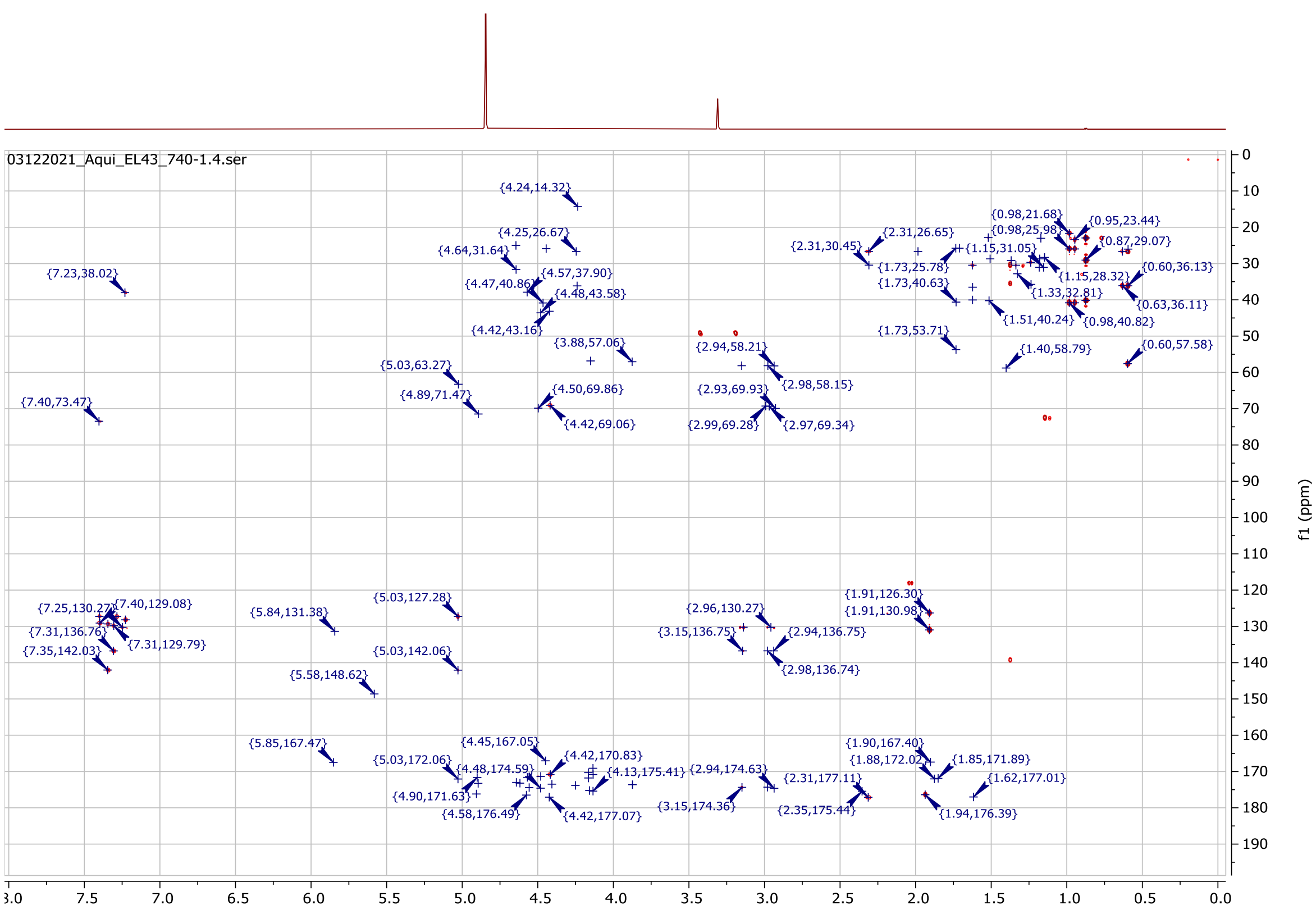

Figure S8. HMBC spectrum of marinocyclin A (**1**) in methanol-d4 (^{1}H 600 MHz, ^{13}C 150 MHz).

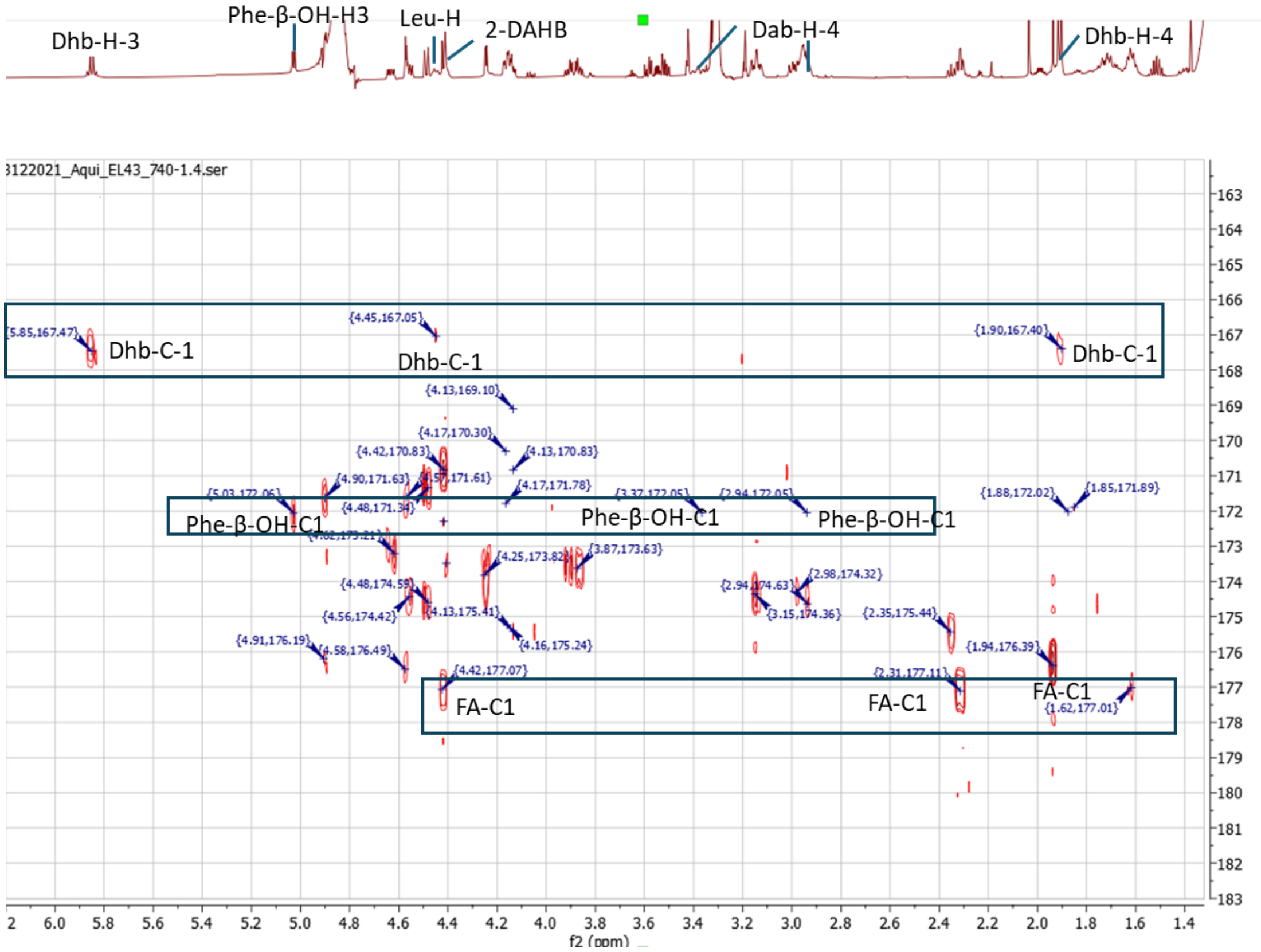

Figure S9. Zoomed HMBC spectrum of marinocyclin A (1) in methanol-d_4 (1H 600 MHz, 13C 150 MHz).

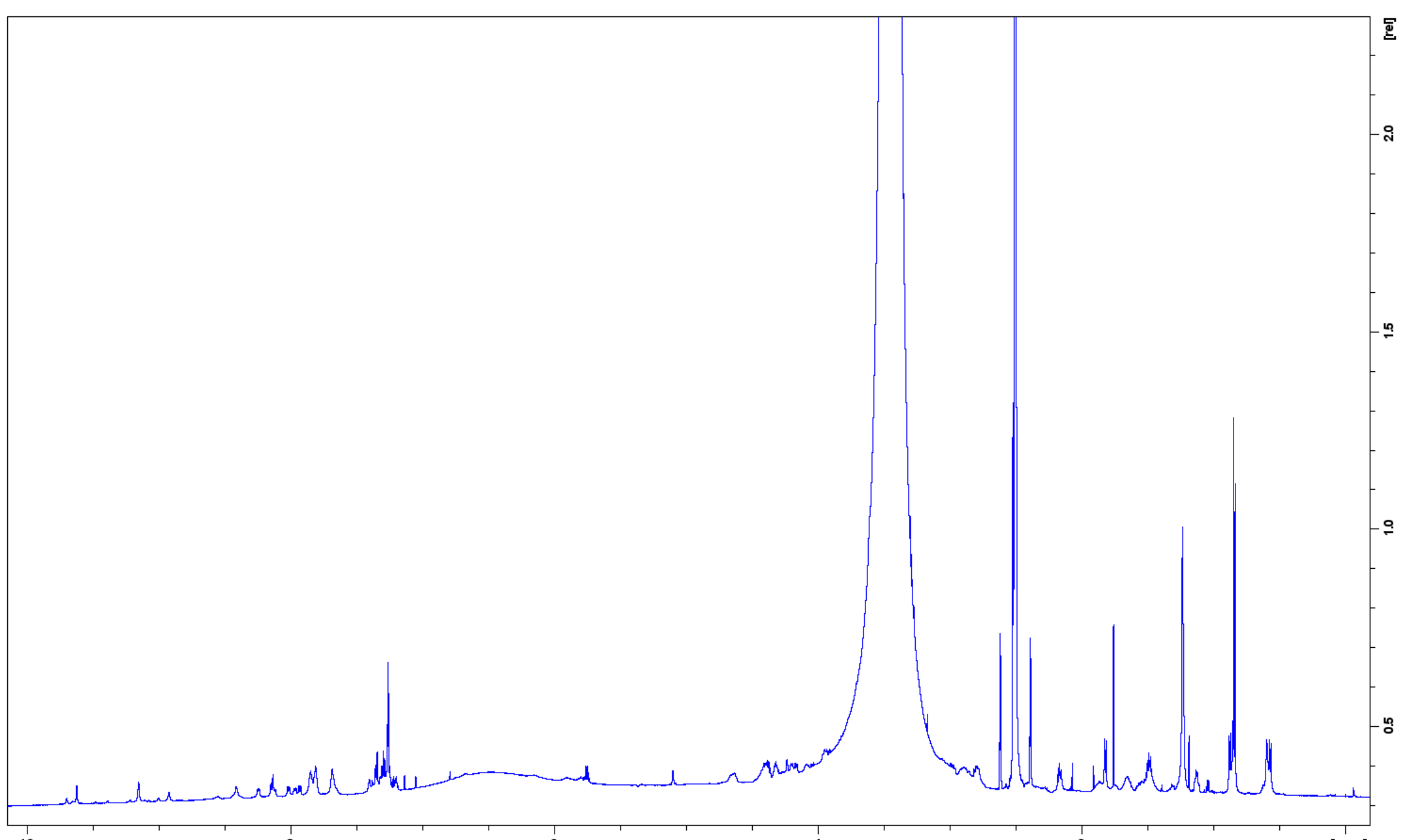

Figure S10. ^{1}H NMR spectrum of marinocyclin A (**1**) in DMSO-d_6 (^{1}H 600 MHz, ^{13}C 150 MHz).

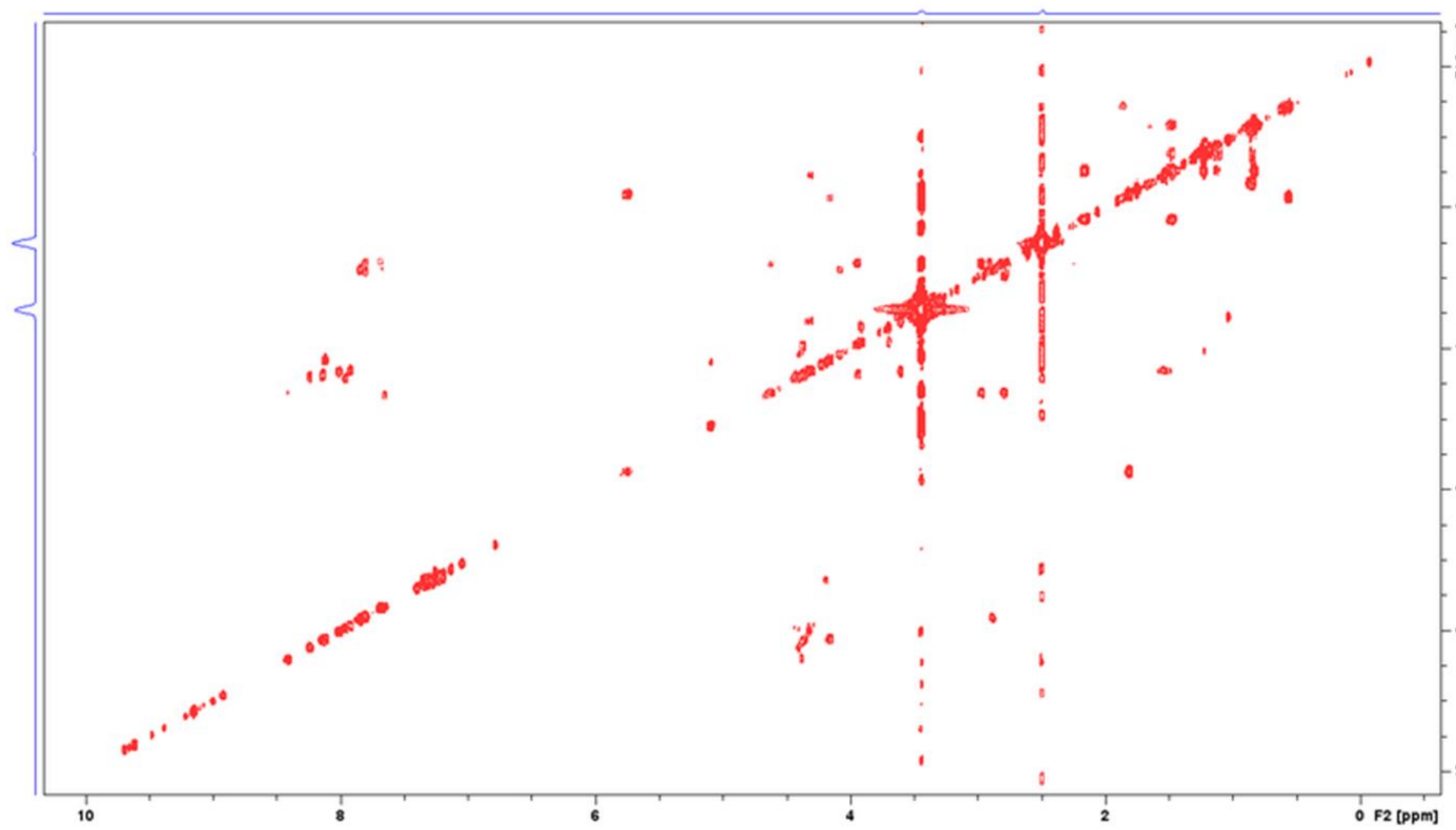

Figure S11. COSY spectrum of marinocyclin A (**1**) in DMSO-d6 (^{1}H 600 MHz).

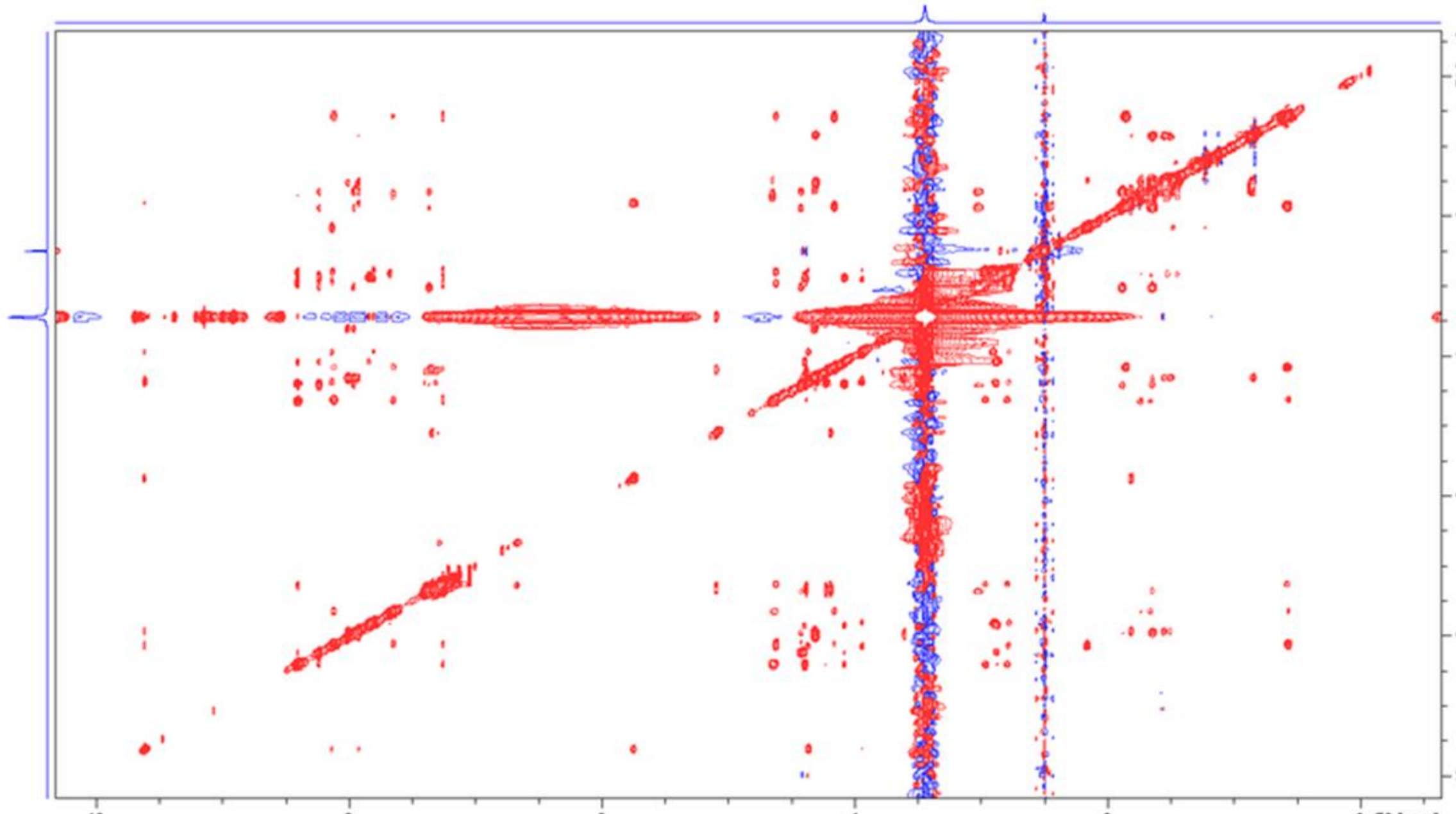

Figure S12. NOESY spectrum of marinocyclin A (**1**) in DMSO-d6 (^{1}H 600 MHz).

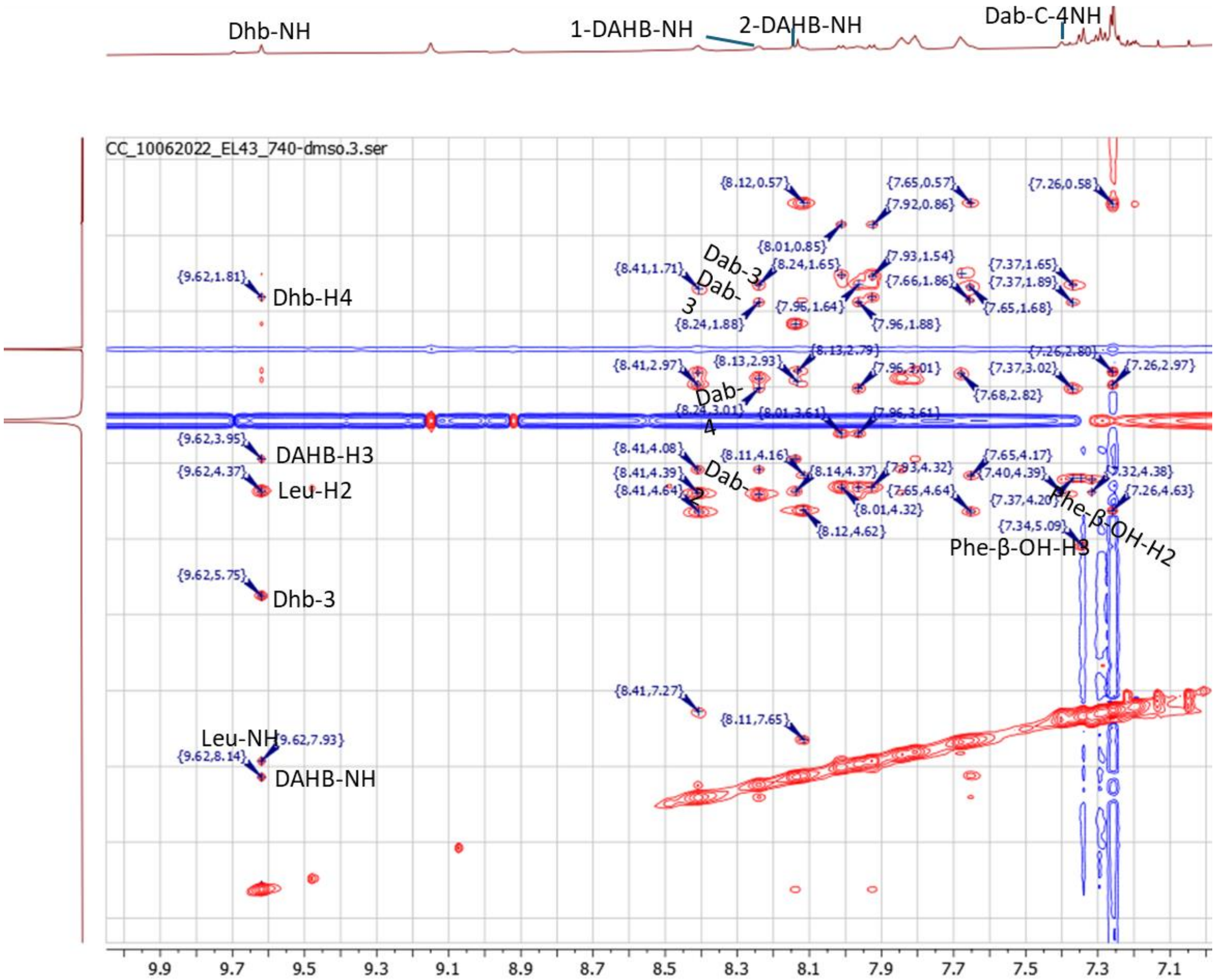

Figure S13. Zoomed NOESY spectrum of marinocyclin A (1) in DMSO-d6 (1H 600 MHz).

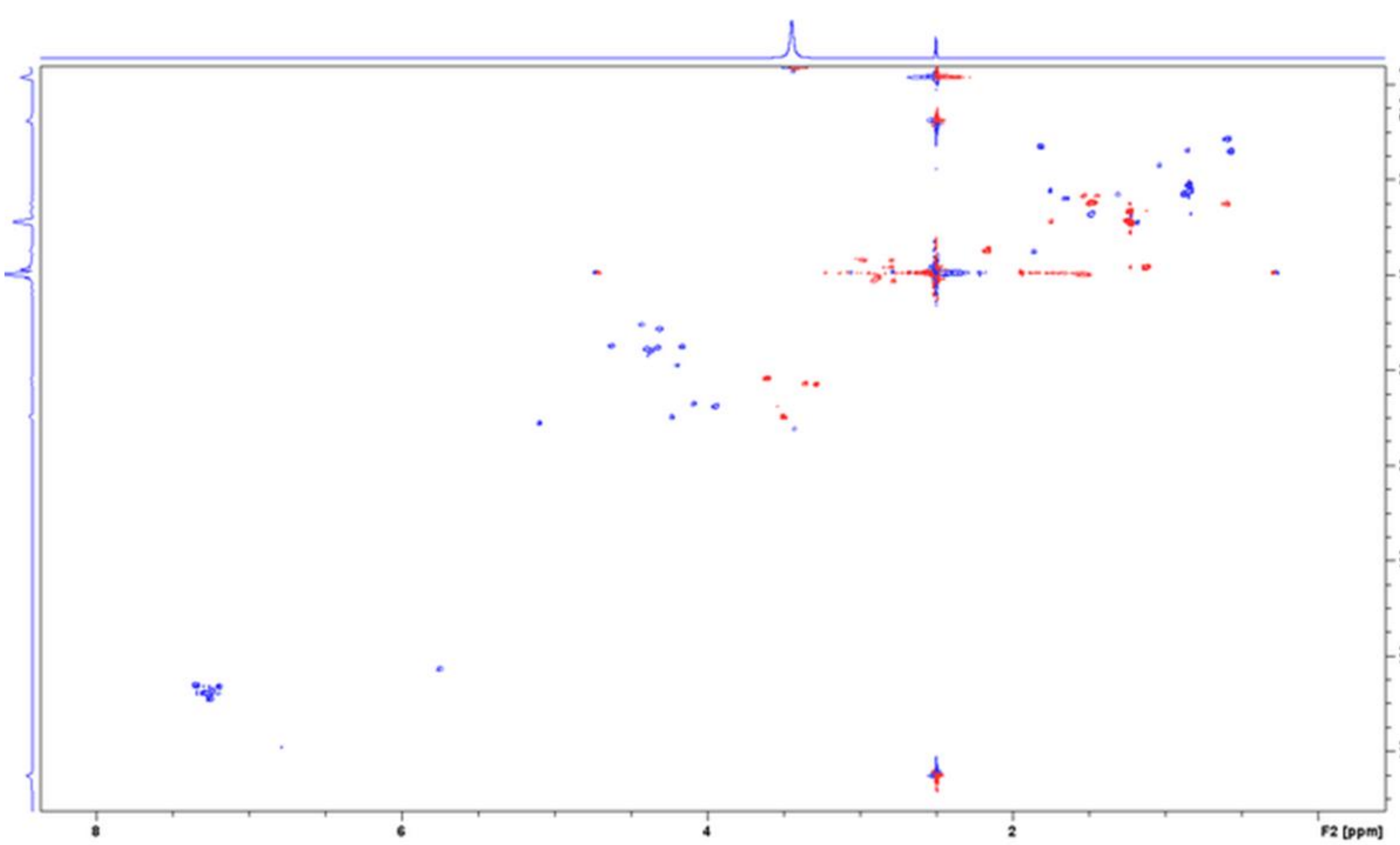

Figure S14. HSQC spectrum of marinocyclin A (**1**) in DMSO-d6 (^{1}H 600 MHz, ^{13}C 150 MHz).

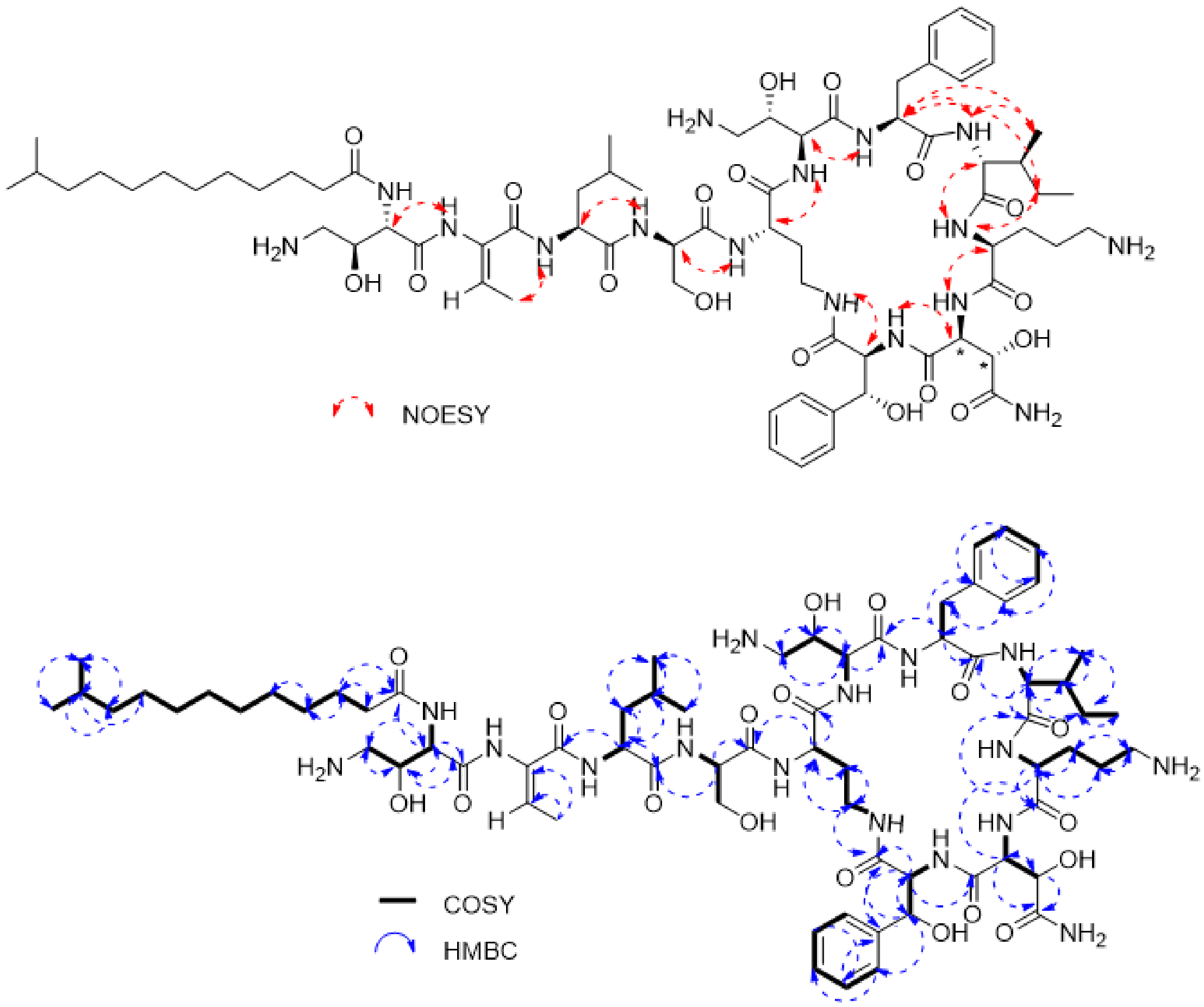

Figure S15. Structure elucidation of marinocyclin A (**1**). Top: NOESY correlations of **1**, bottom: COSY and HMBC correlations of **1**.

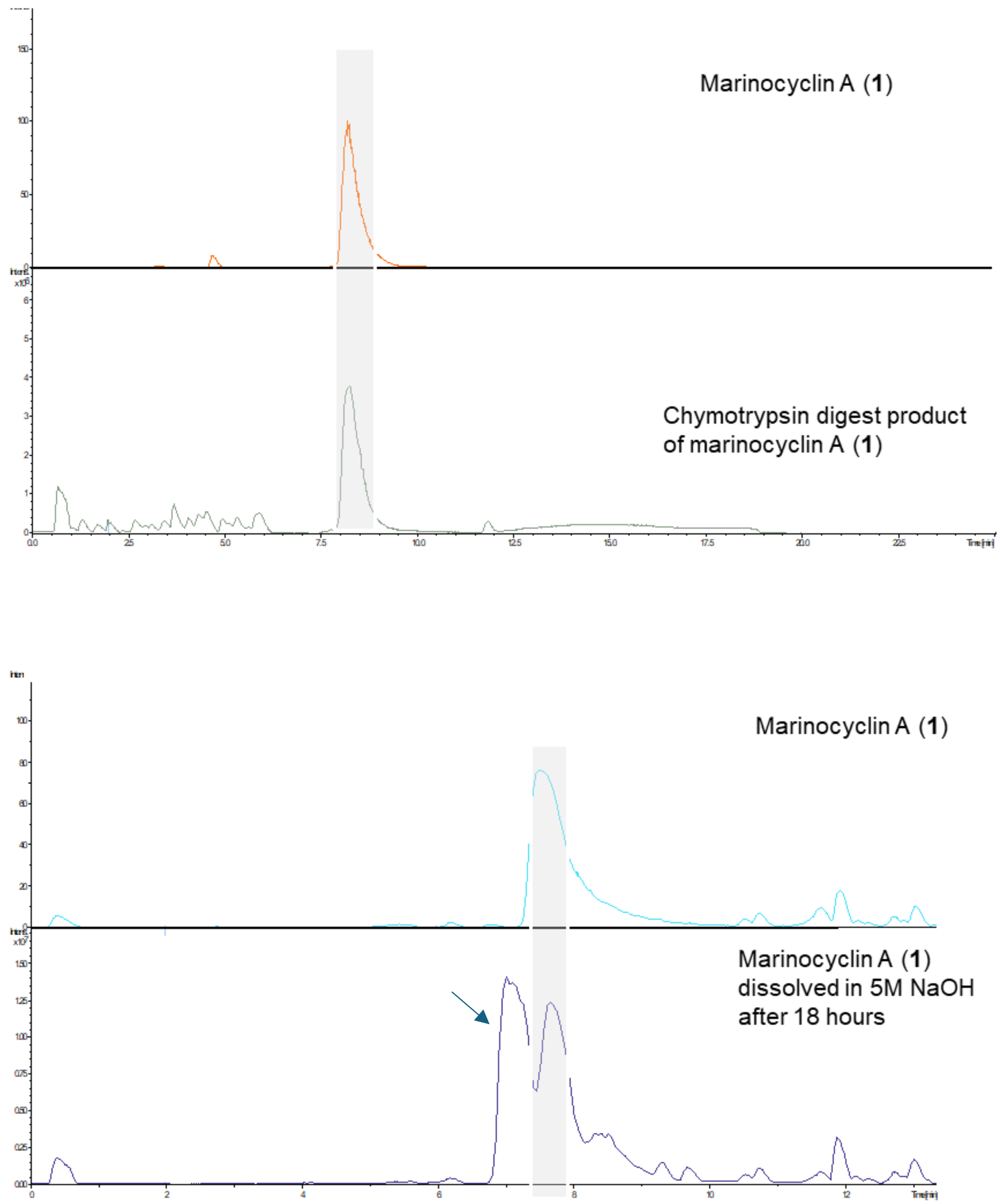

Figure S16. HPLC-HESI-HRMS data of marinocyclin A (**1**), product of **1** after chymotrypsin digest and hydrolysis of **1** with NaOH. Top: Total ion chromatogram of marinocyclin A (**1**), product of **1** after chymotrypsin digest. The cyclic ring in **1** remains intact after chymotrypsin at 25 °C for 18 hours. Bottom: total ion chromatogram of marinocyclin A (**1**) incubated in 5 M NaOH at room temperature for 18 hours. The cyclic ring in **1** remains intact after 18 hours of incubation at room temperature, and over 50% of **1** was isomerized (peak indicated by arrow).

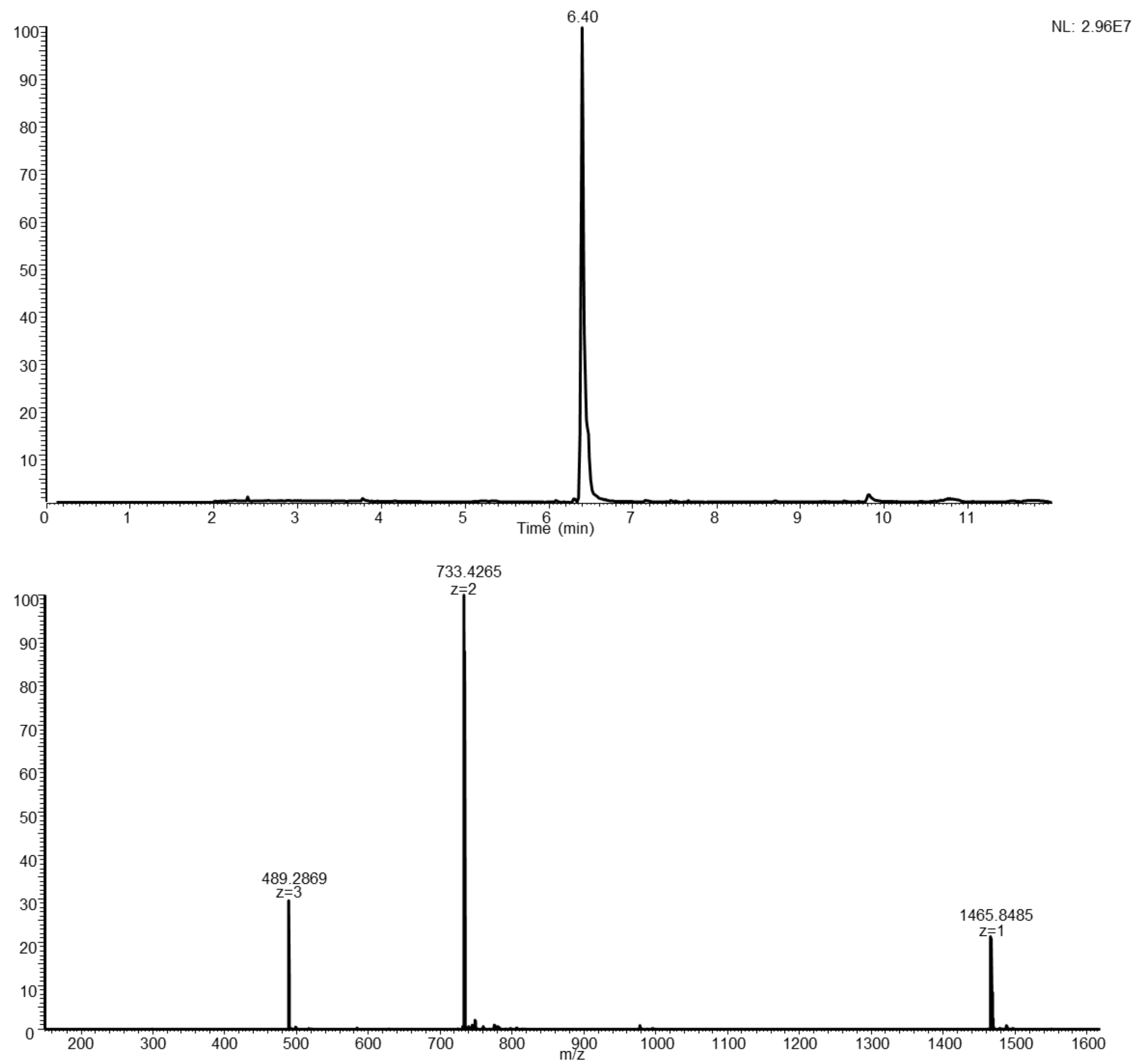

Figure S17. HPLC-HESI-HRMS data of marinocyclin B (**2**). Top: extracted ion chromatogram *m/z* 1465.8485, bottom: MS data of marinocyclin B (**2**) (*m/z* 1465.8485 $[M+H]^+$).

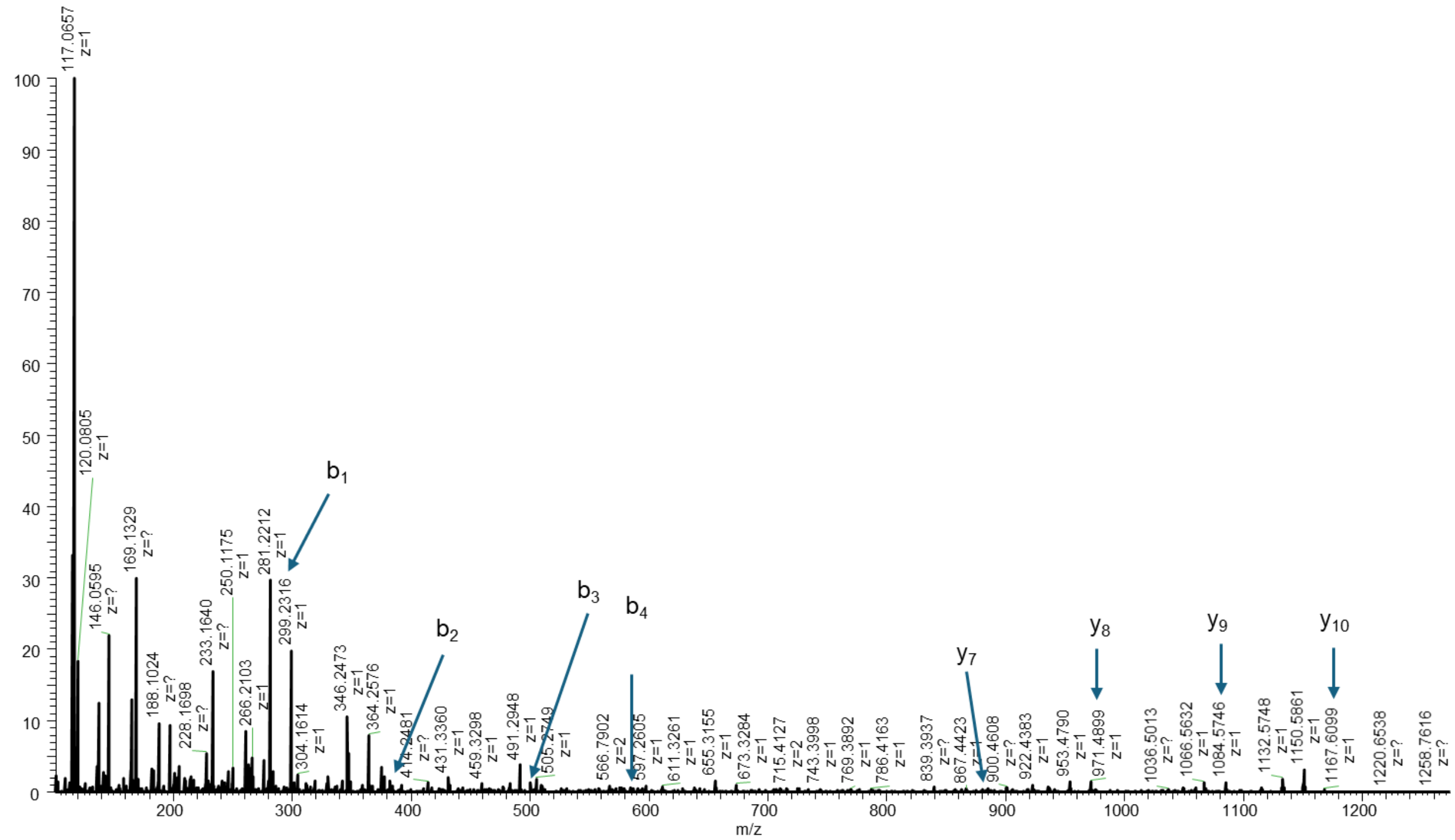

Figure S18. HESI-HRMS[2] data of marinocyclin B (**2**) (*m/z* 733.4232 [M+H][2+]).

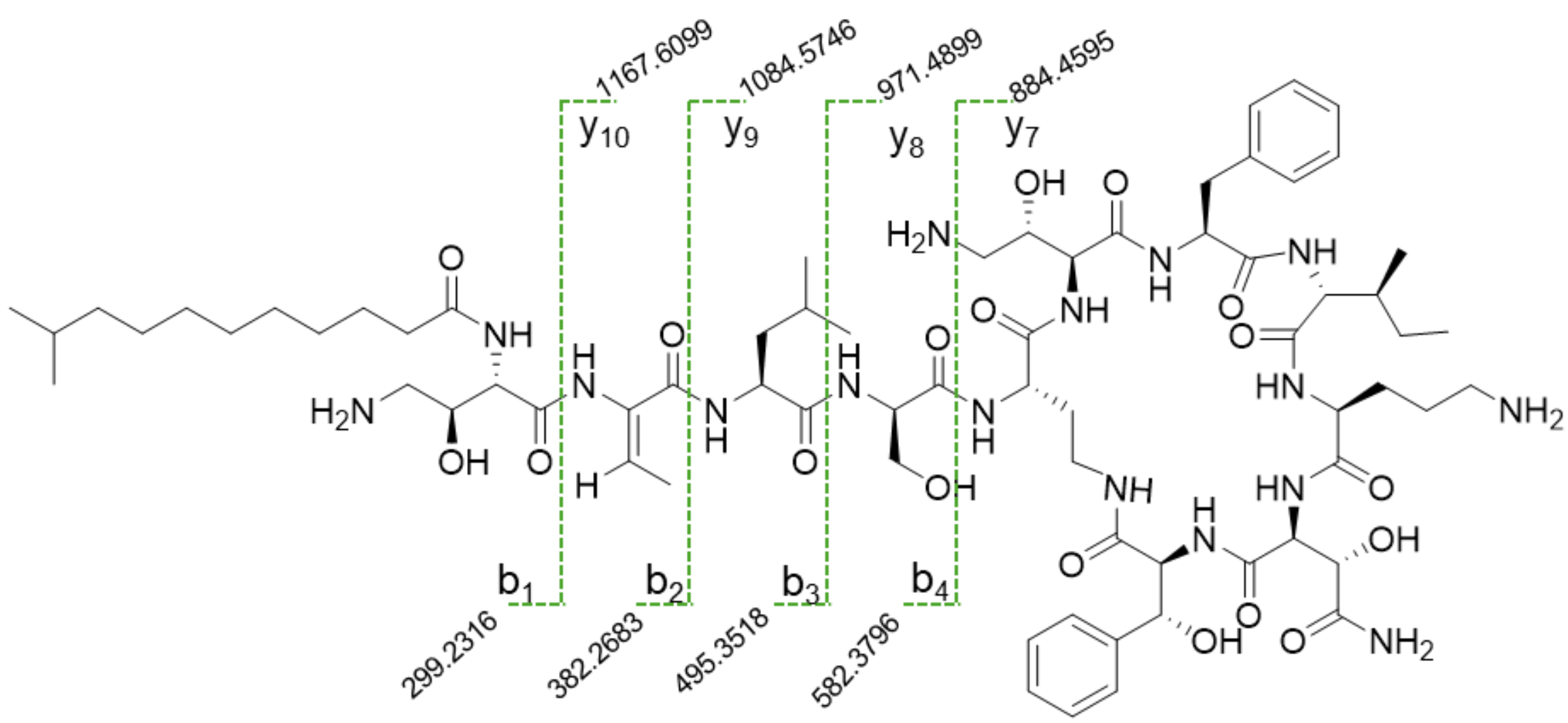

Figure S19. MS[2] fragmentation of marinocyclin B (**2**).

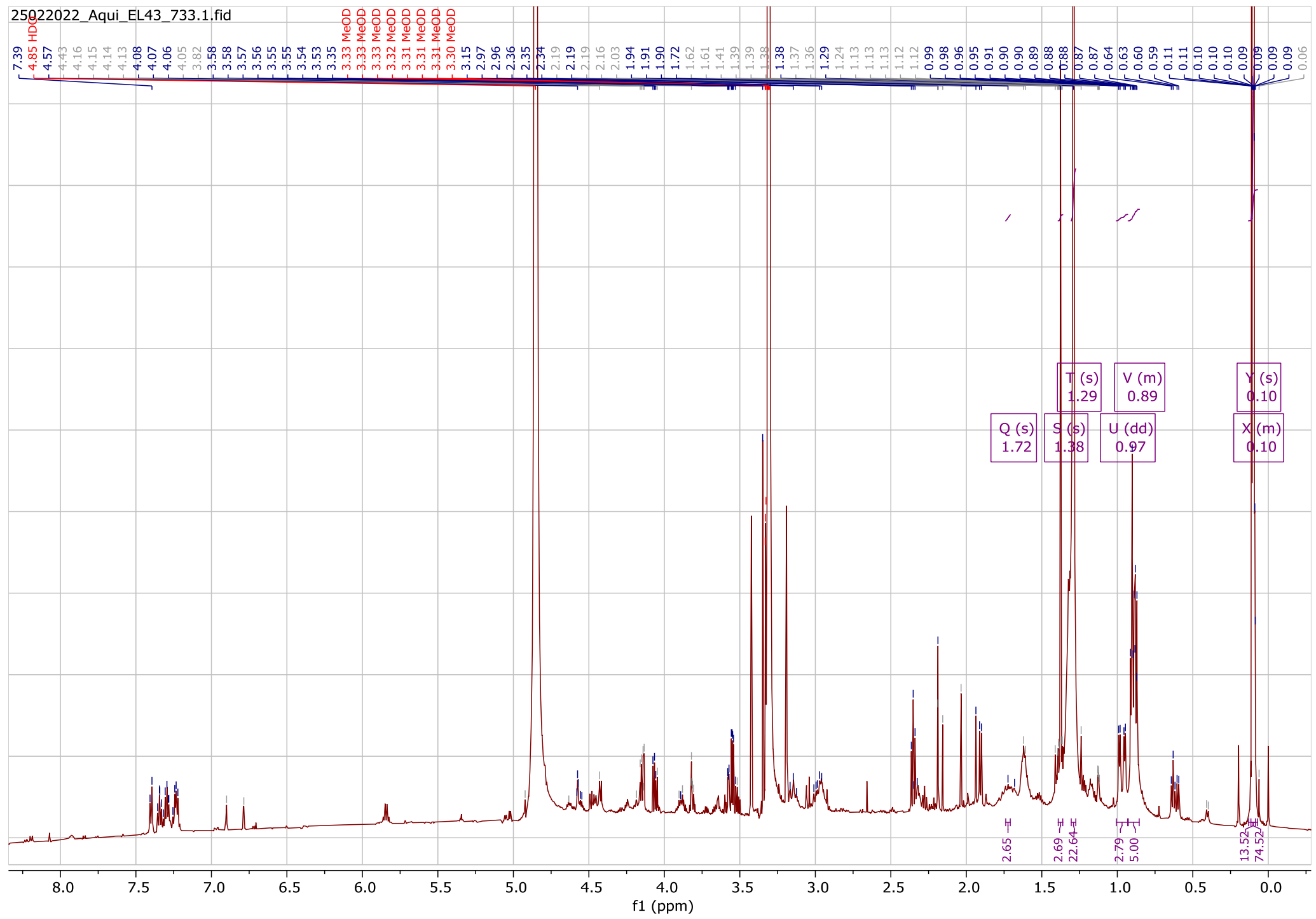

Figure S20. ^{1}H NMR spectrum of marinocyclin B (**2**) in methanol-d_4 (^{1}H 600 MHz).

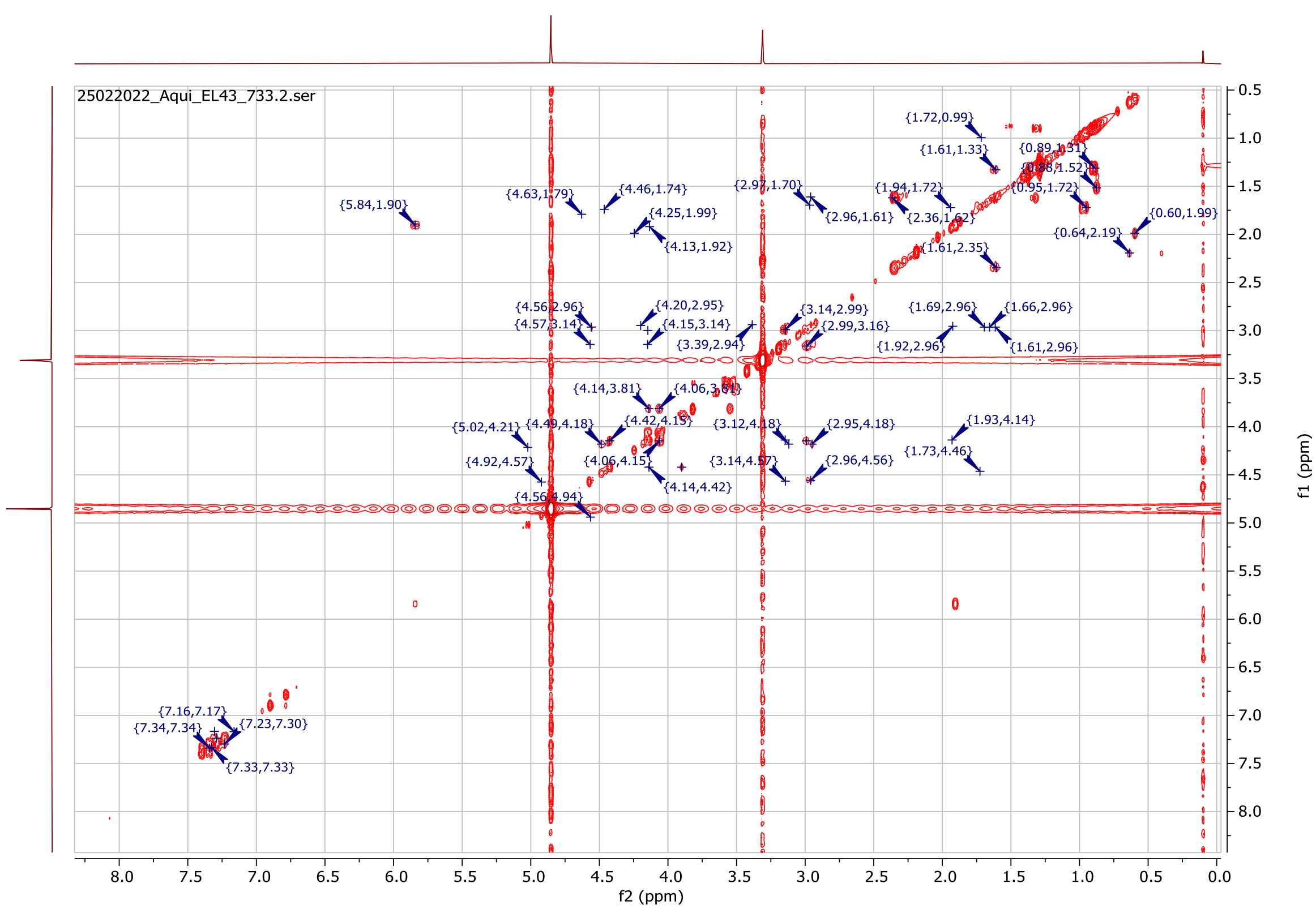

Figure S21. COSY spectrum of marinocyclin B (**2**) in methanol-d_4 (^{1}H 600 MHz).

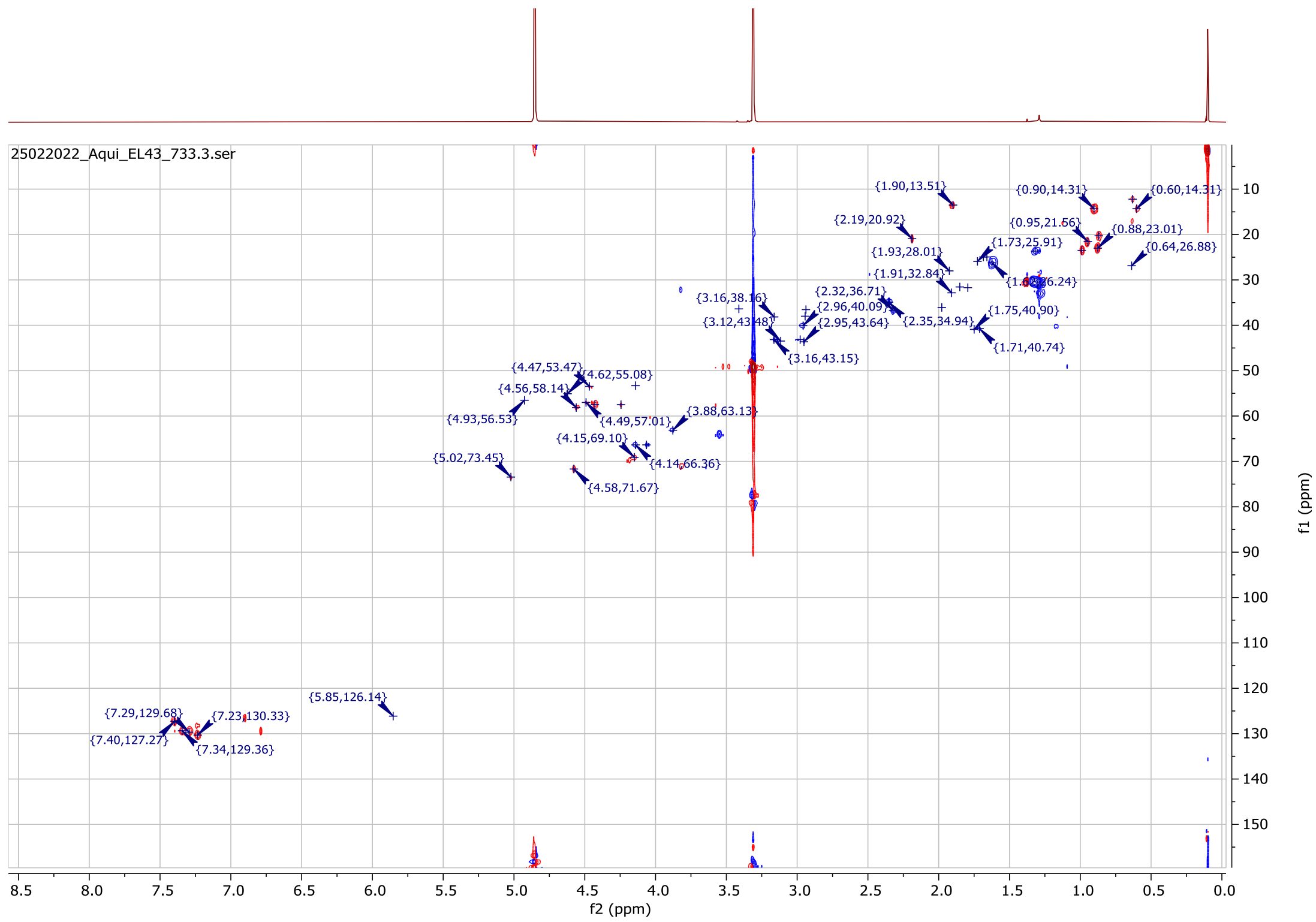

Figure S22. HSQC spectrum of marinocyclin B (**2**) in methanol-d4 (^{1}H 600 MHz, ^{13}C 150 MHz).

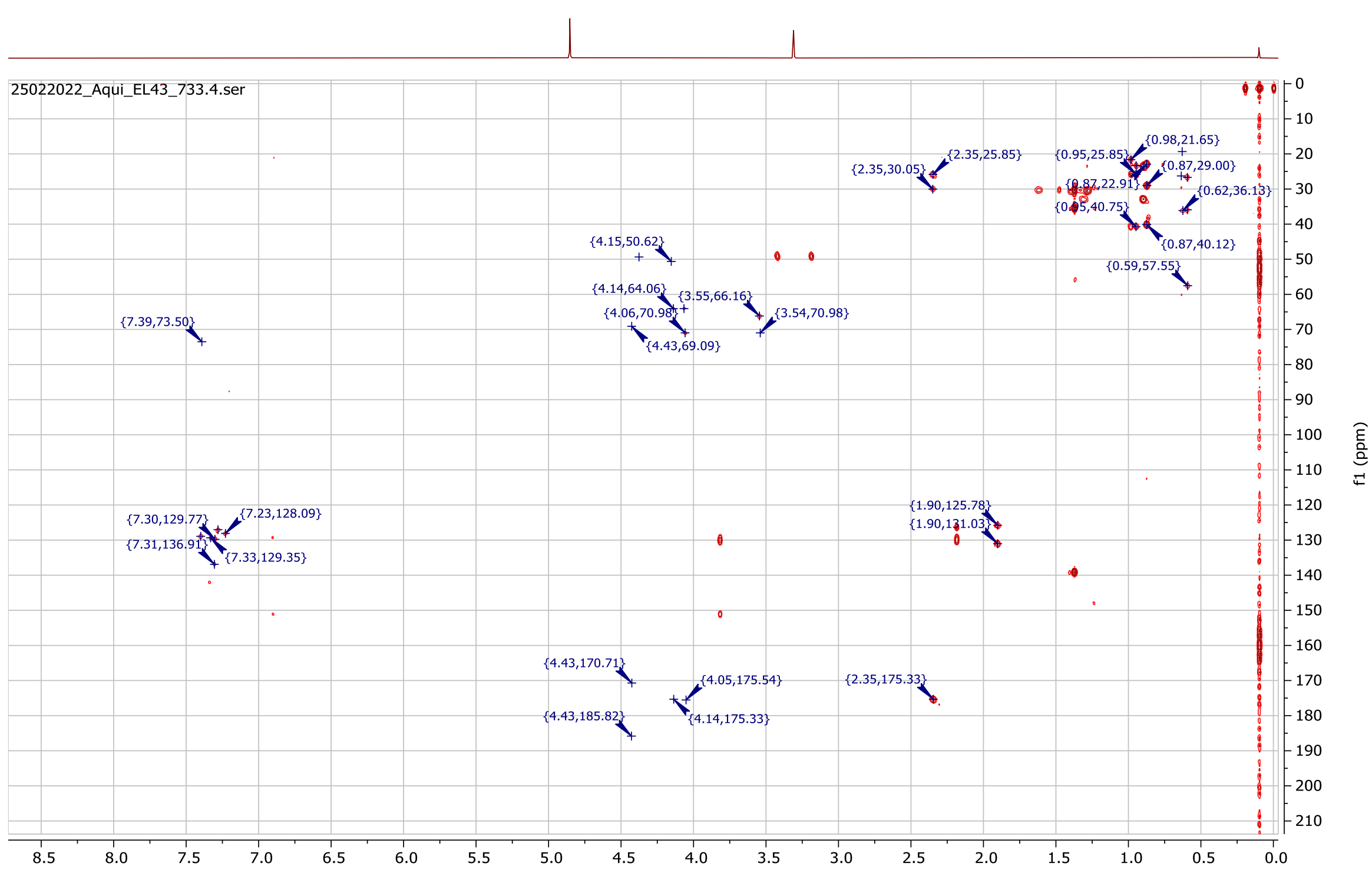

Figure S23. HMBC spectrum of marinocyclin B (**2**) in methanol-d4 (^{1}H 600 MHz, ^{13}C 150 MHz).

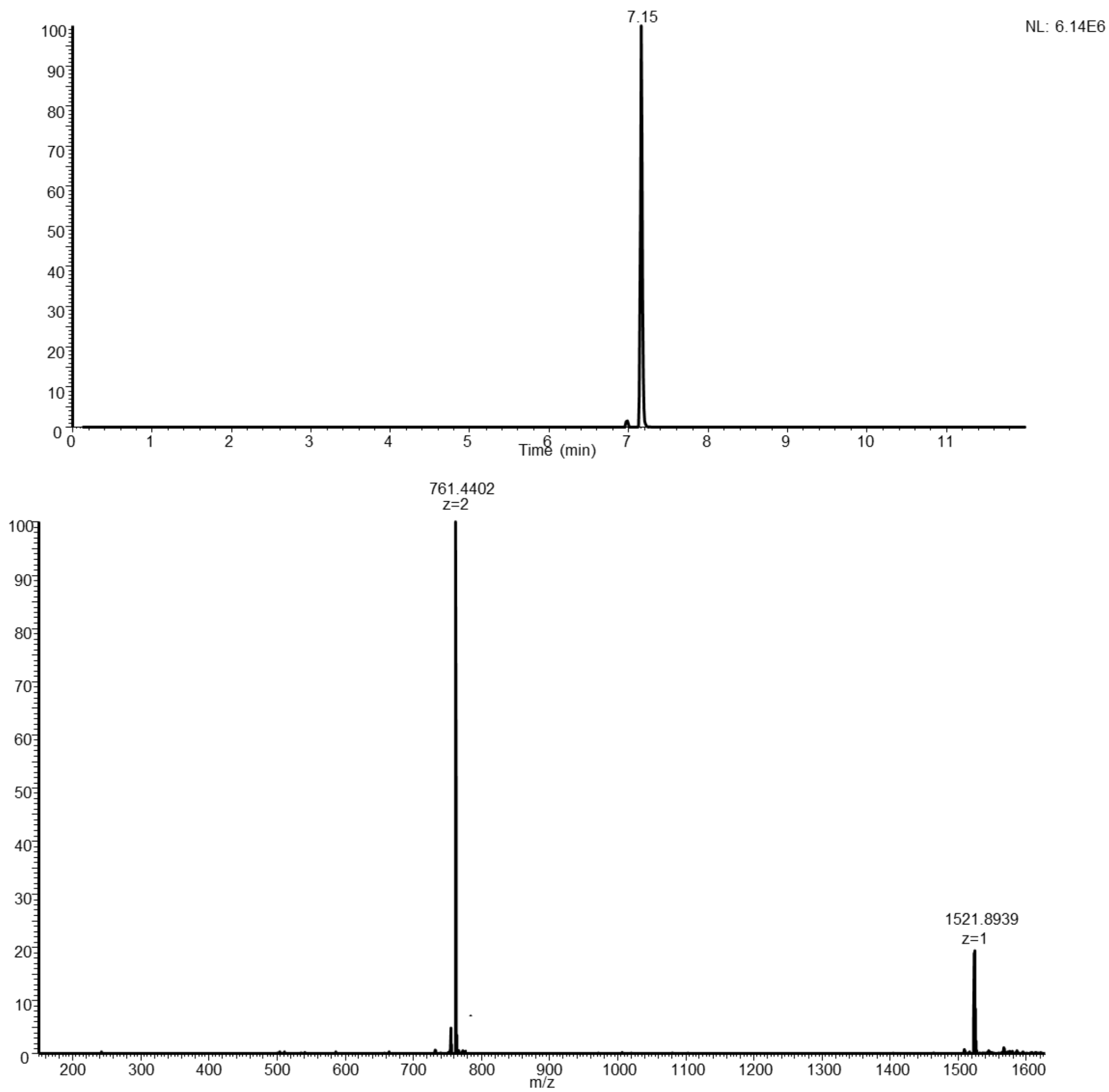

Figure S24. HPLC-HESI-HRMS data of marinocyclin C (**3**).Top: extracted ion chromatogram m/z 1521.8739, bottom: MS data of marinocyclin C (**3**) (*m/z* 1521.8739 $[M+H]^+$).

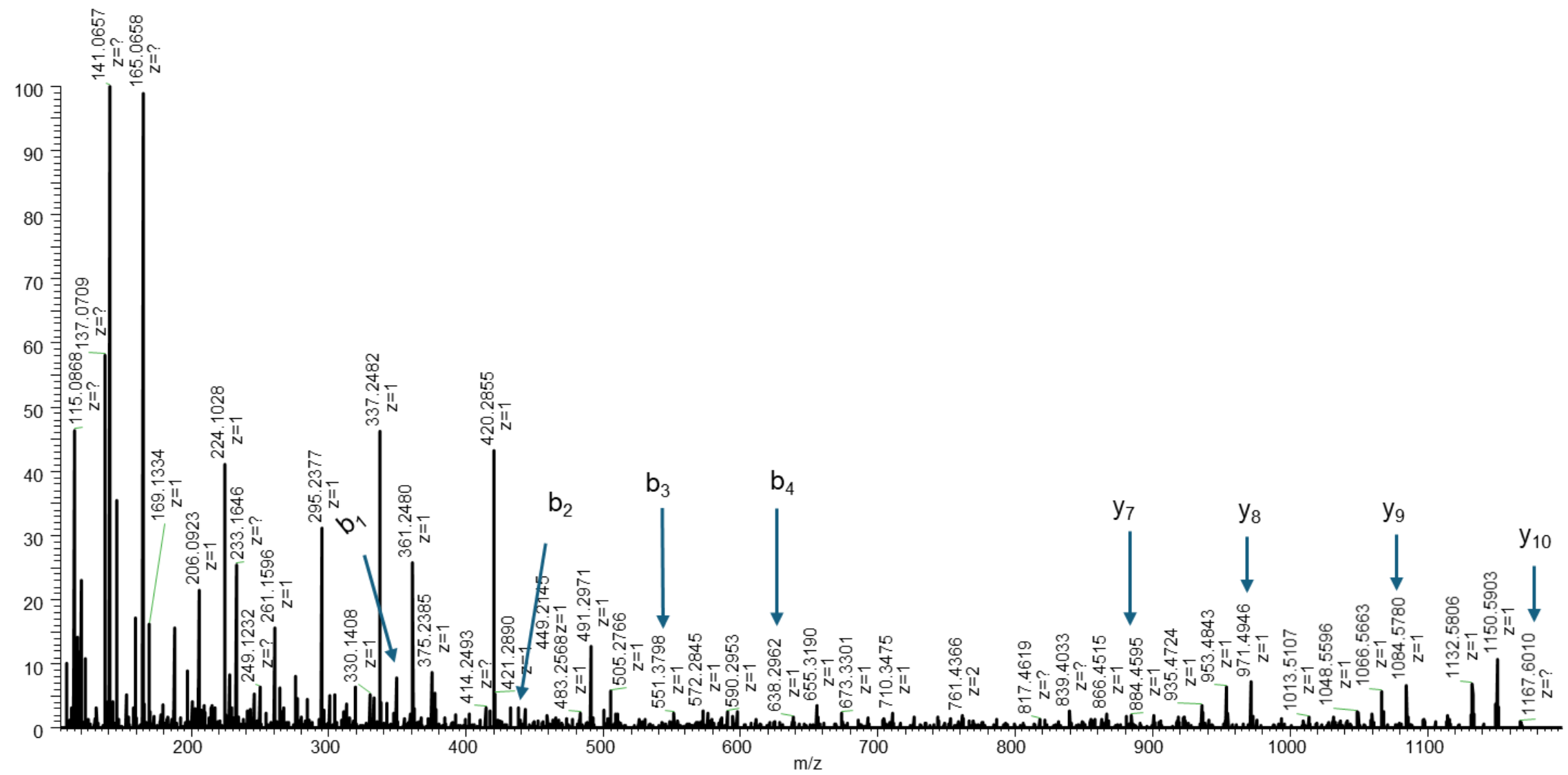

Figure S25. HESI-HRMS^2 data of marinocyclin C (**3**) (*m/z* 761.4381 $[M+H]^{2+}$).

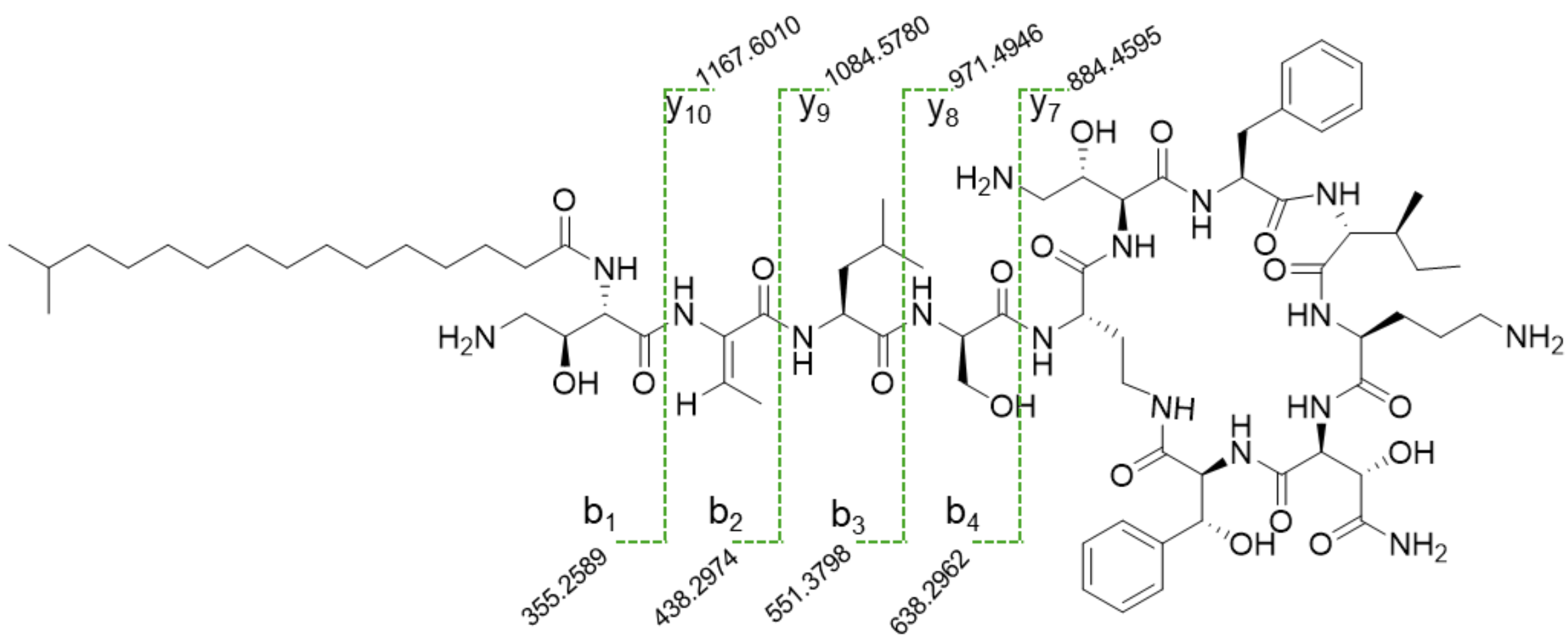

Figure S26. MS^2 fragmentation of marinocyclin C (**3**).

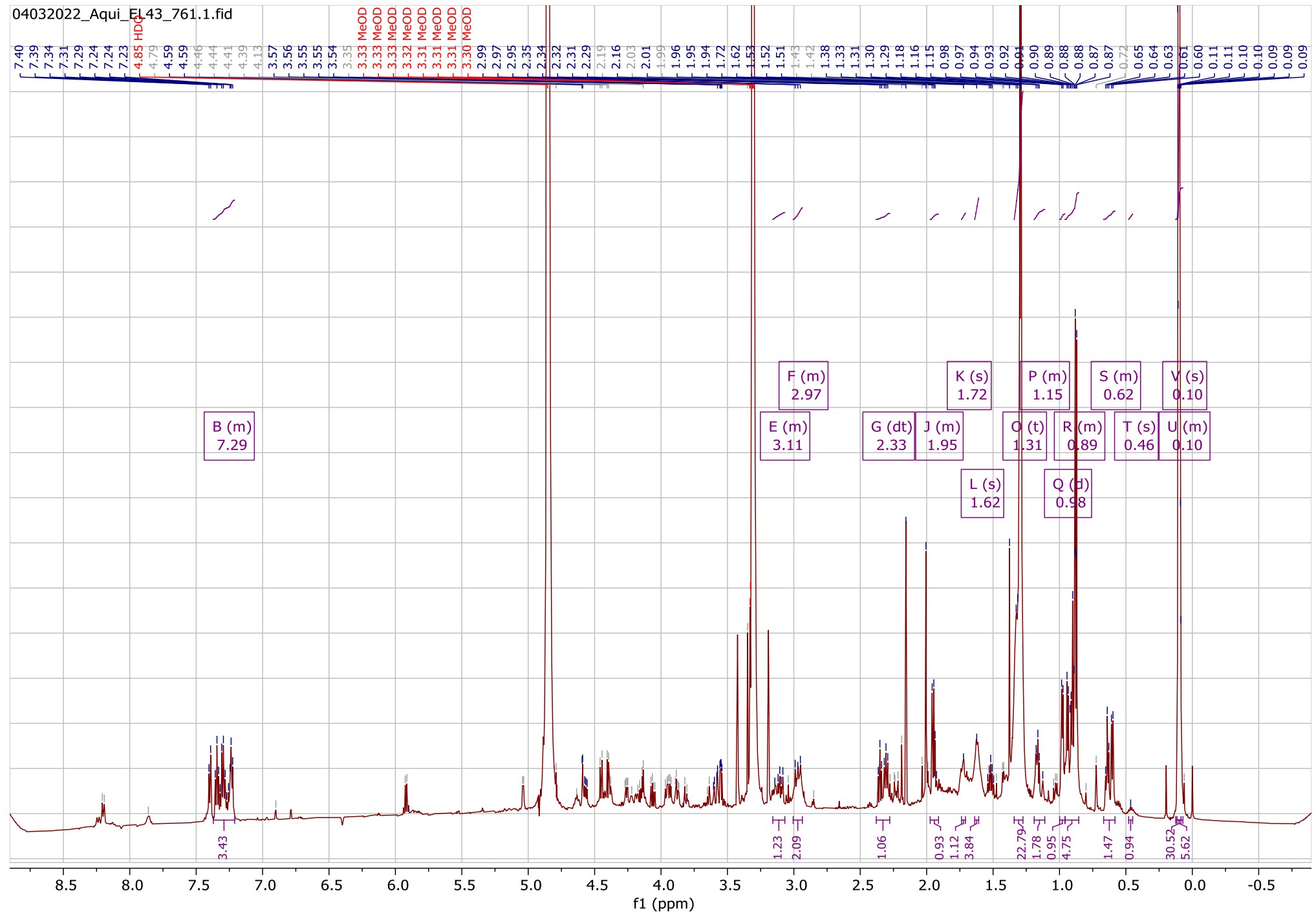

Figure S27. ^{1}H NMR spectrum of marinocyclin C (**3**) in methanol-d_4 (^{1}H 600 MHz).

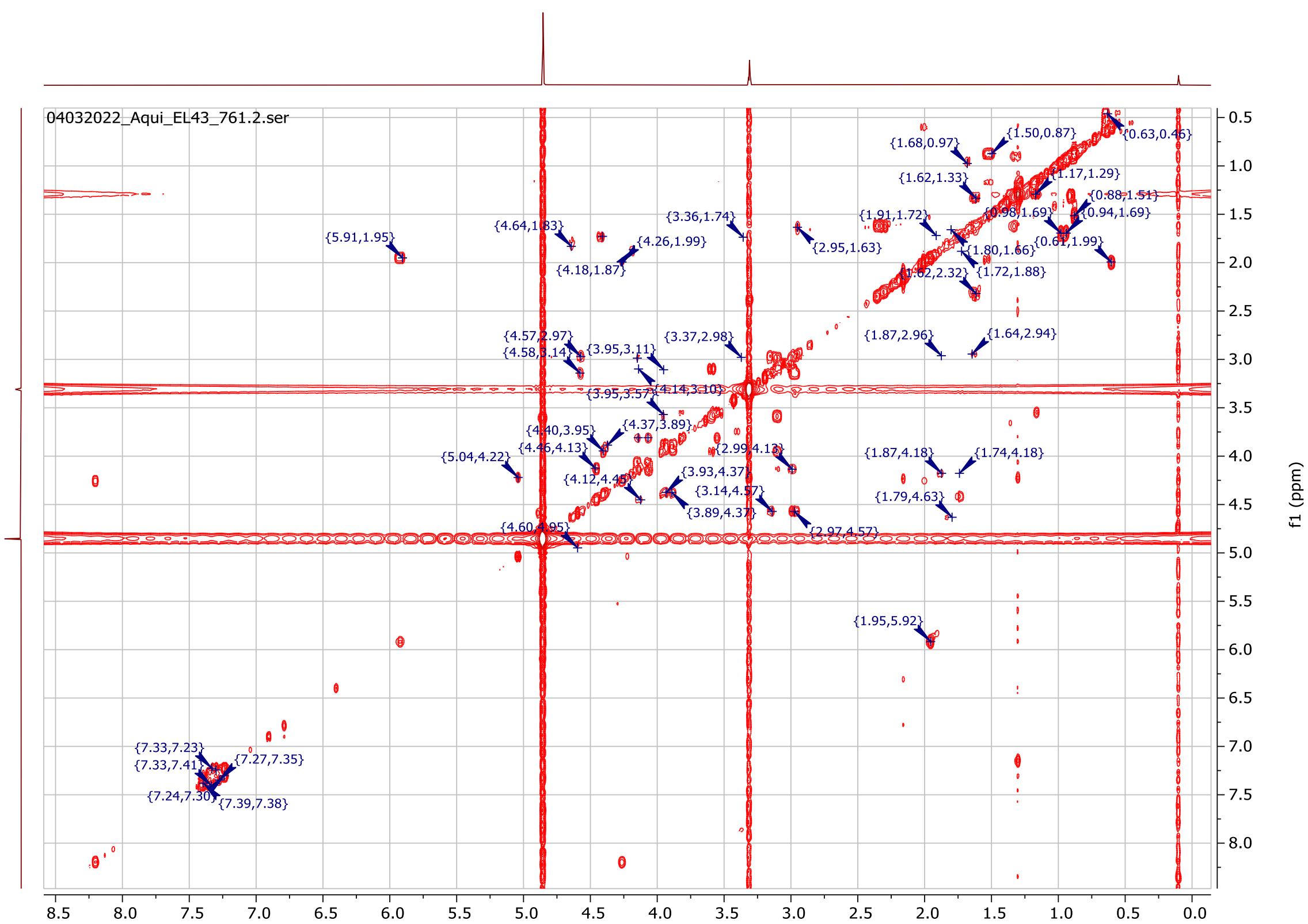

Figure S28. COSY spectrum of marinocyclin C (**3**) in methanol-d_4 (^{1}H 600 MHz).

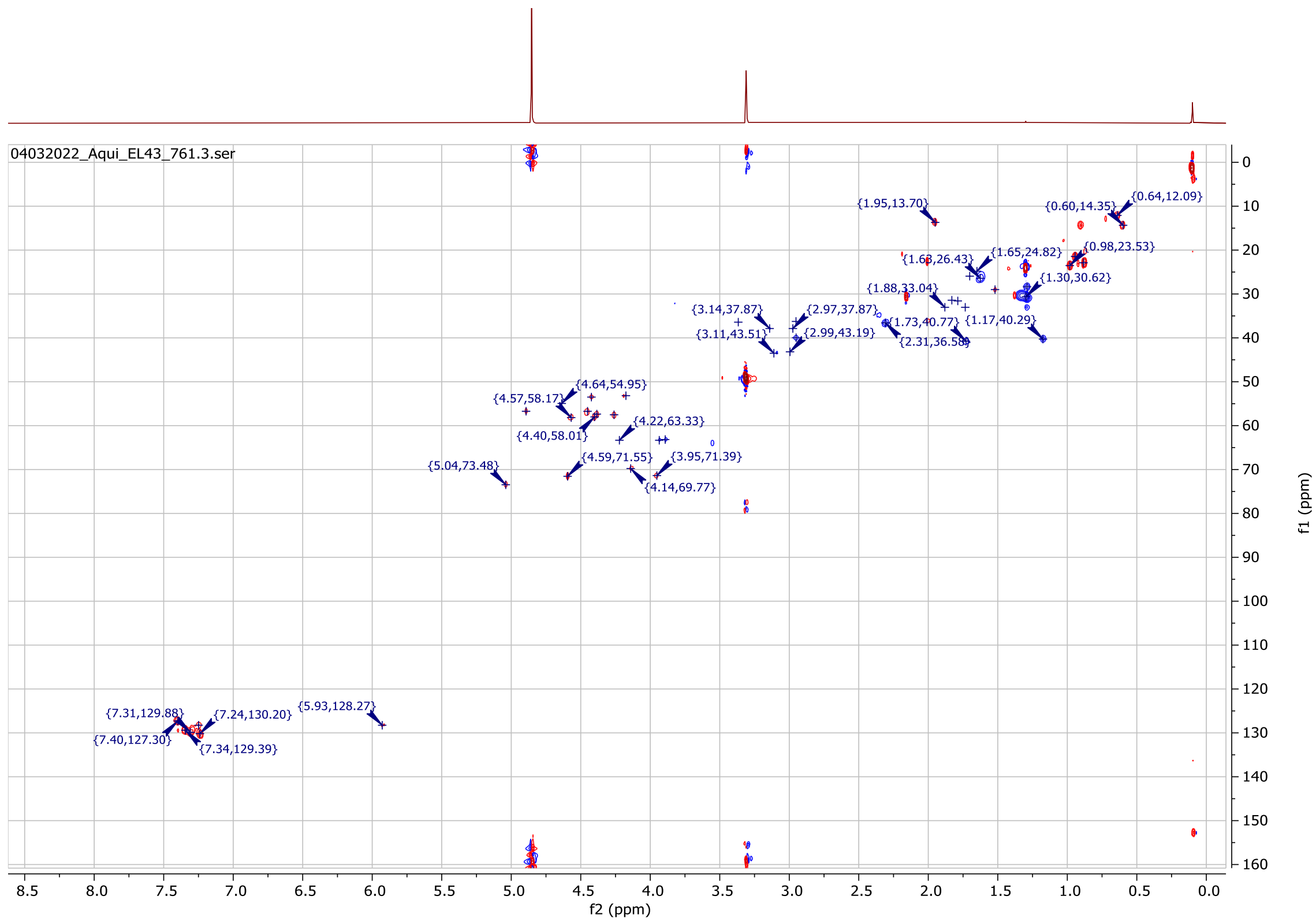

Figure S29. HSQC spectrum of marinocyclin C (**3**) in methanol-d4 (^{1}H 600 MHz, ^{13}C 150 MHz).

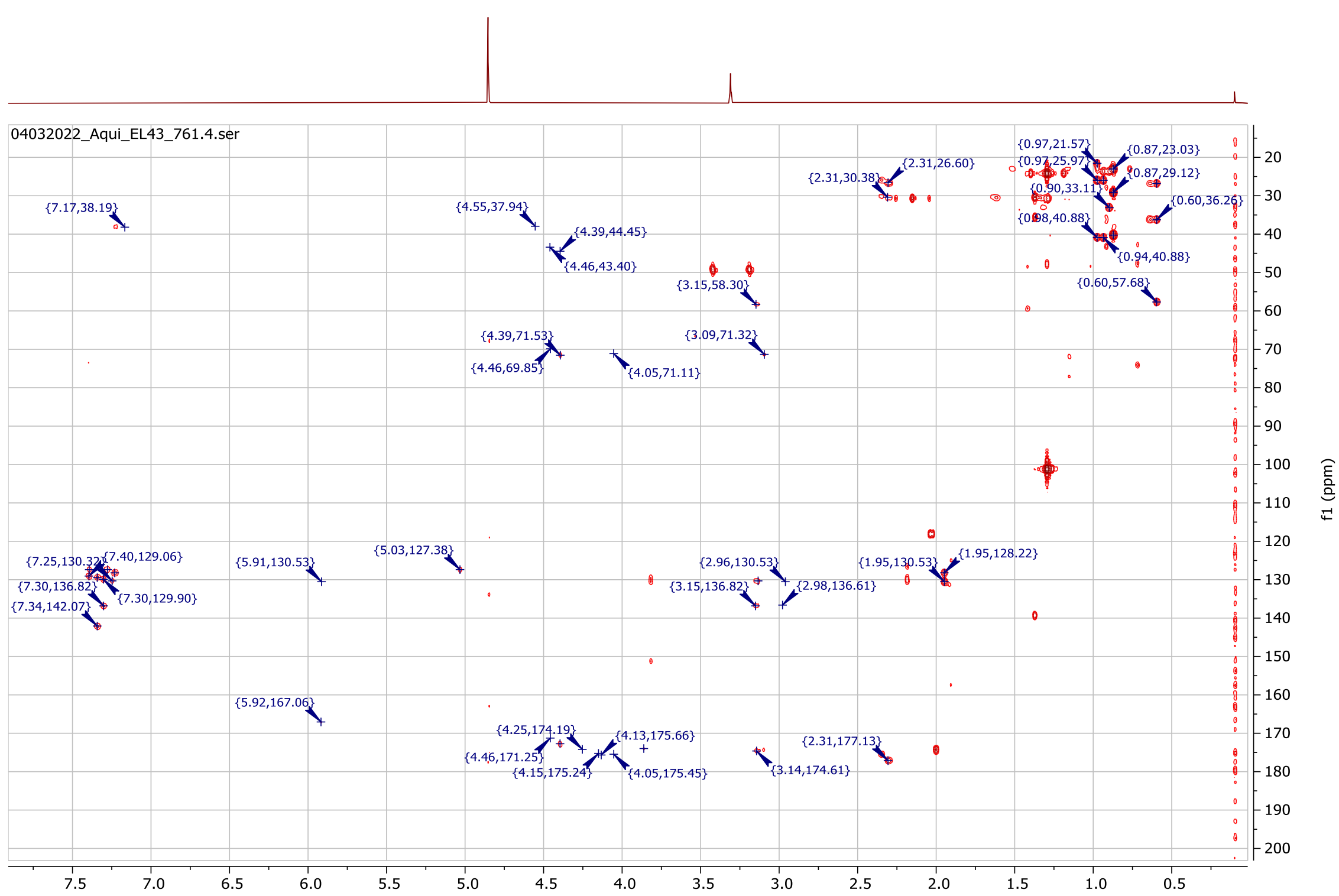

Figure S30. HMBC spectrum of marinocyclin C (**3**) in methanol-d4 (^{1}H 600 MHz, ^{13}C 150 MHz).

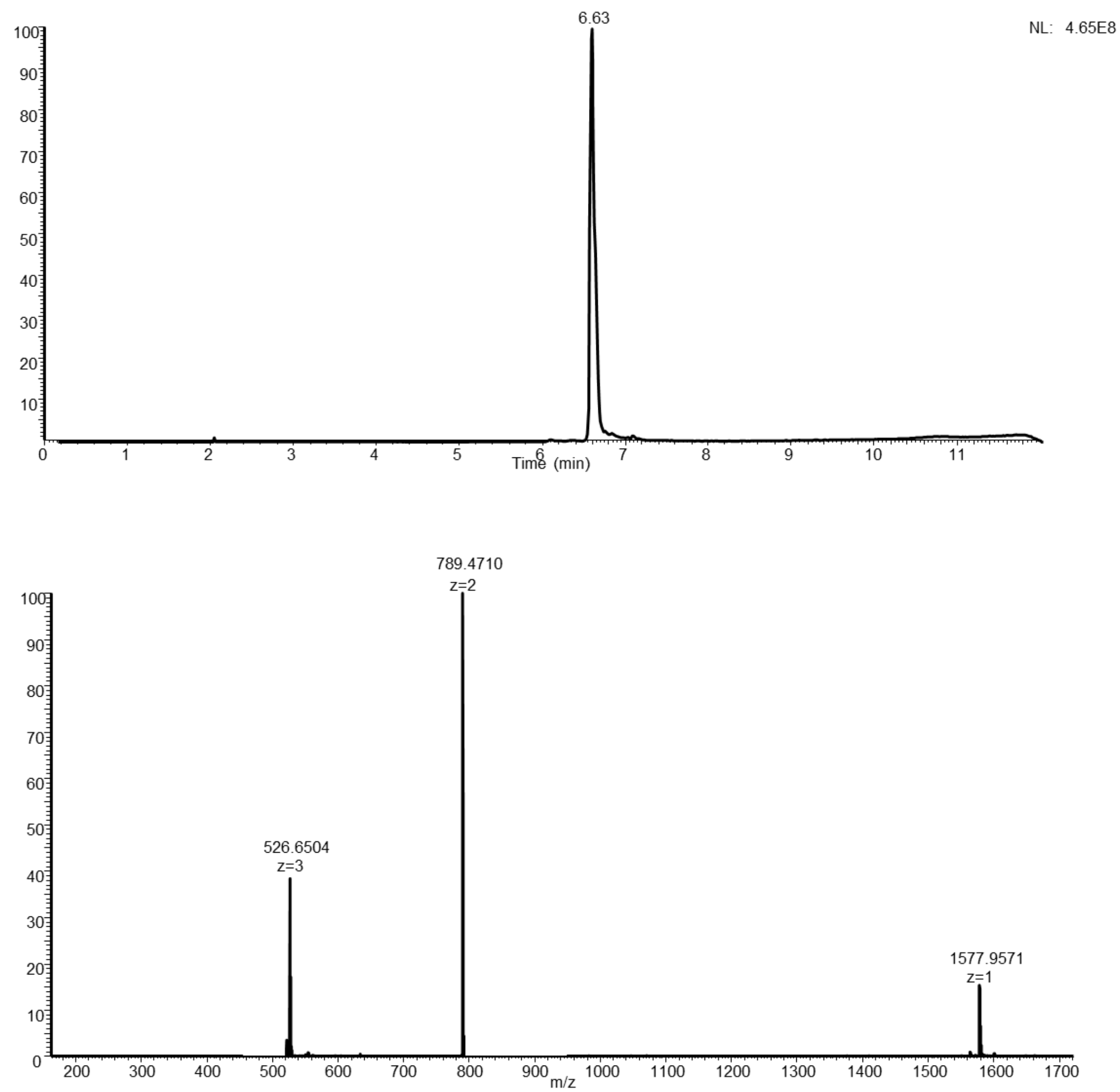

Figure S31. HPLC-HESI-HRMS data of marinocyclin D (**4**).Top: extracted ion chromatogram m/z 1577.9371, bottom: MS data of marinocyclin D (**4**) (*m/z* 1577.9371 $[M+H]^+$).

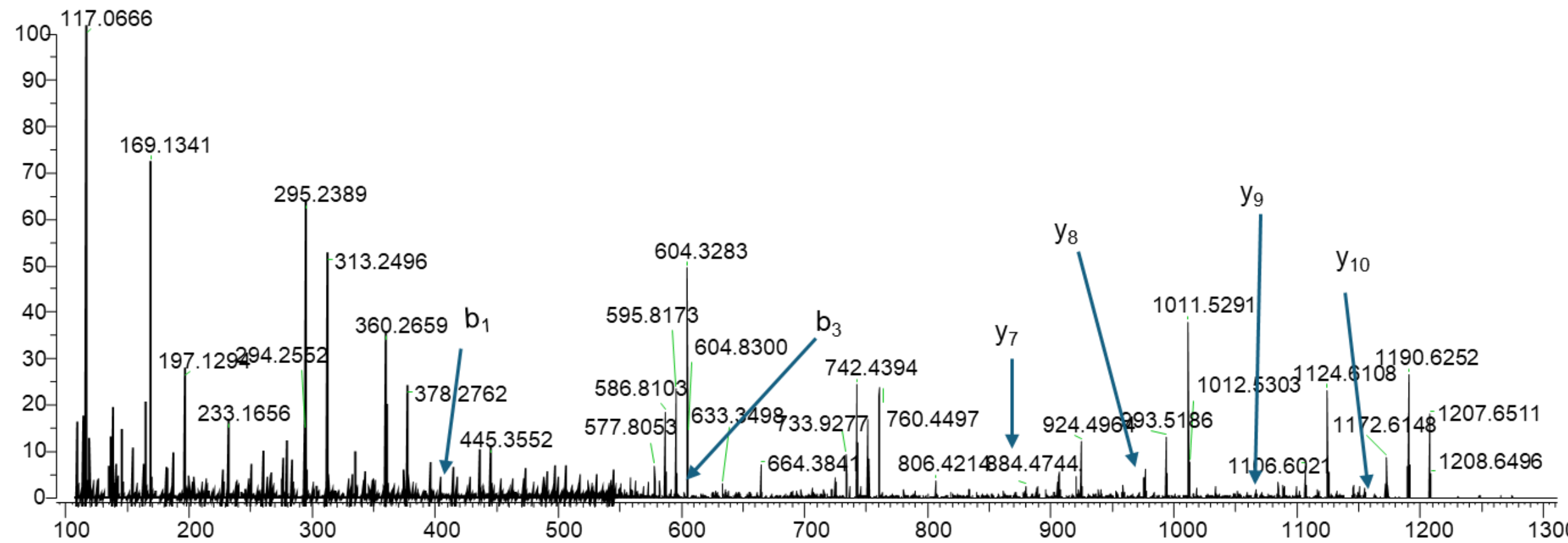

Figure S32. HESI-HRMS^2 data of marinocyclin D (**4**) (m/z 789.4710 [M+H]2+).

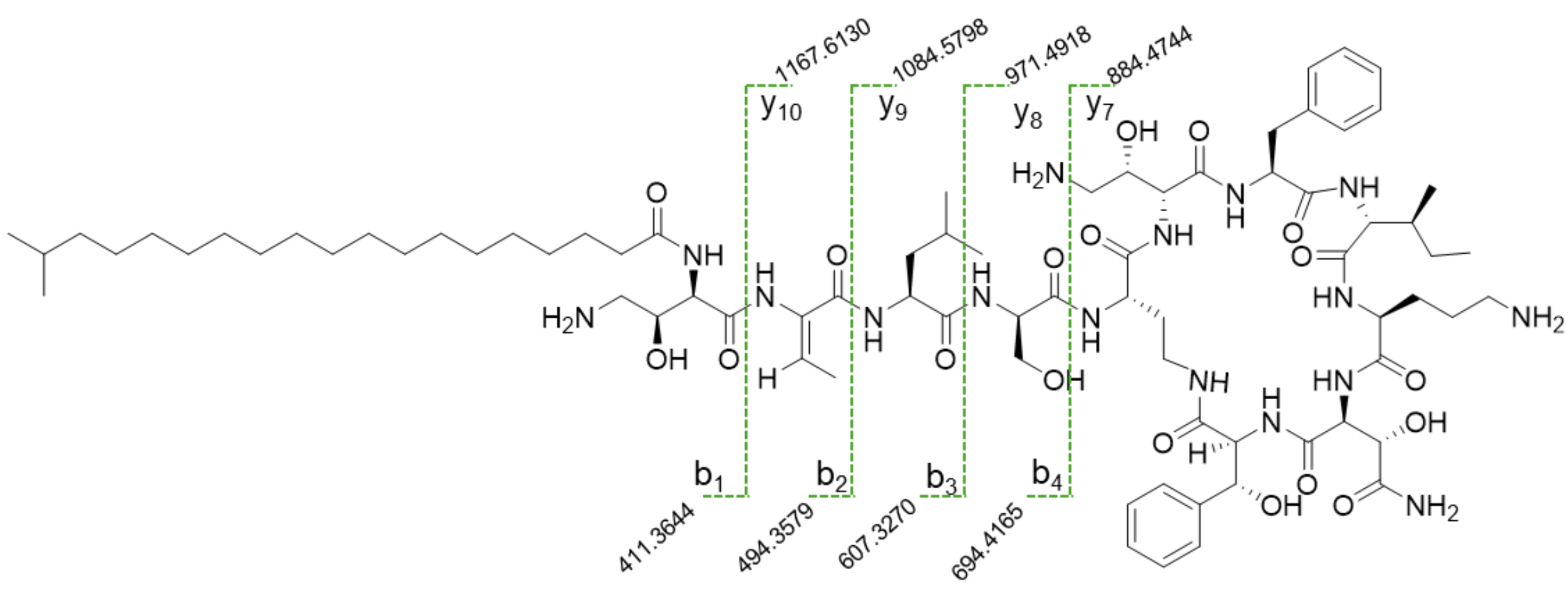

Figure S33. MS^2 fragmentation of marinocyclin D (**4**).

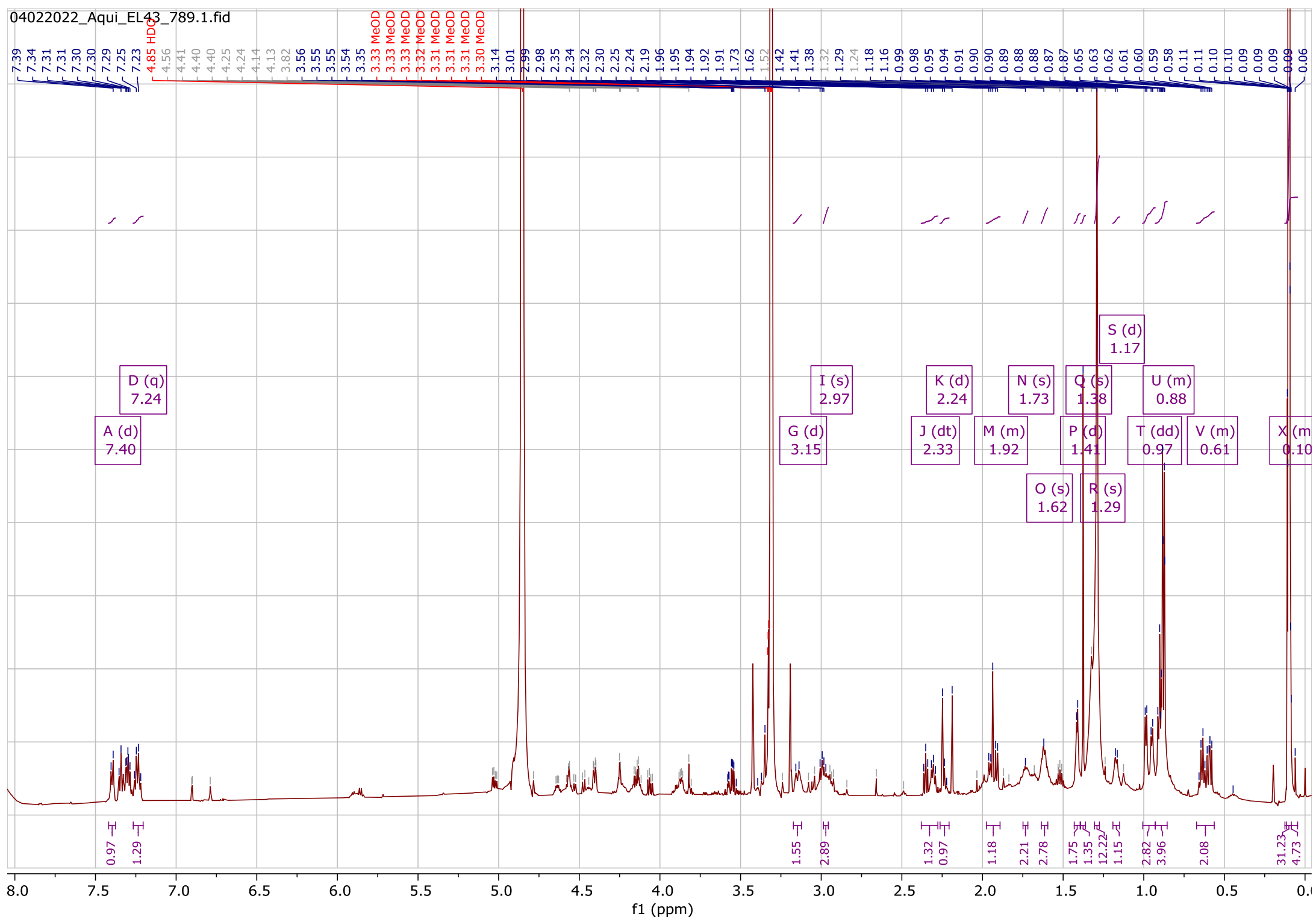

Figure S34. ^{1}H spectrum of marinocyclin D (**4**) in methanol-d_4 (^{1}H 600 MHz).

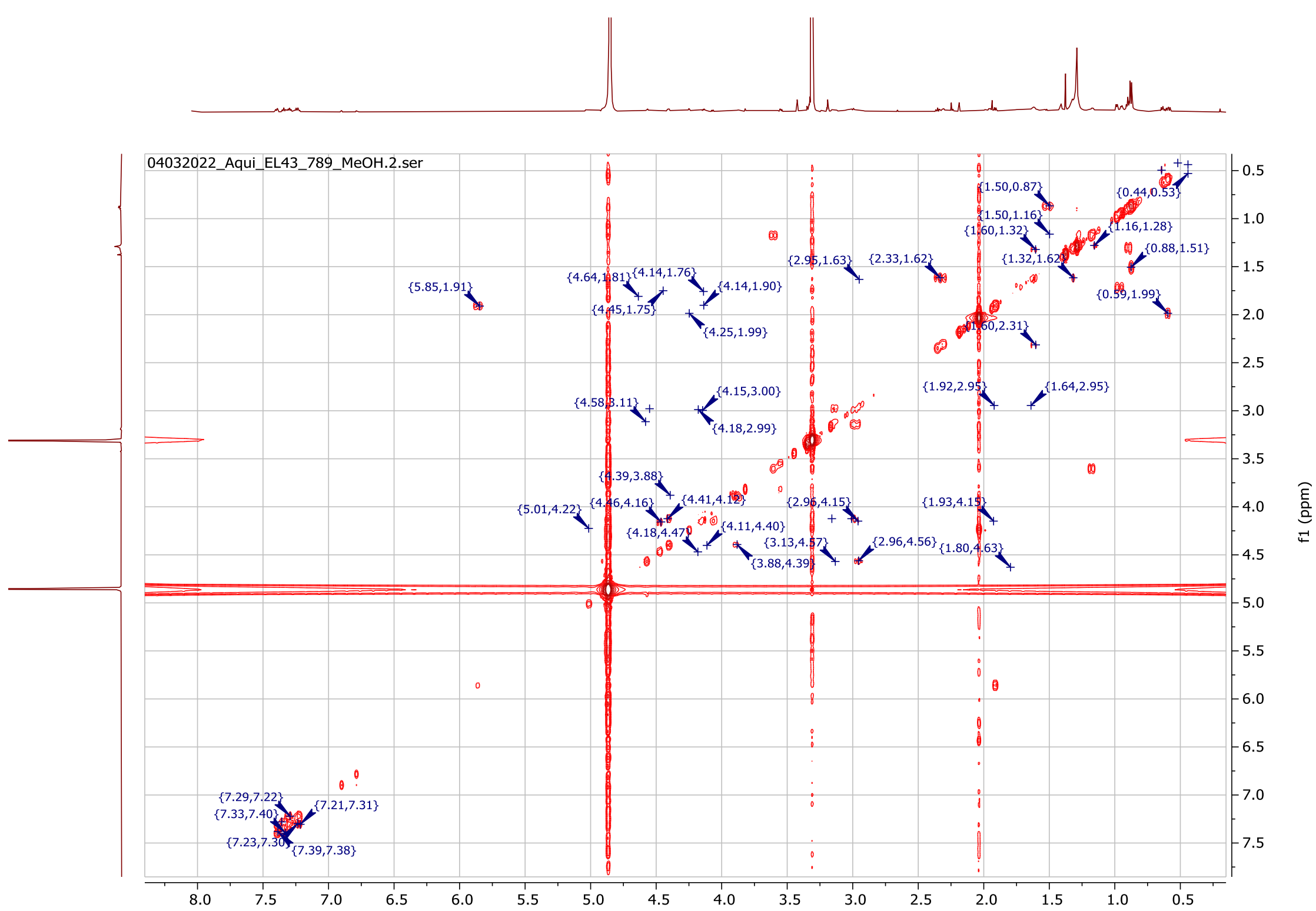

Figure S35. COSY spectrum of marinocyclin D (**4**) in methanol-d_4 (^{1}H 600 MHz).

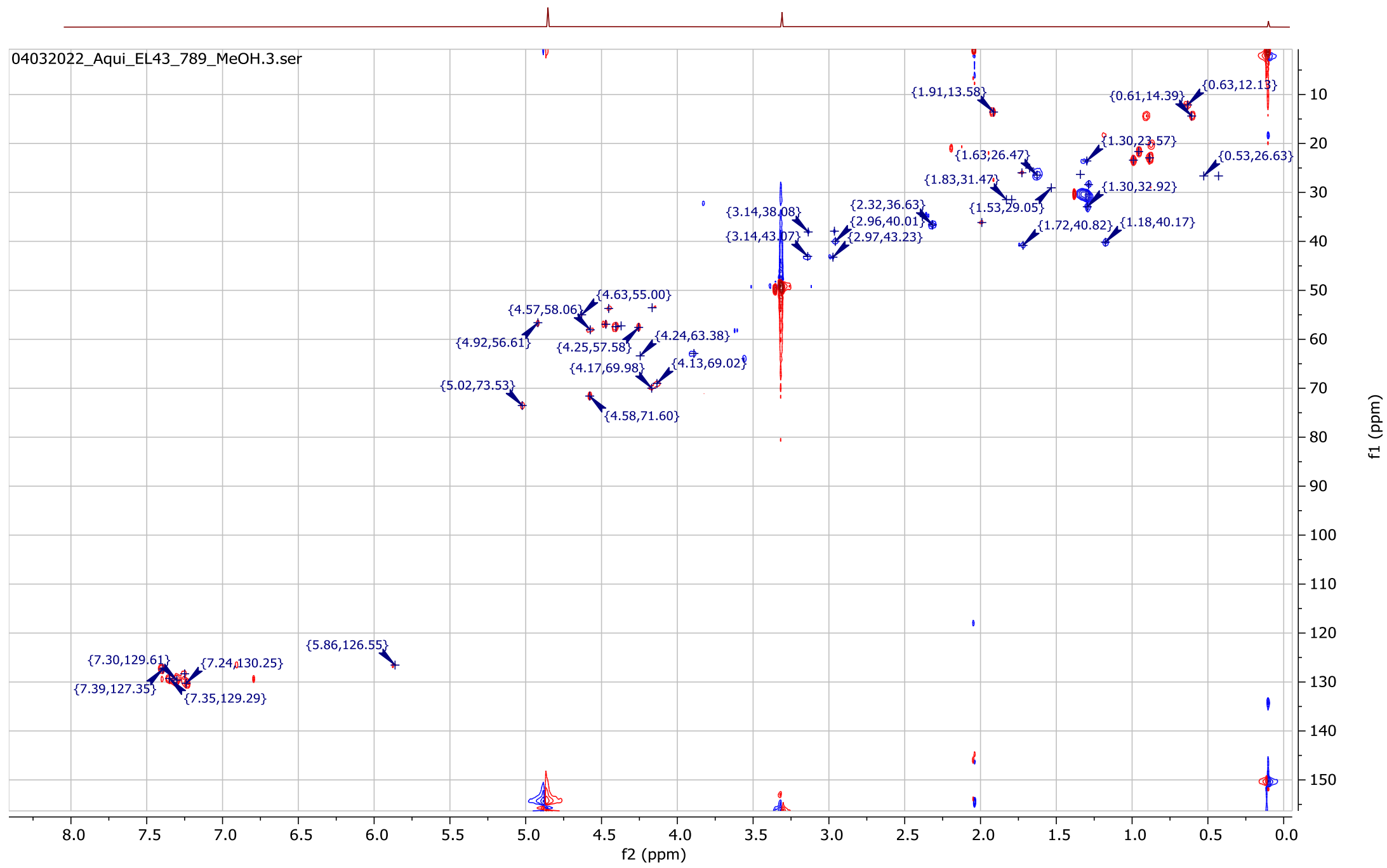

Figure S36. HSQC spectrum of marinocyclin D (**4**) in methanol-d4 (1H 600 MHz, 13C 150 MHz).

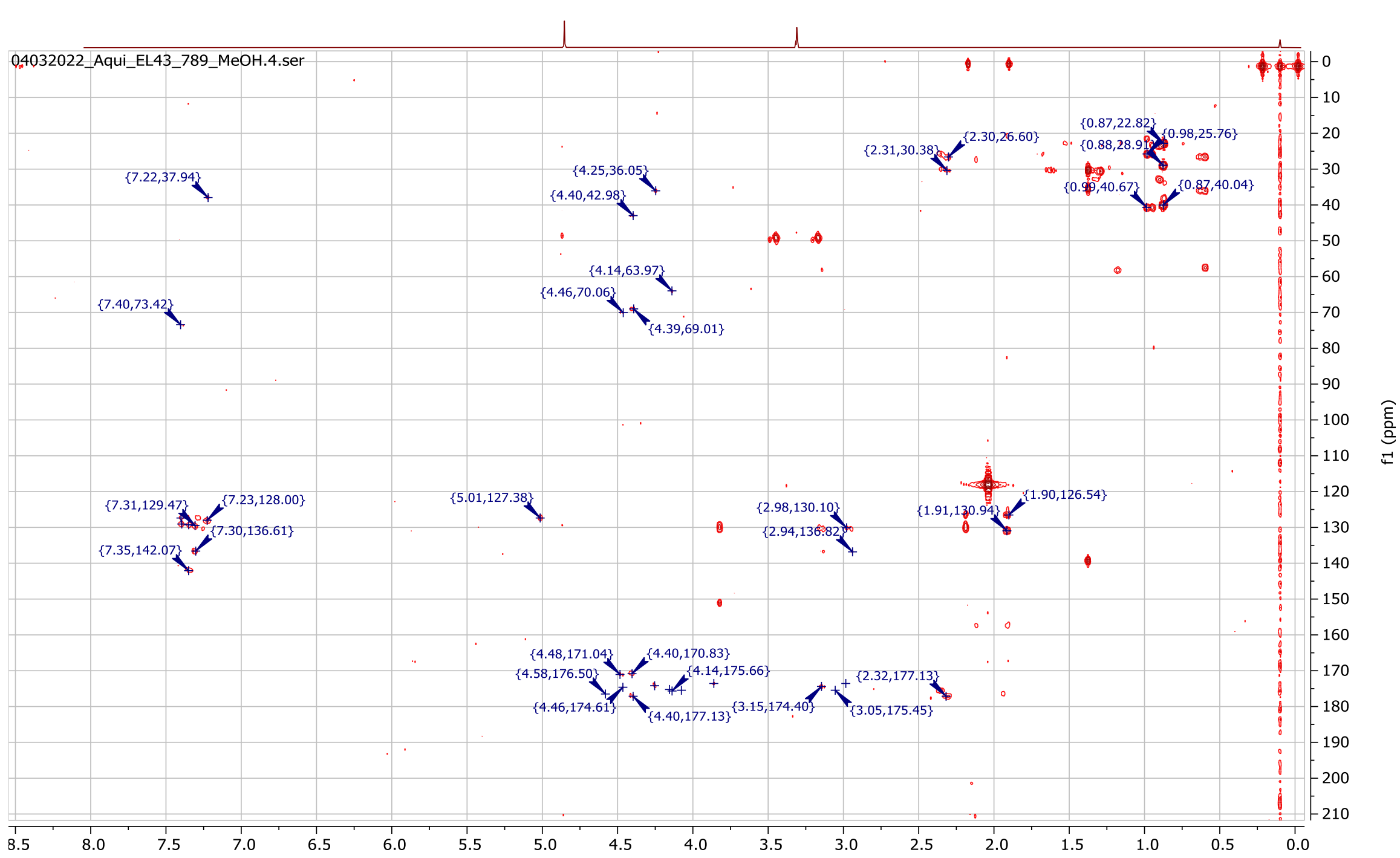

Figure S37. HMBC spectrum of marinocyclin D (**4**) in methanol-d4 (1H 600 MHz, 13C 150 MHz).

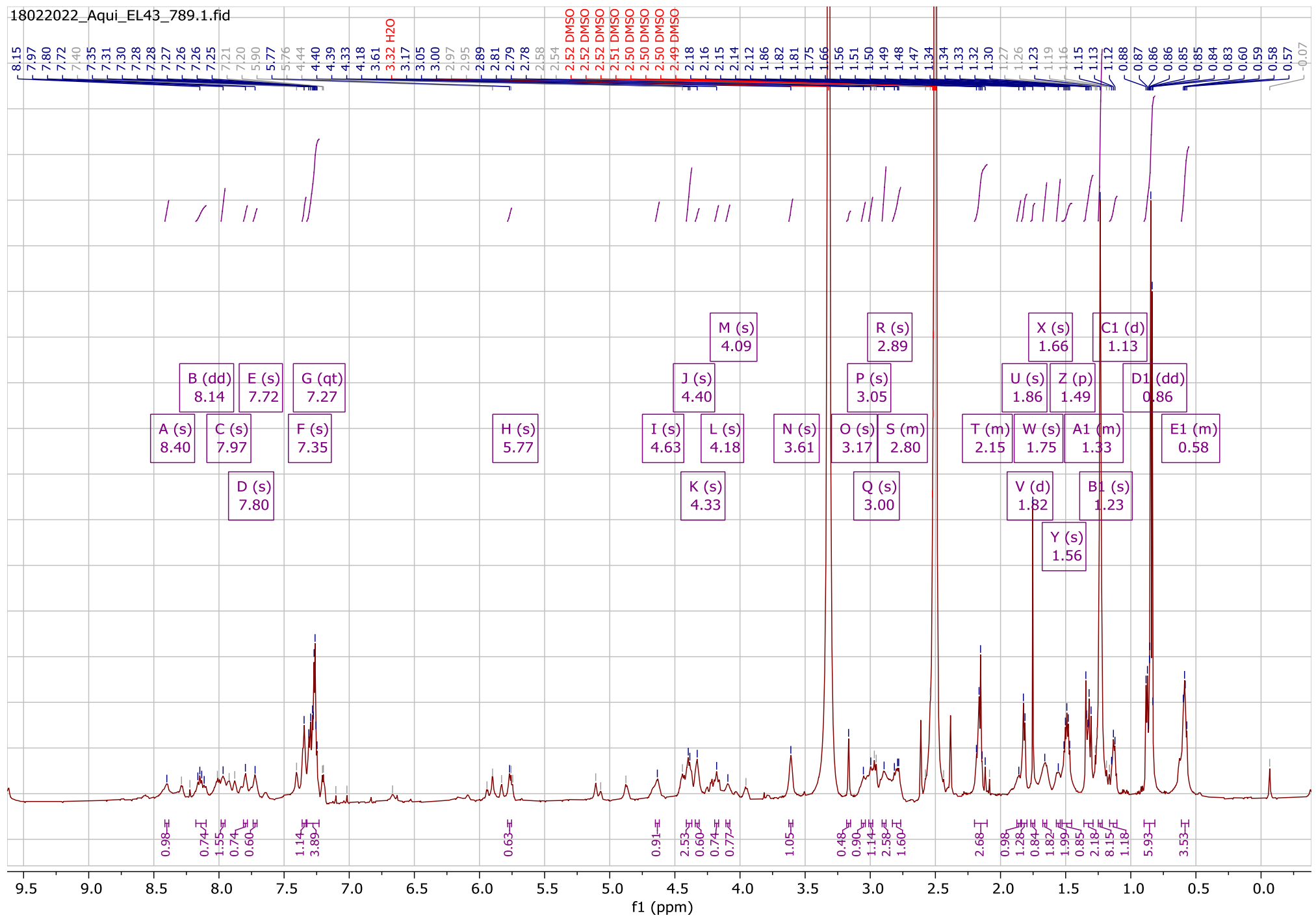

Figure S38. ^{1}H NMR spectrum of marinocyclin D (**4**) in DMSO-d_6 (^{1}H 600 MHz).

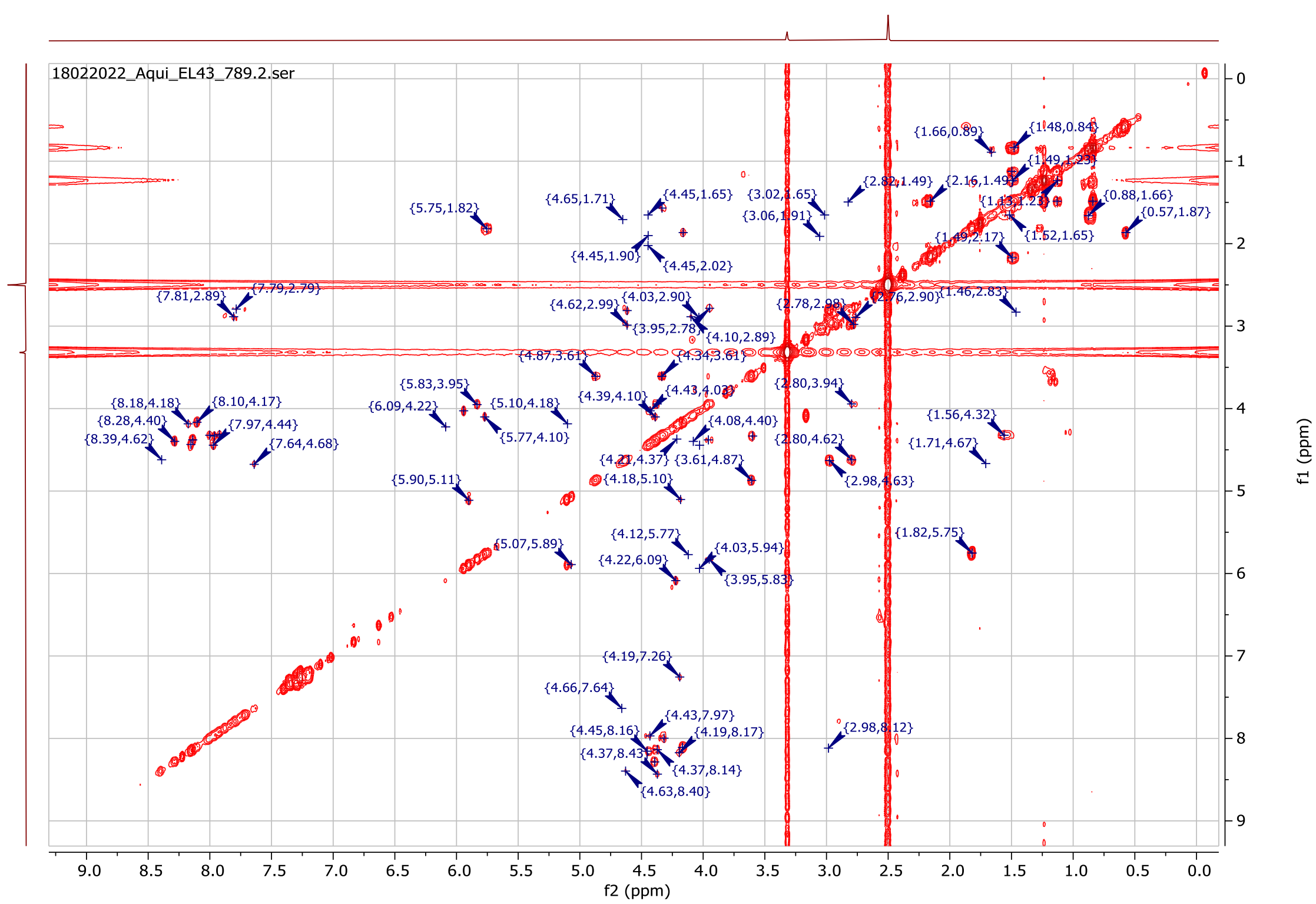

Figure S39. COSY spectrum of marinocyclin D (**4**) in DMSO-d_6 (^{1}H 600 MHz).

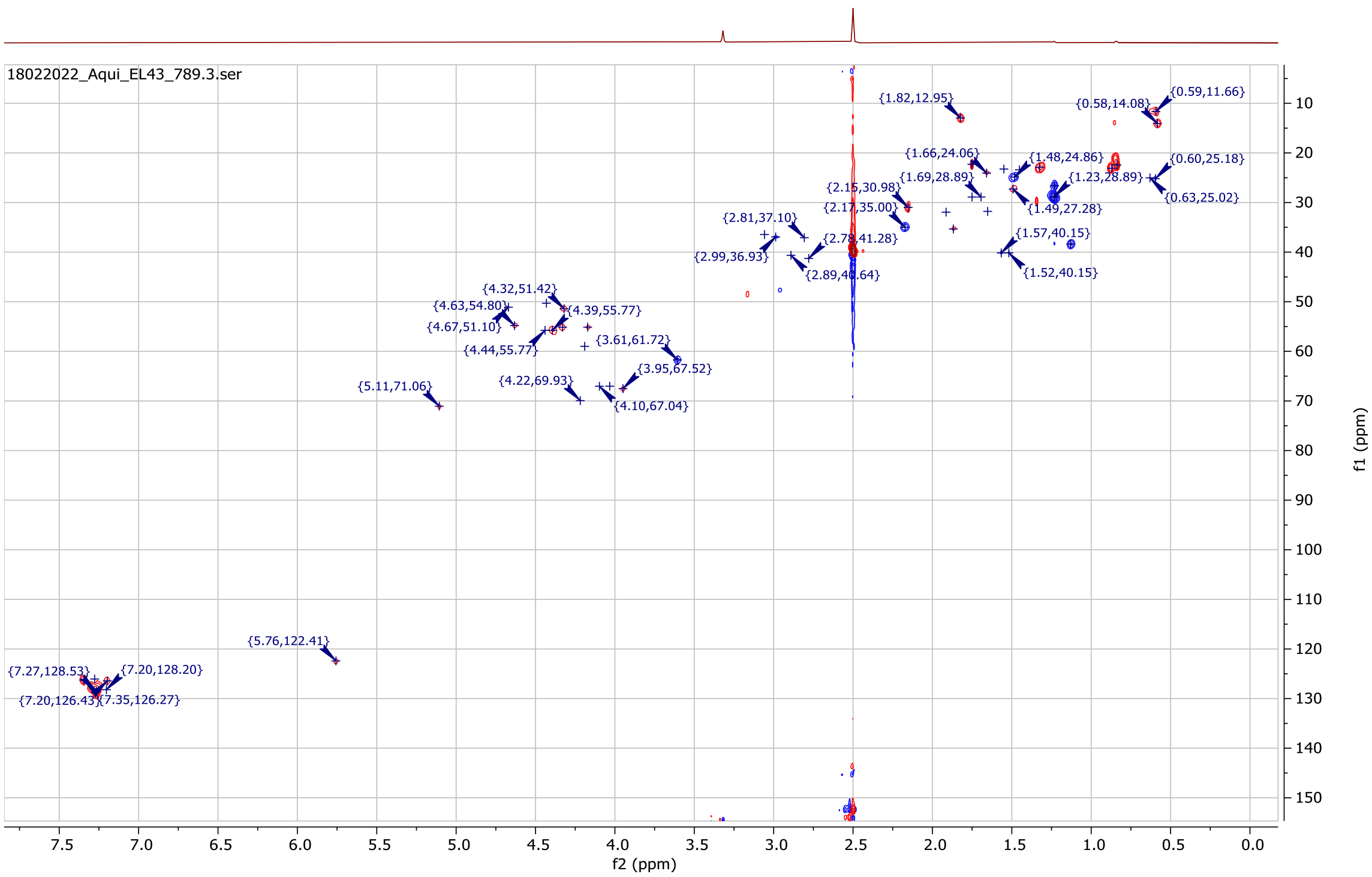

Figure S40. HSQC spectrum of marinocyclin D (**4**) in DMSO-d_6 (^{1}H 600 MHz, ^{13}C 150 MHz).

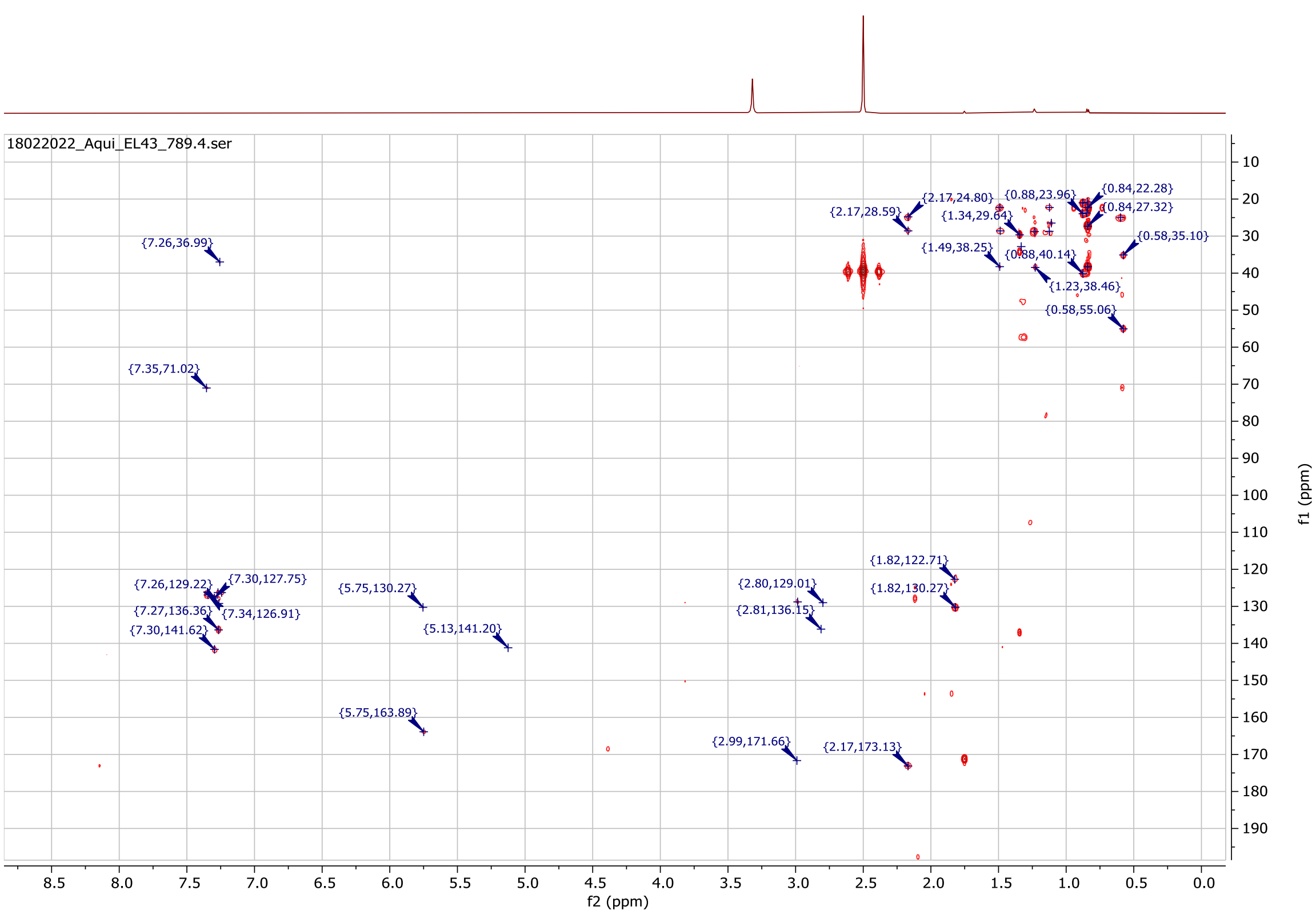

Figure S41. HMBC spectrum of marinocyclin D (**4**) in DMSO-d_6 (^{1}H 600 MHz, ^{13}C 150 MHz).

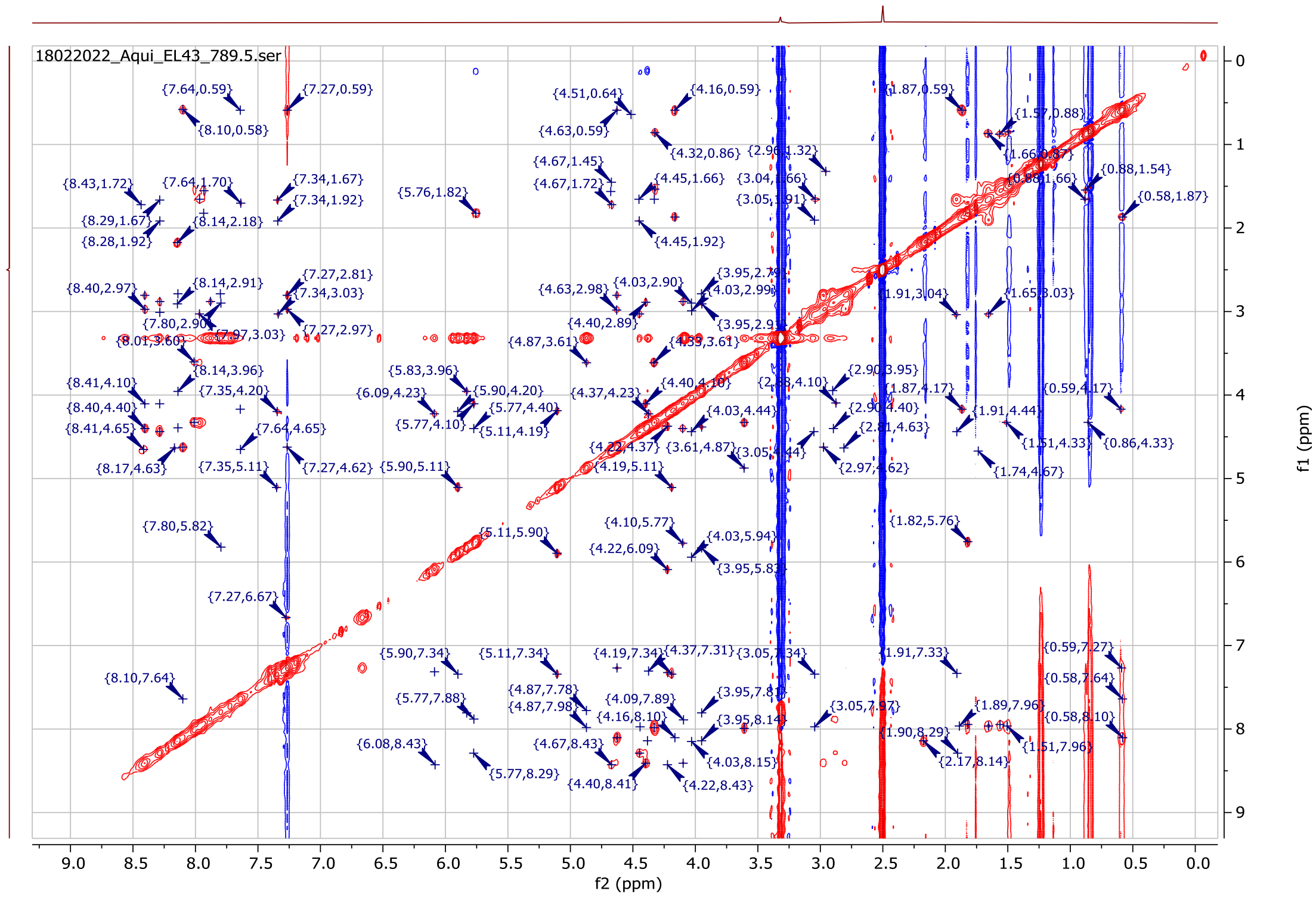

Figure S42. NOESY spectrum of marinocyclin D (**4**) in DMSO-d6 (^{1}H 600 MHz).

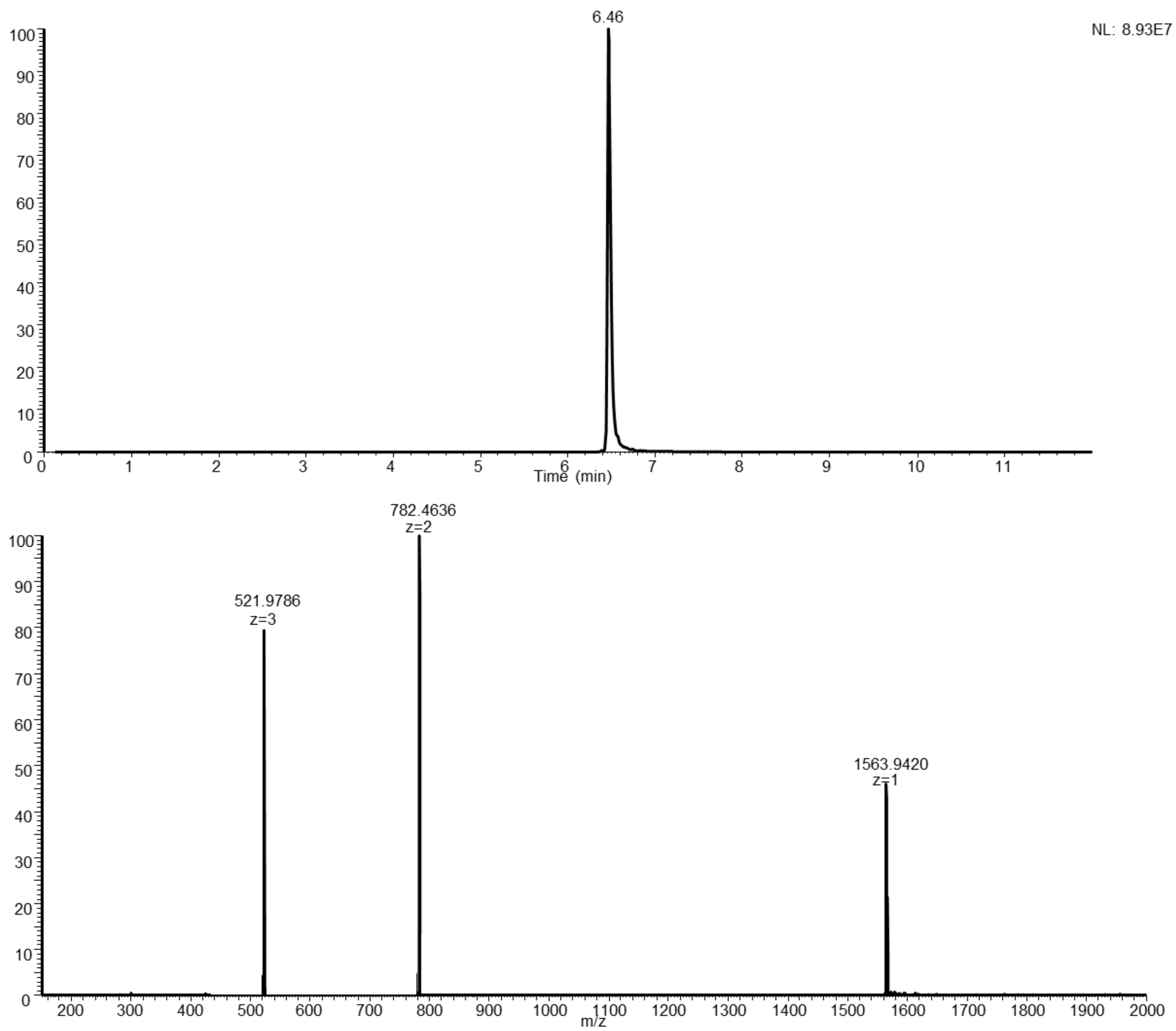

Figure S43. HPLC-HESI-HRMS data of marinocyclin E (**5**). Top: extracted ion chromatogram *m/z* 1563.9220, bottom: MS data of marinocyclin E (**5**) (*m/z* 1563.9220 $[M+H]^+$).

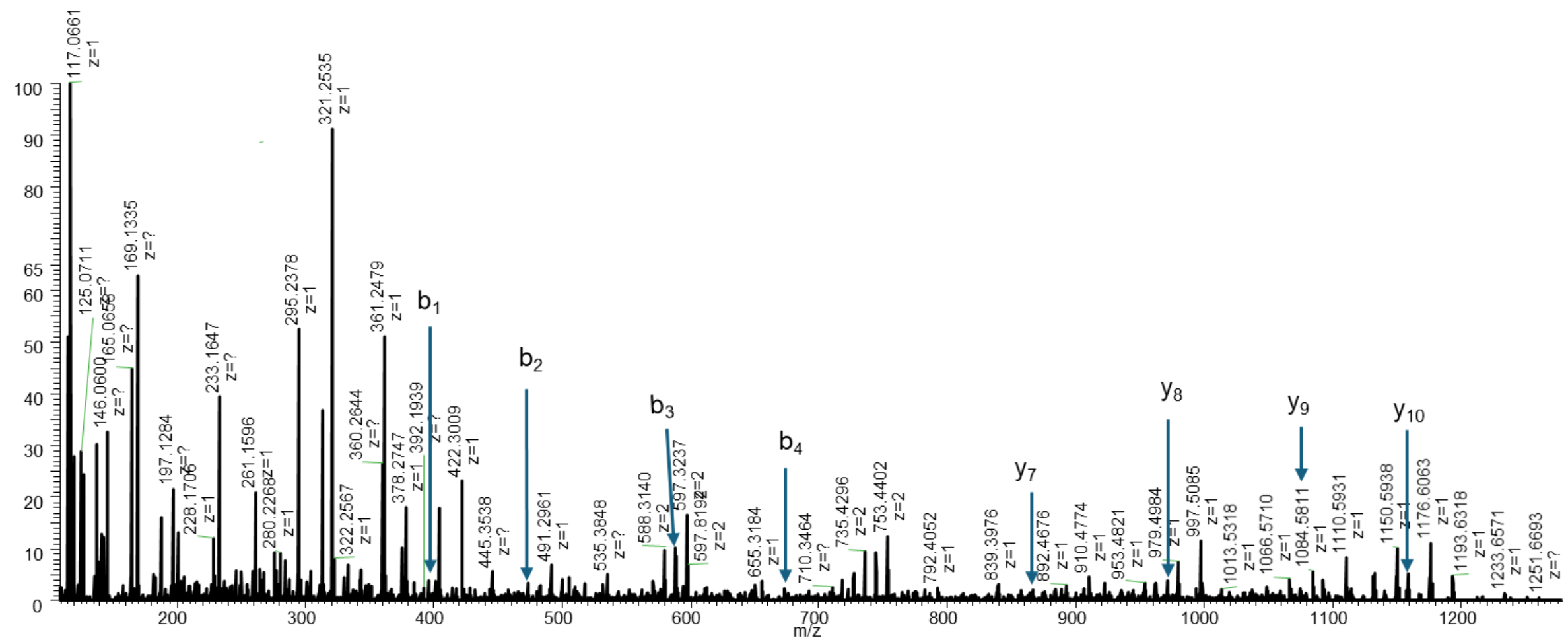

Figure S44. HESI-HRMS[2] data of marinocyclin E (**5**) (*m/z* 782.4591 $[M+H]^{2+}$).

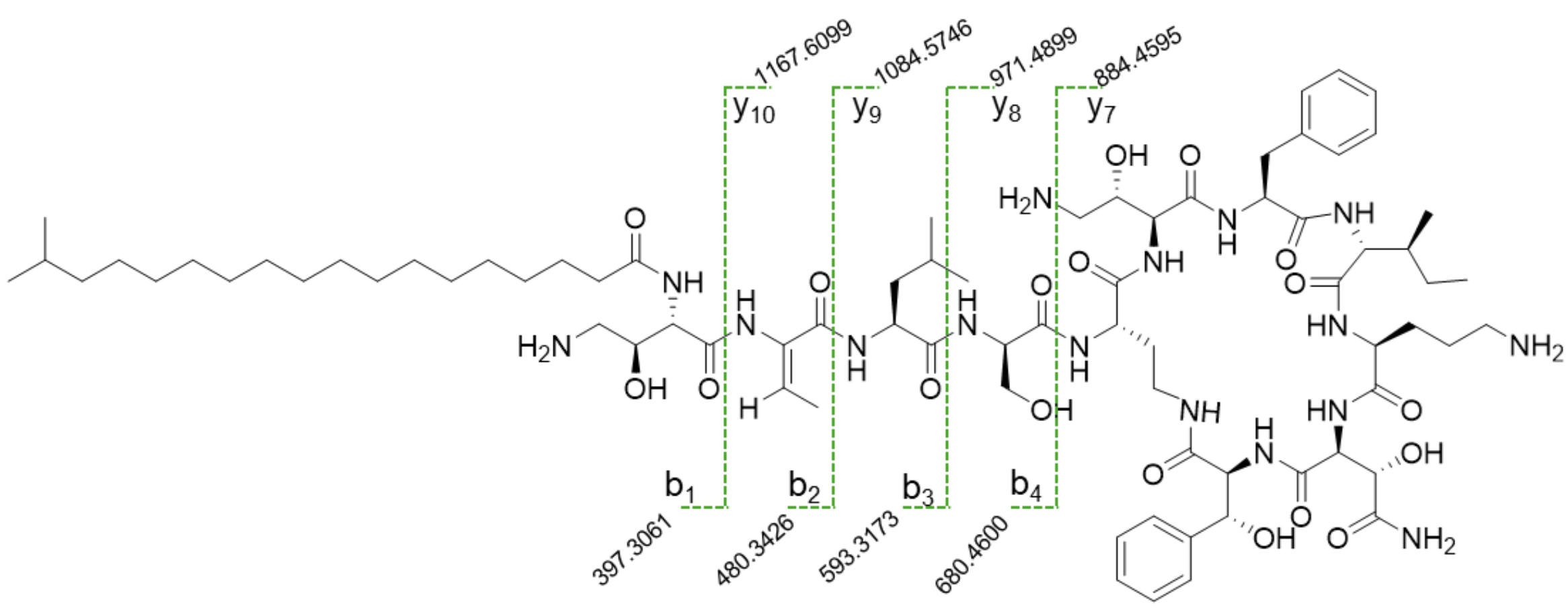

Figure S45. MS[2] fragmentation of marinocyclin E (**5**).

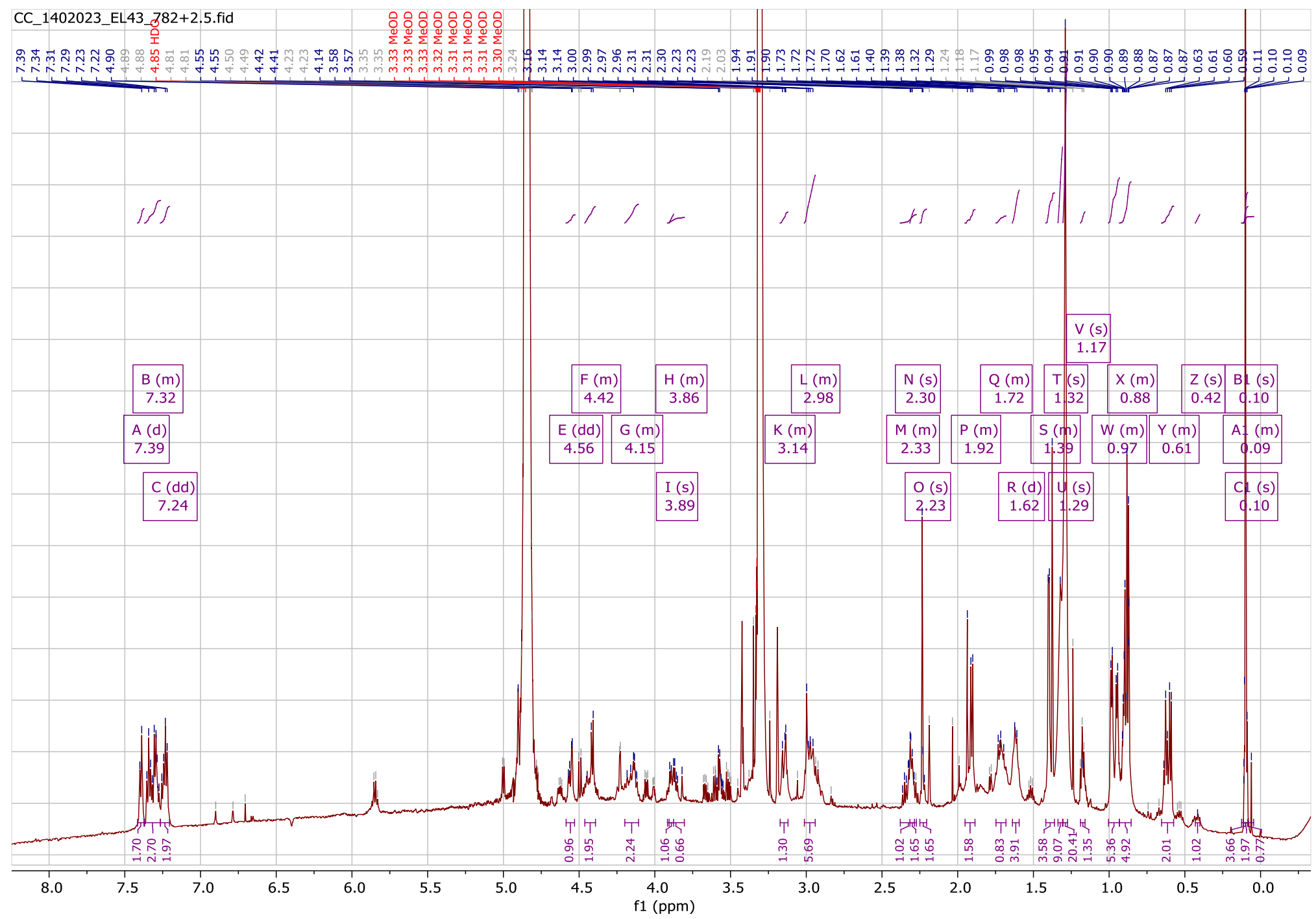

Figure S46. ^{1}H NMR spectrum of marinocyclin E (**5**) in methanol-d_4 (^{1}H 600 MHz).

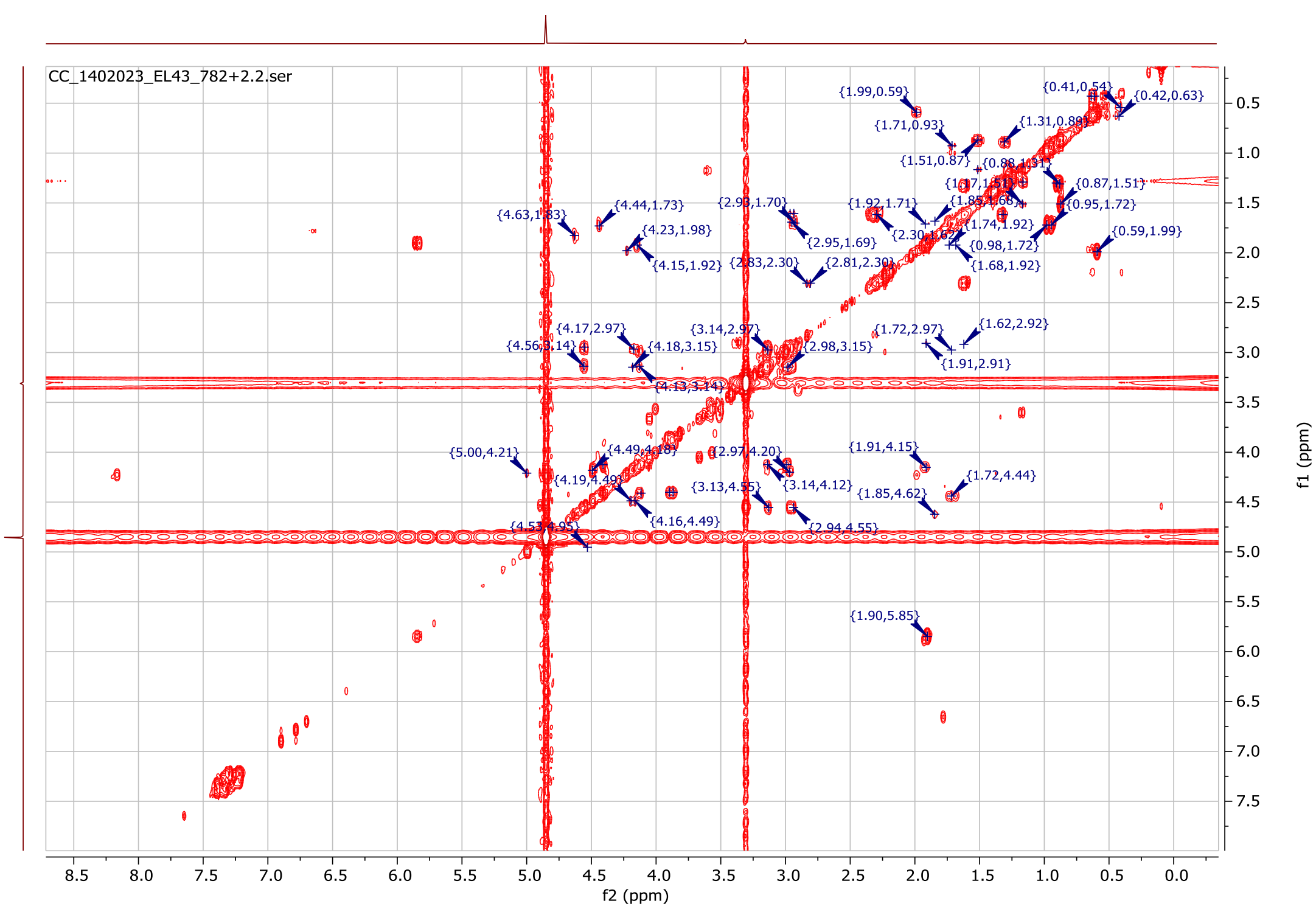

Figure S47. COSY spectrum of marinocyclin E (**5**) in methanol-d_4 (^{1}H 600 MHz).

.

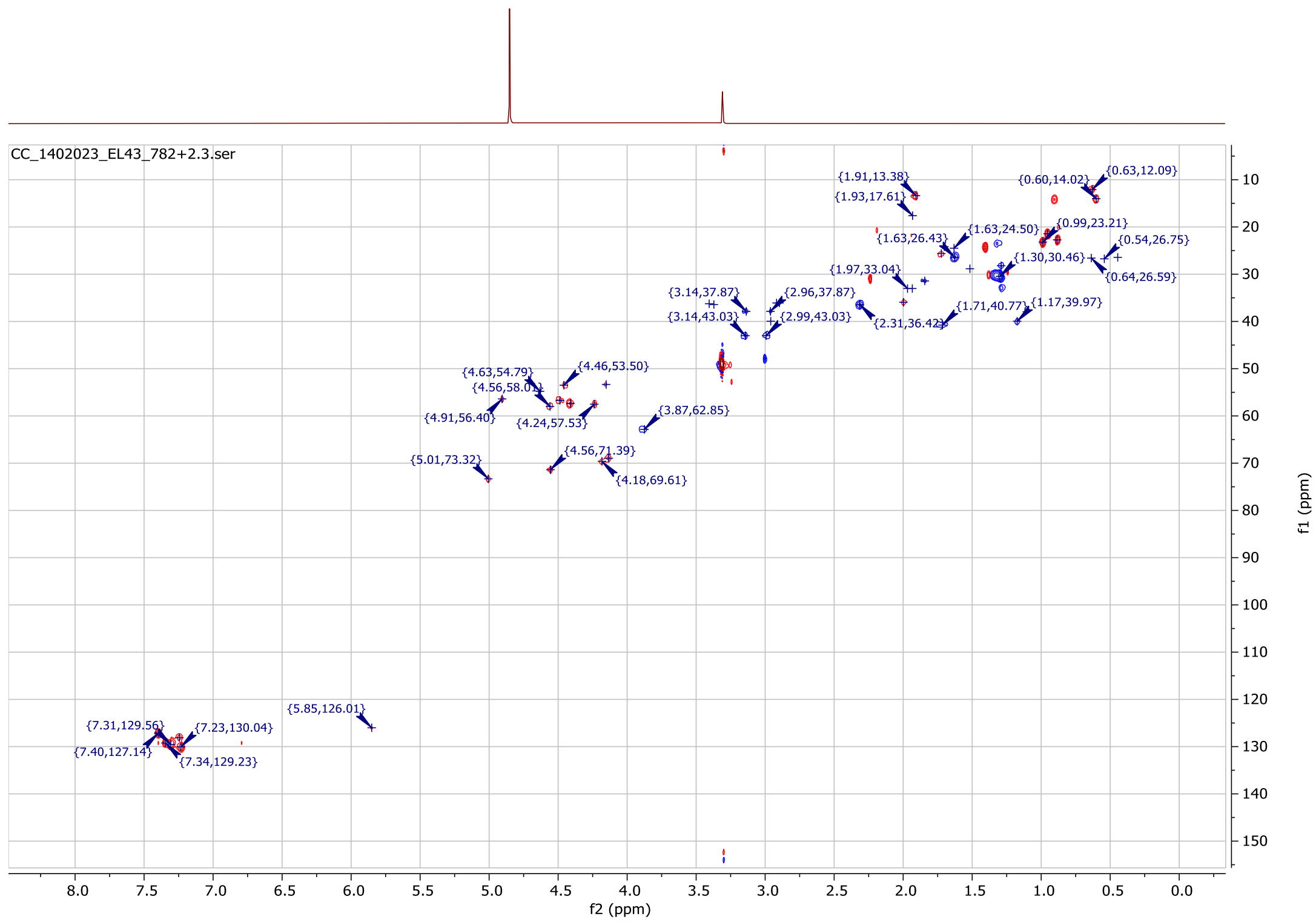

Figure S48. HSQC spectrum of marinocyclin E (**5**) in methanol-d4 (^{1}H 600 MHz, ^{13}C 150 MHz).

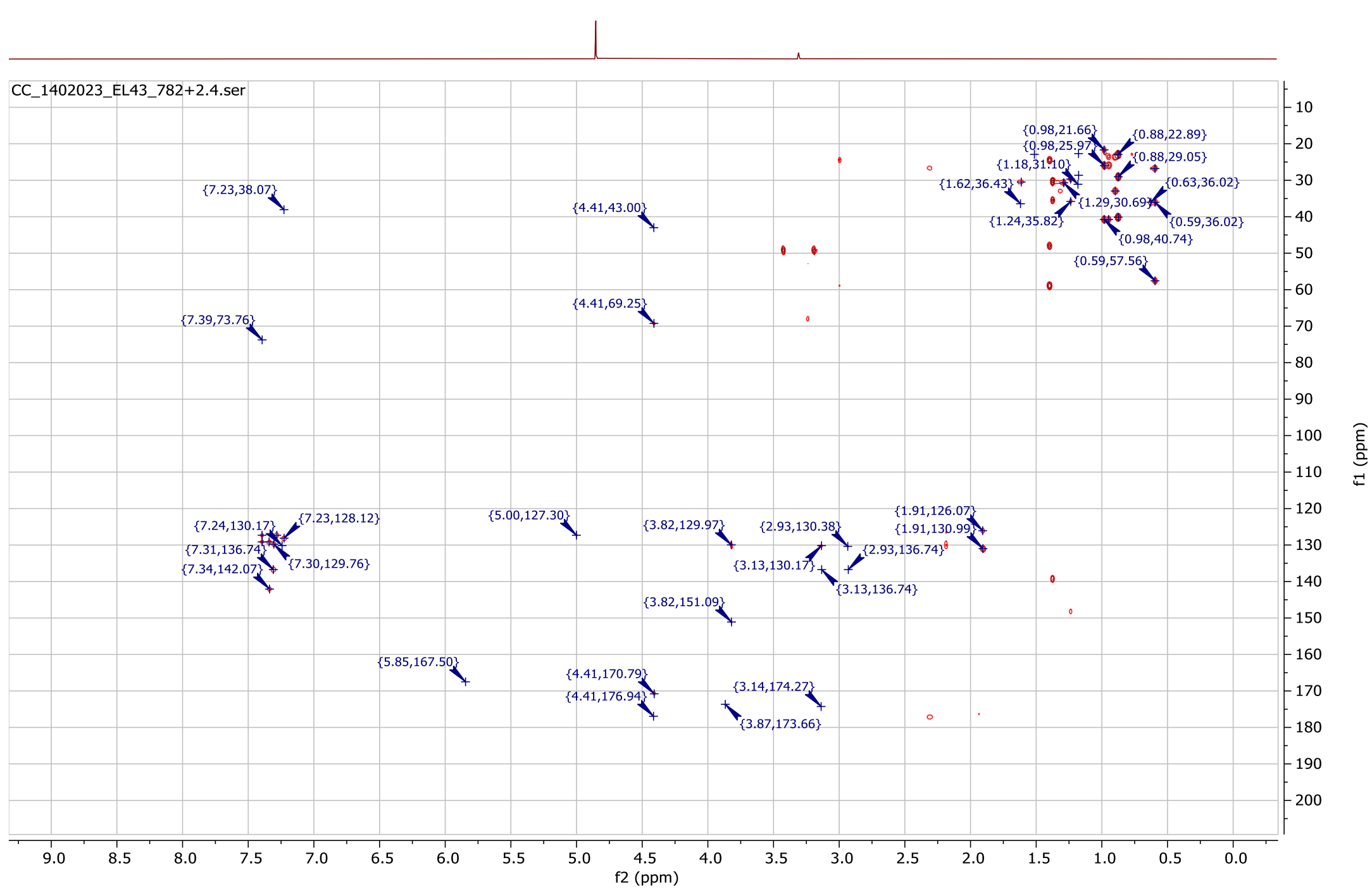

Figure S49. HMBC spectrum of marinocyclin E (**5**) in methanol-d4 (^{1}H 600 MHz, ^{13}C 150 MHz).

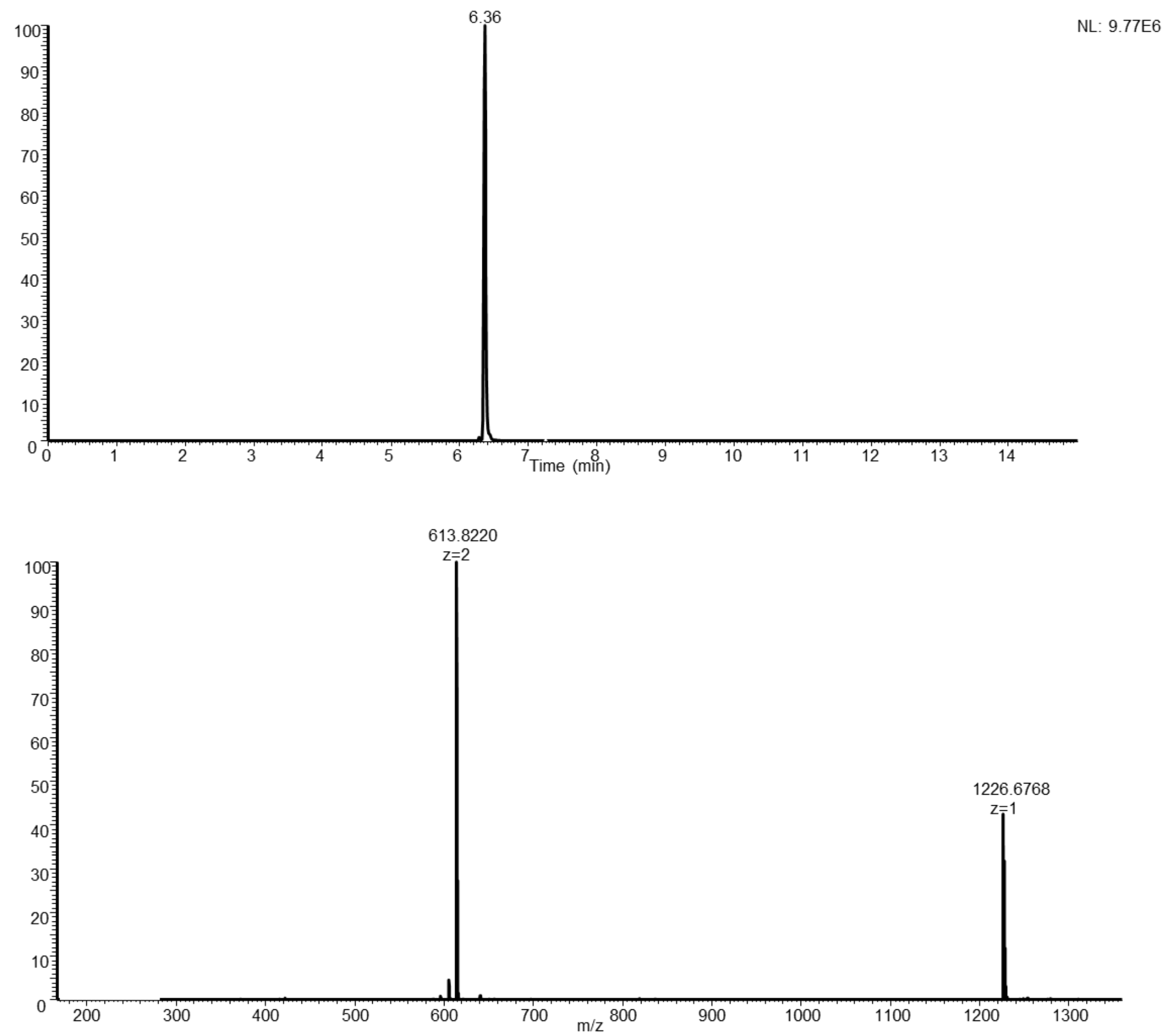

Figure S50. HPLC-HESI-HRMS data of marinocyclin F (**6**). Top: extracted ion chromatogram *m/z* 1226.6368, bottom: MS data of marinocyclin F (**6**) (*m/z* 1226.6368 $[M+H]^+$).

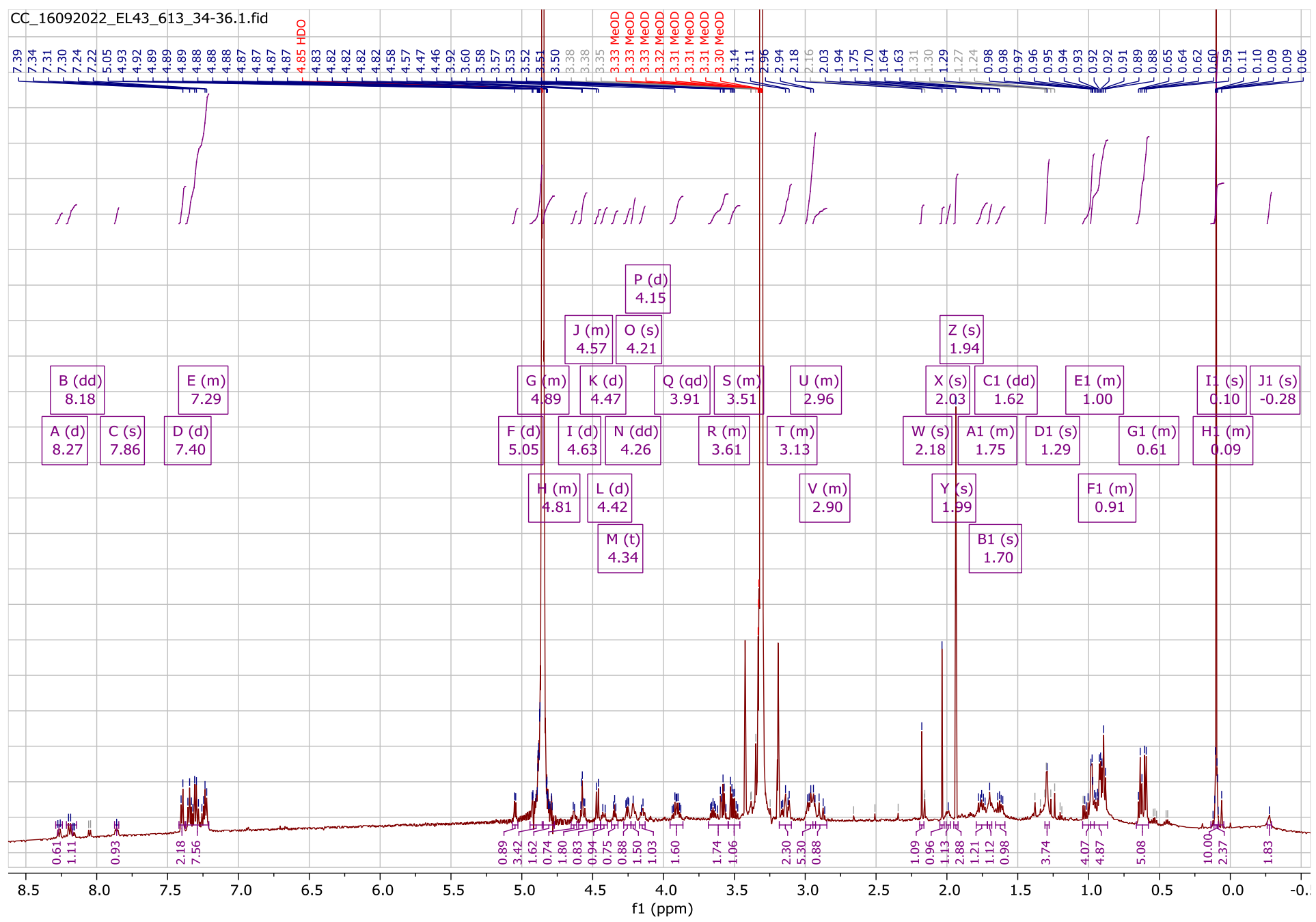

Figure S51. ^{1}H NMR spectrum of marinocyclin F (**6**) in methanol-d$_4$ (^{1}H 600 MHz).

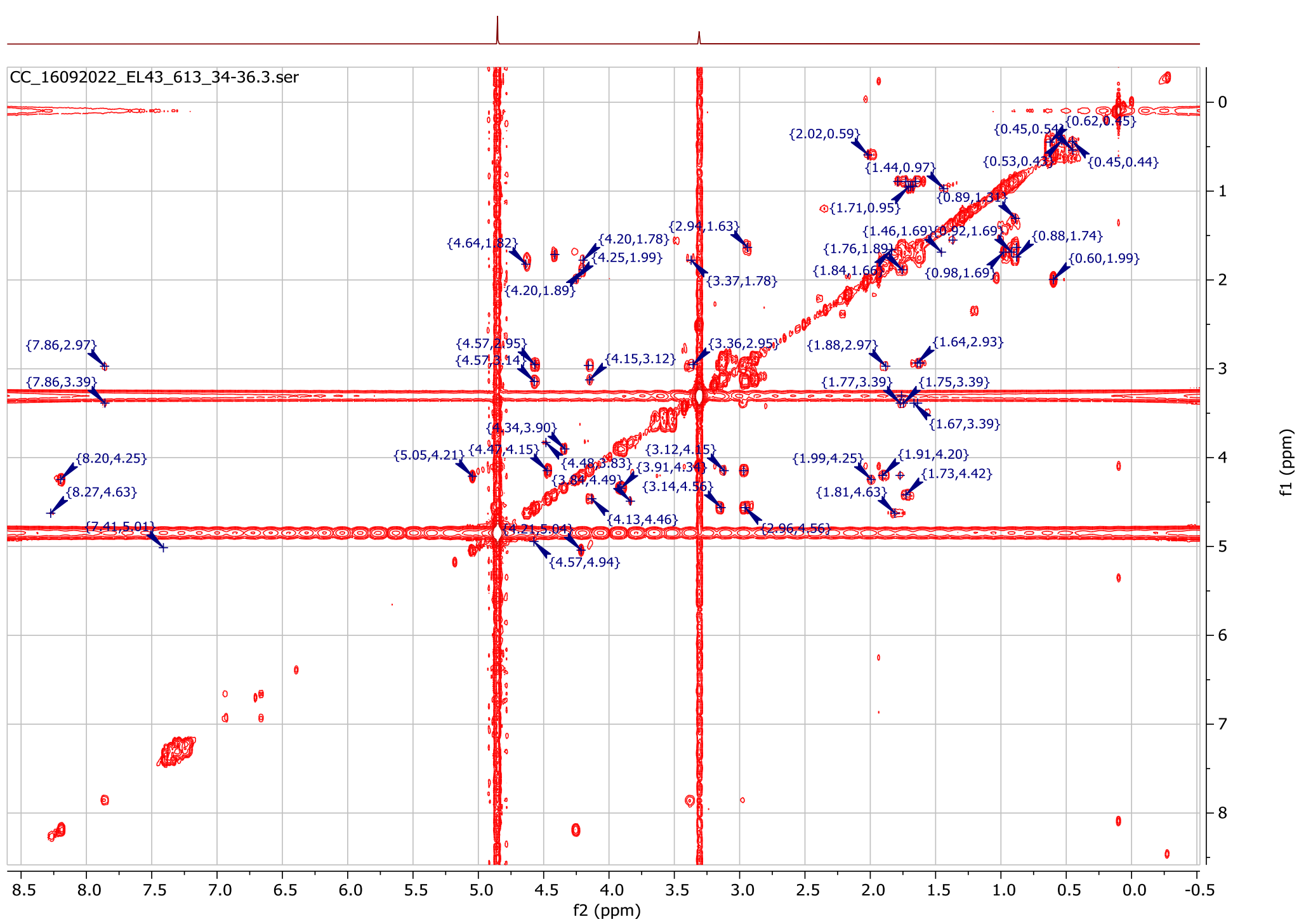

Figure S52. COSY spectrum of marinocyclin F (**6**) in methanol-d$_4$ (^{1}H 600 MHz).

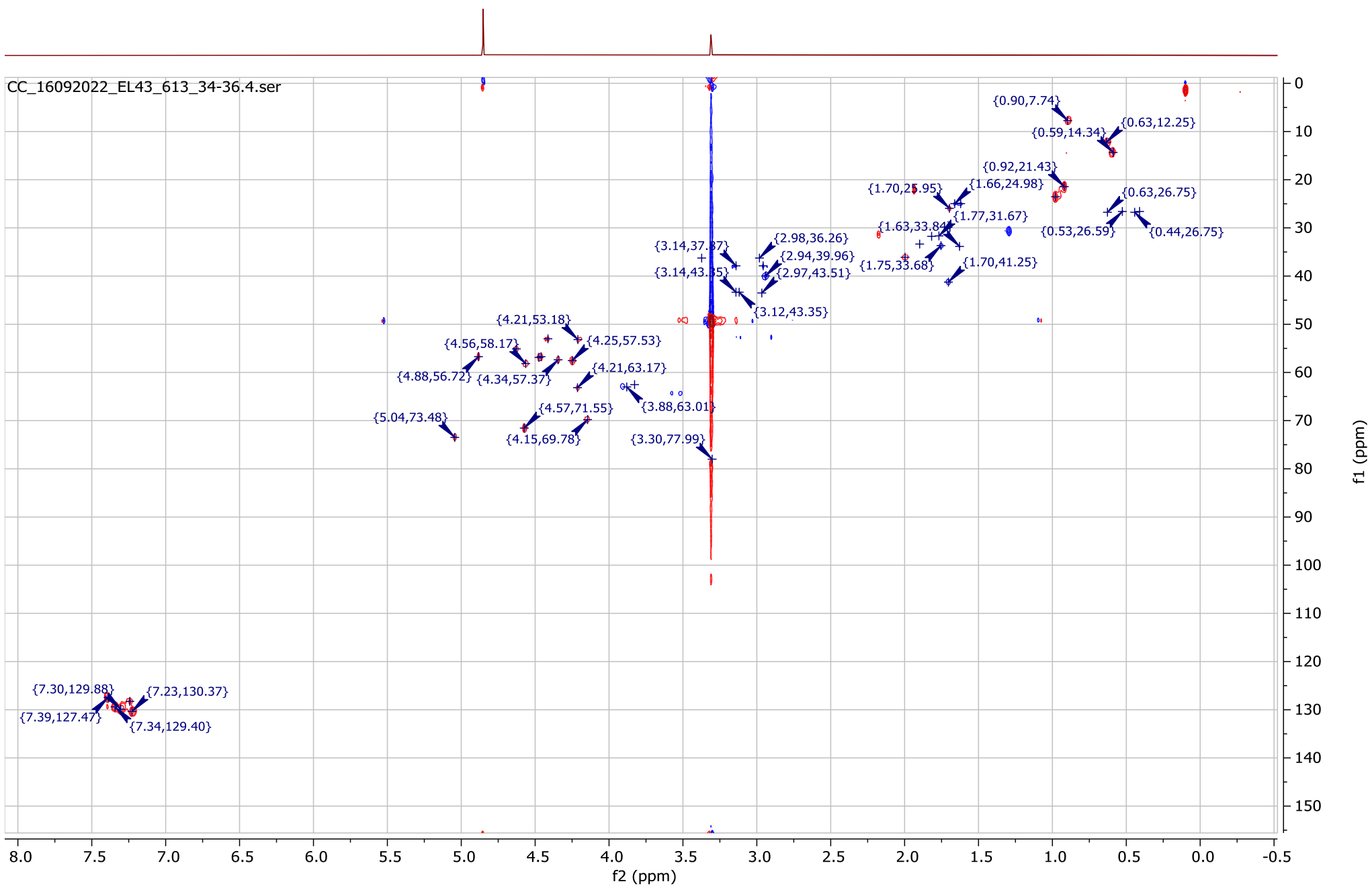

Figure S53. HSQC spectrum of marinocyclin F (**6**) in methanol-d4 (^{1}H 600 MHz, ^{13}C 150 MHz).

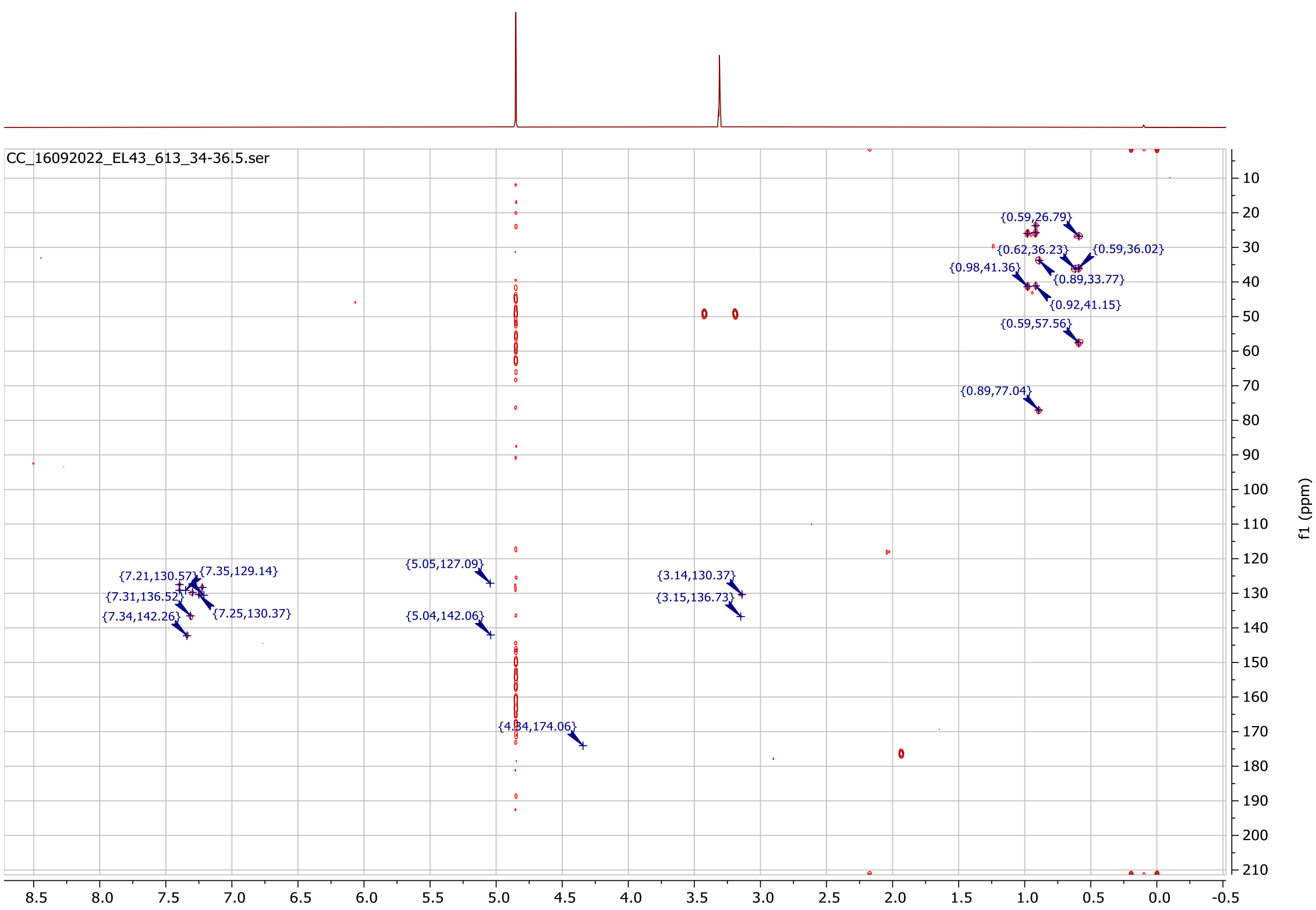

Figure S54. HMBC spectrum of marinocyclin F (**6**) in methanol-d4 (^{1}H 600 MHz, ^{13}C 150 MHz).

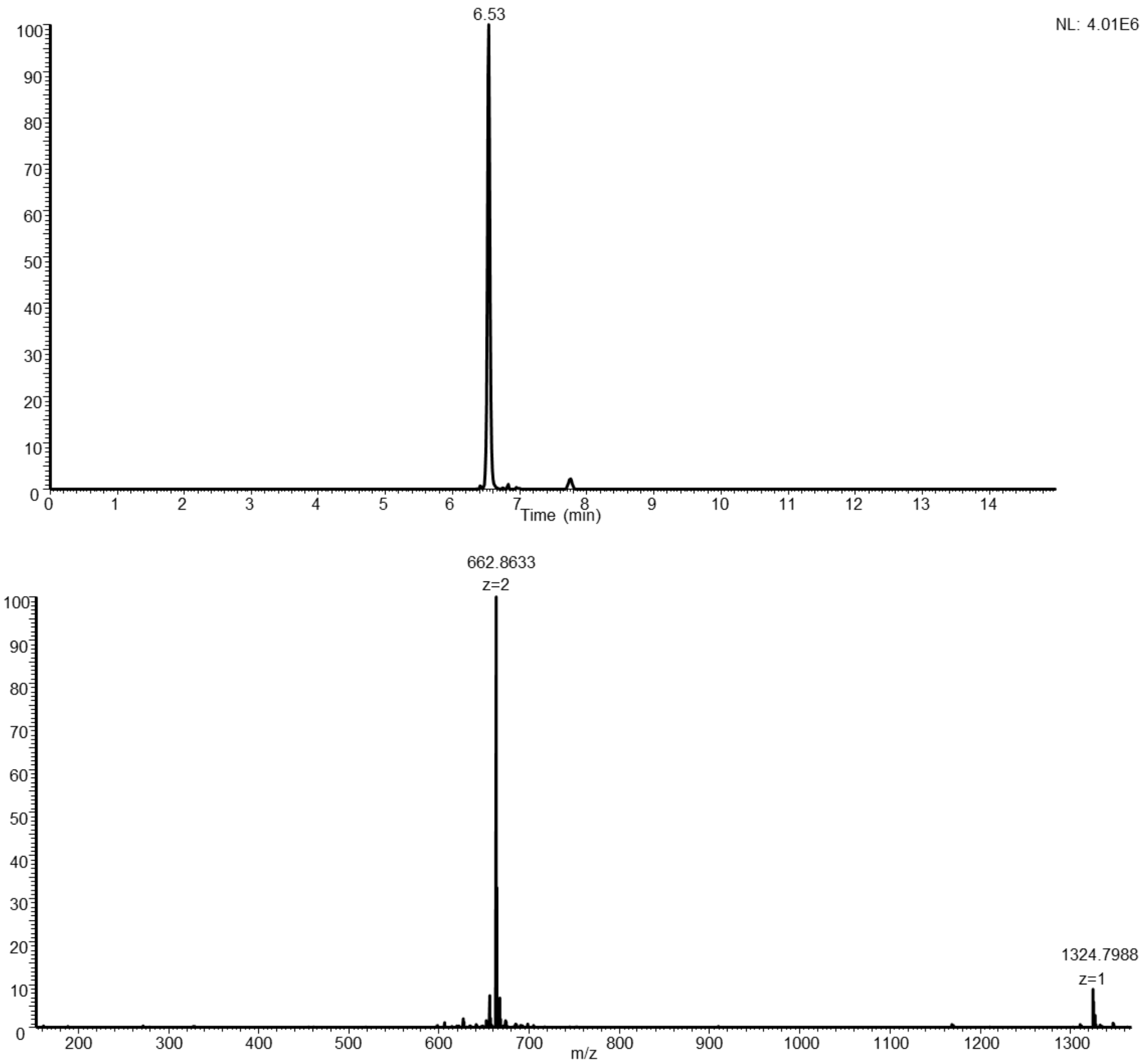

Figure S55. HPLC-HESI-HRMS data of marinocyclin G (**7**). Top: total ion chromatogram *m/z* 1324.7191, bottom: MS data of marinocyclin G (**7**) (*m/z* 1324.7191 $[M+H]^+$).

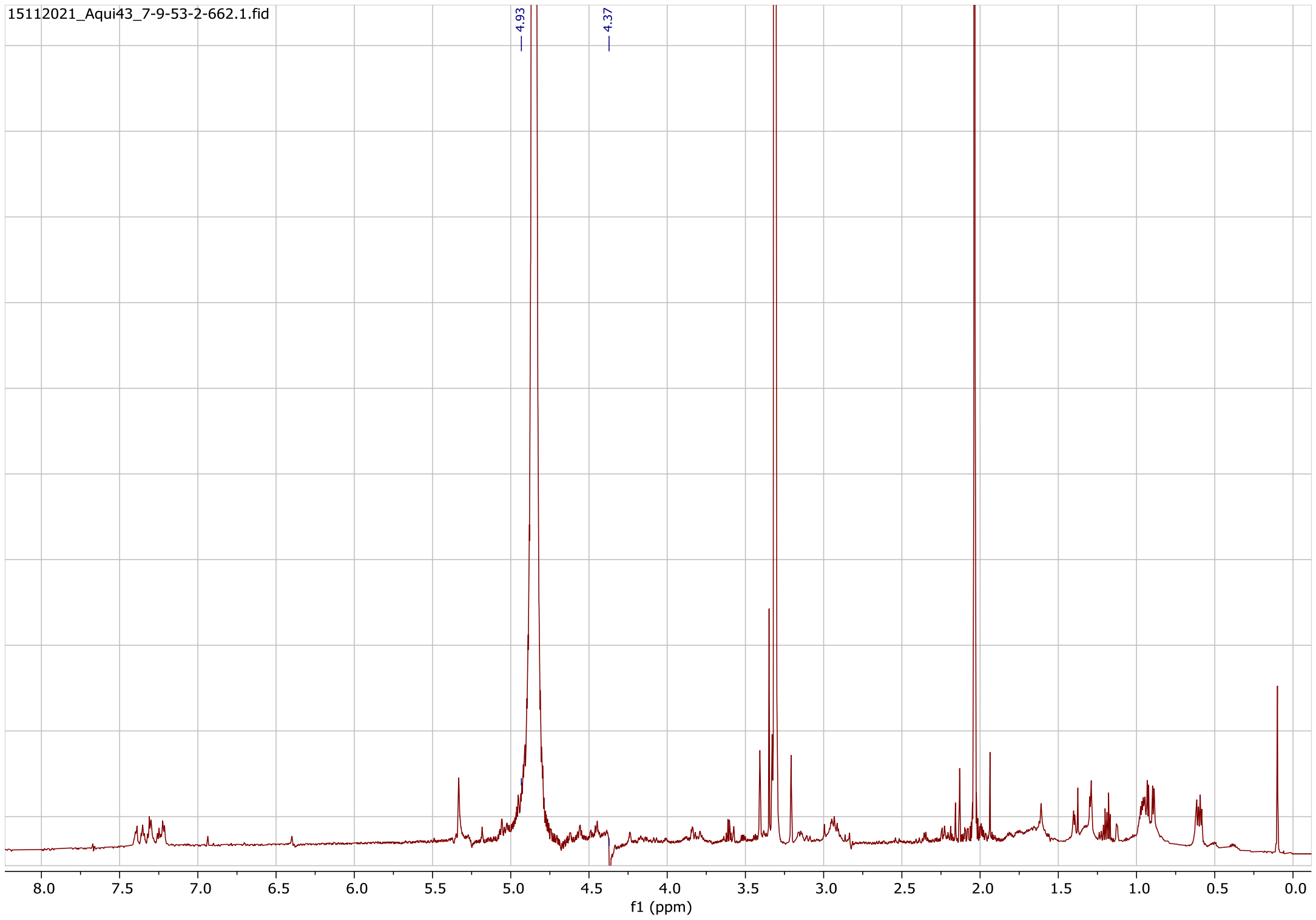

Figure S56. ^{1}H NMR spectrum of marinocyclin G (**7**) in methanol-d$_4$ (^{1}H 600 MHz).

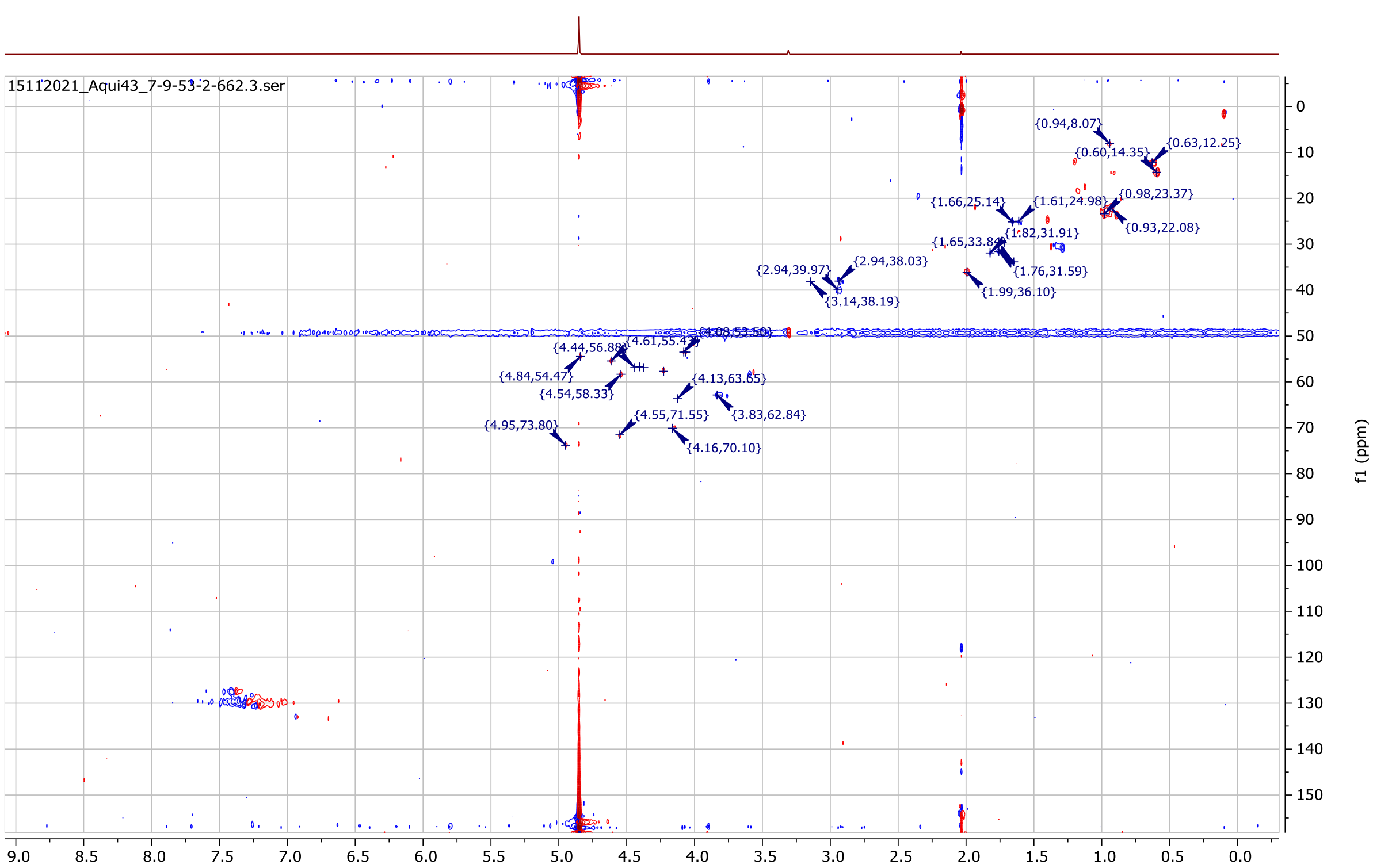

Figure S57. HSQC spectrum of marinocyclin G (**7**) in methanol-d$_4$ (^{1}H 600 MHz, ^{13}C 150 MHz).

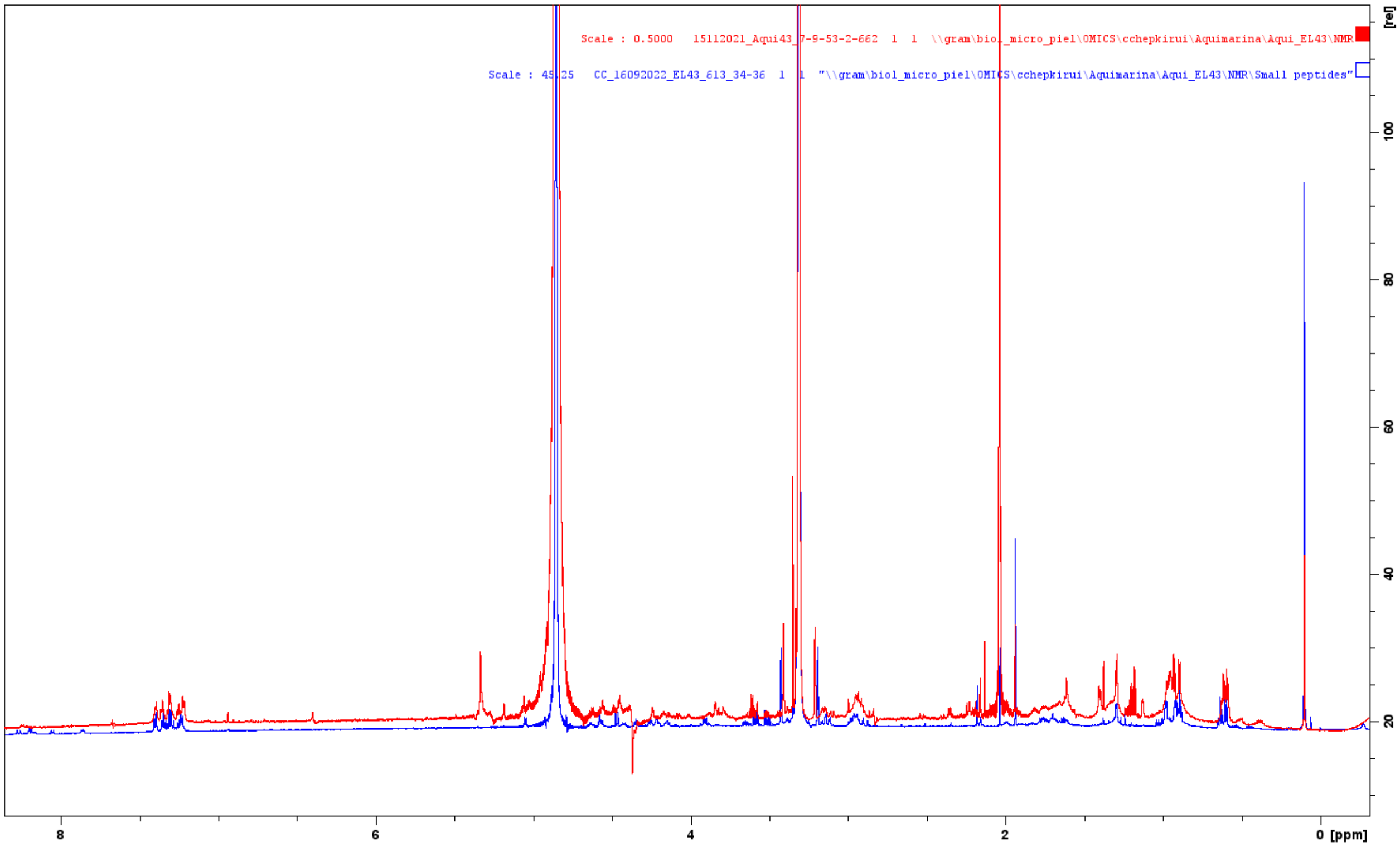

Figure S58. Overlayed ^1H NMR spectra of marinocyclin F (**6**) and marinocyclin G (**7**) in methanol-d_4 (^1H 600 MHz).

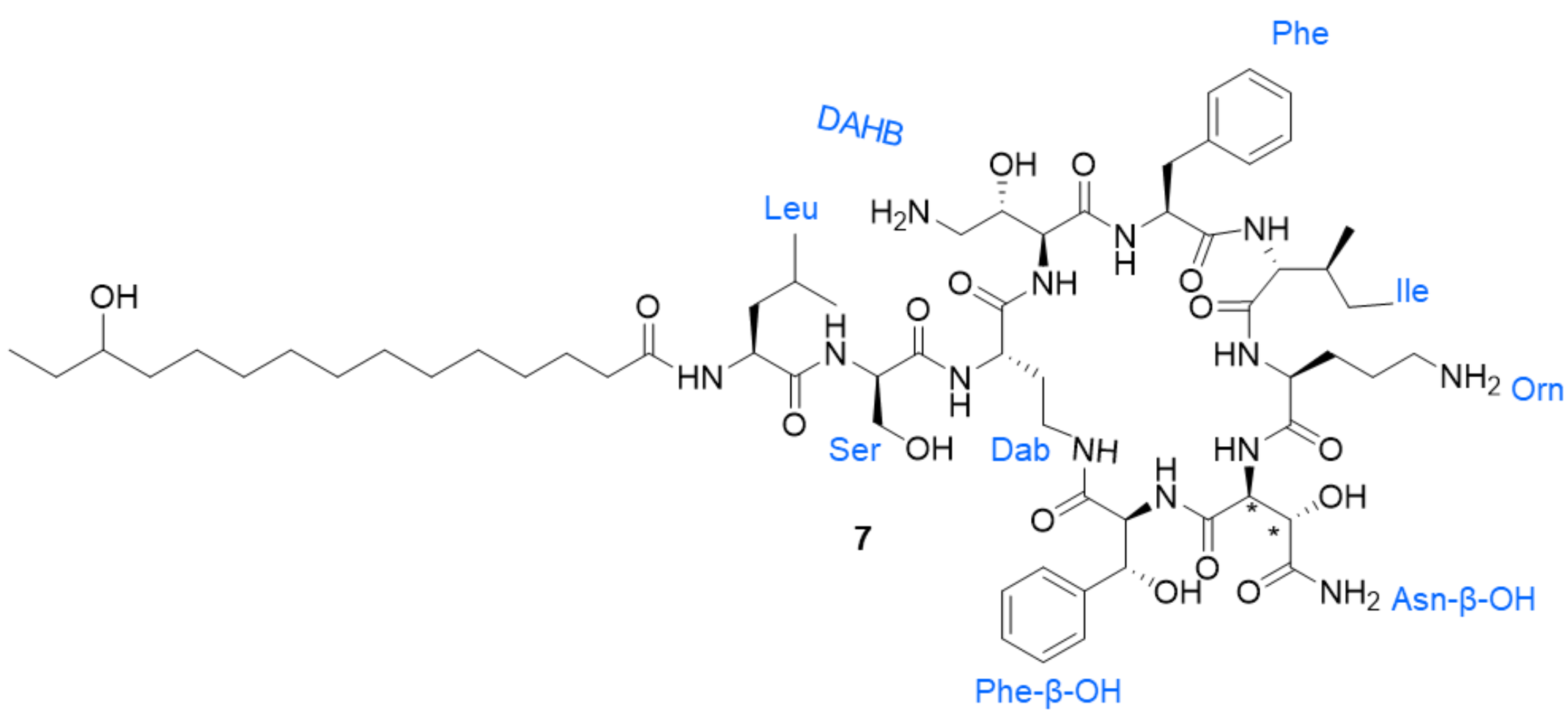

7

Figure S59. Proposed structure of marinocyclin G (**7**).

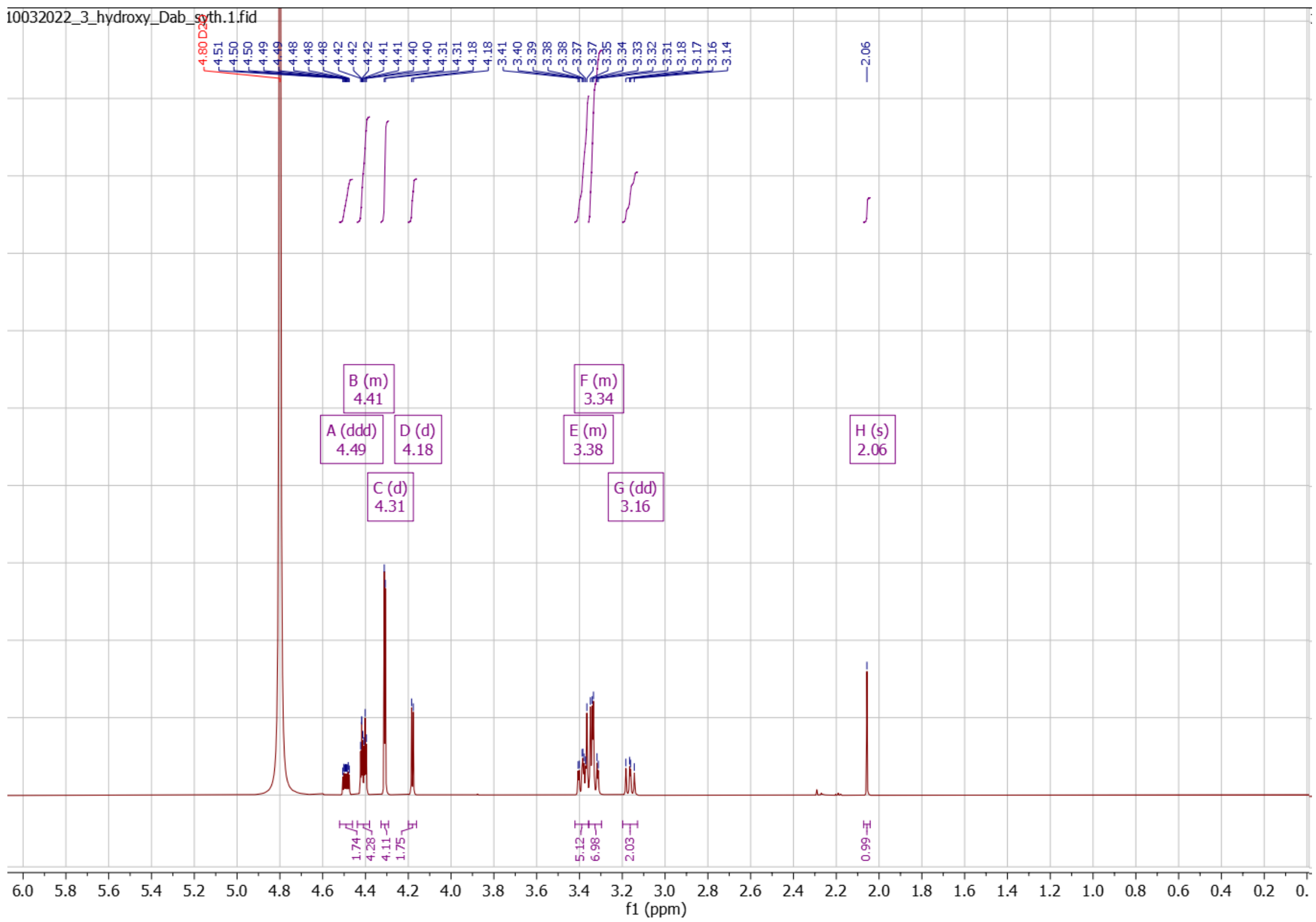

Figure S60. ^{1}H NMR spectrum of (2*S*,3*S*) and (2*R*,3*S*)-2,4-diamino-3-hydroxybutanoic acid diastereomeric mixture in D_2O (^{1}H 600 MHz).

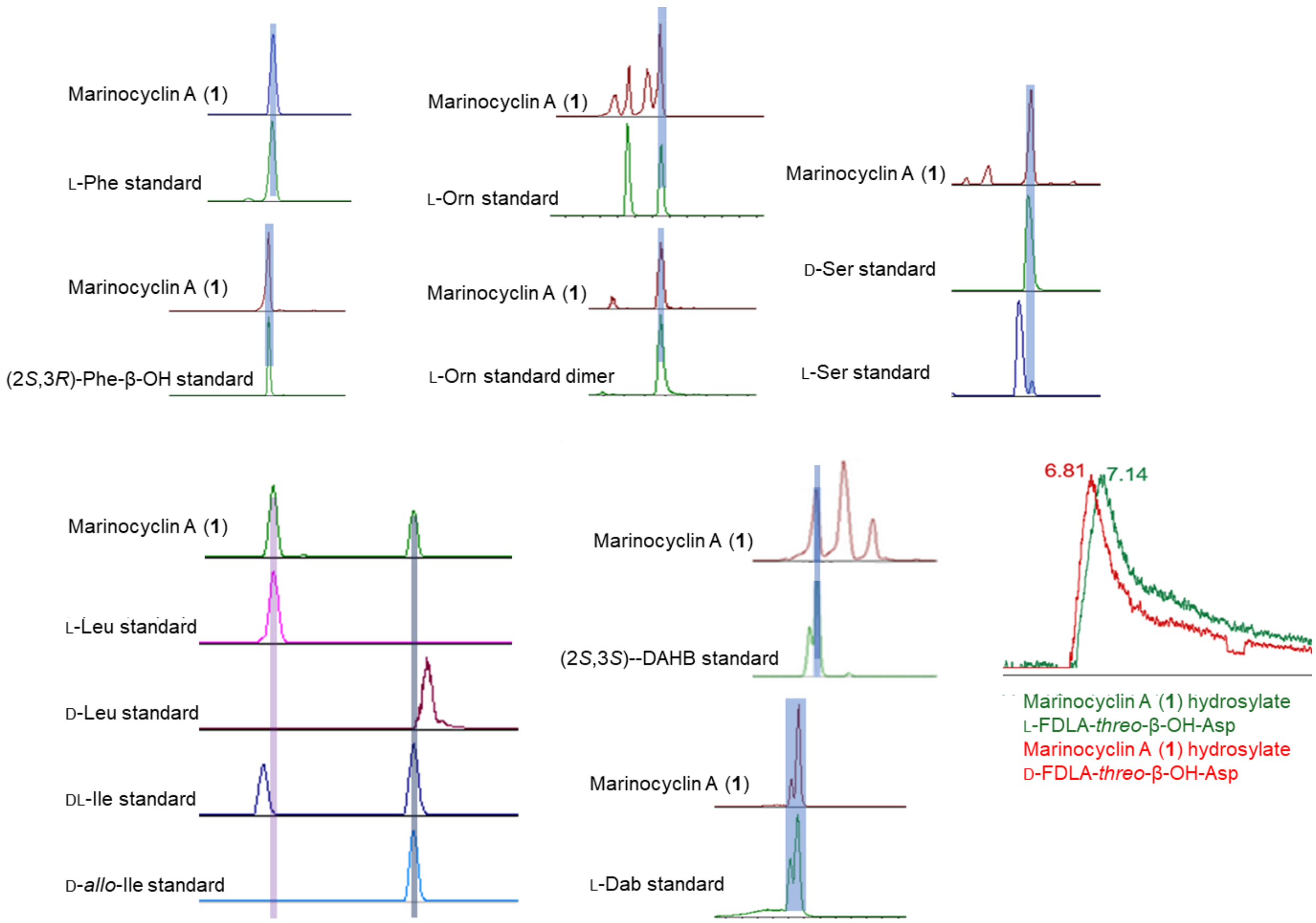

Figure S61. Marfey's analysis of marinocyclin A (**1**)**.** Comparison of Extracted Ion Chromatograms (EICs) of FDVA-derivatized marinocyclin A (**1**) hydrolysate to the EICs of the D- and L-amino acid standards.

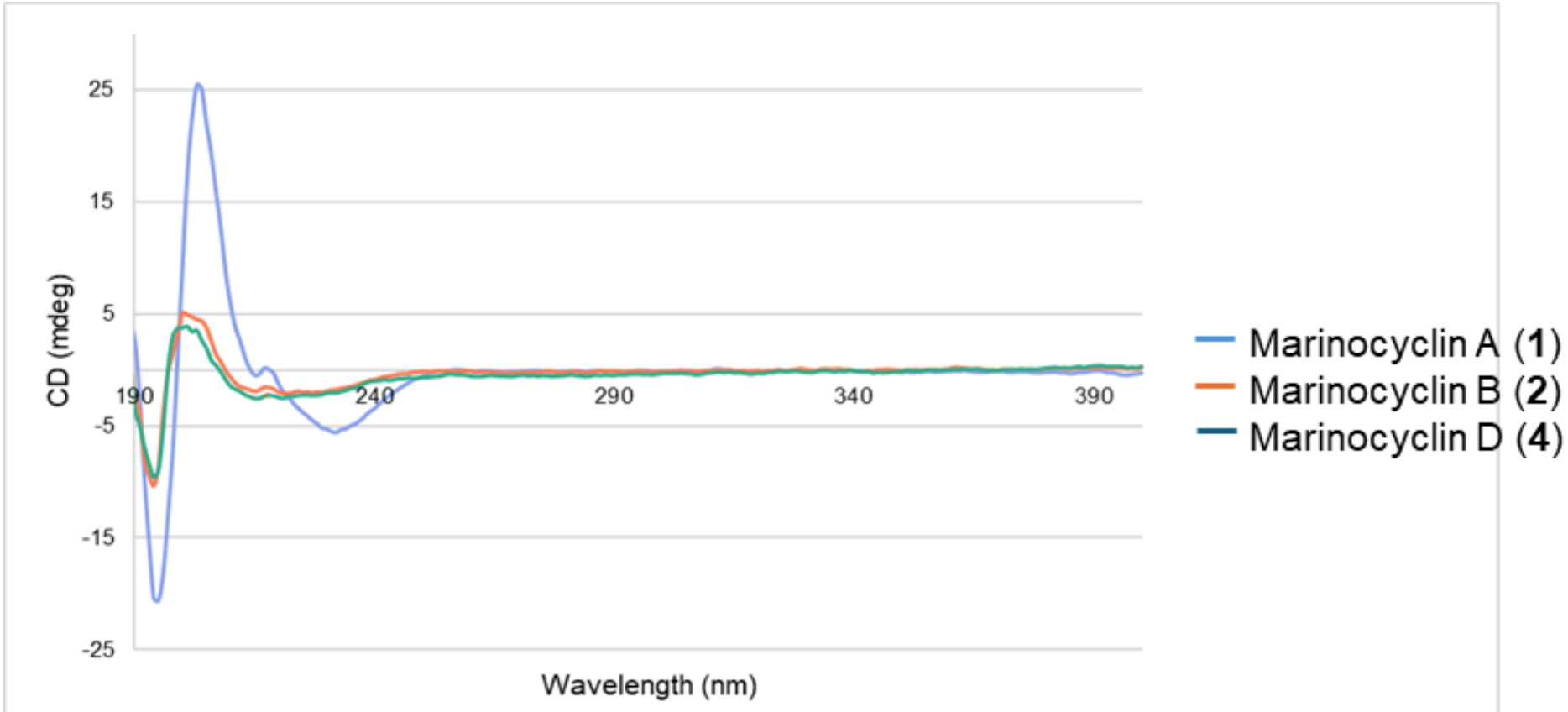

Figure S62. Electronic circular dichroism (ECD) spectra of marinocyclins A (**1**), B (**2**), and D (**4**) in ethanol.

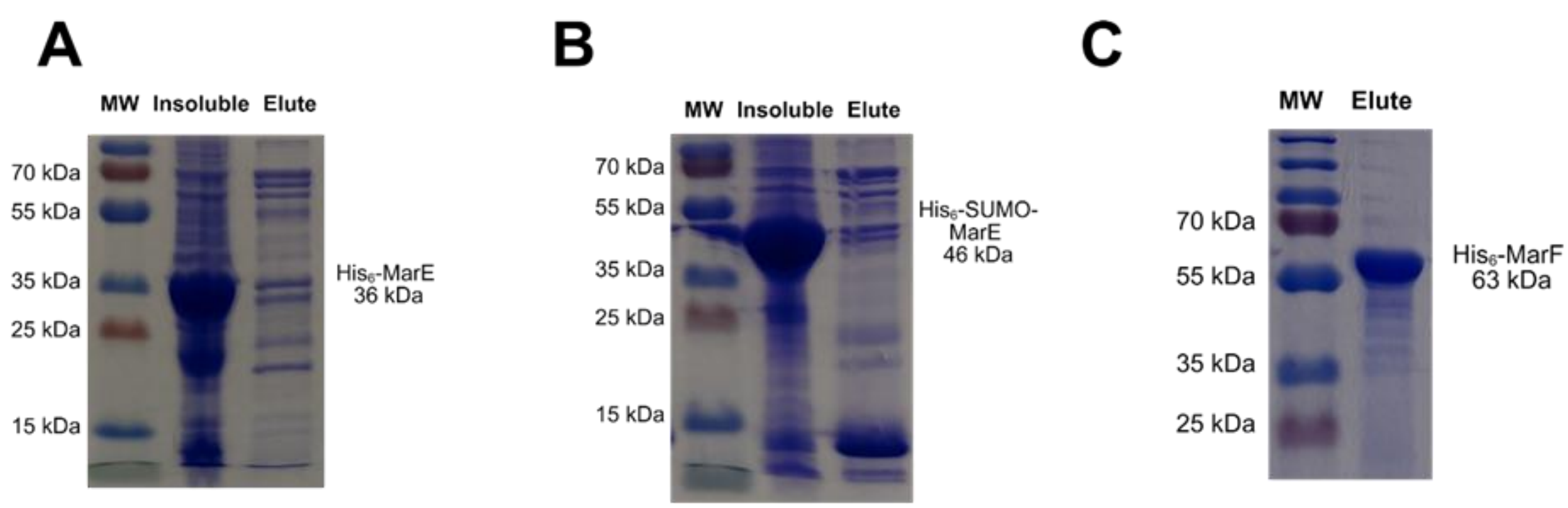

Figure S63. Coomassie-stained sodium dodecylsulfate (SDS) polyacrylamide gel of heterologously produced and purified putative marinocyclin amino acid hydroxylases: **A,** His$_6$-MarE; **B,** His$_6$-SUMO-MarE, and **C,** His$_6$-MarF. MW = molecular weight marker. Insoluble = insoluble fraction pelleted by centrifugation. Elute = elution fraction of the desired protein from Ni-NTA column. His$_6$-SUMO-MarE was mostly insoluble and appeared to be truncated. Therefore, His$_6$-MarE was used for all enzyme assays, though it too was mostly obtained in the insoluble fraction. His$_6$-MarF was highly soluble and overexpressed very well.

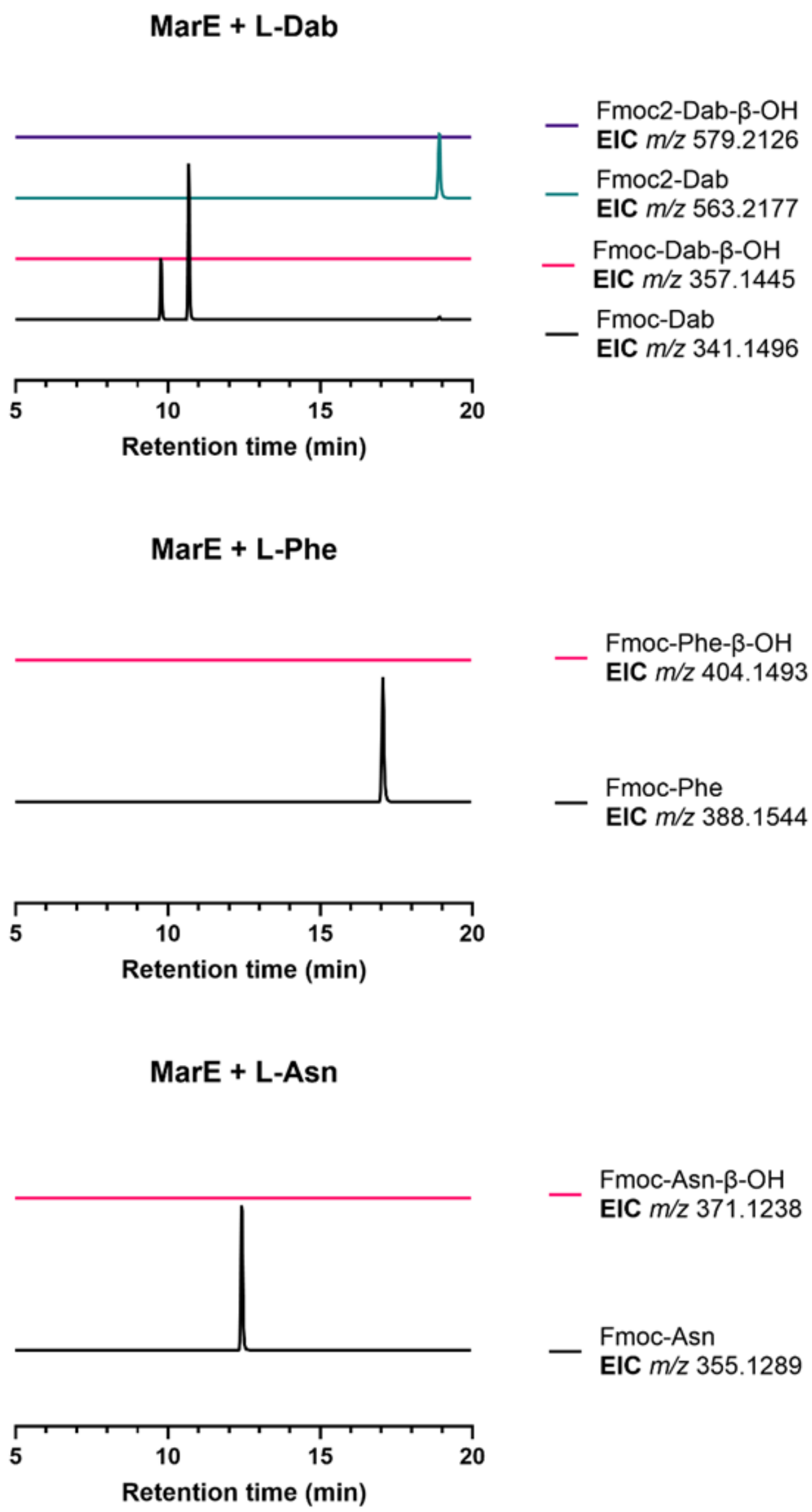

Figure S64. β-Hydroxylation assay of purified Fe^{II}/α-ketoglutarate-dependent enzyme MarE with free amino acid substrates. Panels show extracted ion chromatograms (EICs) of assay reactions for masses of Fmoc-protected amino acid substrates and their expected β-hydroxylated products. For Dab, EICs are provided for both the mono-protected and di-protected ($Fmoc_2$) masses of substrate and expected products.

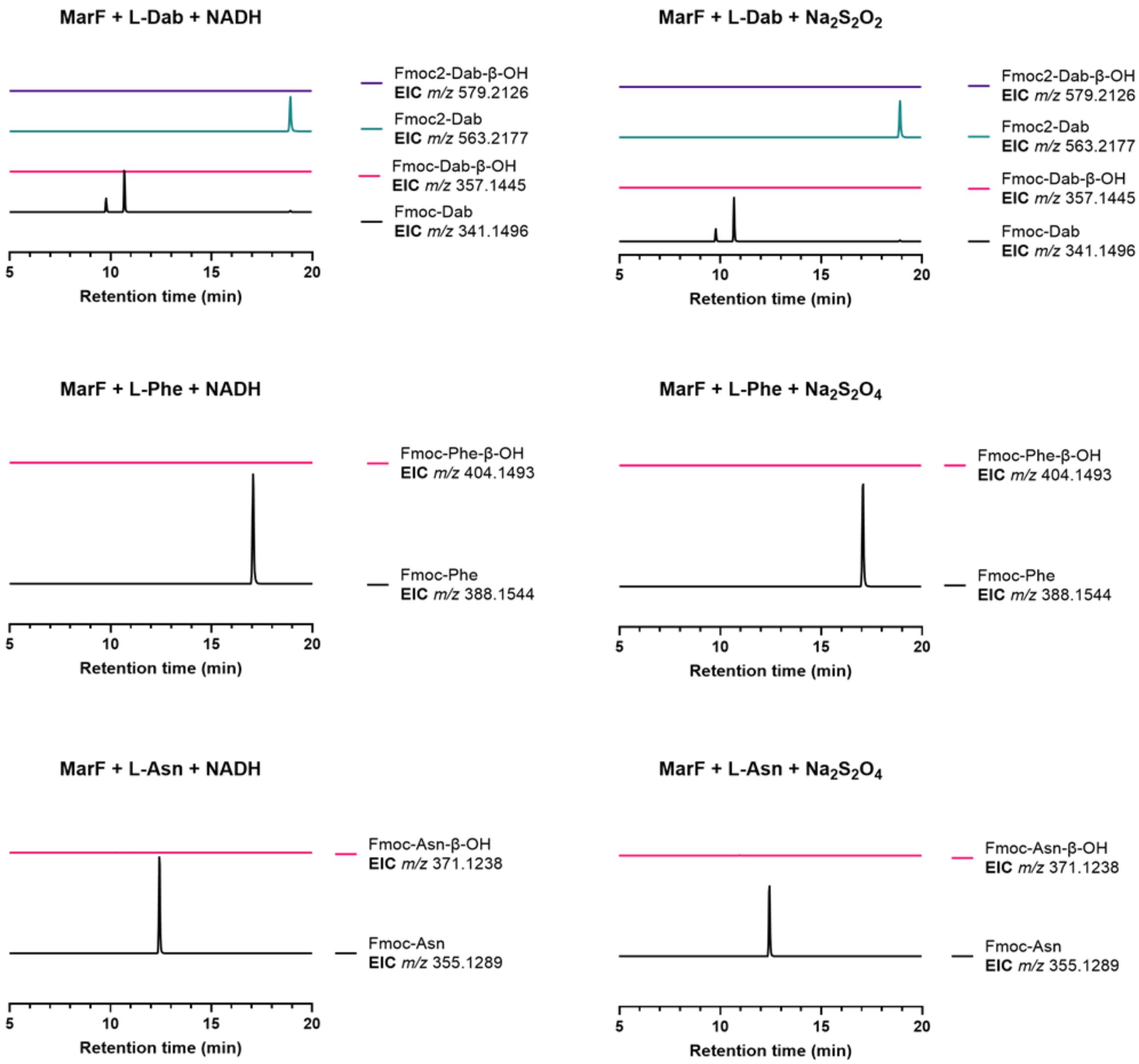

Figure S65. β-Hydroxylation assay of purified non-heme diiron monooxygenase MarE with free amino acid substrates. Right: extracted ion chromatograms of assay reactions for masses of Fmoc-protected amino acid substrates and their expected β-hydroxylated products. Left: reactions performed with NADH and methyl viologen as electron donors, and the right panels show reactions performed with $Na_2S_2O_4$ and methyl viologen as electron donors. For Dab, EICs are provided for both the mono-protected and di-protected (Fmoc2) masses of substrate and expected products.

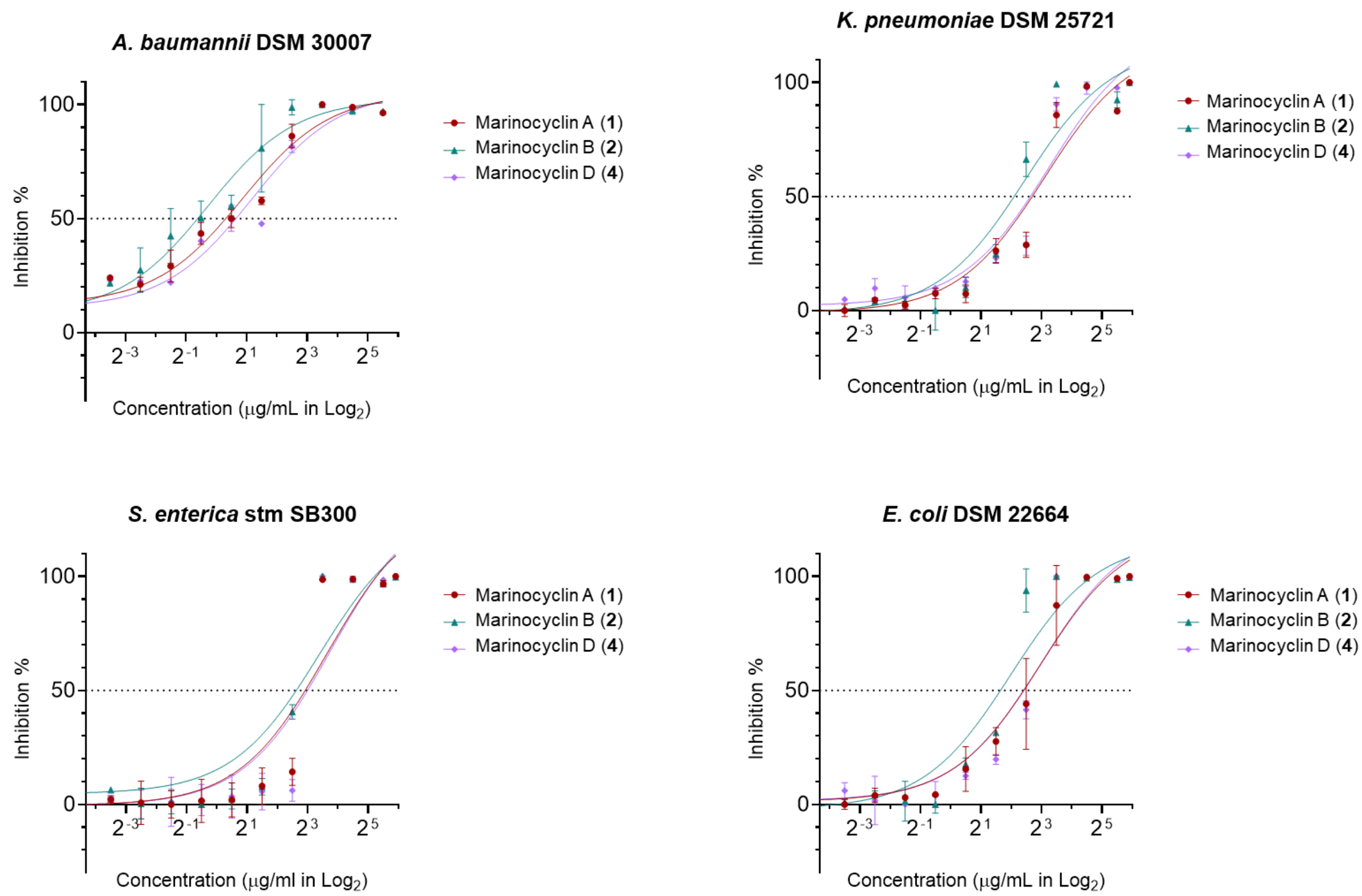

Figure S66. Minimal inhibitory concentration (MIC) determination of marinocyclins against *A. baumannii* DSM 30007, *K. pneumoniae* DSM 25721, *S. enterica* STM SB300, and *E. coli* DSM 22664.

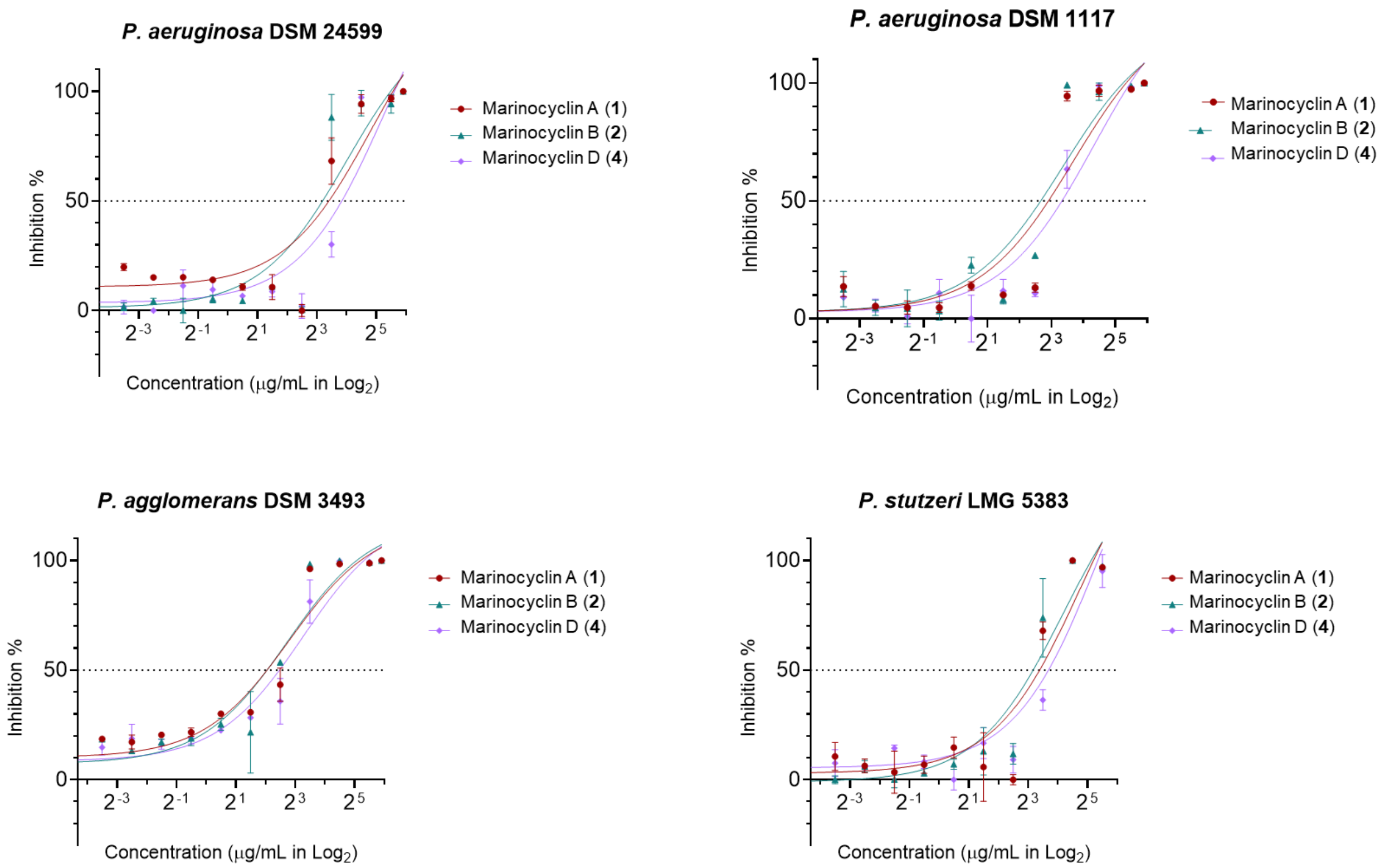

Figure S67. MIC determination of marinocyclins against *P. aeruginosa* DSM 24599, *P. aeruginosa* DSM 1117*, P. agglomerans* DSM 3493, and *P. stutzeri* LMG 5383.

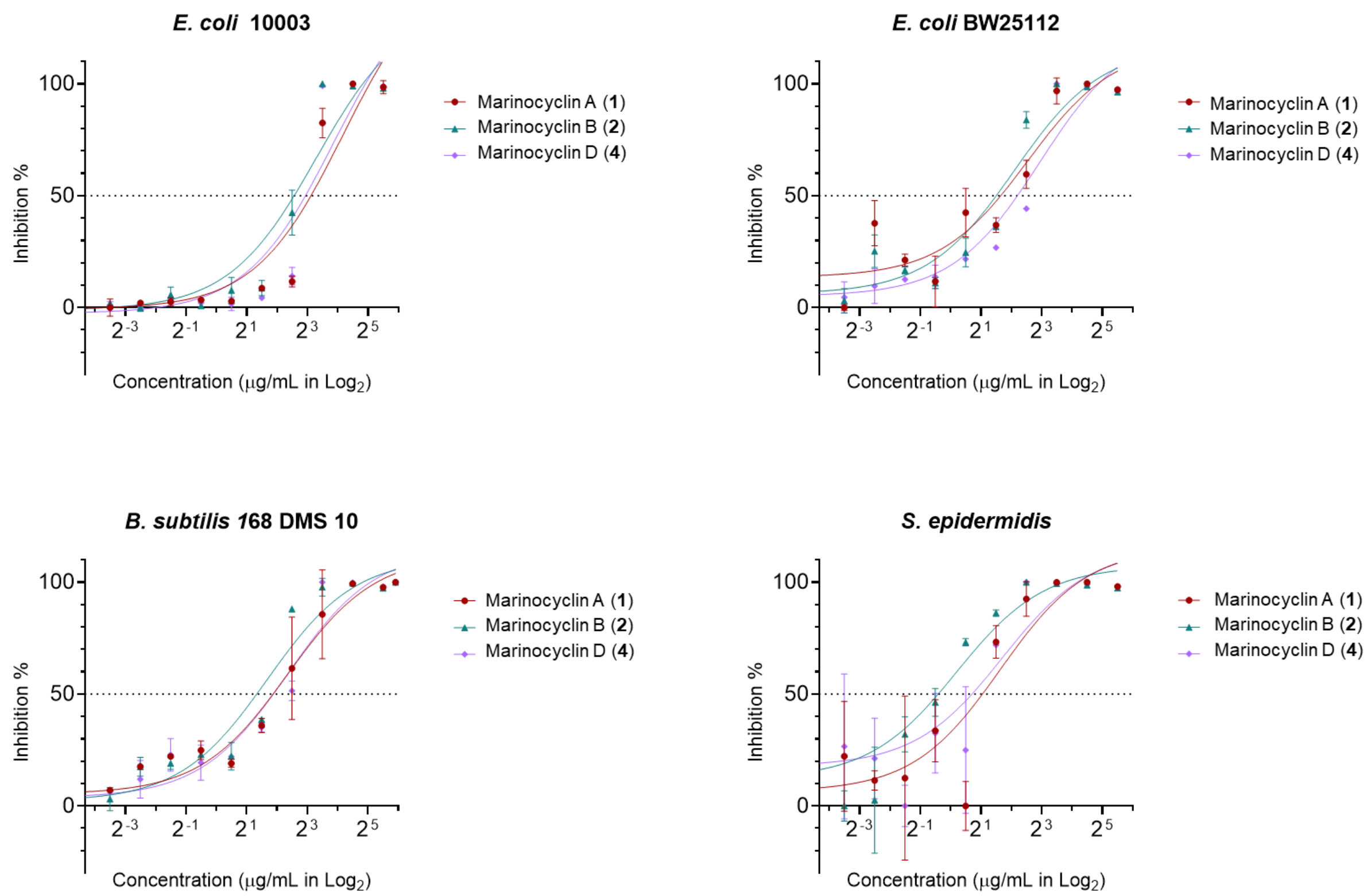

Figure S68. MIC determination of marinocyclins against *E. coli* DSM 1003, *E. coli* BW25112, *B. subtilis* 168 DSM 10, and *S. epidermidis* ATCC 12228.

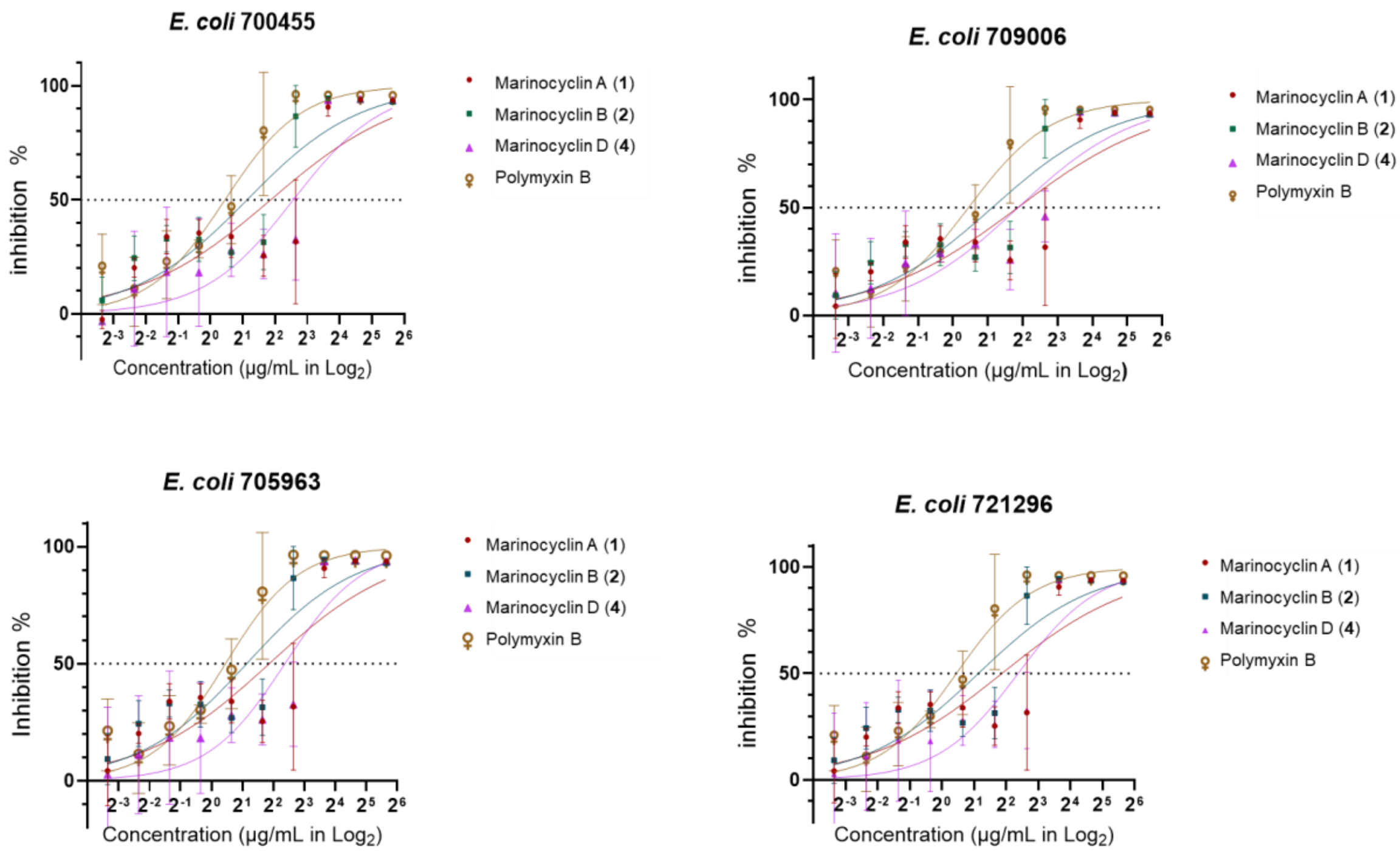

Figure S69. MIC determination of marinocyclins against *E. coli* clinical isolates.

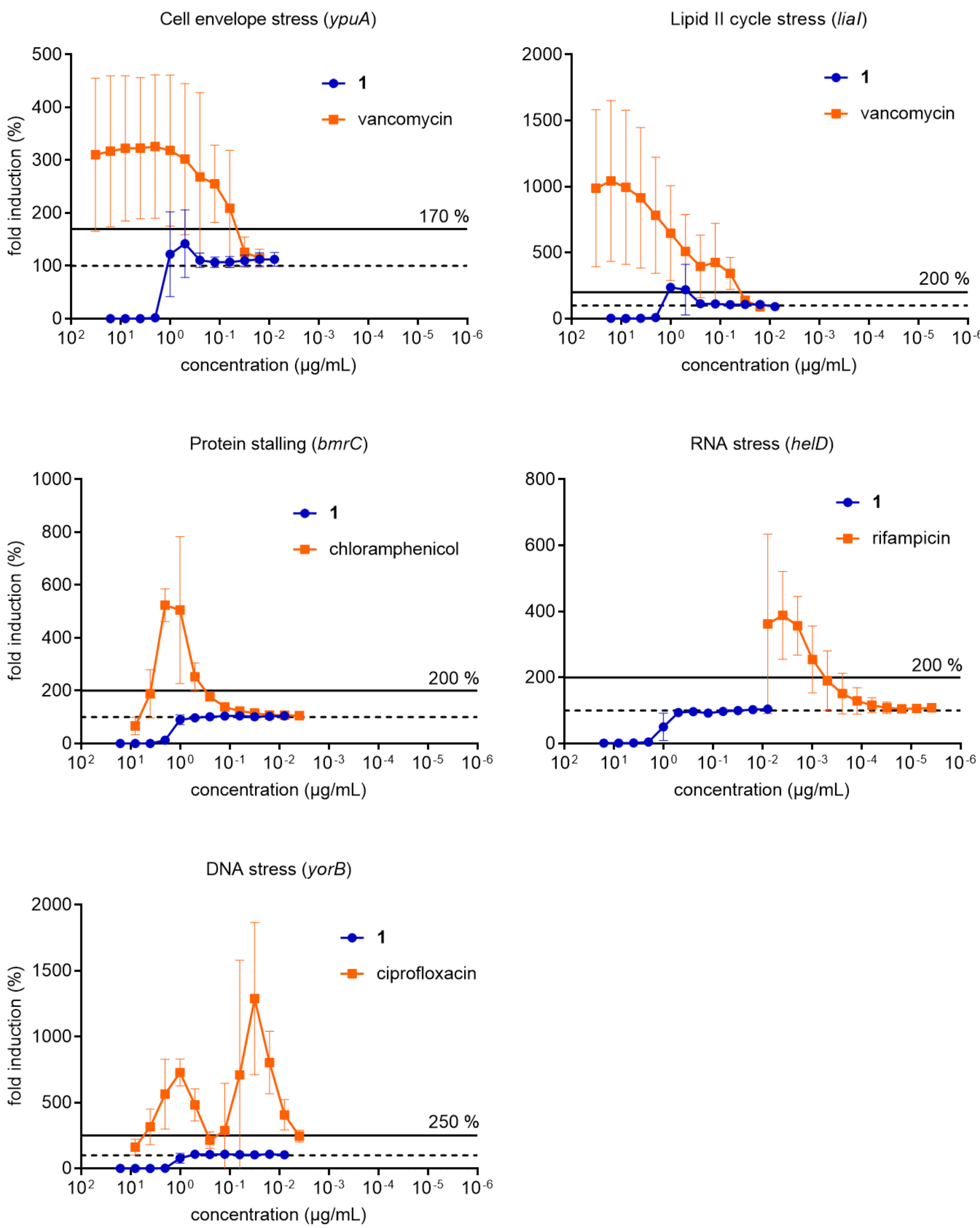

Figure S70. Marinocyclin A-treated *B. subtilis* senses cell envelope stress**.** Stress-response profiling of marinocyclin A (**1**) in isogenic *B. subtilis* 1S34 reporter strains. Promoter induction of pathways linked to cell envelope damage (*ypuA* reacting broadly to membrane damage and beta-lactams), "lipid **II**" cycle stress (*liaI* reacting to impaired C55-P turnover), ribosome stalling (*bmrC*), RNA synthesis inhibition (*helD*) and DNA stress (*yorB*) is shown as fold induction relative to untreated controls. Reference antibiotics serve as validated positive controls. Luciferase expression serves as readout. The lower induction level of marinocyclin A (**1**) compared to the positive controls is probably caused by its rapid membrane disrupting mechanism, which precludes protein synthesis.

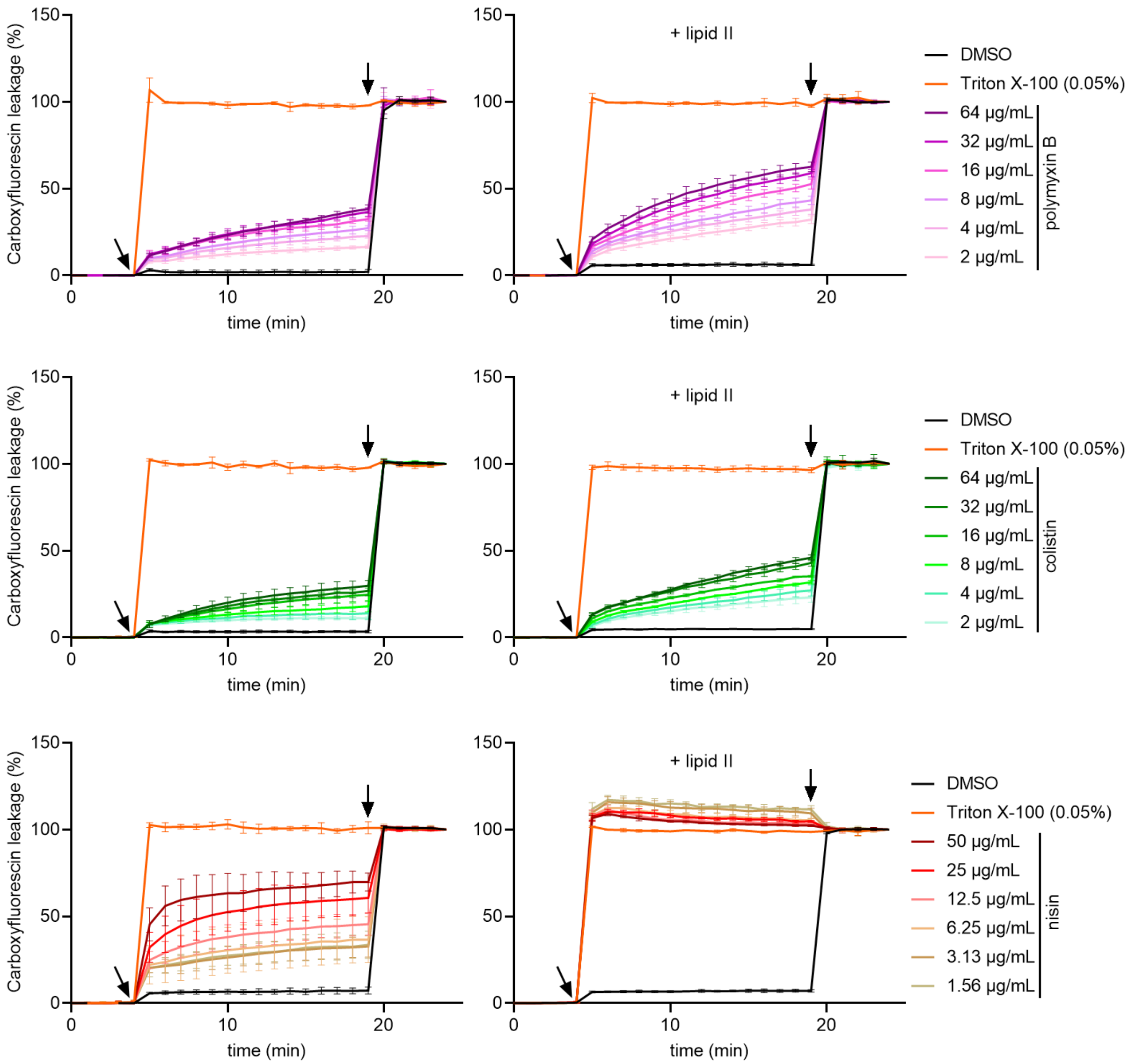

Figure S71. Permeabilization of large unilamellar vesicles (GUVs) resembling the phospholipid head group composition in *B. subtilis* membranes (+/- added 0.5% lipid II) by polymyxin B, colistin, and nisin. While in the vesicle lumen, concentrated carboxyfluorescein is self-quenched. The readout is based on the fluorescence increase of leaking carboxyfluorescein from compromised vesicles (n = 3, mean ± SD). First arrow, compound addition; second arrow, addition of Triton X-100 as internal positive control. DMSO (4%) as negative control.

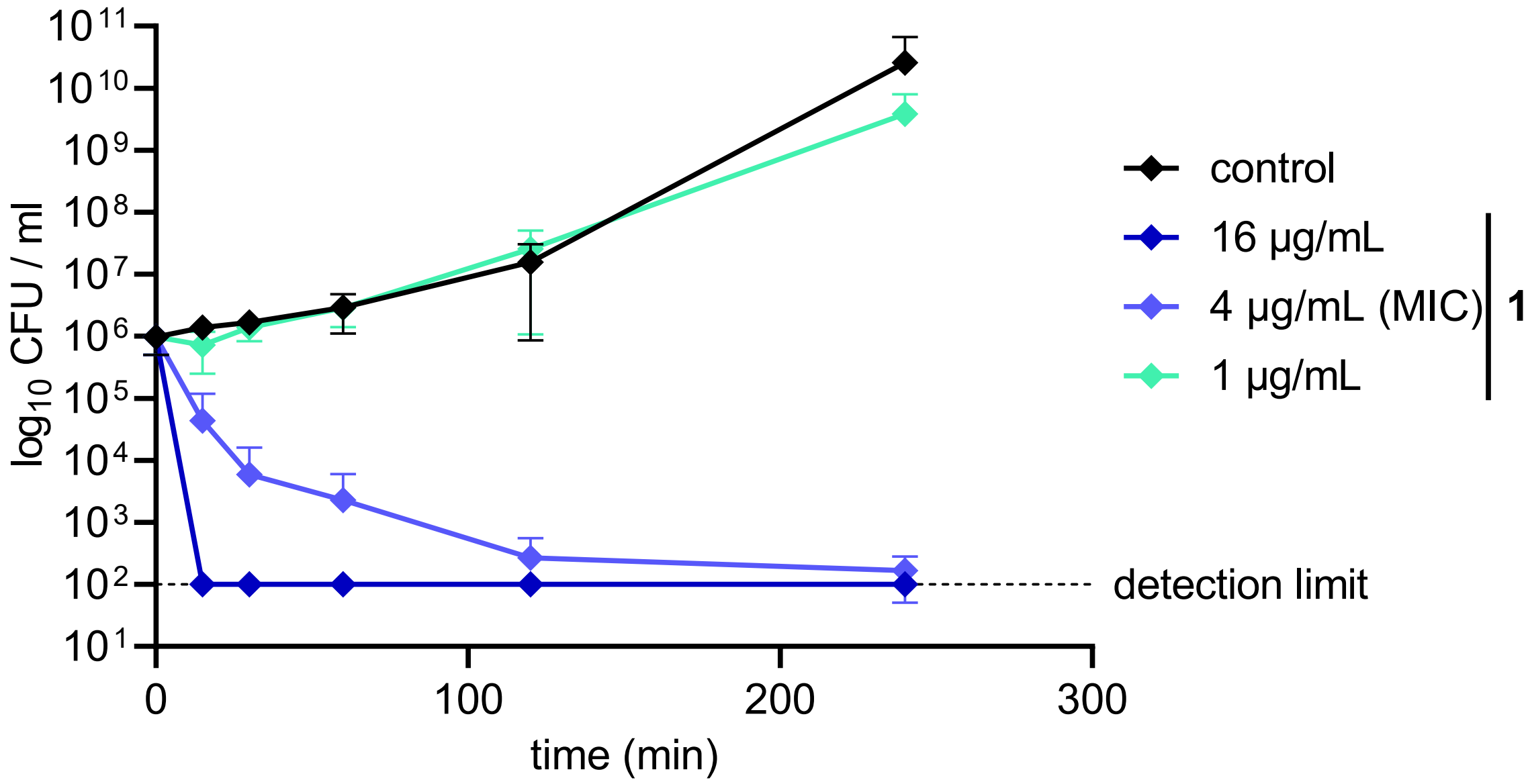

Figure S72. Time-kill curve of *S. aureus* ATCC 29213 treated with marinocyclin A (1) (n = 3, mean ± SD). At the MIC, marinocyclin is bactericidal (defined as 3log10 CFU reduction within 8 h). At 4x MIC, 15 min are sufficient to completely eradicate the culture down to the detection limit.

References

[1] T. Keller-Costa, D. Eriksson, J. M. S. Gonçalves, N. C. M. Gomes, A. Lago-Lestón, R. Costa, *FEMS Microbiol. Ecol*. **2017**, *93*, 143.

[2] J. F. Couceiro, T. Keller-Costa, N. C. Kyrpides, T. Woyke, W. B. Whitman, R. Costa, *Microbiol. Resour. Announc.* **2022**, *11*, 00620-00622.

[3] K. Blin, S. Shaw, L. Vader, J. Szenei, et al. *Nucleic Acids Res.* **2025**, *53*, 32-38.

[4] M. A. Skinnider, C. W. Johnston, M. Gunabalasingam, et al. *Nat Commun*. **2020**, *11*, 6058.

[5] M. Wang, J. J. Carver, V. V. Phelan, et al. *Nat Biotechnol*, **2016**, *34*, 828-837.

[6] K. Fujii, Y. Ikai, T. Mayumi, H. Oka, M. Suzuki, K. Harada, *Anal. Chem.* **1997**, *69*, 3346-3352.

[7] K. Fujii, Y. Ikai, T. Mayumi, H. Oka, M. Suzuki, K. Harada, *Anal. Chem.* **1997**, *69*, 5146–5151.

[8] Y. Li, L. Liu, G. Zhang, N. He, W. Guo, B. Hong, Y. Xie, *Front Chem.* **2020**, *8*,197.

[9] B. W. Miller, A. L. Lim, Y. Lin, et al. Haygood, *Cell Chem. Biol*. **2021**, *28*,1628-1637.

[10] M. Sarciaux, L. Pantel, C. Midrier, M. Serri, et al. *J. Med. Chem*. **2018**, *61*, 7814-7826.

[11] A. Magnusdottir, I. Johansson, L. G. Dahlgren, P. Nordlund, H. Berglund. *Nat. Methods.* **2009**, 6, 477-8.

[12] W. Jiang, R. A. Cacho, G. Chiou, N. K. Garg, Y. Tang, C. T. Walsh*. J. Am. Chem. Soc.* **2013**, 135, 4457-66.

[13] D. A. Wirtz, K. C. Ludwig, M. Arts, et al. *Angew. Chem. Int. Ed. Engl.* **2021**, 60, 13579-13586.

[14] C. Hermes, R. Richarz, D. A. Wirtz, et al. *Nat. Commun.* **2021**,12, 144.

[15] J. Kirschbaum, L. Bernd, B. Wolf-Dieter. *J. Chromatogr. A 661*. **1994**, 1-2, 193-199.

[16] CLSI standard M07. Clinical and Laboratory Standards Institute, Wayne, Pennsylvania, **2022**

[17] A. Urban, S. Eckermann, B. Fast, S. Metzger, M. Gehling, K. Ziegelbauer, H. Rübsamen-Waigmann, C. Freiberg, *Appl. Environ. Microbiol*. **2007**,*73*, 6436-43.

[18] R. E. Hancock, P. G. Wong, *Antimicrob. Agents Chemother*, **1984**, *26*, 48-52.

[19] S. P. Falk, A. T. Ulijasz, B. Weisblum, *J. Biomol. Screen*. **2007**, *12*, 1102-8.

[20] S. Clejan, T. A. Krulwich, K. R. Mondrus, D. Seto-Young. *J. Bacteriol.* 1986, 168, 334-40.

[21] C. Anagnostopoulos, J. Spizizen, *J. Bacteriol*. **1961**, *81*, 41-46.

[22] A. T. Ulijasz, A. Grenader, B. Weisblum, *J. Bacteriol*. **1996**, *178*, 6305-6309.

[23] B. A. Diep, S. R. Gill, R. F. Chang, et al. *Lancet*. **2006**, *367*, 731-739.

[24] C. Weidenmaier, A. Peschel, Y. Q. Xiong, S. A. Kristian, K. Dietz, M. R. Yeaman, S. A. Bayer*, J Infect Dis.* **2005**, 191, 1771-1777.

[25] C. M. Ernst, C. J. Slavetinsky, S. Kuhn, J. N. Hauser, M. Nega, N. N. Mishra, C. Gekeler, A. S. Bayer, A. Peschel. *mBio*. **2018**, 9, e01659-18.

[26] N. Woodford, A. M. Adebiyi, M. F. Palepou, B. D. Cookson, *Antimicrob. Agents Chemother*. **1998**, *42*, 502-508.

[27] G. Werner, I. Klare, W. Witte, *FEMS Microbiol. Lett*. **1997**, *155*, 55-61

[28] M. Tietgen, T. Semmler, S. Riedel-Christ, V. A. J. Kempf, A. Molinaro, C. Ewers, S. Göttig, *Int. J. Antimicrob. Agents*, **2019**, *53*, 554-561.

[29] T. Baba, T. Ara, M. Hasegawa, Y. Takai, Y. Okumura, M. Baba, K. A. Datsenko, M. Tomita, B. L. Wanner, H. Mori, *Mol. Syst. Biol*. **2006**, *2*, 2006-2008.

[30] D. A. Torres, H. M. B. Seth-Smith, N. Joosse, C. Lang, O. Dubuis, M. Nüesch-Inderbinen, V. Hinic, A. Egli, *BMC Microbiol*. **2021**, *21*, 321.

[31] B. Henrichfreise, I. Wiegand, K. J. Sherwood, B. Wiedemann, *Antimicrob. Agents Chemother*. **2005**, *49*, 1668-1669.

[32] K. Leeten, N. Jacques, L. A. Esquembre, et al. *mBio*. **2024**, 15, e0132224.
